# Gut Hormone Pharmacology of a Novel GPR119 Agonist (GSK1292263), Metformin, and Sitagliptin in Type 2 Diabetes Mellitus: Results from Two Randomized Studies

**DOI:** 10.1371/journal.pone.0092494

**Published:** 2014-04-03

**Authors:** Derek J. Nunez, Mark A. Bush, David A. Collins, Susan L. McMullen, Dawn Gillmor, Glen Apseloff, George Atiee, Leonor Corsino, Linda Morrow, Paul L. Feldman

**Affiliations:** 1 GlaxoSmithKline Research and Development, Research Triangle Park, North Carolina, United States of America; 2 Ohio State University Clinical Pharmacology Unit, Columbus, Ohio, United States of America; 3 Worldwide Clinical Trials, San Antonio, Texas, United States of America; 4 Duke University Medical Center, Durham, North Carolina, United States of America; 5 Profil Institute for Clinical Research, Chula Vista, California, United States of America; Glaxo Smith Kline, Denmark

## Abstract

GPR119 receptor agonists improve glucose metabolism and alter gut hormone profiles in animal models and healthy subjects. We therefore investigated the pharmacology of GSK1292263 (GSK263), a selective GPR119 agonist, in two randomized, placebo-controlled studies that enrolled subjects with type 2 diabetes. Study 1 had drug-naive subjects or subjects who had stopped their diabetic medications, and Study 2 had subjects taking metformin. GSK263 was administered as single (25–800 mg; n = 45) or multiple doses (100–600 mg/day for 14 days; n = 96). Placebo and sitagliptin 100 mg/day were administered as comparators. In Study 1, sitagliptin was co-administered with GSK263 or placebo on Day 14 of dosing. Oral glucose and meal challenges were used to assess the effects on plasma glucose, insulin, C-peptide, glucagon, peptide tyrosine-tyrosine (PYY), glucagon-like peptide-1 (GLP-1) and glucose-dependent insulinotropic peptide (GIP). After 13 days of dosing, GSK263 significantly increased plasma total PYY levels by ∼five-fold compared with placebo, reaching peak concentrations of ∼50 pM after each of the three standardized meals with the 300 mg BID dose. Co-dosing of GSK263 and metformin augmented peak concentrations to ∼100 pM at lunchtime. GSK263 had no effect on active or total GLP-1 or GIP, but co-dosing with metformin increased post-prandial total GLP-1, with little effect on active GLP-1. Sitagliptin increased active GLP-1, but caused a profound suppression of total PYY, GLP-1, and GIP when dosed alone or with GSK263. This suppression of peptides was reduced when sitagliptin was co-dosed with metformin. GSK263 had no significant effect on circulating glucose, insulin, C-peptide or glucagon levels. We conclude that GSK263 did not improve glucose control in type 2 diabetics, but it had profound effects on circulating PYY. The gut hormone effects of this GPR119 agonist were modulated when co-dosed with metformin and sitagliptin. Metformin may modulate negative feedback loops controlling the secretion of enteroendocrine peptides.

**Trial Registration:**

Clinicaltrials.gov NCT01119846 Clinicaltrials.gov NCT01128621

## Introduction

Glucagon-like peptide-1 (GLP-1) and glucose-dependent insulinotropic peptide (GIP) are important determinants of glucose disposal following a meal [1]. In type 2 diabetes mellitus (T2D), fasting and post-prandial plasma levels of GIP are normal or increased, but the β-cell response to this peptide is diminished. In contrast, β-cells remain responsive to the insulinotropic action of GLP-1, but meal-stimulated GLP-1 increases are diminished [2]. Enteroendocrine cells also secrete peptide tyrosine-tyrosine (PYY), a peptide implicated in the control of food intake [3]. Dipeptidyl peptidase-IV (DPP-IV) is the protease responsible for the rapid degradation of active GLP-1_7–36_ and GIP_1–42_ and for the conversion of PYY_1–36_ to PYY_3–36_ [4], [5]. Inhibitors such as sitagliptin increase circulating active GLP-1_7–36_ and improve glucose control in patients with T2D [6]. Metformin, a cornerstone in the treatment of patients with T2D, has been reported to alter circulating GLP-1 levels [7]–[9], in addition to its effects on hepatic glucose output and peripheral insulin sensitivity [10], [11].

GPR119 is a G protein-coupled receptor mainly found on enteroendocrine K and L cells and on pancreatic islets [12]. GPR119 agonists increase circulating GLP-1, GIP, and PYY in animal models and healthy humans [13], [14], and they improve glucose metabolism in nonclinical models of T2D. GSK1292263 (GSK263) (5-[({1-[3-(1-methylethyl)-1,2,4-oxadiazol-5-yl]-4-piperidinyl}methyl)oxy]-2-[4-(methylsulfonyl)phenyl]pyridine) is a potent and selective agonist at the rodent and human GPR119 receptors that was discovered at GlaxoSmithKline [15]. It has a pEC_50 = _6.8 for human, rat and mouse GPR119 receptors expressed in an *in vitro* reporter assay, and a pEC_50 = _8.5 for the stimulation of GLP-1 secretion from GLUTag cells. The potential for GSK263 to have off-target interactions was evaluated in 2 ways *in vitro*: (i) when GSK263 was tested in a GSK-customized battery of 52 assays from the Panlabs panel of assays (MDS Pharma Services, King of Prussia, PA), no result met the significance criteria (>50% of maximum stimulation or inhibition) at a concentration of 10 µM of GSK263; (ii) there were no instances of >20% inhibition of binding when GSK263 was tested at a concentration of 1 µM against 50 assays from a Cerep panel (Cerep, Seattle, WA). Like other GPR119 agonists, GSK263 increases glucose-sensitive insulin secretion, improves glucose tolerance and enhances the secretion of gut hormones in normal rats (GlaxoSmithKline, unpublished data). Unlike some GPR119 agonists [16], GSK263 does not alter gastric emptying, reduce food intake, or reduce weight in rats. In healthy subjects, there was a trend for the highest single doses of GSK263 to reduce glucose and insulin excursions after an oral glucose load, while the total GLP-1 and GIP responses were modestly augmented (GlaxoSmithKline, unpublished data). Compared to sitagliptin, single doses of GSK263 increased PYY concentrations progressively through the day and for several hours beyond the evening meal, returning to baseline levels overnight.

Because of the glycemic and gut peptide effects of GSK263 in healthy subjects, we conducted two studies with subjects with T2D to investigate the pharmacology of GSK263 and the effects of co-administration with metformin or sitagliptin, 2 drugs that alter circulating gut hormone levels.

## Research Design and Methods

### Ethics statement

The two studies (www.clinicaltrials.gov: NCT01128621 and NCT01119846) were conducted with subjects with T2D in accordance with ICH Good Clinical Practice guidelines [17], subject privacy requirements, and the principles of the Declaration of Helsinki [18]. Seven sites participated in Study 1 (Elite Research Institute, Miami, FL; Profil Institute for Clinical Research, Inc, Chula Vista, CA, USA; ICON Development Solutions, San Antonio, TX, USA; Ohio State University Clinical Pharmacology Unit, Columbus, OH, USA; Duke University Clinical research Unit, Durham, NC, USA; Dedicated Phase I, Phoenix, AZ, USA; Charles River Clinical Services Northwest, Tacoma, WA, USA), and 2 sites in Study 2 (Elite Research Institute and Profil Institute for Clinical Research, Inc).

The study protocols were approved either by the Independent Investigational Review Board Inc. (Plantation, FL, USA), the Western IRB (Olympia, WA, USA), or the Copernicus Group IRB (Durham, NC, USA). All subjects provided written informed consent before enrolment in these studies.

### Study designs

The protocols for these trials and the supporting CONSORT checklist are available as supporting information; see Checklist S1 and Protocols S1 and Protocol S2.

Study 1 was conducted in 3 parts (first subject, first visit: 5 June 2009; last subject, last visit: 19 March 2010):

- Part A was a single-dose, double-blind (sponsor unblind), randomized, 5-period crossover study that enrolled drug-naïve (diet and exercise treatment only) subjects with T2D. The study treatments were: GSK263 (25 mg, 150 mg, and 800 mg), placebo and open-label 100 mg sitagliptin. The treatments were administered 2 h before an oral glucose tolerance test (OGTT) so that the glucose drink occurred approximately at the time of maximum GSK263 concentrations in the circulation (T_max_).
- Part B was a single-dose, double-blind (sponsor unblind), randomized, 2-period study that enrolled subjects with T2D who stopped prior pharmacological therapy for T2D 1 week before dosing GSK263. GSK263 800 mg was administered in a fasting state and with food after eating breakfast to determine what effect food might have on the bioavailability of the drug. As food increased the systemic concentrations of GSK263, in Part C the drug was give just after a meal. The data from Parts A and B were used for pharmacokinetic/pharmacodynamic (PK/PD) simulations to select the doses for Part C.
- Part C was a 14-day repeat-dose, double-blind (sponsor unblind), randomized study that enrolled subjects with T2D who stopped their usual diabetes drug therapy for 1 week prior to receiving the study drug. There were 6 treatment arms: GSK263 (BID doses of 50 mg, 150 mg, and 300 mg or a QD dose of 600 mg), placebo, or 100 mg open-label sitagliptin, all dosed immediately after a meal. Sitagliptin was included as an active comparator to assess the robustness of the glycemic endpoints. As GSK263 was known to increase GLP-1 in animal models and healthy subjects, a single 100 mg open-label dose of sitagliptin was co-administered on Day 14 to the subjects who were randomized to the GSK263 and placebo arms to investigate the effects of DPP-IV inhibition on the pharmacology of GSK263.

Study 2 was conducted in 2 parts (first subject, first visit: 23 November 2009; last subject, last visit: 12 April 2010):

- Part A was a single-dose, unblinded, 1 period study that enrolled subjects with T2D on metformin (≥1000 mg/day). A single 300 mg dose of GSK263 was co-administered with metformin to determine if there would be a PK interaction between GSK263 and metformin. The data were used for PK/PD simulations to select doses for Part B.
- Part B was a 14-day repeat-dose, double-blind (sponsor unblind), randomized study that enrolled subjects with T2D taking metformin ≥1000 mg/day. The study had five treatment arms (co-administered with metformin): GSK263 (BID doses of 75 mg and 300 mg or a QD dose of 600 mg), placebo or open-label 50 mg BID of sitagliptin, all dosed immediately after a meal. Sitagliptin was included as an active control to assess the robustness of the glycemic parameters.

Randomization codes and treatment assignments were generated by GlaxoSmithKline using a computer-based program.

Further details of study inclusion and exclusion criteria, procedures and endpoints are summarized in Tables 1 and 2.

**Table 1:** Main inclusion and exclusion criteria at Screening.

**Study specific inclusion criteria at screening**	
**Study 1** (NCT01119846; GPR111598)	**Study 2** (NCT01128621; GPR113132)
Type 2 diabetes diagnosed by ADA criteria (HbA1c from 6.5–11% [48–97 mmol/mol] for Part A and 7.0–11% [53–97 mmol/mol] for Parts B and C). Age: 18–60 years. BMI: 21.8–35.2 kg/m^2^, inclusive.	Type 2 diabetes diagnosed by ADA criteria (HbA1c from 6.5–11% [48–97 mmol/mol]) taking metformin monotherapy. Age:18–65 years. BMI: 21.8–37.5 kg/m^2^, inclusive.
**General inclusion and exclusion criteria at screening**	
**Inclusion**	Male or female subjects of non-childbearing potential. No significant known medical conditions other than type 2 diabetes, as determined by the investigators.
**Exclusion**	Prior use of insulin. Plasma triglycerides >450 mg/dL. An estimated glomerular filtration rate <60 mL/min by the MDRD equation† or proteinuria.

† www.MDRD.com.

**Abbreviations**: ADA, American Diabetes Association; BMI, Body Mass Index; HbA1c, Haemoglobin A1c; OGTT, Oral Glucose Tolerance Test.

**Table 2:** Procedures and endpoints in Studies 1 and 2.

**Procedures**
For the single-dose parts, subjects were admitted to the research facility within 24 h prior to the dose period and remained in the unit for 24 h post-dosing. In the repeat-doses parts, subjects were admitted to the research facility within 48 h prior to the dose period and remained in the unit for 24 h post-dosing, and were fed standardized meals (∼2300 kcal; ∼60% carbohydrate, 20% fat, and 20% protein) on the PD profiling days.
For the OGTT, blood samples were drawn 10 min before, just prior to the consumption of the 75 g glucose beverage, and at 10, 20, 30, 60, 90, 120, and 180 minutes after the drink. For meals, samples were drawn fasting and for 3 h after breakfast, lunch and dinner. A limited post-prandial profile was obtained on Day 7. Multiple blood samples were collected for GSK263 and sitagliptin concentrations on Days 1, 13, and 14 in Study 1, and for GSK263, sitagliptin and metformin on Days −1, 1, and 14 in Study 2.
All subjects had a follow-up visit 7–10 days following discharge from the clinical unit.
**Endpoints**
(i) Safety and tolerability parameters including adverse events, clinical laboratory parameters, ECGs and vital signs. In Study 1, adverse events were collected from the start of the washout of prior diabetes medications to the follow-up contact, while in Study 2 they were collected from Day 1 of dosing to the end of the confinement period. Adverse events were graded as mild (Grade 1), moderate (Grade 2), severe (Grade 3), life-threatening (Grade 4) or death (Grade 5) (ii) Pharmacokinetic parameters of GSK263, metformin and sitagliptin. (iii) Fasting and OGTT- and standardized meal-related weighted-mean AUC for glucose, insulin, glucagon, GLP-1 (active and total), C-peptide, total GIP, and total PYY. (iv) Fasting and OGTT- and meal-derived minimal model measures of insulin sensitivity and beta-cell function. (v) Hunger, Craving, and Fullness Questionnaire responses and calorie counts at baseline and end of treatment.

Unless indicated, investigators, subjects and site staff (except for the unblinded pharmacist) were blinded to the allocation of study treatment. The GlaxoSmithKline study team had unblinded access to the data during the course of the study.

The clinical execution of these studies was funded by GlaxoSmithKline Research and Development, Research Triangle Park, NC, USA.

### Assays

Venous blood samples were collected for PD and PK analysis in K^+^-EDTA tubes and rapidly placed on ice until centrifuged at 4°C for 10 min. Plasma was stored at −70°C until analyzed. Samples for clinical chemistry and hematology were measured by the local certified laboratory.

#### PD assays

Plasma samples were analyzed for glucose, insulin, C-peptide, glucagon, total and active GLP-1_7–36_, total GIP and total PYY by BioAgilytix Labs (Durham, NC) using the assays from MesoScale Discovery (Gaithersburg, MD), Millipore (Billerica, MA) and Yellow Springs Instrument Co (Yellow Springs, OH), as shown in Table 3.

**Table 3:** Glucose and peptide assays used in these studies.

Assay	Manufacturer	Catalog Number	Notes on the Assay
**Active GLP-1**	Meso Scale Discovery	K151HZC	Electrochemiluminescent multiplex immunoassay. Standard curve range was 3.13–200 pg/mL
**Total GLP-1**	Meso Scale Discovery	Multiplex assay with glucagon and insulin, catalog number K15160C	Electrochemiluminescent multiplex immunoassay. Standard curve range was 3.13–200 pg/mL
**PYY**	Millipore	EZHPYYT66K	Sandwich enzyme-linked immunosorbent assay. Standard curve range was 40–2000 pg/mL
**GIP**	Millipore	EZHGIP-54K	Sandwich enzyme-linked immunosorbent assay. Standard curve range was 7.18–1000 pg/mL
**Glucagon**	Meso Scale Discovery	Multiplex assay with total GLP-1 and insulin, catalog number K15160C	Electrochemiluminescent multiplex immunoassay. Standard curve range was 25–1600 pg/mL
**C-peptide**	Millipore	EZHCP-20K	Sandwich enzyme-linked immunosorbent assay. Standard curve range was 0.2–20 ng/mL
**Insulin**	Meso Scale Discovery	Multiplex assay with total GLP-1 and glucagon, catalog number K15160C	Electrochemiluminescent multiplex immunoassay. Standard curve range was 22.9–16,667 pg/mL
**Glucose (2300 STAT Plus Glucose Analyzer)**	YSI	Glucose/lactate standard, catalog number 2747	Automated instrument for measuring glucose concentration in plasma or serum

#### PK Assays

GSK263 concentrations were measured as described previously [19]. The concentrations of sitagliptin and metformin were analyzed using validated analytical methods (GlaxoSmithKline, data on file).

### Statistical methods

Sample sizes for all parts were based primarily on feasibility considerations because of the exploratory nature of the studies.

Analyses were performed using SAS, version 8.02 (SAS Institute, Cary, NC). The difference in change from baseline of weighted-mean (WM) area under the plasma-concentration time curve (AUC) PD parameters between groups was examined using an analysis of covariance (ANCOVA) with terms for treatment and baseline measurement. Time-averaged, weighted-mean AUCs were calculated by dividing the area by the time interval.

A validated Hunger, Craving, and Fullness questionnaire (GlaxoSmithKline data on file), calorie counts and weight at baseline (Day −1) and end-of-treatment were used to investigate the effects of GSK263 on food intake.

Minimal model parameters of insulin sensitivity (based on glucose concentrations) and pancreatic beta cell function (based on C-peptide concentrations) [20] were estimated using NONMEM modeling software (ICON, Ellicott City, MD).

PK parameters were calculated by standard noncompartmental methods using WinNonlin Pro Version 5.2.

## Results

In total, 173 subjects were enrolled in the 2 studies, and 158 completed the study. Subject disposition and baseline demographic characteristics are summarized in Table 4 and Table 5, respectively.

**Table 4:** Disposition of the subjects in Studies 1 and 2.

	Study 1			Study 2	
	Part A	Part B	Part C	Part A	Part B
**Number of subjects**					
Enrolled/randomized, N:	13	4	83	6	67
Completed, N (%):	11 (85)	4 (100)	72 (87)	6 (100)†	65 (97)
Withdrawn (any reason), N (%):	2 (15)	0	11 (13)	0	2 (3)
**Reasons for subject withdrawal, N (%)**					
AEs	1 (8)		6 (7)		
Protocol deviation	1 (8)		1 (1)		
Consent withdrawn			4 (5)		
Withdrawal criterion for the electrocardiographic PR interval criterion specified in the protocol (>220 msec) was exceeded by 1 and 4 msec, respectively					2 (3)

† Number of subjects included in PK and PD population (N (%)): 4 (67%). Samples from 2 subjects were unusable because they had thawed in transit to the analytical laboratory.

**Abbreviations:** AE, Adverse Event.

**Table 5:** Baseline demographics of the subjects in Studies 1 and 2.

	Study 1			Study 2	
	Part A	Part B	Part C	Part A	Part B
**Age**, (yrs), mean (SD)	48.0 (7.46)	49.0 (6.06)	51.7 (7.18)	57.0 (3.85)	54.1 (7.29)
**Sex**, n (%)					
Female:	4 (31)	1 (25)	22 (27)	1 (17)	28 (42)
Male:	9 (69)	3 (75)	61 (73)	5 (83)	39 (58)
**BMI (kg/m^2^)**, mean (SD)	29.21 (3.417)	29.28 (3.836)	29.76 (3.223)	28.27 (4.152)	31.20 (3.520)
**HbA1c (%)**, mean (SD), [mmol/mol]	7.47 (1.04) [58]	7.75 (1.34) [61]	8.50 (0.95) [69]	7.82 (0.91) [62]	8.52 (0.93) [70]
**Prior therapy, n**					
Drug Naive (diet and exercise)	13†	3	65		
Metformin monotherapy		1	14	6	67
Sulphonylurea monotherapy			4		

† All subjects were supposed to be on diet and exercise treatment, but 1 subject reported the use of anti-diabetic medications after enrolling in the study.

**Abbreviations:** AE, Adverse Event; BMI, Body Mass Index; HbA1c, Hemoglobin A1c; SD, Standard Deviation.

### Effects on gut hormones

#### Single doses of GSK263 or sitagliptin

The peptide effects of single doses of GSK263 and sitagliptin administered alone prior to an OGTT were similar to the repeat-dose data reported below. Total PYY concentrations increased progressively through the day, reaching a peak of ∼55 pM during the dinner meal after a single 800 mg dose of GSK263, and then falling to pre-dose levels by the next morning.

Although the fed-fasted paradigm was intended to investigate the impact of food on GSK263 bioavailability, we noted that a single dose of GSK263 only had a modest effect on circulating gut hormone levels in the fasted state, whereas secretion of these hormones was markedly increased when GSK263 was taken with food at breakfast (data not shown).

#### Repeat doses of GSK263 or sitagliptin

**Figure 1:**
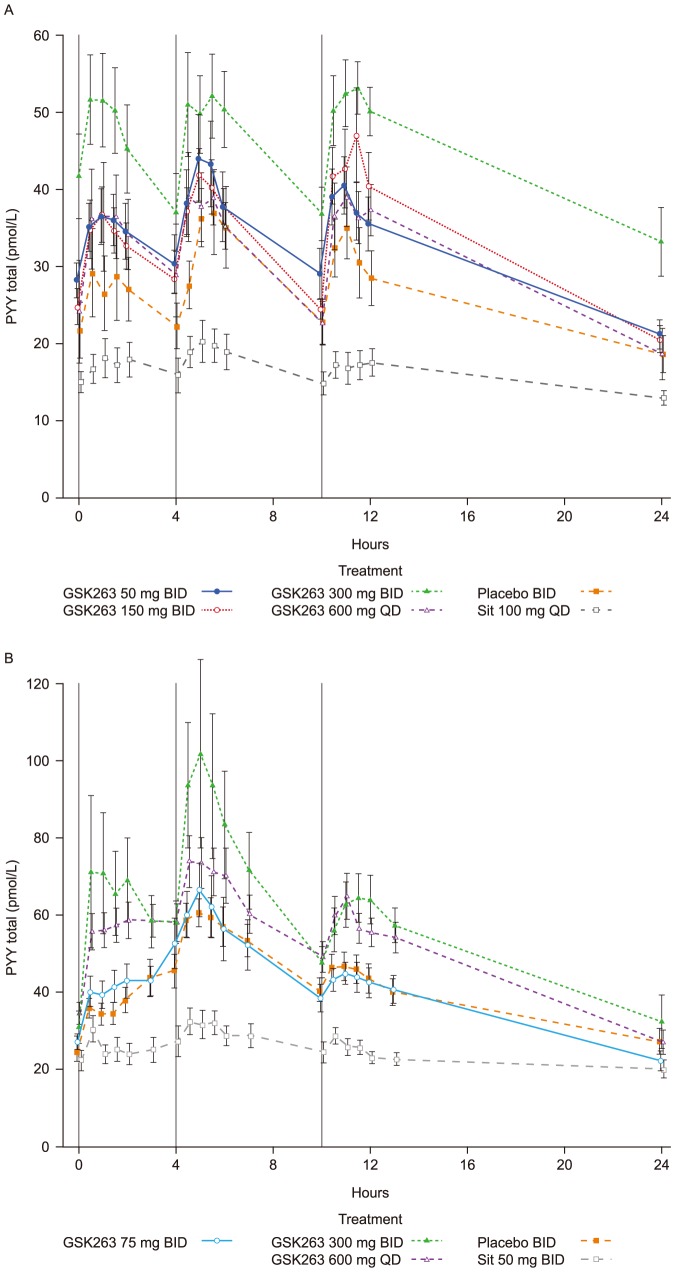
Plasma concentrations of total PYY in Study 1 following 13 days of dosing GSK263, placebo or sitagliptin (A), and in Study 2 on Day 14 of co-dosing these study treatments with metformin (B). The vertical black lines indicate the times of the meals. Values are Means (pmol/L) ± SEM.

After repeated doses of GSK263, the most robust pharmacodynamic response was observed in the 24 h profiles of total PYY which were increased by Day 7, and remained elevated to the end of treatment. Remarkably, peak postprandial PYY values reached ∼50 pM with GSK263 alone (Figure 1A), and increased further when it was co-dosed with metformin, reaching peak levels ∼70–100 pM (Figure 1B). The PYY profiles observed when GSK263 was co-dosed with sitagliptin on Day 14 in Study 1, were suppressed and similar to those observed with sitagliptin alone (data not shown).

The change from baseline of PYY WM-AUCs (±95% Confidence Interval, CI) are summarized in Tables 6 and 7. All BID doses of GSK263 significantly increased WM-AUC_(0–24 h)_ of PYY by ∼25%, while the 600 mg QD dose of GSK263 increased WM-AUC_(0–24 h)_ by ∼16%, and significantly increased WM-AUC_(0–12 h)_ by ∼29%.

**Table 6:** Percent change from baseline WM-AUC_(0–24 h)_ for the gut peptides following repeat-dose administration in Studies 1 and 2.

Study	Day	Regimen	Total GLP-1	Active GLP-1_7–36_	Total PYY	Total GIP
			Mean (95% CI)			
1	13	Placebo BID	−5.12 (−14.41, 4.16)	−2.02 (−11.82, 7.78)	5.08 (−11.90, 22.05)	4.30 (−11.56, 20.15)
1	13	50 mg GSK263 BID	15.65 (−7.02, 38.31)	0.17 (−1.02, 1.35)	26.44 (10.46, 42.42)	17.65 (−4.11, 39.41)
1	13	150 mg GSK263 BID	−14.96 (−26.97, −2.94)	0.15 (−1.98, 2.28)	27.01 (3.05, 50.98)	15.67 (−0.31, 31.64)
1	13	300 mg GSK263 BID	−2.98 (−12.78, 6.82)	−1.70 (−11.62, 8.23)	25.42 (3.25, 47.59)	−6.46 (−18.28, 5.37)
1	13	600 mg GSK263 QD	4.69 (−20.01, 29.39)	0.39 (−3.36, 4.15)	16.88 (−2.04, 35.80)	−0.06 (−12.14, 12.03)
1	13	Sitagliptin 100 mg QD	−16.93 (−31.07, −2.78)	154.82 (75.84, 233.80)	−22.90 (−31.71, −14.09)	−20.04 (−27.38, −13.50)
1	14	Placebo BID + Sitagliptin 100 mg QD	−14.26 (−26.40, −2.12)	139.26 (96.20, 182.32)	−23.75 (−36.50, −11.00)	−12.93 (−24.10, −1.77)
1	14	50 mg GSK263 BID + Sitagliptin 100 mg QD	−3.27 (−20.85, 14.32)	164.89 (126.85, 202.92)	−14.68 (−25.92, −3.44)	−0.69 (−23.84, 22.45)
1	14	150 mg GSK263 BID + Sitagliptin 100 mg QD	−21.66 (−33.31, −10.01)	155.43 (92.11, 218.75)	−10.72 (−25.14, 3.69)	1.50 (−10.89, 13.88)
1	14	300 mg GSK263 BID + Sitagliptin 100 mg QD	−13.68 (−20.22, −7.13)	241.07 (176.27, 305.88)	−14.05 (−33.90, 5.81)	−25.34 (−35.20, −15.49)
1	14	600 mg GSK263 QD + Sitagliptin 100 mg QD	−12.61 (−39.25, 14.03)	146.31 (103.16, 189.46)	−22.51 (−30.17, −14.85)	−15.26 (−28.42, −2.09)
1	14	Sitagliptin 100 mg QD	−16.42 (−32.69, −0.14)	169.01 (111.99, 226.03)	−30.13 (−38.15, −22.11)	−11.42 (−24.85, 2.02)
2	14	Placebo BID	7.64 (−13.89, 29.18)	−10.27 (−25.96, 5.41)	8.71 (−5.64, 23.06)	7.66 (−8.54, 23.86)
2	14	75 mg GSK263 BID	20.34 (6.53, 34.16)	9.09 (1.71, 16.41)	24.84 (8.95, 40.73)	13.28 (0.29, 26.28)
2	14	300 mg GSK263 BID	6.98 (−3.77, 17.73)	−1.73 (−11.00, 7.54)	20.62 (3.67, 37.57)	5.85 (−4.68, 16.38)
2	14	600 mg GSK263 QD	18.10 (−8.39, 44.60)	3.54 (−7.77, 14.85)	20.23 (4.23, 36.23)	7.39 (−6.36, 21.13)
2	14	Sitagliptin 50 mg BID	1.54 (−13.43, 16.51)	400.65 (305.52, 495.79)	−35.01 (−44.39, −25.62)	−0.57 (−29.26, 28.12)

**Abbreviations**: CI, Confidence Interval; GIP, Glucose-dependent Insulinotropic Peptide; GLP-1, Glucagon-Like Peptide-1; PYY, Peptide Tyrosine-Tyrosine; PD, Pharmacodynamics; WM-AUC_(0–24 h)_, Weighted-Mean Area Under the plasma-Concentration time curve time zero to 24 h.

**Table 7:** Percent change from baseline WM-AUC_(0–12 h)_ for the gut peptides following repeat-dose administration in Studies 1 and 2.

Study	Day	Regimen	Total GLP-1	Active GLP-1_7–36_	Total PYY	Total GIP
			Mean (95% CI)			
1	13	Placebo BID	−4.73 (−14.80, 5.35)	−1.51 (−12.44, 9.42)	0.96 (−17.94, 19.87)	−3.64 (−18.60, 11.33)
1	13	50 mg GSK263 BID	19.68 (−7.18, 46.54)	0.31 (−2.35, 2.96)	32.37 (13.30, 51.45)	20.58 (10.72, 40.45)
1	13	150 mg GSK263 BID	−12.03 (−26.99, 2.93)	0.20 (−5.00, 5.40)	22.74 (1.51, 43.98)	26.07 (10.03, 42.11)
1	13	300 mg GSK263 BID	−4.54 (−21.45, 12.37)	−3.85 (−14.53, 6.83)	25.67 (−3.67, 55.01)	−4.14 (−16.47, 8.20)
1	13	600 mg GSK263 QD	0.30 (−22.82, 23.41)	2.28 (−4.62, 9.17)	29.00 (−10.39, 68.38)	6.54 (−5.28, 18.35)
1	13	Sitagliptin 100 mg QD	−18.15 (−30.54, −5.76)	163.64 (85.71, 241.56)	−24.91 (−36.60, −13.23)	−14.01 (19.91, −8.12)
1	14	Placebo BID + Sitagliptin 100 mg QD	−16.35 (−31.76, −0.94)	169.49 (109.09, 229.88)	−21.95 (−35.81, −8.09)	−17.43(−30.58, −4.28)
1	14	50 mg GSK263 BID + Sitagliptin 100 mg QD	0.81 (−21.08, 22.70)	216.25 (167.82, 264.68)	−12.03 (−28.79, 4.72)	1.40 (−16.30, 19.11)
1	14	150 mg GSK263 BID + Sitagliptin 100 mg QD	−22.61 (−33.83, −11.39)	182.24 (110.53, 253.95)	−7.75 (−25.11, 9.61)	6.20 (−7.66, 20.06)
1	14	300 mg GSK263 BID + Sitagliptin 100 mg QD	−13.84 (−23.94, −3.73)	284.17 (214.14, 354.20)	−11.36 (−31.89, 9.17)	−24.36 (−34.51, −14.22)
1	14	600 mg GSK263 QD + Sitagliptin 100 mg QD	−10.66 (−37.52, 16.21)	192.16 (146.83, 237.50)	−10.36 (−34.35, 3.62)	−14.17 (−27.47, −0.87)
1	14	Sitagliptin 100 mg QD	−14.79 (−34.95, 5.36)	197.11 (124.65, 269.57)	−31.28 (−41.44, −21.12)	−7.35 (−21.89, 7.20)
2	14	Placebo BID	7.79 (−14.82, 30.39)	−5.23 (−20.41, 9.95)	9.46 (−5.65, 24.57)	9.90 (−4.06, 23.86)
2	14	75 mg GSK263 BID	24.63 (10.85, 38.40)	8.50 (0.31, 16.69)	38.41 (19.97, 56.84)	16.91 (2.17, 31.65)
2	14	300 mg GSK263 BID	11.91 (1.86, 21.95)	−0.17 (−11.09, 10.75)	31.30 (10.11, 52.49)	12.00 (−0.43, 24.43)
2	14	600 mg GSK263 QD	14.08 (−2.86, 31.01)	6.34 (−6.30, 18.97)	29.04 (12.79, 45.29)	7.90 −5.13, 20.92)
2	14	Sitagliptin 50 mg BID	−1.15 (−14.72, 12.42)	481.94 (374.17, 589.70)	−36.11 (−44.04, −28.17)	−1.73 (−26.93, 23.47)

**Abbreviations**: CI, Confidence Interval; GIP, Glucose-dependent Insulinotropic Peptide; GLP-1, Glucagon-Like Peptide-1; PYY, Peptide Tyrosine Tyrosine; PD, Pharmacodynamics; WM-AUC_(0–12)_, Weighted-Mean Area Under the plasma-Concentration time curve time zero to 12 h.

Sitagliptin significantly reduced WM-AUC_(0–24 h)_ and WM-AUC_(0–12 h)_ of total PYY by ∼25–36%. The total PYY (and total GLP-1 and total GIP) profiles observed when GSK263 was co-dosed with sitagliptin on Day 14 in Study 1 were less suppressed than the levels observed with sitagliptin alone (Tables 6 and 7). Figure 1B suggests that co-administration of sitagliptin with metformin reduced the degree of suppression of total PYY compared to that observed in Study 1 when sitagliptin was administered alone (compare with Figure 1A), but this was not confirmed by the WM-AUC_(0–12 h)_ or WM-AUC_(0–24 h)_ values.

**Figure 2:**
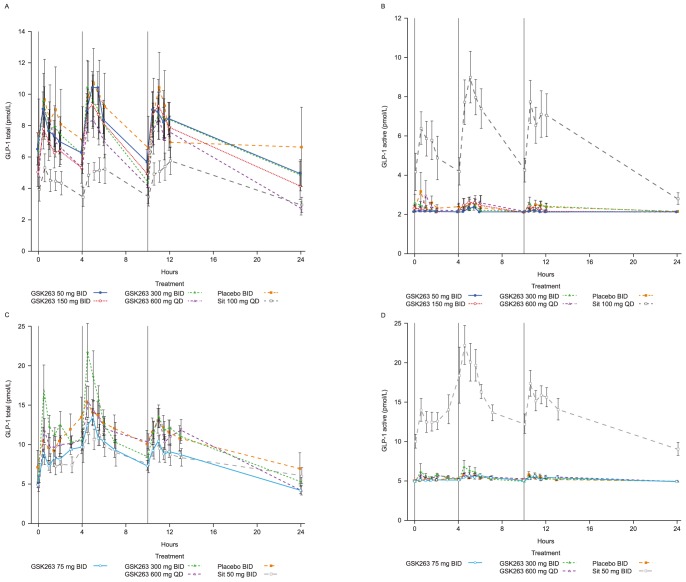
Effects of GSK263 and sitagliptin on plasma total and active GLP-1. Plasma concentrations of total GLP-1 in Study 1 following 13 days of dosing GSK263, placebo or sitagliptin (A), and in Study 2 following 14 days of co-administration of GSK263, placebo or sitagliptin with metformin (C). Plasma concentrations of active GLP-1 in Study 1 following 13 days of dosing (B), and in Study 2 after 14 days of dosing (D). The vertical black lines indicate the times of the meals. Values are Means (pmol/L) ± SEM.

GSK263 alone or with metformin had no significant effect on total (Figures 2A and 2C) or active GLP-1_7–36_ levels (Figures 2B and 2D). In contrast, sitagliptin significantly increased WM-AUC of active GLP-1_7–36_ by 155–160%, and reduced WM-AUC of total GLP-1 by ∼17–18% and GIP by ∼14% (Tables 6 and 7). Metformin alone increased total GLP-1 levels slightly (compare placebo data in Figures 2A and 2C and Tables 6 and 7), but it had no additional effect on active GLP-1_7–36_ (compare Figures 2B and 2D). Metformin augmented the increase in active GLP-1_7–36_ observed with sitagliptin (WM-AUC_(0–12 h)_ increased by ∼400%), while at the same time blunting the reduction of total GLP-1 observed when sitagliptin was dosed alone in Study 1 (WM-AUC_(0–12 h)_ increased by ∼1.5).

**Figure 3:**
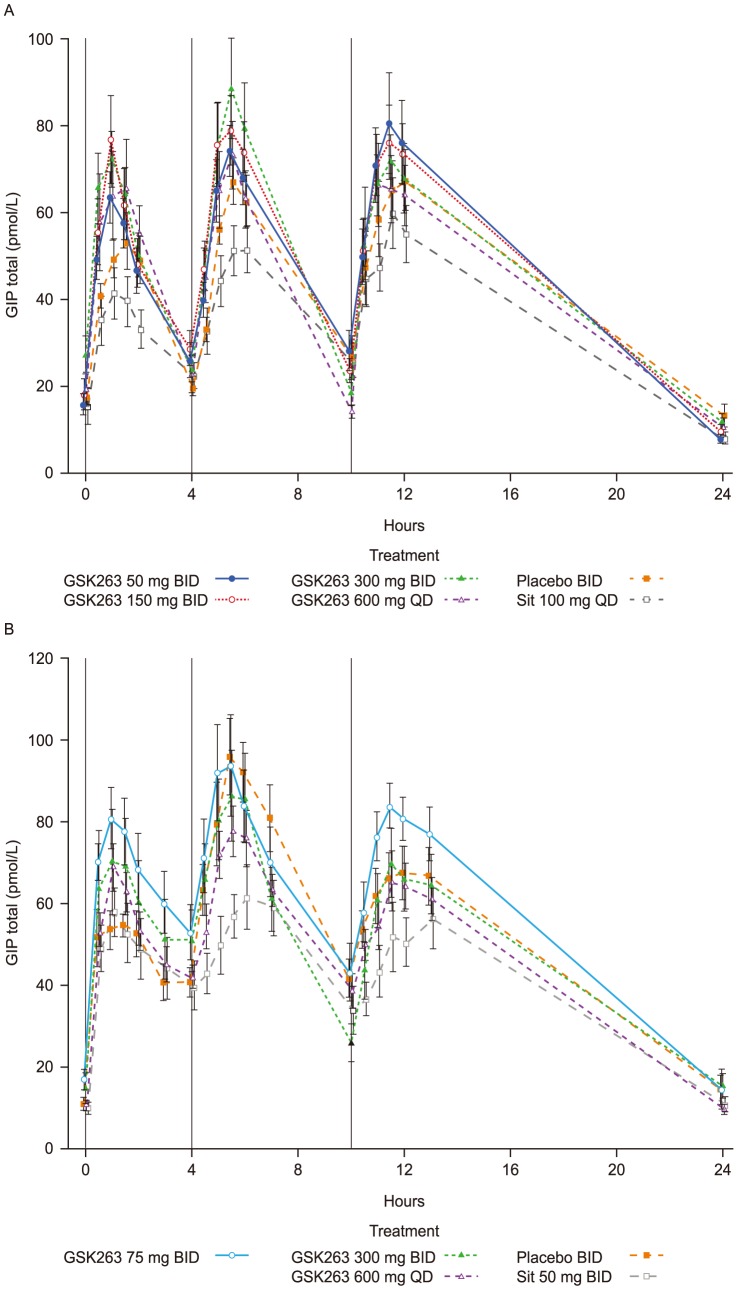
Effects of GSK263 and sitagliptin on plasma total GIP. Plasma concentrations of total GIP in Study 1 following 13 days of dosing GSK263, placebo or sitagliptin (A), and in Study 2 on Day 14 of co-dosing these study treatments with metformin (B). The vertical black lines indicate the times of the meals. Values are Means (pmol/L) ± SEM.

The effects of GSK263 on total GIP were variable and not significant, but there was a strong trend for sitagliptin to reduce GIP levels (Figure 3A and 3B and Tables 6 and 7).

Table 8 provides a summary of the peptide changes observed in these studies.

**Table 8:** Summary of the effects of GSK263, sitagliptin and metformin on circulating PYY, GLP-1 and GIP.

	Total PYY	Total GLP-1	Active GLP-1_7–36_	Total GIP
GSK263	** 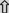 **	** 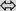 **	** 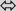 **	** 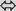 **
Metformin	** 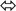 **	**? 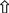 **	** 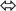 **	** 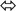 **
GSK263 + Metformin	** 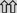 **	** 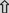 **	** 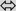 **	** 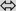 **
Sitagliptin	** 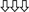 **	** 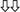 **	** 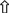 **	** 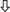 **
GSK263 + Sitagliptin	** 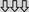 **	** 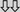 **	** 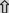 **	** 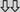 **
Sitagliptin + Metformin	** 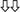 **	** 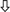 **	** 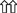 **	** 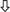 **

### Glucose-related effects

#### Single doses of GSK263 or sitagliptin

**Figure 4:**
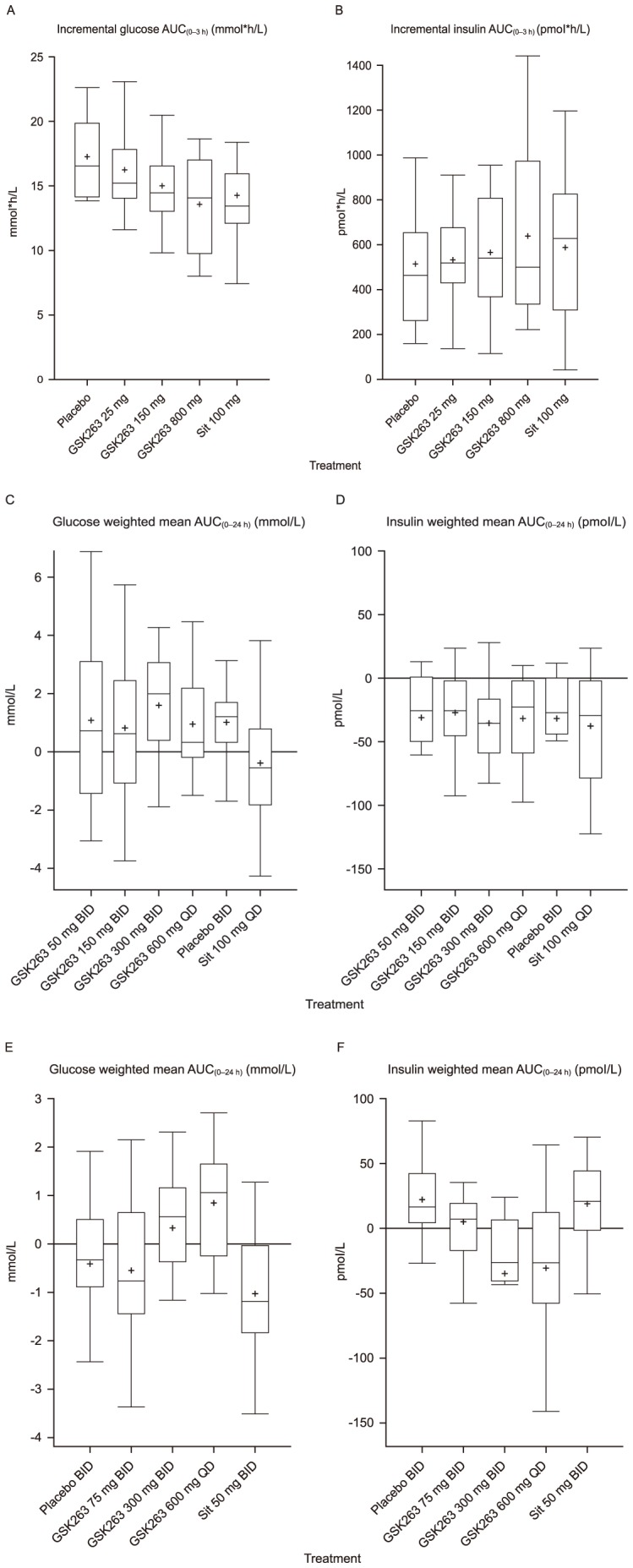
Plasma glucose and insulin. Box plots of incremental plasma glucose AUC_(0–3 h)_ (A; mmol*h/L) and insulin AUC_(0–3 h)_ (B; pmol*h/L) after an OGTT. Single doses of GSK263, placebo and sitagliptin (Sit) were administered in a 5-way cross-over design. Boxplots of WM-AUC_(0–24 h)_ for plasma glucose (C; mmol/L) and insulin (D; pmol/L) in Study 1 following 13 days of dosing GSK263, placebo or sitagliptin (Sit) after stopping prior diabetic medications. Boxplots of WM-AUC_(0–24 h)_ for glucose (E; mmol/L) and insulin (F; pmol/L) following 14 days of co-administration of GSK263, placebo or sitagliptin (Sit) with metformin. Box plots show 25^th^, 50^th^, and 75^th^ percentiles (horizontal bars), 1.5 interquartile ranges (error bars), and mean (+).

There was a trend for a reduction of glucose incremental AUC_(0–3 h)_ during the OGTT with increasing single doses of GSK263 from 25–800 mg (Figure 4A). At 800 mg, the reduction in glucose incremental AUC_(0–3 h)_ was similar to that seen with 100 mg sitagliptin (approximately 20%). The insulin responses to GSK263 were highly variable, and no consistent dose-response was observed (Figure 4B). GSK263 did not alter fasting or OGTT-derived minimal model estimates of insulin sensitivity (data not shown). Pancreatic beta-cell function could not be estimated using the C-peptide minimal model.

#### Repeat doses of GSK263 or sitagliptin

After 13 days or 14 days of dosing, the BID and QD doses of GSK263 did not reduce plasma fasting glucose (data not shown) or glucose WM-AUC_(0–24 h)_ (Figures 4C and 4E) in subjects with T2D, when compared with placebo. In contrast, 100 mg sitagliptin tended to reduce glucose WM-AUC_(0–24)_, but it did not augment the glucose effects of GSK263, compared to placebo, when they were co-administered for 1 day (data not shown).

Overall, there were no significant changes in plasma insulin (Figures 4D and 4F), C-peptide or glucagon WM-AUC_(0–24 h)_, and in feelings of hunger, craving and fullness, caloric intake, or body weight (data not shown).

#### Pharmacokinetics, Safety and tolerability of GSK263

**Figure 5:**
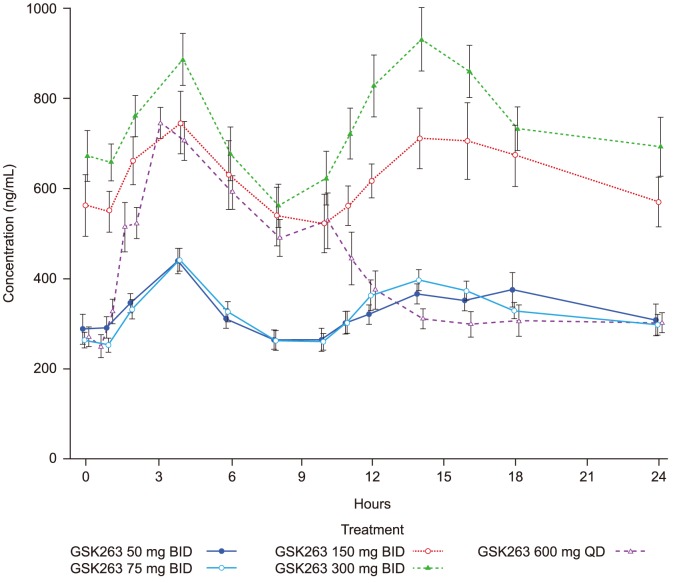
Steady-State plasma concentrations of GSK263 in Studies 1 and 2. Mean (± SEM; ng/mL) concentration-time profiles of GSK263 on Day 13 of dosing in Study 1 (50 mg BID, 150 mg BID, 300 mg BID, and 600 mg QD) and on Day 14 in Study 2 (75 mg BID, 300 mg BID, and 600 mg QD). The concentration data for the 2 studies were combined for groups administered the same doses of GSK263 as there were no meaningful differences in the concentration-time profiles obtained when GSK263 was co-dosed with sitagliptin or metformin.

Mean (± standard error of the mean; SEM) steady-state concentrations of GSK263 across the 2 are presented in Figure 5. A summary of the PK parameters is presented in Table 9.

**Table 9:** Pharmacokinetic parameters of GSK263 following single and repeat-dose administration in Studies 1 and 2†.

Study	Dosing duration	GSK263 Regimen	AUC_(0–24 h)_ (ng.h/mL) Geometric mean (% coefficient of variation)		C_max_ (ng/mL) Geometric mean (% coefficient of variation)	
			Day 1	Steady-State	Day 1	Steady-State
1	Single dose	25 mg QD (Fasted)	524 (21.1)	NA	52.0 (17.1)	NA
		150 mg QD (Fasted)	1685 (31.0)	NA	166 (37.5)	NA
		800 mg QD (Fasted)	3986 (28.4)	NA	380 (28.2)	NA
		800 mg QD (Fasted)	3370 (21.5)	NA	340 (32.6)	NA
		800 mg QD (Fed)	12640 (27.6)	NA	944 (47.3)	NA
	Repeat doses	50 mg BID (Fed)	3552 (15.6)	8026 (22.3)	219 (14.1)	438 (19.5)
		150 mg BID (Fed)	6440 (26.2)	14357 (29.8)	365 (29.2)	716 (30.8)
		300 mg BID (Fed)	11079 (24.4)	19135 (34.5)	584 (30.1)	951 (35.0)
		600 mg QD (Fed)	7153 (34.7)	10167 (26.7)	721 (23.3)	773 (23.0)
2	Single dose	300 mg QD (Fed)	7046 (11.1)	NA	583 (28.1)	NA
	Repeat doses	75 mg BID (Fed)	3773 (30.9)	7785 (19.2)	278 (36.6)	458 (20.4)
		300 mg BID (Fed)	9969 (40.0)	15479 (33.0)	687 (33.2)	902 (30.0)
		600 mg QD (Fed)	6792 (24.6)	9391 (32.9)	611 (29.2)	665 (37.8)

† All calculations of noncompartmental parameters were based on actual sampling times. The maximum plasma concentration (C_max_) was estimated directly from the raw concentration-time data (C_max_). The apparent terminal elimination half-life (t_1/2_) obtained as the ratio of ln2/λz, where λz is the terminal phase rate constant estimated by linear regression analysis of the log transformed concentration-time data. The area under the plasma concentration-time curve to the last quantifiable concentration (AUC_(0–t)_) was determined using the linear trapezoidal rule for increasing concentrations and the logarithmic trapezoidal rule for decreasing concentrations.

**Abbreviations:** NA, Not Applicable.

The terminal elimination half-life of GSK263 ranged from approximately 13 h to 18 h and plasma concentrations reached steady-state within ∼4 days. Food caused a ∼4-fold increase in GSK263 oral bioavailability and delayed time to maximum plasma concentration from ∼2 h to 5 h post-dose. As a result, to maximize plasma concentrations of GSK263, all repeat doses were administered immediately after eating a meal. No pharmacokinetic interactions were observed when GSK263 was co-administered with sitagliptin or metformin.

The adverse events collection process is summarized in Table 2, including the grading method used. GSK263 was generally well-tolerated by the subjects. The reported adverse events in the 2 studies are summarized in Table 10. All adverse events were Grades 1 (mild) or 2 (moderate), except for one Grade 3 (severe) adverse event of myalgia with normal plasma creatine kinase values, as summarized in the footnotes to Table 10. There were no Grade 4 or 5 adverse events.

**Table 10:** Adverse events (AEs) reported in Study 1 and Study 2†.

Summary of all AEs: Study 1 Parts A and B						
	GSK263 25 mg (N = 12)	GSK263 150 mg (N = 12)	GSK263 800 mg (N = 11)	GSK263 800 mg Fed^*^ (N = 4)	Placebo (N = 11)	Sitagliptin 100 mg (N = 13)
Subjects with any AE N (%)	2 (17)	2 (17)	4 (36)	1 (25)	2 (18)	2 (15)
Headache, (%)	1 (8)	1 (8)	2 (18)	1 (25)	2 (18)	0
Dizziness, (%)	2 (17)	1 (8)	0	0	0	0
Vision blurred, (%)	1 (8)	0	1 (9)	0	0	1 (8)
Decreased appetite, (%)	1 (8)	0	0	0	0	0
Hyperglycemia, (%)	0	0	0	0	0	1 (8)
Hypoglycemia, (%)	0	0	1 (9)	0	0	0
Chest pain, (%)	0	1 (8)	0	0	0	0
Fatigue, (%)	1 (8)	0	0	0	0	0
Constipation	0	0	1 (9)	0	0	0
Nausea	1 (8)	0	0	0	0	0
Skin laceration, (%)	0	0	0	0	0	(8)

Summary of AEs occurring in two or more subjects: Study 1 Part C^1,2^						
	GSK263 50 mg BID (N = 14)	GSK263 150 mg BID (N = 16)	GSK263 300 mg BID (N = 12)	GSK263 600 mg QD (N = 14)	Placebo BID (N = 15)	Sitagliptin 100 mg QD (N = 12)
Subjects with Any AE, N (%)	4 (29)	5 (31)	1 (8)	4 (29)	4 (27)	2 (17)
Hyperglycemia, (%)	2 (14)	2 (13)	0	1 (7)	0	0
Constipation, (%)	1 (7)	2 (13)	0	0	0	1 (8)
Vomiting, (%)	1 (7)	0	0	1 (7)	2 (13)	0
Headache, (%)	1 (7)	1 (6)	0	0	2 (13)	0
Diarrhea, (%)	1 (7)	0	0	0	2 (13)	0
Nausea, (%)	1 (7)	0	0	0	2 (13)	0
Dizziness, (%)	1 (7)	0	0	1 (7)	0	0
Upper respiratory tract infection, (%)	1 (7)	0	0	1 (7)	0	0

Summary of AEs occurring in 2 or more subjects: Study 2 Part B					
	GSK263 75 mg BID (N = 13)	GSK263 300 mg BID (N = 12)	GSK263 600 mg QD (N = 15)	Placebo (N = 14)	Sitagliptin 50 mg BID (N = 13)
Subjects with any AE, N (%)	3 (23)	3 (25)	4 (27)	2 (14)	5 (38)
Diarrhea, (%)	1 (8)	1 (8)	3 (20)	0	0
Headache, (%)	0	2 (17)	0	2 (14)	1 (8)
Nausea, (%)	0	2 (17)	1 (7)	0	0
Flatulence, (%)	1 (8)	0	1 (7)	0	0

† There were no AEs reported in the fasted period of Part B of Study 1 or Part A of Study 2. (1) One subject administered 600 mg QD GSK263 developed severe myalgia approximately 9 days after starting dosing that lasted several days after stopping GSK263. The AE was considered by the investigator to be related to the study drug. The myalgia was not accompanied by changes in creatine kinase, aspartate aminotransferase, or viral titers, and the systemic exposure of GSK263 in this subject was comparable to exposures observed in other subjects administered 600 mg QD. No alternative etiological factor for the myalgia was identified in this subject. It is noteworthy that no muscle toxicity had been observed in the routine rodent and dog toxicity studies conducted with GSK263. (2) Seven subjects were withdrawn in Study 1 due to AEs, but none of these were attributed to GSK263 by the investigator.

**Abbreviations:** AE, Adverse Event.

## Discussion

We conducted these diabetes studies because GSK263, a potent and selective GPR119 agonist, altered plasma glucose and gut peptide profiles in rodents and healthy subjects (GlaxoSmithKline, unpublished data). The most remarkable finding in our studies was that repeated doses of GSK263 augmented PYY levels by ∼ 5-fold. The highest levels were achieved with the 300 mg BID and 600 mg QD regimens that were associated with the highest GSK263 concentrations during the daytime period. The 300 mg BID dose also elevated fasting PYY levels, in contrast to the much attenuated fasting responses to the 50 mg BID, 150 mg BID, and 600 mg QD doses that had lower overnight systemic (and presumably gut) concentrations of GSK263. Total PYY levels were augmented by intake of nutrient secretagogues, reaching peak levels of ∼50 pM during the three meals (breakfast≈lunch≈dinner). Furthermore, metformin enhanced the postprandial effects of GSK263 on PYY and altered the meal-related profiles (lunch>breakfast≈dinner). In contrast, co-dosing with sitagliptin abolished the effects of GSK263 on total PYY, GLP-1 and GIP. There was no evidence of tachyphylaxis of the gut peptide effects of GSK263 after 13 or 14 days of dosing, but it should be noted that human enterocytes turn over every few days, and this may have resulted in transient exposure of their GPR119 receptors.

PYY is secreted from enteroendocrine L cells as PYY_1–36_, which is then processed by DPP-IV to the Y2 selective agonist PYY_3–36_ [21]. When GSK263 was dosed alone or with metformin, most of the circulating total PYY that we measured would be expected to be in the form of PYY_3–36_ because of the activity of DPP-IV [5]. In our studies, sitagliptin produced a profound reduction of plasma total PYY levels, expected to be mainly PYY_1–36_ which has little anorexigenic effect [21]. Aaboe *et al.* have reported the effects of 100 mg QD sitagliptin on circulating PYY, GLP-1 and GIP when administered for 12 weeks to subjects with T2D taking metformin [22]. In their study, sitagliptin decreased the AUC for total PYY by 25%, while the AUC for PYY_3–36_ was decreased by more than 60% at the end of the study. PYY_1–36_ was increased by 20% in Week 1, but not at Week 12. These data are consistent with the DPP-IV inhibitor blocking the conversion of PYY_1–36_ to PYY_3–36_, and with our observations based on 2 weeks of dosing with GSK263 or sitagliptin, alone (Study 1) and with metformin (Study 2). The concentrations of total PYY observed with 300 mg BID of GSK263 in our T2D studies (up to 50 pM in Study 1 and 100 pM in Study 2; presumed to be mainly PYY_3–36_) should have reduced food intake based on the reported effects of acute PYY_3–36_ infusion [5], [23], yet we did not observe changes in the self-reported hunger, satiety or craving parameters, or estimations of caloric intake. We acknowledge that the clinical significance of the changes in relative abundance of PYY peptide subtypes will require further evaluation, especially in light of the fact that they have different affinities for NPY receptor subtypes [24].

It is also instructive to compare the peptide profiles observed with GSK263 to those reported after bariatric surgery [25], where rapid improvements in glucose control have been seen post-operatively. The post-prandial rises in PYY after gastric bypass surgery are comparable or somewhat greater than those seen with GSK263 when co-dosed with metformin, but the post-surgical increases in GLP-1 appear to be much greater [26]–[28].

The PYY and GLP-1 profiles shown in Figures 1 and 2 indicate that the PYY responses we observed were different from the changes in circulating total or active GLP-1, even though the precursors of these peptides are commonly co-expressed in the same enteroendocrine L cells [29]. This would suggest that an L cell has the ability to secrete PYY and GLP-1 in a context-specific and independent way or that there may be PYY-predominant or GLP-1-predominant L cells that can respond differently to cell-surface G protein-coupled receptor activation [30].

While GSK263 does not produce weight loss in rodent models, weight loss has been reported with a variety of GPR119 agonists that also reduce gastric emptying, including oleoylethanolamide, MBX-2982, and PSN821 [16]. The data reported by Lan *et al.* [31] indicate that at least some of these phenomena may be off-target effects because they persist in the GPR119 knockout mouse (GlaxoSmithKline unpublished data).

In our study, metformin had little additional effect on active GLP-1_7–36_ levels when dosed alone, but it may increase total GLP-1, suggesting that at commonly used doses it has little inhibitory effect on DPP-IV. The lack of change in active GLP-1_7–36_ cannot be due to our assay procedure, because we clearly detected elevations in the sitagliptin-treated subjects. Some have reported a similar lack of effect of metformin on DPP-IV [32], [33], but others describe variable inhibition of DPP-IV, or increases of active GLP-1_7–36_ [34]–[37] or total GLP-1 [7]. Sitagliptin suppressed total GLP-1 levels while increasing active GLP-1_7–36_, suggesting a negative feedback loop controlling secretion of GLP-1 from enteroendocrine L cells. Others have reported suppression of total GLP-1 and GIP with DPP-IV inhibitors [9], [38], and reduction of endogenous total GLP-1 and PYY levels by GLP-1 receptor agonists [39], [40]. Taken together, our data suggest that metformin alters the feedback characteristics because it augments circulating concentrations of active GLP-1_7–36_ when co-dosed with sitagliptin, without further reducing total GLP-1 levels. Metformin may facilitate the secretion of active GLP-1_7–36_, perhaps through a muscarinic receptor subtype 3/gastrin-releasing peptide pathway [41], which is then protected by the DPP-IV inhibitor. Sitagliptin has been reported to have a direct GLP-1 secretagogue effect on mouse and human intestinal cell lines [42], but these *in vitro* data are difficult to reconcile with the peptide profiles we and others have observed in subjects with type 2 diabetes.

Although we observed a ∼20% reduction in incremental glucose AUC_(0–3 h)_ in drug-naïve subjects with T2D with 800 mg GSK263, this did not translate to a sustained reduction in plasma glucose parameters when subjects were administered 300 mg BID or 600 mg QD for 14 days, even though the steady-state plasma concentrations of GSK263 were greater than those achieved with the single dose of 800 mg. Our data are consistent with the lack of glycemic efficacy reported by Katz and colleagues with a structurally distinct GPR119 agonist, JNJ-38431055 [43]. Minimal modelling post-OGTT had suggested that GSK263 increased insulin sensitivity in healthy subjects with no observable effect on β-cell function, but GPR119 agonists are reported to increase insulin secretion when glucose is infused intravenously [14]. The GPR119 agonists MBX-2982 and PSN821 reduce fasting and post-prandial glucose levels in healthy subjects with fasting hyperglycemia or impaired glucose tolerance [44], [45] and in subjects with T2D [46], but these drugs may have off-target effects on gastric emptying.

Our studies have a number of limitations. These early phase trials of a new chemical entity, GSK263, were of short duration and included relatively small numbers of subjects. The sample sizes were based on feasibility because of the exploratory nature of the PD assessments. Nevertheless, our quantitative assessments of the gut hormones confirm previous reports that GPR119 agonists elevate circulating PYY, but lack glycemic efficacy, while our new insights need to be confirmed in future studies. Our studies were too short to make meaningful assessments of the impact of GSK263 on weight, especially as the subjects were in a clinical unit with limited opportunity to exercise and meals were standardized to provide consistent stimuli for assessment of glycemic parameters. While the fed-fasted paradigm suggested that PYY levels were only elevated in the presence of GSK263 and nutrients, the absence of a placebo arm limits our ability to distinguish the contribution of GSK263 in the presence of nutrient-based augmentation. We have not been able to build a robust PK/PD model for the effects of GSK263 on PYY, suggesting that for this response the concentration of GSK263 in the environment of the GPR119 receptors in the gut epithelium may be more important than systemic concentrations of the drug.

In conclusion, our data indicate that GPR119 agonism with GSK263 causes substantial changes in circulating total PYY levels, but it did not improve glycemic control in subjects with type 2 diabetes when dosed alone or with metformin or sitagliptin. The range and magnitude of the effects on the anorexigenic peptides PYY and GLP-1, and the augmentation by metformin, have not been reported previously.

## Supporting Information

Checklist S1 **CONSORT Checklist.**
(DOC)

Protocol S1 **Trial Protocol 1.**
(PDF)

Protocol S2 **Trial Protocol 2.**
(PDF)

## References

[1] Nauck MA (2009) Unraveling the science of incretin biology. Am J Med 122: S3–S10. 10.1016/j.amjmed.2009.03.012 19464426

[2] Drucker DJ (2006) The biology of incretin hormones. Cell Metab 3: 153–165. 16517403 10.1016/j.cmet.2006.01.004

[3] Tharakan G, Tan T, Bloom S (2011) Emerging therapies in the treatment of ‘diabesity’: beyond GLP-1. Trends Pharmacol Sci 32: 8–15. 21130506 10.1016/j.tips.2010.10.003

[4] Deacon CF (2004) Circulation and degradation of GIP and GLP-1. Horm Metab Res 36: 761–765. 15655705 10.1055/s-2004-826160

[5] le Roux CW, Batterham RL, Aylwin SJ, Patterson M, Borg CM, et al (2006) Attenuated peptide YY release in obese subjects is associated with reduced satiety. Endocrinology 147: 3–8. 16166213 10.1210/en.2005-0972

[6] Deacon CF (2007) Dipeptidyl peptidase 4 inhibition with sitagliptin: a new therapy for type 2 diabetes. Expert Opin Investig Drugs 16: 533–545. 10.1517/13543784.16.4.533 17371200

[7] Mannucci E, Tesi F, Bardini G, Ognibene A, Petracca MG, et al (2004) Effects of metformin on glucagon-like peptide-1 levels in obese patients with and without Type 2 diabetes. Diabetes Nutr Metab 17: 336–342. 15887627

[8] Yasuda N, Inoue T, Nagakura T, Yamazaki K, Kira K, et al (2002) Enhanced secretion of glucagon-like peptide 1 by biguanide compounds. Biochem Biophys Res Commun 298: 779–784. 12419322 10.1016/s0006-291x(02)02565-2

[9] Herman GA, Bergman A, Stevens C, Kotey P, Yi B, et al (2006) Effect of single oral doses of sitagliptin, a dipeptidyl peptidase-4 inhibitor, on incretin and plasma glucose levels after an oral glucose tolerance test in patients with type 2 diabetes. J Clin Endocrinol Metab 91: 4612–4619. 16912128 10.1210/jc.2006-1009

[10] Correia S, Carvalho C, Santos MS, Seica R, Oliveira CR, et al (2008) Mechanisms of action of metformin in type 2 diabetes and associated complications: an overview. Mini Rev Med Chem 8: 1343–1354. 18991752 10.2174/138955708786369546

[11] Natali A, Ferrannini E (2006) Effects of metformin and thiazolidinediones on suppression of hepatic glucose production and stimulation of glucose uptake in type 2 diabetes: a systematic review. Diabetologia 49: 434–441. 16477438 10.1007/s00125-006-0141-7

[12] Odori S, Hosoda K, Tomita T, Fujikura J, Kusakabe T, et al (2013) GPR119 expression in normal human tissues and islet cell tumors: evidence for its islet-gastrointestinal distribution, expression in pancreatic beta and alpha cells, and involvement in islet function. Metabolism 62: 70–78. 22883930 10.1016/j.metabol.2012.06.010

[13] Ohishi T, Yoshida S (2012) The therapeutic potential of GPR119 agonists for type 2 diabetes. Expert Opin Investig Drugs 21: 321–328. 10.1517/13543784.2012.657797 22292451

[14] Katz LB, Gambale JJ, Rothenberg PL, Vanapalli SR, Vaccaro N, et al (2011) Pharmacokinetics, pharmacodynamics, safety, and tolerability of JNJ-38431055, a novel GPR119 receptor agonist and potential antidiabetes agent, in healthy male subjects. Clin Pharmacol Ther 90: 685–692. 21975348 10.1038/clpt.2011.169

[15] Fang J, Tang J, Carpenter AJ, Peckham G, Conlee CR, et al. (2008) Preparation of piperidine derivatives as GPR119 agonists for treating metabolic disorders. United States patent PCT/US2007/086434

[16] Shah U, Kowalski TJ (2010) GPR119 agonists for the potential treatment of type 2 diabetes and related metabolic disorders. Vitam Horm 84: 415–448. 21094910 10.1016/B978-0-12-381517-0.00016-3

[17] International Conference on Harmonisation (1996) ICH Harmonised Tripartite Guideline. Guideline for Good Clinical Practice. Version 10.

[18] World Medical Association (2000) Declaration of Helsinki: ethical principles for medical research involving human subjects. JAMA 284: 3043–3045. 11122593

[19] Polli JW, Hussey E, Bush M, Generaux G, Smith G, et al (2013) Evaluation of drug interactions of GSK1292263 (a GPR119 agonist) with statins: from in vitro data to clinical study design. Xenobiotica 43: 498–508. 23256625 10.3109/00498254.2012.739719

[20] Dalla MC, Campioni M, Polonsky KS, Basu R, Rizza RA, et al (2005) Two-hour seven-sample oral glucose tolerance test and meal protocol: minimal model assessment of beta-cell responsivity and insulin sensitivity in nondiabetic individuals. Diabetes 54: 3265–3273. 16249454 10.2337/diabetes.54.11.3265

[21] Karra E, Chandarana K, Batterham RL (2009) The role of peptide YY in appetite regulation and obesity. J Physiol 587: 19–25. 19064614 10.1113/jphysiol.2008.164269 PMC2670018

[22] Aaboe K, Knop FK, Vilsboll T, Deacon CF, Holst JJ, et al (2010) Twelve weeks treatment with the DPP-4 inhibitor, sitagliptin, prevents degradation of peptide YY and improves glucose and non-glucose induced insulin secretion in patients with type 2 diabetes mellitus. Diabetes Obes Metab 12: 323–333. 20380653 10.1111/j.1463-1326.2009.01167.x

[23] Batterham RL, Cohen MA, Ellis SM, le Roux CW, Withers DJ, et al (2003) Inhibition of food intake in obese subjects by peptide YY3–36. N Engl J Med 349: 941–948. 12954742 10.1056/NEJMoa030204

[24] Babilon S, Morl K, Beck-Sickinger AG (2013) Towards improved receptor targeting: anterograde transport, internalization and postendocytic trafficking of neuropeptide Y receptors. Biol Chem 394: 921–936. 23449522 10.1515/hsz-2013-0123

[25] Beckman LM, Beckman TR, Earthman CP (2010) Changes in gastrointestinal hormones and leptin after Roux-en-Y gastric bypass procedure: a review. J Am Diet Assoc 110: 571–584. 20338283 10.1016/j.jada.2009.12.023 PMC4284064

[26] le Roux CW, Aylwin SJ, Batterham RL, Borg CM, Coyle F, et al (2006) Gut hormone profiles following bariatric surgery favor an anorectic state, facilitate weight loss, and improve metabolic parameters. Ann Surg 243: 108–114. 16371744 10.1097/01.sla.0000183349.16877.84 PMC1449984

[27] le Roux CW, Welbourn R, Werling M, Osborne A, Kokkinos A, et al (2007) Gut hormones as mediators of appetite and weight loss after Roux-en-Y gastric bypass. Ann Surg 246: 780–785. 17968169 10.1097/SLA.0b013e3180caa3e3

[28] Chronaiou A, Tsoli M, Kehagias I, Leotsinidis M, Kalfarentzos F, et al (2012) Lower Ghrelin Levels and Exaggerated Postprandial Peptide-YY, Glucagon-Like Peptide-1, and Insulin Responses, After Gastric Fundus Resection, in Patients Undergoing Roux-en-Y Gastric Bypass: A Randomized Clinical Trial. Obes Surg 22: 1761–70. 22911148 10.1007/s11695-012-0738-5

[29] Engelstoft MS, Egerod KL, Lund ML, Schwartz TW (2013) Enteroendocrine cell types revisited. Curr Opin Pharmacol 13: 912–921. 24140256 10.1016/j.coph.2013.09.018

[30] Engelstoft MS, Egerod KL, Holst B, Schwartz TW (2008) A gut feeling for obesity: 7TM sensors on enteroendocrine cells. Cell Metab 8: 447–449. 19041758 10.1016/j.cmet.2008.11.004

[31] Lan H, Vassileva G, Corona A, Liu L, Baker H, et al (2009) GPR119 is required for physiological regulation of glucagon-like peptide-1 secretion but not for metabolic homeostasis. J Endocrinol 201: 219–230. 19282326 10.1677/JOE-08-0453

[32] Migoya EM, Bergeron R, Miller JL, Snyder RN, Tanen M, et al (2010) Dipeptidyl peptidase-4 inhibitors administered in combination with metformin result in an additive increase in the plasma concentration of active GLP-1. Clin Pharmacol Ther 88: 801–808. 21048706 10.1038/clpt.2010.184

[33] Hinke SA, Kuhn-Wache K, Hoffmann T, Pederson RA, McIntosh CH, et al (2002) Metformin effects on dipeptidylpeptidase IV degradation of glucagon-like peptide-1. Biochem Biophys Res Commun 291: 1302–1308. 11883961 10.1006/bbrc.2002.6607

[34] Cuthbertson J, Patterson S, O'Harte FP, Bell PM (2009) Investigation of the effect of oral metformin on dipeptidylpeptidase-4 (DPP-4) activity in Type 2 diabetes. Diabet Med 26: 649–654. 19538242 10.1111/j.1464-5491.2009.02748.x

[35] Green BD, Irwin N, Duffy NA, Gault VA, O'Harte FP, et al (2006) Inhibition of dipeptidyl peptidase-IV activity by metformin enhances the antidiabetic effects of glucagon-like peptide-1. Eur J Pharmacol 547: 192–199. 16945366 10.1016/j.ejphar.2006.07.043

[36] Lindsay JR, Duffy NA, McKillop AM, Ardill J, O'Harte FP, et al (2005) Inhibition of dipeptidyl peptidase IV activity by oral metformin in Type 2 diabetes. Diabet Med 22: 654–657. 15842525 10.1111/j.1464-5491.2005.01461.x

[37] Thondam SK, Cross A, Cuthbertson DJ, Wilding JP, Daousi C (2012) Effects of chronic treatment with metformin on dipeptidyl peptidase-4 activity, glucagon-like peptide 1 and ghrelin in obese patients with Type 2 diabetes mellitus. Diabet Med 29: e205–e210. 22486277 10.1111/j.1464-5491.2012.03675.x

[38] El-Ouaghlidi A, Rehring E, Holst JJ, Schweizer A, Foley J, et al (2007) The dipeptidyl peptidase 4 inhibitor vildagliptin does not accentuate glibenclamide-induced hypoglycemia but reduces glucose-induced glucagon-like peptide 1 and gastric inhibitory polypeptide secretion. J Clin Endocrinol Metab 92: 4165–4171. 17698900 10.1210/jc.2006-1932

[39] Sze L, Purtell L, Jenkins A, Loughnan G, Smith E, et al (2011) Effects of a single dose of exenatide on appetite, gut hormones, and glucose homeostasis in adults with Prader-Willi syndrome. J Clin Endocrinol Metab 96: E1314–E1319. 21632815 10.1210/jc.2011-0038

[40] Naslund E, Bogefors J, Skogar S, Gryback P, Jacobsson H, et al (1999) GLP-1 slows solid gastric emptying and inhibits insulin, glucagon, and PYY release in humans. Am J Physiol 277: R910–R916. 10484511 10.1152/ajpregu.1999.277.3.R910

[41] Mulherin AJ, Oh AH, Kim H, Grieco A, Lauffer LM, et al (2011) Mechanisms underlying metformin-induced secretion of glucagon-like peptide-1 from the intestinal L cell. Endocrinology 152: 4610–4619. 21971158 10.1210/en.2011-1485

[42] Sangle GV, Lauffer LM, Grieco A, Trivedi S, Iakoubov R, et al (2012) Novel Biological Action of the Dipeptidylpeptidase-IV Inhibitor, Sitagliptin, as a Glucagon-Like Peptide-1 Secretagogue. Endocrinology 153: 564–573. 22186413 10.1210/en.2011-1732

[43] Katz LB, Gambale JJ, Rothenberg PL, Vanapalli SR, Vaccaro N, et al (2012) Effects of JNJ-38431055, a novel GPR119 receptor agonist, in randomized, double-blind, placebo-controlled studies in subjects with type 2 diabetes. Diabetes Obes Metab 14: 709–716. 22340428 10.1111/j.1463-1326.2012.01587.x

[44] Roberts B, Gregoire FM, Karpf DB, Clemens E, Lavan B, et al (2009) MBX-2982, a novel oral GPR119 agonist for the treatment of Type 2 Diabetes: Results of single and multiple dose studies. Diabetes 58: A43.

[45] Roberts B, Karpf DB, Martin R, Lavan B, Wilson M, et al (2010) MBX-2982, a novel GPR119 agonist, shows greater efficacy in patients with the most glucose intolerance: Results of a Phase 1 study with an improved formulation. Diabetes 59: 603–P.

[46] Goodman ML, Dow J, van Vliet AA, Hadi S, Karbiche D, et al (2011) The novel GPR119-recepor agonist PSN821 shows glucose lowering and decreased energy intake in patients with T2DM after 14 days treatment. Diabetes 60: A84.

