# Supplementary material for: Gut Hormone Pharmacology of a Novel GPR119 Agonist (GSK1292263), Metformin, and Sitagliptin in Type 2 Diabetes Mellitus: Results from Two Randomized Studies
Source: PLoS One. 2014 Apr 3;9(4):e92494. doi: 10.1371/journal.pone.0092494 (PMC3974707; doi:10.1371/journal.pone.0092494)
Supplement: Protocol S1 — Trial Protocol 1. (PDF) [file pone.0092494.s002.pdf]

Division: Worldwide Development
Retention Category: GRS019
Information Type: Protocol Amendment

**Title:**	A study in type 2 diabetic subjects of single and multiple doses of orally administered GSK1292263 to investigate the safety, tolerability, pharmacokinetics and pharmacodynamics of the compound

Compound Number: GSK1292263

Effective Date: 23-DEC-2009

Protocol Amendment Number: 04

Description: This amendment includes (i) preliminary safety and tolerability information from Parts A and B of the current study, (ii) preliminary PK information from Parts A, B and C of the current study, (iii) preliminary data from the 13 week toxicology studies in the rat and dog, and (iv) sets the maximum daily dose to be evaluated in Part C at 600mg.

Subject: safety, tolerability, pharmacokinetics, glucose, pharmacodynamics, male, female, type 2 diabetes

Author:

[redacted] CPSSO, Metabolic Pathways
EE Discovery Medicine, Metabolic Pathways CEDD
CPMS, Clinical Pharmacology Modeling and Simulation
Discovery Biometrics - Metabolic

Copyright 2009 the GlaxoSmithKline group of companies. All rights reserved. Unauthorized copying or use of this information is prohibited.

Revision Chronology:

RH2008/00140/00	2009-MAY-12	Original
RH2008/00140/01	2009-JUN-19	Amendment No.: 01 This amendment adds criteria recommended by the IRB and alters Selection Criteria based on comments from the Investigator, in response to recruitment problems.
RH2008/00140/02	2009-AUG-06	Amendment No.: 02 The changes in this amendment are (i) adds co-dosing of GSK1292263 with sitagliptin on Day 14 of Part C. (ii) Cohort 4 will no longer be optional and will evaluate QD or BID doses of GSK1292263, depending on the dosing regimen chosen for Cohort 3, (iii) the maximum daily dose of GSK1292263 that may be administered in this study is set at 800mg total daily dose, (iv) the prior anti-diabetic therapy inclusion criteria for Part C are modified, (v) modifications to sampling times, (vi) adds HCFQ on Day 13.
RH2008/00140/03	2009-SEP-22	Amendment No.: 03 This amendment allows for venous blood or blood plasma glucose for glucose monitoring and selection criteria, clarifies that Part C subjects can only be on monotherapy, clarifies timing of HCFQ, clarifies that glycerol is a PD marker, updates language regarding the central randomization system., updates the T&E table for Part C, clarifies inclusion of non-childbearing potential women, expands the BMI range, clarifies creatinine clearance, adds details on GI surgery inclusion criterion, changes pre-dose ECGs in Part 3 from single to triplicate on specified days, clarifies that telemetry was only in Parts A and B, and adds some details around meals and dosing in Part C.

RH2008/00140/04	2009-DEC-23	Amendment No.: 04 This amendment includes (i) preliminary safety and tolerability information from Parts A and B of the current study, (ii) preliminary PK information from Parts A, B and C of the current study, (iii) preliminary data from the 13 week toxicology studies in the rat and dog, and (iv) sets the maximum daily dose to be evaluated in Part C at 600mg.

CONFIDENTIAL RH2008/00140/04
GPR111598

SPONS[redacted]

[redacted] 23 December 2009

Date

[redacted]
Director and Head
EnteroEndocrine Discovery Medicine
Metabolic Pathways CEDD

SPONSOR/MEDICAL MONITOR INFORMATION PAGE

Medical Monitor and Sponsor Contact Information:

Role	Name	Day Time Phone Number & email	After-hours Phone/Cell/ Pager Number	Fax Number	GSK Address
Primary Medical Monitor	[redacted]	[redacted]	[redacted]	[redacted]	[redacted]
Secondary Medical Monitor	[redacted]	[redacted]	[redacted]	[redacted]	[redacted]
Tertiary Medical Monitor	[redacted]	[redacted]	[redacted]	[redacted]	[redacted]

Sponsor Registered Address:

GlaxoSmithKline Research & Development Limited
980 Great West Road
Brentford
Middlesex, TW8 9GS
UK

In some countries, the clinical trial sponsor may be the local GlaxoSmithKline affiliate company (or designee). If applicable, the details of the alternative Sponsor and contact person in the territory will be provided to the relevant regulatory authority as part of the clinical trial application.

Regulatory Agency Identifying Number(s): 103,221

INVESTIGATOR PROTOCOL AGREEMENT PAGE

I confirm agreement to conduct the study in compliance with the protocol, as amended by this protocol amendment.

Investigator Name:	
Investigator Address:	
Investigator Phone Number:	
Investigator Signature	Date

TABLE OF CONTENTS

	Page
ABBREVIATIONS	11
1. INTRODUCTION	15
1.1. Background	15
1.2. Human Experience with GSK1292263	16
1.2.1. GPR111596 – First Time in Human (FTIH) Study	16
1.2.2. GPR111598 - First in T2DM Subjects: Preliminary Safety and PK Data from Parts A and B	19
1.3. Sitagliptin	22
1.4. Rationale	23
1.4.1. Study Rationale	23
1.4.2. Dose Rationale	26
1.4.3. Stopping Criteria	34
1.5. Summary of Risk Management	35
1.5.1. Risks Related to Washout of Anti-Diabetic Medications	35
1.5.2. Risks Related to GSK1292263	35
1.5.3. Capillary Blood Glucose (CBG) Monitoring	40
2. OBJECTIVE(S)	41
2.1. Primary	41
2.2. Secondary	41
2.3. Exploratory	42
3. ENDPOINT(S)	42
3.1. Primary	42
3.2. Secondary	42
3.3. Exploratory Analyses	43
4. INVESTIGATIONAL PLAN	43
4.1. Discussion of Design	43
4.1.1. Part A: Crossover Study Comparing a Low, Mid and High Dose of GSK1292263, Placebo and Sitagliptin (Cohort 1)	43
4.1.2. Part B: GSK1292263 with Food (Cohort 2)	44
4.1.3. Part C: Repeat Dosing (Cohorts 3 and 4)	45
4.1.4. Screening	48
4.1.5. Washout (Parts B and C Only)	48
4.1.6. Treatment regimen	49
4.1.7. Follow-up Visit	49

4.1.8. Pharmacodynamic Testing 50
4.2. Treatment Assignment 52
4.3. Investigational Product Dosage/Administration 53
4.4. Dose Adjustment/Stopping Criteria 54
4.4.1. Liver Chemistry Stopping Criteria 55
4.4.2. QTc Withdrawal Criteria 56
4.4.3. Blood Glucose Withdrawal Criteria 56
4.4.4. Additional Withdrawal Criteria 56
4.5. Time and Events Table (Parts A and B) 57
4.6. Time and Events Table (Part C) 59
5. STUDY POPULATION 62
5.1. Number of Subjects 62
5.2. Eligibility Criteria 62
5.2.1. Inclusion Criteria 62
5.2.2. Exclusion Criteria 64
5.2.3. Other Eligibility Criteria Considerations 67
6. DATA ANALYSIS AND STATISTICAL CONSIDERATIONS 68
6.1. Hypotheses and Treatment Comparisons 68
6.2. Sample Size Considerations 69
6.2.1. Sample Size Assumptions 69
6.2.2. Sample Size Sensitivity 70
6.2.3. Sample Size Re-estimation 70
6.3. Data Analysis Considerations 70
6.3.1. Interim Analysis 70
6.3.2. Final Analyses 71
7. STUDY ASSESSMENTS AND PROCEDURES 73
7.1. Demographic/Medical History Assessments 74
7.2. Safety 74
7.2.1. Physical Exams 74
7.2.2. Vital Signs 74
7.2.3. Electrocardiogram (ECG) 75
7.2.4. Clinical Laboratory Assessments 75
7.3. Pregnancy 76
7.3.1. Time period for collecting pregnancy information 76
7.3.2. Action to be taken if pregnancy occurs 76
7.4. Pharmacokinetics 76
7.4.1. Blood Sample Collection 76

7.4.2. Sample Analysis . . . 77
7.5. Biomarker(s)/Pharmacodynamic Markers . . . 77
7.5.1. Type II Diabetes Biomarkers/Pharmacodynamic Markers . . . 77
7.5.2. Exploratory Biomarkers . . . 77
7.6. Pharmacogenetics . . . 77
8. LIFESTYLE AND/OR DIETARY RESTRICTIONS . . . 78
8.1. Contraception requirements for Male Subjects . . . 79
9. CONCOMITANT MEDICATIONS AND NON-DRUG THERAPIES . . . 79
9.1. Permitted Medications . . . 79
9.1.1. Anti-Viral Therapies for Influenza . . . 80
9.2. Medications Permitted at Screening but Requiring Washout before Enrollment . . . 80
9.3. Prohibited Medications . . . 80
9.4. Non-Drug Therapies . . . 81
10. COMPLETION OR EARLY WITHDRAWAL OF SUBJECTS . . . 81
10.1. Subject Completion . . . 81
10.2. Subject Withdrawal Criteria . . . 81
10.3. Subject Withdrawal Procedures . . . 81
10.3.1. Subject Withdrawal from Study . . . 81
10.3.2. Subject Withdrawal from Investigational Product . . . 82
10.4. Treatment After the End of the Study . . . 82
10.5. Screen and Baseline Failures . . . 82
11. INVESTIGATIONAL PRODUCT(S) . . . 82
11.1. Blinding . . . 82
11.2. Packaging and Labeling . . . 83
11.3. Preparation/Handling/Storage/Accountability . . . 83
11.4. Assessment of Compliance . . . 84
11.5. Treatment of Investigational Product Overdose . . . 84
12. ADVERSE EVENTS (AE) AND SERIOUS ADVERSE EVENTS (SAE) . . . 84
12.1. Definition of Adverse Events . . . 85
12.2. Definition of Serious Adverse Events . . . 86
12.3. Method of Detecting AEs and SAEs . . . 87
12.4. Recording of AEs and SAEs . . . 87
12.5. Evaluating AEs and SAEs . . . 87
12.5.1. Assessment of Intensity . . . 87
12.5.2. Assessment of Causality . . . 88

12.6. Follow-up of AEs and SAEs 88
12.7. Prompt Reporting of SAEs to GSK 89
12.8. Regulatory Reporting Requirements for SAEs 89
13. LIVER CHEMISTRY TESTING PROCEDURES 90
14. STUDY CONDUCT CONSIDERATIONS 92
14.1. Regulatory and Ethical Considerations, Including the Informed Consent Process 92
14.2. Quality Control (Study Monitoring) 92
14.3. Quality Assurance 93
14.4. Study and Site Closure 93
14.5. Records Retention 93
14.6. Provision of Study Results and Information to Investigators 94
14.7. Data Management 94
15. REFERENCES 95
Appendices 96
Appendix 1: Liver Safety Algorithms 96
Appendix 2: Pharmacogenetic research 97
Appendix 3: Hunger, Craving, and Fullness Questionnaire 101
Appendix 4: Protocol Amendment Changes 104

ABBREVIATIONS

µM	Micromoles
ADME	Absorption, Distribution, Metabolism and Excretion
AE	Adverse Event
ALT	Alanine aminotransferase (SGPT)
ANCOVA	Analysis of Co-variance
ANOVA	Analysis of Variance
AST	Aspartate aminotransferase (SGOT)
AUC	Area under concentration-time curve
AUC(0-∞)	Area under the concentration-time curve from time zero (pre-dose) extrapolated to infinite time
AUC(0-τ)	Area under the concentration-time curve over the dosing interval
AUC(0-t)	Area under the concentration-time curve from time zero (pre-dose) to last time of quantifiable concentration within a subject across all treatments
AUC(0-x)	Area under the concentration-time curve from zero (pre-dose) to some fixed nominal time x
BID	Twice daily
BMI	Body mass index
BP	Blood pressure
BPM	Beat Per Minute
BUN	Blood urea nitrogen
Cτ	Trough concentration
CBG	Capillary blood glucose
CCK	Cholecystokinin
CI	Confidence Interval
CL/F	Apparent clearance following oral dosing
Cmax	Maximum observed concentration
CO_2	Carbon dioxide
CPDS	Clinical Pharmacology Data Sciences
CPK	Creatine phosphokinase
CPKMS	Clinical Pharmacokinetics Modelling & Simulation
CRF	Case Report Form
CPSSO	Clinical Pharmacology Science and Study Operations
CV	Coefficient of variance
Cτ	Pre-dose (trough) concentration at the end of the dosing interval
D	Day
DILI	Drug Induced Liver Injury
dL	Deciliters
DMPK	Drug Metabolism and Pharmacokinetics
DNA	Deoxyribonucleic acid
DPP-IV	Dipeptidyl peptidase-IV
ECG	Electrocardiogram
eCRF	Electronic Case Report Form
FDA	Food and Drug Administration
FFA	Free fatty acids
FPG	Fasting plasma glucose

FSH	Follicle Stimulating Hormone
FTIH	First time in humans
FU	Follow-up
g	Grams
GCP	Good Clinical Practice
GCSP	Global Clinical Safety and Pharmacovigilence
GGT	Gamma glutamyltransferase
GI	Gastrointestinal
GIP	Glucose-dependent insulinotropic peptide
GLDH	Glutamate dehydrogenase
GLP	Good Laboratory Practice
GLP-1	Glucagon-like peptide-1
GSK	GlaxoSmithKline
h/hr	Hour(s)
HbA1c	Glycosylated hemoglobin
HBsAg	Hepatitis B surface antigen
HCFQ	Hunger, Craving and Fullness Questionaire
HDL	High-density lipoprotein
Hep B	Hepatitis B
Hep C	Hepatitis C
HIV	Human Immunodeficiency Virus
HR	Heart rate
HWE	Hardy-Weinberg Equilibrium
IB	Investigator’s Brochure
IEC	Independent Ethics Committee
IgM	Immunoglobulin
IGT	Impaired glucose tolerance
IND	Investigational New Drug
IRB	Institutional Review Board
IVRS	Interactive Voice Response System
Kg	Kilogram
L	Liter
LDH	Lactate dehydrogenase
LDL	Low-density lipoprotein
MCH	Mean corpuscular hemoglobin
MCHC	Mean corpuscular hemoglobin concentration
MCV	Mean corpuscular volume
MDRD	Modification of diet in renal disease
mg	Milligrams
min	Minutes
mL	Milliliter
mmHg	Millimeters of Mercury
ms	Milliseconds
MSDS	Material Safety Data Sheet
msec	Milliseconds
MTT	Meal Tolerance Test
ng.h/mL	Nanogram hours per milliliter
ng/mL	Nanograms per milliliter

NOAEL	No observed adverse event level
NSAID	Non-steroidal anti-inflammatory drug
OGTT	Oral glucose tolerance test
PD	Pharmacodynamic
PGx	Pharmacogenetics
PI	Primary Investigator
PIB	Powder in bottle
PK	Pharmacokinetic
PP	Pancreatic polypeptide
PRN	As needed
PYY	Peptide Tyrosine-Tyrosine
QD	Once daily
QTc	Corrected QT interval
QTcB	QT duration corrected for heart rate by Bazett's formula
QTcF	QT duration corrected for heart rate by Fridericia's formula
RAP	Reporting and Analysis Plan
RBC	Red blood cells
RNA	Ribonucleic acid
Ro	Accumulation ratio
Rs	Time invariance ration
SAE	Serious adverse event(s)
SAS	Statistical Analysis Software
SCr	Serum creatinine
SD	Standard deviation
SGOT	Serum glutamic oxaloacetic transaminase
SGPT	Serum gluatmic pyruvic transaminase
SNP	Single nucleotide polymorphism
SPM	Study Procedures Manual
SSRI	Selective serotonin receptor inhibitor
t½	Terminal phase half-life
T2DM	Type 2 diabetes mellitus
tlag	Lag time before observation of drug concentrations in sampled matrix
tmax	Time of occurrence of Cmax
TSH	Thyroid stimulating hormone
ULN	Upper limit of normal
USA	United States of America
V/F	Volume of distribution
WBC	White blood cells
WGS	Whole genome screen
WPW	Wolf-Parkinson-White Syndrome
X-over	Crossover

Trademark Information

Trademarks of the GlaxoSmithKline group of companies
NONE

Trademarks not owned by the GlaxoSmithKline group of companies
Byetta
Chiron RIBA
InForm
Januvia
SAS
WinNonlin

1. INTRODUCTION

GSK1292263 is a potent and selective agonist of GPR119, a receptor that represents a novel approach to target the enteroinsular axis and other target tissues to improve glucose homeostasis. This investigational agent is being developed as a potential treatment for Type 2 Diabetes Mellitus (T2DM), either as a monotherapy or in combination with other marketed oral anti-diabetic agents.

1.1. Background

T2DM is a metabolic disorder primarily characterized by insulin resistance and relative insulin deficiency. Insulin resistance alone is insufficient to produce T2DM, but when accompanied by progressive dysfunction of pancreatic islet ß-cells, the result is prandial and fasting hyperglycemia. By altering nutrient-mediated insulin and glucagon secretion, the incretin hormones, glucagon like peptide-1 (GLP-1) and glucose-dependent insulinotropic peptide (GIP), are both major determinants of glucose disposal following a meal.

In T2DM, plasma levels of GIP appear to be normal or possibly increased, while the ß-cell response to GIP is diminished. It has been shown that ß-cell responses to GIP increase with improved glycemic control. Conversely, although ß-cells remain responsive to the insulinotropic action of GLP-1, the meal-stimulated GLP-1 plasma increases are diminished in patients with impaired glucose tolerance and also in subjects with T2DM. Therefore, therapeutic strategies have focused on increasing GLP-1, either by administration of exogenous GLP-1 analogues, such as exenatide (Byetta) or by preserving endogenous GLP-1 with inhibitors of dipeptidyl peptidase-IV (DPP-IV), the protease responsible for the rapid degradation of incretins following their release from intestinal L-cells.

In preclinical studies, GSK1292263 was noted to have 3 principal mechanisms of action that produce lowering of blood glucose: (i) nutrient-independent and nutrient-augmented release of incretin and gastrointestinal (GI) hormones, (ii) increased glucose-stimulated insulin release, and (iii) increased apparent insulin sensitivity. In addition, a putative natural ligand for GPR119, oleoylethanolamide, is an endocannabinoid that reduces food intake in animal models, and this effect may be augmented by elevations of incretins and PYY.

The durability of GPR119 agonism has been demonstrated in a rodent model in which the glucose lowering effect of GSK1292263 during an OGTT was maintained for 10 weeks.

1.2. Human Experience with GSK1292263

1.2.1. GPR111596 – First Time in Human (FTIH) Study

1.2.1.1. Design

The FTIH study for GSK1292263 in normal volunteers was a single-blinded, randomized study conducted in 5 parts:

Part A (Cohorts 1 and 2): Single dose escalation with 4 periods in each cohort.

- Cohort 1: Single doses of GSK1292263 as 10, 40, 80, 200mg powder-in-bottle (PIB) were administered in a fasting state, with a 75g oral glucose tolerance test (OGTT) given 2h or 4h after dosing. Six subjects received GSK1292263 and 2 subjects received placebo in each period.
- Cohort 2: Single doses of GSK1292263 as 400mg and 50mg PIB were administered in a fasting state with a 75g OGTT given 2h after dosing (Periods 1 and 2). In Periods 3 and 4, the doses of 400mg and 50mg PIB were followed by a breakfast meal taken 30min after dosing. Nine subjects received GSK1292263 and 3 subjects received placebo in each period. Note: Dose escalation was halted at 400mg based on the Investigator's concern regarding hypoglycemic events (see Section 1.2.1.2). At the time, the investigator was blinded to the study drug designation.

Part B (Cohort 4): Three-way crossover to evaluate a single dose of 100mg in a tablet formulation administered in a fasted state or 30min after a breakfast meal, with 100mg PIB administered in a fasting state as a comparator. Twelve subjects were enrolled in this part.

Part C (Cohort 5): Three-way crossover to evaluate the effect of adding-on sitagliptin 100mg to a single 250mg PIB dose of GSK1292263. The doses were administered in a fasting state with a 75g OGTT given 2h after dosing. Twelve subjects were enrolled in this part.

Part D (Cohort 6): A single dose of 250mg of GSK1292263 was administered as PIB on each of 2 consecutive days, followed by a breakfast meal 30min after dosing. Nine subjects received GSK1292263 and 3 subjects received placebo.

Part E (Cohort 8): A single dose of 250mg of GSK1292263 was administered as PIB on each of 5 consecutive days, followed by a breakfast meal taken 30min after dosing. Gastric emptying was assessed using acetaminophen absorption at baseline (Day -1), and on Day 2 and Day 5. Nine subjects received GSK1292263 and 3 subjects received placebo.

Cohorts 3 and 7 were not conducted.

The final data from Parts A-C and preliminary data from Parts D and E of this study are summarized in a Supplement to the Investigator Brochure [GlaxoSmithKline Document Number RM2009/00168/00].

1.2.1.2. Safety Overview

- GSK1292263 was generally well-tolerated over the dose range of 10mg to 400mg. With regards to safety, no significant safety issues were identified that would preclude further clinical investigation.
- The majority of AEs were mild in intensity; no events were severe in intensity. No deaths were reported. The most common AEs attributed to study drug (excluding sitagliptin) were: hypoglycaemia (see below) (n=14), headache (5), dizziness (5), hyperhydrosis (2), cold sweat (1), dry mouth (1) and flushing (1). Two subjects were withdrawn because of (1) an SAE labelled as "viral gastroenteritis" with associated AEs of syncope, hypotension, orthostatic hypotension, hyperhydrosis, cold sweat, diarrhea, nausea, vomiting, viral gastroenteritis and pyrexia (this subject had received a single dose of 400mg GSK1292263), and (2) a series of AEs that were labelled as "gastroenteritis" (this subject had received 2 doses of 250mg GSK1292263). These 2 withdrawals were not attributed to study drug by the Investigator.
- No clinically significant, study drug related changes from baseline were noted in routine blood and urine chemistry panels, complete blood count, vital signs, ECGs and physical examinations during the study.
- Because the *hERG* IC_{50} for GSK1292263 is 0.746 μM and 1 dog in the cardiovascular study had a 3-13% increase in the rate-corrected QT interval at 48-52h, a thorough review of QTc intervals during Part A of the study was performed and did not identify a clinically significant effect of GSK1292263 when blood glucose levels were stable. In addition, there were no clinically significant abnormalities on telemetry with doses up to 400mg (over a monitoring period of 48 hours) in Part A. In Part D (250mg dosed for 2 days), telemetry was performed for 60 hours, beginning 1 hour prior to dosing on Day 2, and in Part E (250mg dosed for 5 days) telemetry was performed for 24 hours, beginning 1 hour prior to dosing on Days 1, 3 and 5. No clinically significant electrocardiographic abnormalities were detected during these observation periods.
- In Part C, 1 subject was noted to have intermittent, clinically significant hematuria and proteinuria on urinalysis. On subsequent investigation, this subject was found to have a history of crystalluria.
- Some episodes of dizziness were reported by the subjects during and after the OGTT, and on occasions these were associated with low blood glucose levels either on finger stick capillary blood measurement or formal laboratory analysis. Blood glucose levels <60mg/dL were defined by the Investigator as 'hypoglycemia' AEs. The episodes of 'hypoglycemia' were mild in all cases and resolved spontaneously without dose alteration or glucose rescue. These episodes we generally more frequent 2-4h after the start of the OGTT, and when the OGTT was administered 4h after dosing. The hypoglycemic events were reduced by (i) dosing the study drug earlier, (ii) administering the OGTT 2h after dosing, and (iii) feeding the subjects lunch at ~12:30. There was no clear relationship to the dose of GSK1292263. Some episodes of hypoglycemia occurred after the subjects had received placebo. Of note,

there was no increased propensity to hypoglycemia when GSK1292263 250mg was co-administered with sitagliptin 100mg.

[Note: there is error in Table 9 in the Investigator Brochure supplement that emerged during QC of the preliminary data – the number of adverse events of hypoglycemia in Part E should be 0, and not 5 events as in the table]

1.2.1.3. Pharmacokinetic Summary

- GSK1292263 was readily absorbed following single dose administration with the median Tmax achieved between 2-8 hours post-dose across different dose levels.
- The mean t½ of GSK1292263 ranged from 12 to 20 hours, and was not dose dependent or affected by food intake.
- GSK1292263 exhibited dose-dependent pharmacokinetics with a less than dose proportional increase in plasma exposure as doses increased above 100mg.
- The tablet and PIB formulation produced similar plasma exposures when administered in the fasting state.
- Following administration of GSK1292263 30min before a breakfast meal there was an increase in plasma exposure by 2- to 3-fold relative to dosing in the fasted state (pre-OGTT).
- GSK1292263 did not affect the pharmacokinetics of sitagliptin, but sitagliptin 100mg increased the exposure of GSK1292263 by ~50%.
- Plasma GSK1292263 exposures in Part D were reduced by ~30% on Day 2 compared to Day 1, whereas simulations based on single-dose PK had predicted an increase in exposure of approximately 25%.
- In Part E, the PK profile of GSK1292263 did not confirm the reduced exposure seen on Day 2 in Part D. There was progressive accumulation of the drug as anticipated, and exposure was approximately doubled by Day 5, compared to Day 1. It should be noted that exposures on Day 1 were lower than would be predicted from exposures in Part A, but were in the expected range of variability.

1.2.1.4. Pharmacodynamic Summary

- GSK1292263 produced dose-related reductions in glucose and insulin AUC during an OGTT. Mathematical modelling of the glucose and insulin concentrations indicated that GSK1292263 increases apparent insulin sensitivity.
- GSK1292263 appeared to elevate total GLP-1 and PYY (from gut L cells), GIP (from gut K cells), and glucagon (from pancreatic islet alpha cells) during the prandial period. This corresponds to the distribution of GPR119 receptors in the human.

- Sitagliptin increased active GLP-1 levels, but reduced the concentration of total GLP-1, GIP and PYY when co-administered with GSK1292263. GSK1292263 is an incretin/PYY secretagogue and co-administration with sitagliptin increased the fraction of circulating GLP-1 that is ‘active’. There appeared to be a negative feedback of the ‘active’ incretins on secretion, resulting in the reduction of total GLP-1 and GIP when GSK1292263 and sitagliptin were co-dosed.
- An investigation into the effect of GSK1292263 on gastric emptying was performed in Part E using an acetaminophen challenge. Preliminary analysis of acetaminophen PK data indicated no clear impact on gastric emptying. Additional analyses are ongoing to assess more subtle effects.

1.2.2. GPR111598 - First in T2DM Subjects: Preliminary Safety and PK Data from Parts A and B

- Part A (completed): a 5-way crossover evaluating single doses of 25, 150 and 800mg of GSK1292263, placebo and open-label sitagliptin 100mg. Subjects were administered a 75g oral glucose tolerance test 2 hours after dosing.
- Part B (completed): a 2-way crossover evaluating a single dose of 800mg GSK1292263 administered in the fed and fasting state.

It is important to note that the investigators are still blinded to the treatment allocation in this study, and only preliminary results without treatment allocation are presented below.

1.2.2.1. Part A

Safety

In Part A, 12 T2DM subjects received singles doses of 25mg and 150mg GSK1292263, and 11 received 800mg single doses of GSK1292263 (the twelfth subject was withdrawn before completing the 800mg period; see below). These doses of GSK1292263 were safe and well tolerated.

Points of note:

- There was no association of study drug to significant AEs (see Table 1).
- There was one AE of asymptomatic hypoglycemia several hours after lunch on glucometer testing (48mg/dL) that was not observed on repeat laboratory testing.
- There were episodes of blurred vision and dizziness that were not associated with hypoglycemia and in some cases related to rapid glucose changes during the OGTT.
- There were no clinically significant changes in vital signs.
- There were no clinically significant ECG or telemetry changes, including QTc.
- There were no clinically significant safety lab changes, other than increased blood glucose levels typical of T2DM subjects and one instance of elevated Mg^{2+} that was considered to be a laboratory error.

One subject was withdrawn from the study in Period 1 because of severe hyperglycemia during Day 1 after receiving sitagliptin 100mg. On further questioning it was discerned that this subject was not drug naïve, as required for enrolment into the study, and had taken oral anti-diabetic medications and insulin in the past. This subject was replaced.

Another subject was withdrawn on Day -1 prior to dosing in Period 4 because of a positive cotinine test and high fasting blood glucose. The subject had completed Periods 1-3. This subject was not replaced.

Table 1 Most Frequent Adverse Events For GPR111598 (Part A)

	Single Dose		
Most Frequent Adverse Events	Placebo<br>N=11	Sitagliptin<br>N=12	GSK1292263<br>N=12
	n (%)		
Any AE	2 (18%)	2 (17%)	2 (17%)
Any AE related to investigational product	1 (9%)	0	1 (9%)
Most Common AEs:			
Headache	2 (18%)	0	2 (17%)
Blurry Vision	0	1 (9%)	2 (17%)
Hyperglycemia	0	1 (9%)	0

Pharmacokinetics

Table 2 and Table 3 show the preliminary AUC and Cmax PK data for GSK1292263 after singles doses of 25, 150 and 800mg administered in the fasted state 2h before an OGTT. Note that complete PK profiles are available from 12 subjects who received 25mg and 150mg and from 11 subjects who received 800mg.

Table 2 Preliminary AUC(0-24) Following Single Doses of GSK1292263 (Fasted)

Dose (mg)	AUC(0-24h), ng.h/mL (CV%)		Exposure ratio relative to mean exposure at NOAEL for 14-day toxicity study [1]	Exposure ratio relative to individual exposure at NOAEL for 14-day toxicity study [2]
25	537 (20)		92	101
150	1748 (32)		28	24
800	4109 (25)		12	13

1. Mean AUC(0-24h) limit = 49400 ng.h/mL
2. Individual AUC(0-24h) limit = 72700 ng.h/mL – Based on maximum individual exposure

Table 3 Preliminary Cmax Following Single Doses of GSK1292263 (Fasted)

Dose (mg)	Cmax, ng/mL (CV%)		Exposure ratio relative to mean exposure at NOAEL for 14-day toxicity study [1]	Exposure ratio relative to individual exposure at NOAEL for 14-day toxicity study [2]
25	53 (17)		51	50
150	175 (32)		15	13
800	393 (27)		7	6

1. Mean Cmax limit = 2693 ng/mL
2. Individual Cmax limit = 3585 ng/mL – Based on maximum individual exposure

1.2.2.2. Part B

The four T2DM subjects in Part B received 800mg in the fasted and fed state. Three subjects were drug naïve, and the fourth was washed off metformin.

Safety

Points of note:

- There was no association of study drug to significant AEs.
- There were no clinically significant changes in vital signs.
- There were no clinically significant ECG or telemetry changes, including QTc, except for 1 short episode of Wenckebach AV block that occurred at ~3am and resulted in 1 dropped QRS complex.
- There were no clinically significant safety lab changes, other than increased blood glucose levels typical of T2DM.

Pharmacokinetics

Table 4 and Table 5 summarize the preliminary AUC and Cmax PK data for GSK1292263 after single doses of 800mg in fasted and fed states. Note that complete PK profiles are available from all 4 subjects in this period. Across subjects, GSK1292263 AUC(0-24h) increased by 2.6- to 4.5-fold in the fed state and Cmax increased 1.3- to 4.7-fold in the fed state. Mean AUC(0-24h) and Cmax increased 3.8- and 3.1-fold, respectively, in the fed state.

Table 4 Preliminary AUC(0-24) Following Single Doses of 800mg GSK1292263 (Fed and Fasted)

Dose (mg)	Meal	AUC(0-24h), ng.h/mL (CV%)	Exposure ratio relative to mean exposure at NOAEL for 14-day toxicity study [1]	Exposure ratio relative to individual exposure at NOAEL for 14-day toxicity study [2]
800	Fasted	3429 (22)	14	17
800	Fed	12997 (29)	4	4

1. Mean AUC(0-24h) limit = 49400 ng.h/mL
2. Individual AUC(0-24h) limit = 72700 ng.h/mL – Based on maximum individual exposure

Table 5 Preliminary Cmax Following Single Doses of 800mg GSK1292263 (Fed and Fasted)

Dose (mg)	Meal	Cmax, ng/mL (CV%)	Exposure ratio relative to mean exposure at NOAEL for 14-day toxicity study [1]	Exposure ratio relative to individual exposure at NOAEL for 14-day toxicity study [2]
800	Fasted	351 (27)	8	8
800	Fed	1009 (37)	3	3

1. Mean Cmax limit = 2693 ng/mL
2. Individual AUC(0-24h) limit = 3585 ng/mL – Based on maximum individual exposure

1.2.2.3. Doses of GSK1292263 Selected for Evaluation in Part C

Based on the safety, tolerability and PK from Parts A and B, the actual doses of GSK1292263 being administered in Part C are: 50mg BID, 150mg BID, 300mg BID and 600mg QD.

1.3. Sitagliptin

In this study, open-label sitagliptin is being used as a comparator to allow estimation of the relative efficacy of GSK1292263 on glycemic parameters. A single dose of open-label sitagliptin will be co-administered with GSK1292263 or placebo (placebo arm) on Day 14 in Part C (Cohorts 3 and 4) to determine the extent of pharmacokinetic and pharmacodynamic interactions in subjects with T2DM.

Sitagliptin (Januvia) is a marketed DPP-IV inhibitor that is approved for use as monotherapy or in combination therapy for the treatment of T2DM. The recommended dose is 100mg once daily, which can be taken without regard to food.

The pharmacokinetics of sitagliptin are generally similar between healthy volunteers and T2DM subjects, with tmax occurring at 1-4 h, t½ approximately 12 hours, and AUC and Cmax approximately 3468ng.h/mL and 387ng/mL, respectively following a single 100mg dose.

Sitagliptin is mainly excreted unchanged in the urine (79%) with limited metabolism. In Part C of the FTIH study, GSK1292263 250mg did not affect the pharmacokinetics of sitagliptin, but sitagliptin 100mg increased the exposure of GSK1292263 by ~50%. The mechanism of the elevation in GSK1292263 exposure is unknown.

Additional information may be found in the sitagliptin Prescribing Information [**Januvia** Package Insert, 2007].

By blocking DPP-IV, sitagliptin increases the systemic levels of the active form of the incretins, GLP-1 and GIP, and reduces the level of PYY_{3-36}, a form that has anorexigenic properties. At the same time it reduces the levels of total GLP-1, GIP and PYY via a putative negative feedback loop at the secretory L and K cells in the gut. In the FTIH study (GPR111596), a reduction in total GLP-1, GIP and PYY was observed when 250mg GSK1292263 was co-administered with 100mg sitagliptin to normal healthy subjects, compared to the levels observed with GSK1292263 alone.

In a recent clinical study in patients with T2DM, single-doses of sitagliptin caused a near maximal glucose-lowering efficacy [Herman, 2006]. Following 25 and 200mg single doses of sitagliptin to T2DM subjects, a 1.4- to 2-fold augmentation of active GLP-1 and GIP levels were observed post-OGTT. This was accompanied by ~25% reduction in Glucose AUC. Thus, an effect after administration of a 100mg single dose is expected.

1.4. Rationale

Data from this study will be used to assess the potential of the GPR119 agonist GSK1292263 as a treatment for T2DM, and will aid the design and dose selection of future studies of longer duration in T2DM subjects that will evaluate GSK1292263 alone or in combination with other anti-diabetic drugs, such as a DPP-IV inhibitor or metformin.

1.4.1. Study Rationale

This is the second clinical study conducted with GSK1292263. As described in Section 1.2.1, GSK1292263 has been tested in healthy volunteers as single doses from 10-400mg, as multiple doses of 250mg (2 or 5 days administration), and when co-administered as a single 250mg dose with sitagliptin 100mg.

This will be the first use of GSK1292263 in T2DM subjects, and is intended to assess the safety, tolerability, pharmacokinetic and pharmacodynamic profile of this investigational drug following single doses (Parts A [Cohort 1] and B [Cohort 2]), and subsequently 14 days of dosing in Part C.

There are two scenarios for frequency of dosing in Part C of this study, summarized below:

Scenario 1: QD dosing Part C, Cohort 3 and BID dosing in Part C, Cohort 4

Scenario 2: BID dosing in Part C, Cohort 3 and QD dosing in Part C, Cohort 4

The actual scenario of dosing (QD or BID) in Part C will be determined based on preliminary PK/PD, safety and tolerability data in Parts A and B.

In Parts A and C, open-label sitagliptin is included as a comparator to provide data that will aid the assessment of the therapeutic potential of GSK1292263. Part B is being conducted to provide an estimate, in T2DM, of the magnitude of the effect of food on the PK of GSK1292263 at the highest dose that will be used in the study. If necessary, this will allow refinement of the doses selected for Part C. (See the SPM for timings of meals in relation to dosing.)

In Part C, subjects randomized to GSK1292263 or placebo will have study drug co-administered with a single dose of 100mg of sitagliptin on Day 14 to determine the extent of pharmacokinetic and pharmacodynamic interactions in subjects with T2DM. For BID dosing regimens, sitagliptin will be co-administered with the first daily dose of GSK1292263 on Day 14. In the FTIH study GPR111596, sitagliptin increased the exposure of GSK1292263 by ~50%, but GSK1292263 did not alter the exposure of sitagliptin. The pharmacokinetics of both GSK1292263 and sitagliptin will be assessed on Day 14 in Part C.

Inhibition of DPP-IV by sitagliptin increases the circulating concentrations of active GLP-1 after nutrient stimulation and this may result in a further augmentation of the levels of active incretins when co-administered with GSK1292263. On the other hand, the DPP-IV inhibitor may reduce the levels of circulating $PYY_{[3-36]}$ and $GLP_{[9-36]}$ when co-administered with GSK1292263 because DPP-IV cleaves inactive $PYY_{[1-36]}$ to form active $PYY_{[3-36]}$, as well as active $GLP_{[7-36]}$ to form $GLP_{[9-36]}$, which also has biological effects.

It is important to note that the current study GPR111598 with T2DM subjects offers the opportunity to safely investigate doses higher than 400mg, the highest dose evaluated in the FTIH study GPR111596.

This is important for 3 reasons:

1. To establish the safety and tolerability of a dose (800mg) that is a substantial multiple of the predicted therapeutic dose (currently approximately 100mg). This is relevant to the conduct of an adequate ‘Thorough ECG’ study in the future.

2. It provides an opportunity to investigate the pharmacological effects, if any, of a high dose that is predicted to increase systemic exposure by an amount that is less than dose proportional in the fed and fasted states (See Figure 2). This may allow further understanding of the relative importance of systemic exposure versus local exposure in the g astrointestinal tract for the pharmacological effects of a GPR119 agonist, and will aid in the development of future non-absorbable molecules if the efficacy is mediated primarily by mechanisms in the gut lumen.
3. The data from the FTIH study indicate that the effect of food on systemic exposure is dose-dependent. The 800mg dose administered with and without food in Part B will allow further understanding of the effect of food and safety/tolerability at a dose that may be evaluated in Part C when subjects will be administered the study drug with food.

Based on emergent data, there may also be an adjustment of the dosing regimen in the GSK1292263 arms of Cohort 3 in Part C to allow more extensive evaluation of BID doses. See Figure 1.

While the maximum allowable total daily dose in the current study was 800mg based on the drug substance used in this study that may contain a trichloromethyloxadiazole process impurity, GSK2116107A, which was mutagenic in a mini-Ames test (Amendment No. 2), Amendment No. 4 sets the new maximum total daily dosage for Part C in this protocol at 600mg based on the preliminary data from the 13 week toxicology study in the dog. (See Section 1.5.2).

Figure 1 Study Design

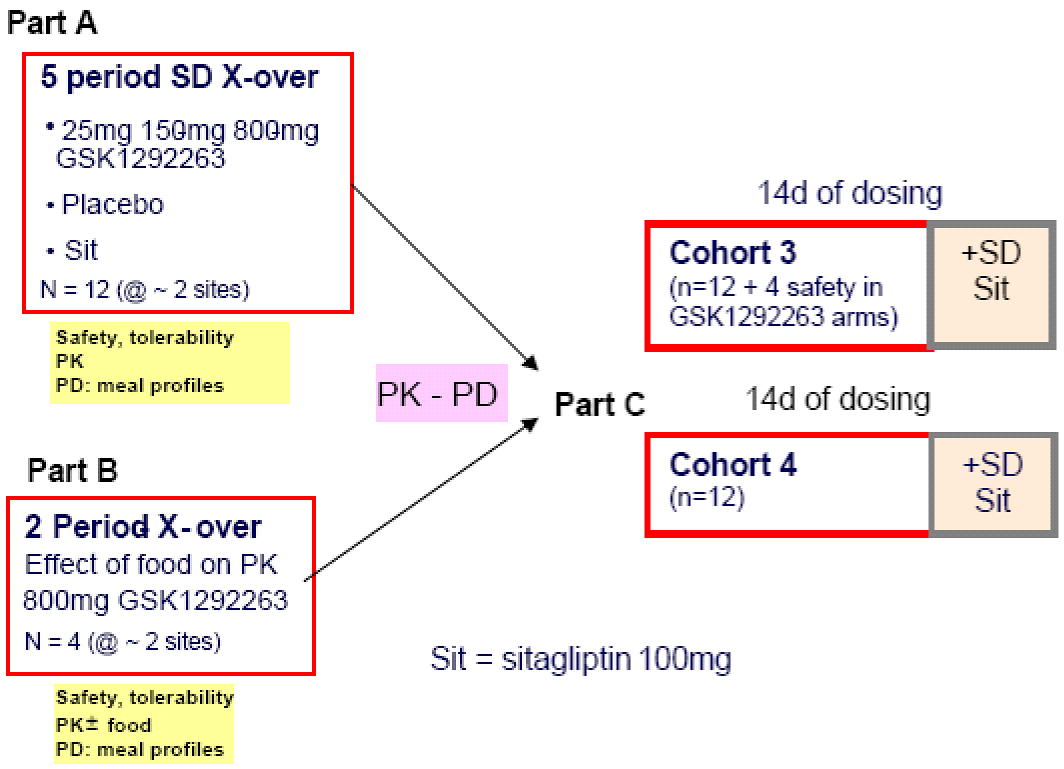

PART C	Example of QD/BID Dosing Scenario	Example of BID/QD Dosing Scenario
Cohort 3	25mg QD<br>(n=12 + 4 safety)	25mg BID<br>(n=12 + 4 safety)
	150mg QD<br>(n=12 + 4 safety)	150mg BID<br>(n=12 + 4 safety)
	800mg QD<br>(n=12 + 4 safety)	400mg BID<br>(n=12 + 4 safety)
	Placebo QD<br>(n=12)	Placebo BID<br>(n=12)
	Sitagliptin 100mg QD<br>(n=12)	Sitagliptin 100mg QD<br>(n=12)
Cohort 4	400mg BID<br>(n=12)	800mg QD<br>(n=12)

1.4.2. Dose Rationale

1.4.2.1. GSK1292263

In the FTIH study GPR111596, there were dose-related reductions in glucose and insulin AUC during an OGTT, and apparent elevation of total GLP-1, GIP and PYY during the prandial period. Interestingly, one subject with impaired glucose tolerance (IGT) showed a reduction of ~30% (versus placebo) of Glucose $AUC_{0-120min}$ during an OGTT at a dose of 50mg, compared to a reduction of ~13% of Glucose $AUC_{0-120min}$ for normal volunteers at 400mg.

Because IGT is considered a pre-diabetic condition with similar, but less severe dysglycemia, it is anticipated that a dose of 50mg will produce a reduction of the glucose AUC in T2DM subjects.

With the data from the FTIH study (GPR111596), modeling and simulation has been used to determine the optimal doses that would best characterize the dose/exposure-response of GSK1292263 in this study.

1.4.2.2. Predicted Maximum Exposures Relative to Safety Limits

Based on the results of non-clinical toxicology studies (see Investigator Brochure [GlaxoSmithKline Document Number RM2008/00434/00]), exposures in the current study were not to exceed a mean steady-state AUC(0-24h) of 49,400ng.h/mL and mean Cmax of 2693ng/mL which are 80% of the gender-averaged no-observed-adverse-effect-level (NOAEL) mean exposure in beagle dogs at Day 14 at the highest dose of 1000mg/kg/day. Furthermore, no individual was to exceed an AUC(0-24h) of 72,700ng.h/mL or Cmax of 3585ng/mL, which are 80% of the highest AUC and Cmax observed in dog at 1000mg/kg NOAEL dose group in the 14 day toxicology study. These exposures were consistent with limits that were approved for the conduct of the FTIH study, GPR111956, by the GSK Global Safety Board and the local IRB, and were not objected to by the FDA.

In this Amendment No. 4, preliminary data from the 13-week toxicology study in the dog are summarized in Section 1.5.2.1 and Section 1.5.2.3. Briefly, three of four males given 1000mg/kg/day and one male given 20 mg/kg/day had minimal to mild degeneration/depletion of seminiferous epithelium in the testes. The AUC(0-24h) and Cmax associated with the no effect threshold for testicular findings were 36772ng.h/mL and 2537ng/mL, respectively, following 13 weeks of dosing. The lowest individual AUC(0-24h) and Cmax for the dog testicular effect were 48,863ng.h/mL and 2757 ng/mL, respectively, following 13 weeks of dosing.

Adverse GSK1292263-related changes were also observed in the liver. Moderate to marked increases in ALT (3.3X to 11.3X in males, 4.3X to 11.7X in females based on individual animal values relative to pretest) and marked increases in GLDH (4.4X to 22.3X in males, 5.8X to 15X in females based on individual animal values relative to pretest) were observed at 1000 mg/kg/day. There was no hepatocellular necrosis evident in any of the dogs given 1000 mg/kg/day so there was no clear histopathology correlate explaining the ALT and GLDH elevations. While the maximum allowable total daily dose in the current study was 800mg based on the potential impurity in the drug substance (Amendment No. 2; see Section 1.5.2), Amendment No. 4 sets the new maximum total daily dosage for Part C in this protocol at 600mg based on the preliminary data from the 13 week toxicology study in the dog. A dose of 300mg BID, or a dosing regimen resulting in equivalent exposure, given for up to 14 days does not pose a significant clinical risk from the perspective of either mean or individual exposures to date.

GSK1292263 exhibited dose-dependent PK in the FTIH study. As doses were increased a clear plateau in exposure was observed in the fasted state. Dosing with food increased GSK1292263 exposure in a dose-dependent manner with maximal increase in exposure of 2- to 3-fold relative to the fasted pre-OGTT state [GlaxoSmithKline Document Number RM2009/00168/00].

Figure 2 Effect of Dose and Food on GSK1292263 Exposure in the FTIH Study

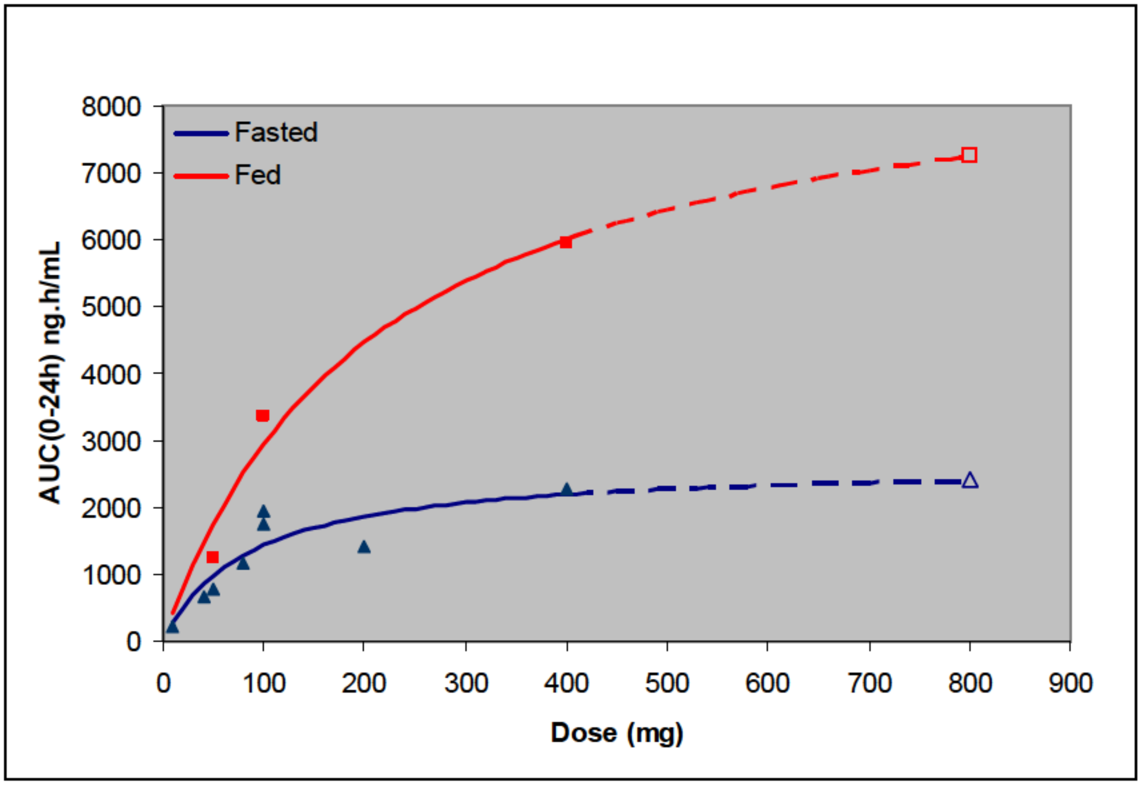

To estimate maximum potential exposures relative to non-clinical safety findings, simulations were performed for two scenarios:

1) exposures plateau as seen in the FTIH study with a dose-dependent food effect, and

2) no plateau is observed and a dose proportional increase in exposure is observed relative to 400mg exposures in the FTIH study.

Amendment No. 4 Note: For both of the following simulation scenarios, please refer to Section 1.4.2.5 and Section 1.5.2 for preliminary exposure data from Part C of this study and the 13 week toxicology studies in the rat and dog.

Scenario 1:

In Parts A and B, a maximum single dose of 800mg may be administered in the fasted (Parts A and B) or fed (Part B) states. A single dose of 800mg administered in the fasted or fed state is predicted to provide mean AUC(0-24) of ~2404 and 7243ng.h/mL, respectively, and Cmax of ~155 and 529ng/mL, respectively. In Part C an 800mg once daily dose administered for 14 days (fed) is predicted to achieve a steady-state AUC(0-24) and Cmax of 14,485ng.h/mL and 1058ng/mL, respectively. For a 400mg dose administered BID in Part C (fed only), the predicted steady-state AUC(0-24h) and Cmax are 25,789 and 1602ng/mL, respectively. The predicted maximum mean exposures in this study remain below non-clinical toxicology exposure limits. Further, no individual is

predicted to exceed the individual AUC(0-24) of 72,700ng.h/mL, which is 80% of the highest AUC and Cmax observed in dog at 1000mg/kg NOAEL dose group in the 14-day toxicology study. Following administration of 400mg BID at steady-state a small percentage of subjects (~2%) are predicted to slightly exceed the individual Cmax of 3585 ng/mL.

Scenario 2:

To provide a conservative estimate of maximal potential exposures, a dose-proportional increase in exposure relative to 400mg exposures observed in the FTIH study was considered. Following a single dose of 800mg in Parts A and B, the projected fasted and fed single-dose AUC(0-24) values are 4568 and 11873ng.h/mL, respectively, and Cmax values are ~379 and 916ng/mL, respectively. In Part C an 800mg dose administered daily for 14 days (fed state only) is predicted to achieve steady-state AUC(0-24h) and Cmax of 23,756ng.h/mL and 1831ng/mL, respectively. The maximum potential BID dose to be administered in Part C is 400mg. As a result, the maximum anticipated exposures for Scenario 1 and Scenario 2 are comparable (see Scenario 1 for predicted exposures). In Scenario 2, the predicted mean exposures for the maximum allowable dose in this study remain below non-clinical toxicology exposure limits. Further, no individual is predicted to exceed the individual AUC(0-24) of 72700ng.h/mL, which is 80% of the highest AUC and Cmax observed in dog at 1000mg/kg NOAEL dose group in the 14-day toxicology study. Under this scenario, following 400mg BID and 800mg daily at steady-state a small percentage (2-3%) of subjects are predicted to slightly exceed the individual Cmax of 3585 ng/mL.

It is important to note that the known solubility-limited absorption and observed pharmacokinetic profile of GSK1292263 in the FTIH study makes Scenario 1 more likely to occur.

1.4.2.3. Planned Doses and Predicted Exposures

Given the known solubility-limited absorption profile of GSK1292263 and the modeling discussed in Section 1.4.2.2, all exposures presented below are based on Scenario 1 and assume a clear plateau in exposure in the fasted state with a dose-dependent food effect.

Part A

Modeling and simulation was performed to select 3 doses most likely to adequately characterize the dose/exposure-response of GSK1292263 in Part A. The planned doses of GSK1292263 for Part A are 25, 150 and 800mg. These doses may be modified based on emergent safety, tolerability and PD data from earlier periods of Part A. The 800mg dose is being evaluated to determine if the higher dose in the gastrointestinal tract produces greater PD effects through direct luminal mechanisms that do not involve systemic exposure.

Doses up to 400mg in the fasted and fed states have been evaluated in normal volunteers and have been shown to be safe and generally well tolerated in study GPR111596. This will be the first administration of GSK1292263 at a dose above 400mg to a human

volunteer. However, all exposures are predicted to remain below limits established by non-clinical toxicology data.

The predicted exposures relative to non-clinical toxicology limits are summarized in Table 6.

Table 6 Predicted Exposures for Single Doses (Part A)

Dose (mg)[4]	Predicted Single-Dose Exposure[1]		Exposure Ratio Relative to Exposure at NOAEL for 14-day Toxicity Study [2,3]	
	AUC(0-24h) (ng.h/mL)	Cmax (ng/mL)	AUC(0-24h)	Cmax
25	603	75	82	36
150	1696	135	29	20
800	2404	155	21	17

1. Predictions based on fasted exposure in FTIH study (GPR111596)
2. AUC(0-24h) limit = 49,400 ng.h/mL, Cmax limit = 2,693 ng/mL 80% of NOAEL in dogs at 1000mg/kg/day
3. No individuals predicted to exceed AUC(0-24h) limit of 72,700 ng.h/mL and Cmax of 3585ng/mL, which are 80% of the highest AUC and Cmax observed in dog at 1000mg/kg NOAEL dose group
4. Predictions based on PK in the fasted state

PK/PD analysis will be performed on preliminary data from Part A to characterize the dose/exposure-response relationship for GSK1292263 in T2DM subjects. Along with safety and tolerability data, this analysis will be used to confirm or modify the planned doses for Parts B and C.

Part B

The planned dose for Part B is 800mg administered in the fasted and fed states. The actual dose to be administered in Part B may be adjusted based on PK/PD and safety/tolerability data in Part A. However, if dose adjustment is required, the maximum dose to be administered in Part B will not exceed 800mg, and will be selected to maintain exposures within limits based on non-clinical toxicology. Predicted exposures in Part B are summarized in Table 7.

Table 7 Predicted Exposures for GSK1292263 Fasted and Fed Dosing (Part B)

Dose (mg)	Predicted Single-Dose Exposure[1]		Exposure Ratio Relative to Exposure at NOAEL for 14-day Toxicity Study [2,3]	
	AUC(0-24h) (ng.h/mL)	Cmax (ng/mL)	AUC(0-24h)	Cmax
800 (Fasted)	2404	155	21	17
800 (Fed)	7243	529	7	5

1. Predictions based on fasted exposure in FTIH study (GPR111596)
2. AUC(0-24h) limit = 49,400 ng.h/mL, Cmax limit = 2,693 ng/mL 80% of NOAEL in dogs at 1000mg/kg/day
3. No individuals predicted to exceed AUC(0-24h) limit of 72,700 ng.h/mL and Cmax of 3585ng/mL, which are 80% of the highest AUC and Cmax observed in dog at 1000mg/kg NOAEL dose group

Part C – Once Daily (QD) Dosing (See Section 1.4.2.5 for Preliminary Exposure Data from Part C)

If GSK1292263 is dosed QD, the planned doses for Cohort 3 of Part C are 25, 150, and 800mg administered once daily for 14 days in the fed state, and Cohort 4 will evaluate half the maximum QD dose administered twice daily (see below). The predicted steady-state exposures for the planned doses relative to non-clinical toxicology limits for these doses are summarized in Table 8. The final doses to be administered may be modified to ensure that exposures do not exceed exposure limits based on nonclinical toxicology data and based on PK/PD, safety and tolerability data from Parts A and B. For co-administration of GSK1292263 with sitagliptin on Day 14 it is assumed that steady-state AUC(0-24h) and Cmax will increase by ~56% and ~35%, respectively, compared to GSK1292263 alone on Day 13. These assumptions are based on increases in exposure observed in the FTIH study following single-dose co-administration of GSK1292263 and sitagliptin. Predicted co-dosing exposures are conservative (highest exposure) given that GSK1292263 and sitagliptin will only be administered as a single dose on Day 14 after GSK1292263 has been administered alone for 13 days. It should be noted that GSK1292263 did not affect the pharmacokinetics of sitagliptin in the FTIH study. PK/PD analysis will be performed on preliminary data from Parts A and B to characterize the dose/exposure-response relationship for GSK1292263. Along with safety and tolerability data, this analysis will be used to confirm the planned doses for Part C.

Based on the preliminary toxicokinetic data from the 13 week toxicology study in the dog (Section 1.5.2.3), any adjustments to QD doses in Part C will not exceed 600mg total daily dose, or a dosing regimen resulting in equivalent exposure.

Table 8 Predicted Steady-State Exposures for GSK1292263 Daily Dosing (Part C)

Dose (mg)[4]	Predicted Steady-State Exposure[1]		Exposure Ratio Relative to Exposure at NOAEL for 14-day Toxicity Study [2,3]	
	AUC(0-24h) (ng.h/mL)	Cmax (ng/mL)	AUC(0-24h)	Cmax
25	1951	215	25.0	12.5
100	5911	562	8.4	4.8
400	11999	940	4.0	2.9
800	14485	1058	3.4	2.5
400 + Sitagliptin	18718	1268	2.6	2.1
800 + Sitagliptin	22598	1429	2.2	1.9

1. Predictions based on fasted exposure in FTIH study (GPR111596)
2. AUC(0-24h) limit = 49,400 ng.h/mL, Cmax limit = 2,693 ng/mL 80% of NOAEL in dogs at 1000mg/kg/day
3. No individuals predicted to exceed AUC(0-24h) limit of 72,700 ng.h/mL and Cmax of 3585ng/mL (80% of the highest AUC and Cmax observed in dog at 1000mg/kg NOAEL dose group), assuming plateau of exposures
4. Predictions based on PK in the fed state. Co-administration with sitagliptin (100mg) is assumed to increase steady-state AUC(0-24h) and Cmax by 56% and 35%, respectively, based on single-dose PK in FTIH study

Part C - Twice-Daily (BID) Dosing (Cohort 3 or 4) (See Section 1.4.2.5 for Preliminary Exposure Data from Part C)

PK/PD analysis will be performed on preliminary data from Parts A to characterize the dose/exposure-response relationship for GSK1292263. If Cohort 3 in Part C is dosed QD, then Cohort 4 will be conducted to evaluate half the maximum QD dose used in Cohort 3 administered twice a day for 14 days.

Based on the preliminary data from the 13 week toxicology study in the dog, the maximum dose level for twice daily dosing is 300mg BID (total daily dose – 600mg). However, based on safety, tolerability and PK/PD analysis from Part A, B or C, the BID dose level may be reduced. A dose of 300mg BID, or a dosing regimen resulting in equivalent exposure, given for up to 14 days does not pose a significant clinical risk from the perspective of either mean or individual exposures to date [see Table 8 (PK data from Part C) and Table 11 (PK for the 13-week toxicology studies)].

Predicted steady-state exposures following a dose of 400mg BID for 14 days in the fed state are summarized in Table 9. For co-administration of GSK1292263 with sitagliptin on Day 14 it is assumed that steady-state AUC(0-24h) and Cmax will increase by ~56% and ~35%, respectively, compared to GSK1292263 alone on Day 13. These assumptions are based on increases in exposure observed in the FTIH study following single-dose co-administration of GSK1292263 and sitagliptin. Predicted co-dosing exposures are conservative (highest exposure) given that GSK1292263 and sitagliptin will only be administered as a single dose on Day 14 after GSK1292263 has been administered alone for 13 days. It should be noted that GSK1292263 did not affect the pharmacokinetics of sitagliptin in the FTIH study. All exposures are predicted to remain below the group mean exposure limits based on non-clinical toxicology data. No subjects are predicted to exceed the individual exposure limits based on non-clinical 14-day toxicology data (see Section 1.4.2.5 and Section 1.5.2.3 for preliminary exposure data from Part C and the 13-week toxicology studies in the rat and dog).

Based on emergent data from Parts A and B, BID dosing of GSK1292263 may be investigated in Cohort 3 by administering the doses of GSK1292263 or placebo as a BID regimen, keeping the dose and exposure limits outlined above. In this case Cohort 4 will evaluate a QD dose of GSK1292263 that will match the total daily dose of the highest BID regimen in Cohort 3.

Table 9 Predicted Steady-State Exposures for GSK1292263 BID Dosing (Part C)

BID Dose (mg)[4]	Predicted Steady-State Exposure[1]		Exposure Ratio Relative to Exposure at NOAEL for 14-day Toxicity Study [2,3]	
	AUC(0-24h) (ng.h/mL)	Cmax (ng/mL)	AUC(0-24h)	Cmax
400	25789	1602	1.9	1.7
400mg + Sitagliptin	40230	2163	1.2	1.2

1. Predictions based on fasted exposure in FTIH study (GPR111596)
2. Mean AUC(0-24h) limit = 49,400 ng.h/mL, Cmax limit = 2,693 ng/mL 80% of NOAEL in dogs at 1000mg/kg/day
3. No subjects predicted to exceed individual AUC(0-24h) limit of 72,700 ng.h/mL (80% of the highest AUC and Cmax observed in dog at 1000mg/kg NOAEL dose group). Approximately 2% of subjects predicted to slightly exceed individual Cmax limit of 3585 ng/mL
4. Predictions based on PK in the fed state. Co-administration with sitagliptin assumed to increase steady-state AUC(0-24h) and Cmax by 56% and 35%, respectively, based on single-dose PK in FTIH study

1.4.2.4. Preliminary Human Exposures from the Ongoing Part C and the Toxicokinetics from the 13-Week Toxicology Study in the Dog

In the 13-week toxicology study in the dog, three of four males given 1000mg/kg/day and one male given 20mg/kg/day had minimal to mild degeneration/depletion of seminiferous epithelium in the testes. The AUC(0-24h) and Cmax values in an individual animal associated with the no effect threshold for testicular findings were 36772ng.h/mL and 2537ng/mL, respectively. The lowest individual AUC(0-24h) and Cmax for the dog testicular effect were 48,863ng.h/mL and 2757ng/mL, respectively.

The maximum GSK1292263 exposures observed in the current study have been associated with steady-state during Part C. The final doses of GSK1292263 selected in Part C were 50 mg BID, 150 mg BID, 300 mg BID and 600 mg QD. A summary of preliminary exposures of GSK1292263 (n=6) at steady-state (Day 13 and Day 14) are presented in Table 10.

Table 10 Summary of Preliminary GSK1292263 Exposure at Steady-State in Part C

Dose	Day 13		Day 14	
	AUC(0-24h) (ng.h/mL) Mean ±SD [min, max]	Cmax (ng/mL) Mean ±SD [min,max]	AUC(0-24h) (ng.h/mL) Mean ±SD [min, max]	Cmax (ng/mL) Mean ±SD [min, max]
50mg BID	7944 ± 1362 [5357, 9203]	429 ± 58 [396, 536]	8153 ± 1240 [5758, 9331]	452 ± 53 [369, 515]
150mg BID	16986 ± 5863 [12449, 27137]	985 ± 312 [691, 1517]	17257 ± 5656 [12789, 26895]	952 ± 304 [717, 1479]
300mg BID	23101 ± 7900 [15606, 35959]	1258 ± 376 [912, 1885]	23810 ± 8517 [15585, 38771]	1222 ± 343 [863, 1834]
600mg QD	9330 ± 1847 [6555, 11964]	866 ± 145 [653, 1098]	10221 ± 2519 [7333, 13870]	795 ± 207 [601, 1221]

The maximum observed group mean AUC(0-24h) and Cmax values to date are 23810 and 1258 ng/mL, respectively. Of note, (i) the highest exposures have been observed in the 300mg BID group, and (ii) there is no significant effect of sitagliptin on GSK1292263 exposures in these T2DM subjects.

1.4.2.5. Sitagliptin

Subjects in Part A will receive a single open-label dose of 100mg sitagliptin as one of the 5 dosing periods.

Subjects in Part C (Cohort 3 and Cohort 4) randomized to GSK1292263 or placebo will be co-administered with a single open-label dose of 100mg sitagliptin on Day 14. Subjects in the sitagliptin-alone arm will receive 14 consecutive days of dosing with open-label 100mg sitagliptin.

This is the recommended dose of sitagliptin for the treatment of T2DM.

1.4.3. Stopping Criteria

The target range of exposures for this study has been selected to allow for a robust characterization of safety, tolerability, PK and PD of GSK1292263 in T2DM volunteers. Based on the preliminary data from the 13-week toxicology study in the dog, the highest total daily dose of GSK1292263 to be administered in the study will be 600mg (administered as single or divided doses). A dose of 300mg BID, or a dosing regimen

resulting in equivalent exposure, given for up to 14 days does not pose a significant clinical risk from the perspective of either mean or individual exposures to date.

In Parts A and B, safety and tolerability will be evaluated after each dosing period, and the doses of GSK1292263 may be adjusted up or down from the proposed schemes presented in Table 6 and Table 7. A maximum 6.5-fold exposure escalation between the mid and high doses in Part A will be allowed because modelling and simulation indicates that (i) in normal volunteers systemic exposures plateau above 100mg in the fasted state, (ii) exposure limits based on the preclinical toxicology studies are not exceeded even assuming a conservative approach using dose proportional increase in exposure (Section 1.4.2.2).

Doses in this study may be adjusted if warranted by emergent safety and tolerability data, and/or PK data that indicate that the exposure limits presented in Section 1.4.2.2 may be exceeded.

1.5. Summary of Risk Management

1.5.1. Risks Related to Washout of Anti-Diabetic Medications

GSK1292263 has only been evaluated in conjunction with one approved anti-diabetic medication, sitagliptin. Therefore, all subjects enrolled in this study will be drug naïve or washed off of their usual anti-diabetic medications.

Because of the length of the crossover evaluation in Part A (5 periods; approximately 5 weeks), all subjects enrolled in this part will be drug naïve subjects with T2DM who are being managed with diet and exercise. This will minimize significant deterioration of glycemic control during this part of the study.

However, as there is only limited availability of drug naïve subjects with T2DM, subjects in Parts B and C will be allowed to enter the study after washing off certain anti-diabetic medications for 1 week. By enrolling subjects previously maintained only on monotherapy or sub-maximal doses of combination therapy, it is anticipated that this will not cause significant deterioration of glycemic control during the approximately 2 weeks' duration of these parts.

The risk that glycemic control will deteriorate in Part C is partially mitigated by the fact that one group of subjects will receive sitagliptin at the dose approved for the treatment of T2DM (100mg). In addition, in the FTIH study GPR111596, GSK1292263 significantly reduced the Glucose AUC in a subject with impaired glucose tolerance, a pre-diabetic condition.

1.5.2. Risks Related to GSK1292263

GSK1292263 was generally safe and well-tolerated when administered in single doses up to 400mg in the fasted and fed states, and when administered at a dose of 250mg for 2 or 5 days in healthy volunteers.

In this study, subjects will be carefully evaluated prior to, during and after dosing. In Parts A and B, they will be dosed in the unit under close supervision, and will remain there until all 24hr post-last-dose assessments, including safety, have been completed. Adverse event data, including clinically significant abnormalities of clinical labs, vital signs and ECGs will be reviewed by the medical monitor and study team, prior to dosing in each period.

In Part C, subjects will remain in-unit under close supervision from Day -2 through the morning of Day 15 (final checkout). Clinical chemistry, hematology, urinalysis, vital signs, and ECGs will be monitored at regular intervals throughout this part of study.

Glucose levels will be monitored by capillary blood glucose (CBG) or venous or blood plasma glucose at least twice daily (fasting and pre-evening meal) when the subject is in the unit and during washout of anti-diabetic medications prior to dosing a study drug. In addition, at the blood draws after dosing in Parts A and B:

- A drop of blood will be used to provide real-time glucometer readings of capillary glucose levels (no additional blood required)
- Subjects will be asked 'how are you feeling' to provide an assessment of neuroglycopenia

Subjects will have IV access post-dosing for rapid treatment of hypoglycemia, if required.

Stopping criteria for these parameters are specified in Section 1.5.3.

The risk in T2DM subjects of clinically-significant hypoglycemia due to GSK1292263 is believed to be low because:

- The mechanism relies on glucose-dependent insulin secretion.
- GSK1292263 increases glucagon secretion during insulin-induced hypoglycaemia in animals studies.
- No fasting hypoglycemia was observed 24h after dosing in the FTIH study.
- T2DM subjects should be less prone than healthy normal volunteers to hypoglycemia associated with an OGTT or Meal Tolerance Test.

Details of any hypoglycemic episodes will be captured in the Case Report Form.

As indicated in Supplement 2 of the IB ([GlaxoSmithKline Document Number RM2009/00168/01]), Amendment No. 2 set the maximum allowable total daily dose in the current study at 800mg (administered as single or divided doses) based on the possibility that the drug substance used in this study may contain a trichloromethyloxadiazole process impurity, GSK2116107A, which was mutagenic in a mini-Ames test. Quantitative assessments indicate that GSK2116107A is not present in the final drug substance at a limit of detection of 50ppm (which based on a clinical dose of 800 mg/day, equates to a total oral dose of less than 40 μg/day). There are no safety concerns for the conduct of a clinical trial at doses up to 800mg/day for up to 28 days because the level of this impurity is below the staged TTC (Threshold of Toxicological

Concern) limit of 60µg/day for clinical trials of up to one month duration. Amendment No. 4, however, limits the maximum total daily dose allowed in the current study to 600mg based on the preliminary data from the 13 week toxicology study in the dog (Section 1.5.2).

Overall, the potential risk to subjects who receive GSK1292263 is likely to be very small because:

- The likelihood of the plasma exposures exceeding NOAEL limits is small based on FTIH and preliminary Part A, B and C PK data from the current study (see Section 1.4.2 and Section 1.5.2.
- So far, GSK1292263 has been safe and well tolerated in normal human subjects at doses ≤ 400mg, with no clinically significant changes in vital signs, ECGs, telemetry and lab parameters related to the study drug.
- The subjects are dosed in the clinical unit and observed closely for 24h.
- There is no indication that the mechanism of action of this drug would predict an increased risk of hypoglycaemia in subjects with T2DM. In addition:
  - Part A uses the dosing-OGTT paradigm that reduced hypoglycemic episodes in the FTIH study GPR111596
  - At the request of the IRB, in Part A and B the staff at the clinical site will be monitoring blood glucose in 'real time' using a glucometer, as indicated in Amendment 1 of the protocol GPR111598 ([GlaxoSmithKline Document Number RH2008/00140/01])
  - Subjects will be asked 'how are you feeling' to provide an assessment of neuroglycopenia.
- The drug substance used in this study has a lower level of impurities than that used in the FTIH study.

1.5.2.1. Preliminary Data from 13-week Toxicology Study in the Dog

In the 13-week dog study (4, 20, 1000 mg/kg/day), preliminary histopathological evaluation revealed adverse GSK1292263-related changes in (i) testes (minimal to slight tubular degeneration/depletion and Leydig cell hypertrophy) at ≥ 20mg/kg/day, and (ii) in the liver (elevated ALT and GLDH in both sexes; minimal inflammation in males) at 1000 mg/kg/day. There were no treatment-related findings in the liver or testes in the dog at 4 mg/kg/day.

<u>Testis</u>:

Three of four males given 1000mg/kg/day and one male given 20mg/kg/day had minimal to mild degeneration/depletion of seminiferous epithelium in the testes. The change primarily affected spermatids in the superficial layers of the seminiferous epithelium indicating the test article effect was occurring in the later steps of the spermatogenic process while sparing the early spermatogonial stem cells. This suggests reversibility is likely to occur on cessation of treatment.

Liver:

Adverse GSK1292263-related changes were observed in the liver (increased ALT and GLDH in both sexes; minimal inflammation in males) at 1000mg/kg/day. No recovery groups were included on this study. Moderate to marked increases in ALT (3.3X to 11.3X in males, 4.3X to 11.7X in females based on individual animal values relative to pretest) and marked increases in GLDH (4.4X to 22.3X in males, 5.8X to 15X in females based on individual animal values relative to pretest) were observed at 1000mg/kg/day, with changes increasing in severity between Week 4 and Week 13. Although minimal inflammation with a mixed inflammatory cell population was present in males given 1000mg/kg/day, it was not present in females although females had higher enzyme elevations. There was no hepatocellular necrosis evident in any of the dogs given 1000mg/kg/day so there was no clear histopathology correlate explaining the ALT and GLDH elevations.

1.5.2.2. Preliminary Data from 13-week Toxicology Study in the Rat

In the 13-week rat study (10, 150, 2000mg/kg/day) there were no treatment-related findings in liver or testes up to 2000 mg/kg/day (mean male rat AUC0-24hr of 43,551ng·hr/mL).

1.5.2.3. Toxicokinetic Parameters from the 13-Week Toxicology Studies in Rat and Dog

The toxicokinetic parameters are shown in Table 11.

Table 11 Comparative Assessment of Mean Systemic Exposure Following Oral Administration of GSK1292263 in the Rat and Dog 13-week studies

Duration	Dose (mg/kg/day)	Sex	C_{max} (ng/mL)		AUC_{0-24} (ng.h/mL)	
			1st TK	End of Study	1st TK	End of Study
Rat (13 week)	10	M	488	395	4429	3702
		F	712	995	7002	10868
	150	M	1696	1081	22016	16285
		F	2390	2991	33178	42869
	2000	M	3394 (2899-3684)	2543 (2332-2755)	55601 (49322-63416)	43551 (30628-56474)
		F	6779 (5818-7372)	5749 (4977-6521)	92517 (83695-103185)	89976 (82895-97057)
Dog (13 week)	4	M	870	702	14034	17517
		F	1087	969	9541	12860
	20	M	2100 (1824-2467)	2573 (1885-3145)	32536 (28785-38282)	43504 (27838-59195)
		F	1716 (1402-1956)	2377 (2237-2478)	23153 (20103-25929)	36930 (32808-39672)
	1000	M	5091 (2801-6846)	5453 (2757-7304)	86890 (38952-124997)	107751 (48863-144523)
		F	5323 (4857-5816)	5743 (4628-6900)	94643 (81316-108038)	106938 (89998-124774)

1.5.2.4. Risk Assessment based on the Preliminary Observations from the 13-week Toxicology Studies in Rats and Dogs

There were no treatment-related effects in liver or testes in the rat and dog up to 2000mg/kg/day and 1000mg/kg/day GSK1292263, respectively, at systemic exposures of 58,405ng·hr/mL (male rats) and 49,505ng·hr/mL (male dogs) on Day 14. In addition, stage-dependent qualitative evaluation of spermatogenesis demonstrated normal progression up to the limit dose in both species indicating there were no subtle morphological changes in the testes following 14 days of dosing. In the 13-week rat study (10, 150, 2000mg/kg/day) there were no treatment-related findings in liver or testes up to 43,551ng·hr/mL (mean male rat AUC0-24hr).

For single dose administration of GSK1292263 in clinical subjects, preclinical data support mean clinical exposures up to 49,400ng·hr/mL (as described in the current Investigator Brochure, [GlaxoSmithKline Document Number RM2008/00434/00]).

Testis: In the ongoing clinical trials there are no means to readily monitor for the testicular effect observed in the 13-week dog study. The mean clinical AUC achieved at 300 mg BID (23,810ng.h/mL), the highest intended dose, is below the no-effect AUC for the testicular effect in any dog in the 3-month study (36,772ng.h/mL), and is less than 50% of the mean no-effect AUC in the dog 14-day study (49,505ng.h/mL).

The highest individual clinical AUC achieved at 300mg BID (38,771ng.h/mL) is well below the highest exposure achieved in an individual dog at the NOAEL following 14 days of dosing (78,414ng·hr/mL), and is also less than the lowest individual dog AUC for a testicular effect following 3 months of dosing (48,863ng.h/mL). Although 38,771ng.mL does exceed the highest individual no-effect exposure in the 3-month dog study (36,772ng.h/mL), the dog testicular change is regarded as a time-dependant effect and the intended clinical dose duration is 1/6 of the duration that caused testicular toxicity (14 versus 90 days) in a single species.

Liver: There are safety margins based on systemic exposure (AUC) for the liver effects observed in the 13-week dog study (4.5-fold at the effect dose of 1000mg/kg/day; 1.7-fold at the no-effect dose of 20mg/kg/day, compared to the clinical AUC at 300 mg BID) (Table 11). Importantly, liver effects are readily monitorable and liver function monitoring and withdrawal criteria are already in place in this study.

In summary, the doses of GSK1292263 being evaluated in Part C of this study (top BID dose limited to 300mg BID, or a dosing regimen resulting in equivalent exposure) do not pose a significant clinical risk from the perspective of either mean or individual exposures to date.

1.5.3. Capillary Blood Glucose (CBG) Monitoring

Subjects who are washed off prior anti-diabetic medications prior to dosing in Parts B and C must check fasting capillary glucose at least twice daily (fasting and prior to the evening meal) during the washout period, and at any time symptoms of hypoglycemia or hyperglycemia are experienced. A written diary card should be kept for each of these subjects. Subjects who are drug naïve are not required to measure CBG and maintain a diary card prior to dosing in Parts B and C.

Fasting capillary glucose values >270mg/dL or <70mg/dL must be reported to the site at once. If fasting glucose levels are >270mg/dL or <70mg/dL on any two consecutive days during the wash-out period, the subject will be discontinued and appropriate anti-diabetic therapy reinstated. If subjects do not already have a way to test blood glucose at home, a glucometer, instructions on its use, and testing strips will be provided to them.

While in-clinic, fasting glucose and pre-evening meal glucose will be monitored daily by CBG, venous blood or plasma glucose.

When subjects are in the unit they should alert study staff any time they experience symptoms that might be related to hypoglycaemia. Study staff should then test their CBG values. Hypoglycemia documented by a CBG ≤ 50mg/dL, or any hypoglycemia

requiring assistance should be assessed and a decision taken, in consultation with the GSK Medical Monitor, whether or not to withdraw the subject from the study.

Subjects are required to alert site staff while in the unit, or to call the study center while not in the unit:

- When they have CBG values that are >270mg/dL or < 70mg/dL
- When they have any concerns relating to their CBG levels
- When they have rapid, unexplained changes in their blood glucose levels
- When they have symptomatic hypoglycemia which is not confirmed by their blood glucose values

For the purpose of this protocol, 'hypoglycemia' as an AE is defined as any confirmed blood glucose value ≤50mg/dL, which may or may not be symptomatic. Episodes of hypoglycemia unconfirmed by blood glucose measurement may be recorded as an AE if the subject experienced symptoms consistent with their usual hypoglycemic episodes.

2. OBJECTIVE(S)

2.1. Primary

- To investigate in subjects with T2DM the safety and tolerability of GSK1292263 administered as single ascending (Part A) and repeat oral doses administered QD or BID (Part C).
- To determine the pharmacokinetic parameters of GSK1292263 in subjects with T2DM following single ascending (Part A) and repeat oral doses administered QD or BID (Part C).
- To evaluate in T2DM subjects the pharmacodynamic effects of GSK1292263 following single ascending doses (Part A) and repeated oral doses administered QD or BID (Part C), and the pharmacokinetic/pharmacodynamic relationships.

2.2. Secondary

- To investigate in subjects with T2DM the safety and tolerability of GSK1292263 when administered as a single dose with food (Part B).
- To determine the pharmacokinetic parameters of GSK1292263 in subjects with T2DM when administered as a single dose with food (Part B).
- To establish the pharmacodynamic effects of GSK1292263 in subjects with T2DM when administered as a single dose with food (Part B).
- To investigate the safety and tolerability of GSK1292263 co-administered with a single dose of sitagliptin (Part C).

- To determine the pharmacokinetic parameters of (i) GSK1292263 and sitagliptin when co-administered with a single dose of sitagliptin on Day 14, and (ii) sitagliptin alone (sitagliptin arm) on Day 14. (Part C)
- To evaluate the pharmacodynamic effects GSK1292263 when co-administered with a single dose of sitagliptin (Part C).
- To estimate the relative bioavailability of GSK1292263 and sitagliptin when administered together, compared to sitagliptin alone and GSK1292263 alone (Part C).

2.3. Exploratory

- To investigate the mechanism of action of GSK1292263.
- To compare the PD effects of GSK1292263 to those of sitagliptin.

3. ENDPOINT(S)

3.1. Primary

- Safety and tolerability parameters following single and repeat doses of GSK1292263 administered QD or BID, including adverse events, and assessments of clinical laboratory, ECGs and vital signs.
- Pharmacokinetic parameters following single and repeat doses of GSK1292263 administered QD or BID: Cmax, Tmax, t½, tlag, CL/F, V/F, AUC(0-τ), AUC(0-∞) for single dose and repeat doses, accumulation ratio (Ro), time invariance ratio (Rs), as data permit.
- Pharmacodynamic/biomarker endpoints will include fasting and meal or OGTT-related weighted mean AUC for glucose, insulin, glucagon, GLP-1 (active and total), C-peptide, total GIP, and total PYY, as well as insulin secretion and insulin sensitivity parameters, as data permit. Relationships between GSK1292263 drug exposures and pharmacodynamic parameters (e.g., glucose, insulin), safety (e.g., QTc), and tolerability will be evaluated, as appropriate.

3.2. Secondary

- Safety and tolerability parameters when GSK1292263 is administered as a single-dose with food (Part B), including adverse events, and assessments of clinical laboratory, ECGs and vital signs.
- Pharmacokinetic parameters when GSK1292263 is administered as a single-dose with food (Part B): Cmax, Tmax, t½, tlag, CL/F, V/F, AUC(0-τ), AUC(0-∞) for single dose, as data permit.
- Pharmacodynamic/biomarker endpoints will include fasting and meal or OGTT-related weighted mean AUC for glucose, insulin, glucagon, GLP-1 (active and total), C-peptide, total GIP, and total PYY, as well as insulin secretion and insulin sensitivity parameters, as data permit.

- Pharmacokinetic parameters: AUC(0-τ), AUC(0-24), AUC(0-12), Cmax, and tmax of GSK1292263 and sitagliptin when co-administered, and sitagliptin alone (sitagliptin arm) (Part C), as data permit.

3.3. Exploratory Analyses

- Pharmacodynamic/biomarker endpoints will include fasting and meal or OGTT-related weighted mean AUC for glucose, insulin, glucagon, GLP-1 (active and total), C-peptide, total GIP, and total PYY, as data permit.
- Correlation between drug dose or exposure and parameters of pharmacodynamics, safety, and tolerability.
- Pharmacodynamic/biomarker endpoints including measures of insulin sensitivity, beta cell function, GI peptide profiles and other exploratory lipidomic, peptidomic and metabolomic biomarkers, as data permit
- Exploratory analyses relating to biomarkers of T2DM or related metabolic diseases, biomarkers of GSK1292263, sitagliptin pharmacodynamics or safety may be performed using (i) small molecular weight metabolites, including, but not limited to, branch chain amino acids, acylcarnitines, and lipids, (ii) blood polypeptide analytes including, but not limited to, leptin, ghrelin, adiponectin, and (iii) novel biomarkers derived from peptidomic, lipidomic and metabolomic analysis of blood.

4. INVESTIGATIONAL PLAN

4.1. Discussion of Design

4.1.1. Part A: Crossover Study Comparing a Low, Mid and High Dose of GSK1292263, Placebo and Sitagliptin (Cohort 1)

Part A (Cohort 1) is a single-blind, randomized, placebo-controlled, 5-period crossover study in which drug naïve T2DM subjects will receive escalating doses of GSK1292263 in each of 3 periods and placebo and open-label sitagliptin in the other 2 periods. The sequence will be randomized, but will maintain the low, medium and high dose order for GSK1292263 (see Section 1.4.2). The dose of sitagliptin will be 100mg.

Modeling and simulation has determined that the optimal 3 doses to characterize the dose/exposure-response of GSK1292263 in Part A are 25, 150 and 800mg (fasted), but these doses may be changed based on emergent safety, tolerability and PD data. These doses will be administered to the subjects in an ascending sequence irrespective of the randomization order for placebo and sitagliptin. In other words, in any given sequence, subjects will always receive the low dose prior the medium dose, and the medium dose prior to the high dose, regardless of periods the sitagliptin and placebo occur in (see Table 12).

The subjects in Part A will be administered a 75g oral glucose tolerance test (OGTT) approximately 2h after dosing to assess the PD effects of GSK1292263, sitagliptin and placebo. On completion of the OGTT, a standardized lunch and evening meal will be provided as described by Mari, *et al.* [Mari, 2005].

Subjects will undergo screening procedures within 28 days of the first dosing period.

Subjects will be randomized upon admission to the research facility after successfully completing all screening and baseline assessments. Eligible drug-naïve T2DM subjects will be asked to comply with a dietary and exercise program starting 1 week prior to administration of study drug in Period 1 to ensure stability of their diabetic state during the 5 periods of the study.

Subjects will be admitted to the research facility within 24 hours of each dose period and will remain in unit for 24hr post dosing. The 24-hour time period when the subjects received placebo will be defined as the baseline, and the treatment periods will be defined as those in which the subjects received GSK1292263 or sitagliptin.

A follow-up visit will occur between 7 and 10 days after the last dose in Period 5.

The maximum amount of time a subject can expect to spend in this part of the study is approximately 10 weeks, including the 28-day period allotted for screening assessments.

A tablet formulation will be administered in all dose periods. Subjects will receive a single dose of GSK1292263 or placebo in the GSK1292263 and placebo periods, and a single dose of sitagliptin 100mg in the sitagliptin period. Based on the t½ of GSK1292263 observed in normal volunteers (~12 - 17hr) and sitagliptin (~12.4 hr), each period will be separated by approximately 6 days of washout.

For Part A, folate and iron supplementation may be started for 2 weeks after completion of Period 5 procedures, as necessary (see also Section 4.1.4).

4.1.2. Part B: GSK1292263 with Food (Cohort 2)

Part B (Cohort 2) is a single-blind, randomized, 2-period study in which T2DM subjects will receive a single dose of GSK1292263, fasted or fed. T2DM subjects on monotherapy or sub-maximal anti-diabetic medications will wash-off these medications for 1 week prior to study receiving study drug. Before subjects in Part B are dosed, the safety and tolerability of 800mg GSK1292263 administered in the fasted state to 9 subjects in Part A will be reviewed to ensure that it is safe to proceed.

In the FTIH study, GPR111596, food increased the exposure of GSK1292263 by up 2-to 3-fold in normal volunteers. Part B will comprise a two-period crossover of a single tablet dose of GSK1292263 administered in the fed or fasted state. The planned dose in Part B will be 800mg and will be administered after eating a high fat breakfast meal (see the SPM for specifics). This dose may be changed based on emergent safety, tolerability and PD data. Results from Part B will be used to confirm the ‘food effect’ seen in study GPR111596, and will allow assessment of the extent of the change in PK exposure in the

presence of food in T2DM patients. This will aid in the selection of doses in Part C, where the subjects will eat a standardised meal tolerance test (MTT) at breakfast.

Subjects in Part B may be drug-naïve or subjects who have washed off their usual anti-diabetic medications. Eligible T2DM subjects on monotherapy or sub-maximal anti-diabetic medications will wash off these medications for 1 week prior to receiving study drug.

Subjects will undergo screening procedures within 28 days of the first dose period, and will be randomized upon admission to the research facility after successfully completing all screening and baseline assessments.

Subjects will be admitted to the research facility within 24 hours of each dose period and will remain in unit for 24hr post-dosing.

The 24-hour time period when the subjects received GSK1292263 in the fasted state will be defined as the baseline, and the treatment period will be defined as that in which the subjects received GSK1292263 in the fed state. Each period will be separated by approximately 6 days of washout.

A follow-up visit will occur between 7 and 10 days after the last dose in Period 2.

The maximum amount of time a subject can expect to spend in this part of the study is approximately 7 weeks, including the 28-day period allotted for screening assessments.

4.1.3. Part C: Repeat Dosing (Cohorts 3 and 4)

Part C (Cohort 3 and Cohort 4) is a single-blind, randomized, placebo-controlled, 6-arm study of 14 days of dosing with GSK1292263, placebo or open-label sitagliptin. If Cohort 3 is dosed QD, Cohort 4 will be enrolled to investigate the safety, tolerability, pharmacokinetics and pharmacodynamics of GSK1292263 when dosed in a BID regimen. Alternatively, if Cohort 3 is dosed BID, then Cohort 4 will be enrolled to investigate the safety, tolerability, pharmacokinetics and pharmacodynamics of GSK1292263 when dosed in a QD regimen. Based on emergent data, there may also be an adjustment of the dose or dosing regimen in the GSK1292263 arms of Part C to allow more extensive evaluation of QD or BID doses.

If GSK1292263 is dosed QD in Cohort 3 of Part C, the planned dosed are 25, 150, 800mg QD. In the event of a dose adjustment, the maximum total daily dose to be administered will not exceed 600mg. Cohort 4 will evaluate half the maximal QD dose used in Cohort 3 administered twice a day for 14 days.

If Cohort 3 of Part C is dosed BID, then the planned doses will not exceed 300mg BID (total daily dose of 600mg), and Cohort 4 will evaluate the maximum total daily dose used in Part C administered QD for 14 days.

These doses may be changed based on emergent safety, tolerability, PK and PD data from Parts A, B and C. Ethics boards will be advised of the final doses chosen for Part C prior to the dosing of subjects. Safety, tolerability, PK and PD data from Parts A and B will be assessed prior to initiation of Part C.

Part C (Cohorts 3 and 4) will be conducted to assess safety, tolerability, PK and PD of GSK1292263 and open-label sitagliptin in T2DM subjects after 14-days of dosing, as well as the safety, tolerability, PK and PD of (i) a 100mg single dose of open-label sitagliptin co-administered with GSK1292263 or placebo (placebo arm), and (ii) sitagliptin alone (sitagliptin arm) on Day 14.

Subjects in Cohort 3 will be randomized into one of 5 treatment arms. Planned doses for Cohort 3 are as follows: 25mg dose of GSK1292263 once-daily, 150mg dose of GSK1292263 once-daily, 800mg dose of GSK1292263 once-daily, placebo or sitagliptin. Alternatively, GSK1292263 may be administered BID in 3 treatment arms, for comparison to placebo (administered BID) and open label sitagliptin administered QD.

Cohort 4 of Part C will be conducted to assess the PK and PD of BID or QD dosing of GSK1292263 in T2DM subjects after 14-days of dosing, as well as the safety, tolerability, PK and PD of a 100mg single dose of open-label sitagliptin co-administered with GSK1292263 on Day 14. If GSK1292263 is dosed QD in Cohort 3 of Part C, Cohort 4 will evaluate half the maximum QD dose used in Cohort 3 administered twice a day for 14 days. Alternatively, if Cohort 3 of Part C is dosed BID, then the planned doses will not exceed 300mg BID, and Cohort 4 will evaluate the maximum total daily dose used in Part C administered QD for 14 days.

Amendment No 4 Note: The actual doses being administered in Part C are: 50mg BID, 150mg BID, 300mg BID and 600mg QD.

Figure 3 Part C Schematic of Design

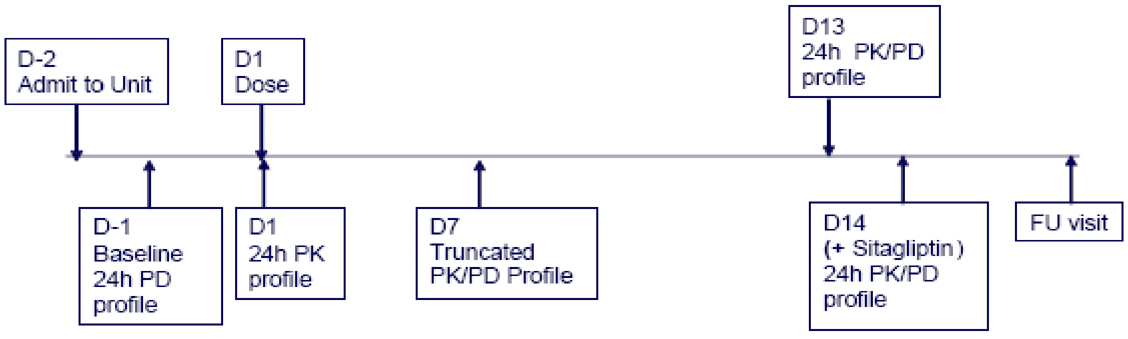

Subjects in Part C may be drug-naïve or subjects who have washed off their usual anti-diabetic medications. Eligible T2DM subjects on monotherapy or sub-maximal anti-diabetic medications will wash-off these medications for 1 week prior to receiving study drug.

Subjects from Parts A and B may also participate in Part C if, (i) they meet all inclusion/exclusion criteria, (ii) the length of washout of prior medications does not expose them to unacceptable deterioration of glycemic control, given that there will be an interval of 2-4 weeks between the completion of Parts A and B, and the start of Part C to allow for analysis of PK and PD data, and (iii) the Investigator considers the subject will not be compromised by the blood sampling in Part C.

Subjects will undergo screening procedures within 28 days of first dose. Subjects will be randomized upon admission to the research facility after successfully completing all screening and baseline assessments.

As shown in Figure 3, subjects will be admitted to the research facility within 48 hours (Day -2) of first dose (Day 1). The 24-hour time period prior to dosing (Day-1) will be defined as the baseline, and the treatment period will be defined as Days 1-14. On Day 14, subjects randomized to GSK1292263 or placebo (QD or BID) will also receive a single dose of 100mg sitagliptin. Full 24hr profiles for PK will be conducted on Days 1, 13 and 14, for GSK1292263. A full 24 hr PK profile will be conducted on Day 14 for sitagltipin. Full 24hr profiles for PD will be conducted on Days -1, 13 and 14. Truncated PK and PD profiles will be obtained on Day 7 to evaluate the time-course of the PD changes.

On Day 14, subjects will have the final PK and PD assessments, and will remain in unit for 24hr post dosing. Subjects may be discharged from the unit on the morning of Day 15, following all post-last dose safety, PK and PD assessments.

A follow-up visit will occur between 7 and 10 days after the last dose in each cohort.

The maximum amount of time a subject can expect to spend in this part of the study is approximately 7 weeks, including the 28-day period allotted for screening assessments.

Subjects will receive 14 consecutive-day doses of GSK1292263, placebo or sitagliptin. Subjects will be administered doses that are expected to be safe and well-tolerated based on data from study GPR11956 and Parts A and B of this protocol. Safety, tolerability, and 24-hour PK and PD profiles will be assessed on an ongoing basis following administration of study drug.

During the dosing period the subjects will eat standardized meals while in the unit. See the Study Procedure Manual (SPM) for specific instructions.

4.1.4. Screening

To determine subject eligibility for enrollment in the study, a Screening visit will be performed within 28 days of first dose administration. For the purposes of subject eligibility for enrollment in the study, screening assessments are defined as any assessments performed prior to the first dose of study drug, including baseline assessments that are used to qualify the subject for enrollment.

For Part A, subjects who are eligible for enrollment in the study after the Screening procedure will be counselled on dietary methods and may be prescribed folate and iron, if appropriate, from the Screening visit to 7 days before randomization to minimize the effects of blood draws on haemoglobin levels.

If a subject is not eligible for the study based on the Inclusion and Exclusion Criteria at the initial attempt, but may become eligible at a later date during the enrollment period, the investigator should contact the GSK Medical Monitor to discuss the possibility of re-screening the subject.

4.1.5. Washout (Parts B and C Only)

Subjects in Part A will be drug naïve and therefore will not require washout of anti-diabetic medications.

In order to evaluate the effect of GSK1292263 on plasma lipids, subjects who qualify for Parts B or C of the trial will begin washout of fibrate medications 14 days prior to Day 1. All subjects will return to the clinic 7 days prior to Day 1. If relevant, at this visit, subjects will discontinue use of concurrent medications such as metformin, sulfonylureas, DPP-IV inhibitors, exenatide and statins, fat absorption blocking agents, and bile acid sequestrants, and will remain off of these medications until discharged from the clinic after the final post-last-dose study assessments (end of Period 2 in Part B, and Day 15 in Part C). The investigator may use his/her discretion with respect to modifying the doses of these medications when they are restarted, and should review glucose results obtained during the in-house period to determine whether adjustments to oral anti-diabetic medication doses should be made.

As outlined in Section 1.5.3, subjects must check fasting capillary glucose at home during the washout phase prior to Parts B or C. A written log should be kept by each subject. The subject will be instructed on the use of a glucometer and procedures to follow if fasting capillary glucose values fall outside of the stipulated acceptable ranges:

- CBG: >270mg/dL or < 70mg/dL

4.1.6. Treatment regimen

4.1.6.1. Part A

Drug naïve T2DM subjects will enroll into Part A, check into the unit on Day -1 and be randomized to a treatment sequence including 3 dose levels of GSK1292263, matching placebo, or open-label sitagliptin.

4.1.6.2. Part B

After the appropriate washout period, subjects will enroll into Part B, check into the unit on Day -1 and be randomized to a treatment sequence involving a dose of GSK1292263 administered in the fed or fasted state.

4.1.6.3. Part C

Subjects will be randomized to 14 days of dosing with three dose levels of GSK1292263 (planned QD doses: 25, 150, 800mg; planned BID doses not to exceed 400mg BID) or matching placebo, or open-label sitagliptin 100mg. On Day 14, subjects randomized to GSK1292263 or placebo will also receive a single dose of 100mg sitagliptin. Subjects will check into the unit on Day -2, followed by 24h PK assessments on Days 1, 13 and 14, and PD assessments on Days -1, 13 and 14. Truncated PK and PD profiles will also be obtained on Day 7. PK samples will be collected for all subjects and will be analyzed for GSK1292263 concentrations on Days 1, 7 and 13. PK samples will be analyzed for both sitagliptin and GSK1292263 concentrations on Day 14. Sitagliptin PK will be measured only on Day 14 for subjects in the sitagliptin arm.

If GSK1292263 is dosed in a BID regimen in Part C (Cohort 3 or 4), on Day 14 sitagliptin will be dosed QD with the morning dose of GSK1292263.

Amendment No 4 Note: The actual doses being administered in Part C are: 50mg BID, 150mg BID, 300mg BID and 600mg QD.

After completion of Part C, the doses of prior anti-diabetic medications to be restarted may be modified as needed by the Investigator, who should review glucose results obtained during the 14-day dosing period to determine whether adjustments to these prior medication doses should be made.

4.1.7. Follow-up Visit

A follow-up visit will occur 7 to 10 days following discharge from the clinic. Any subject withdrawing from the trial prematurely should also be asked to complete follow-up procedures.

See Section 4.5 and Section 4.6 for details regarding all study procedures performed during the course of this trial.

4.1.8. Pharmacodynamic Testing

4.1.8.1. Oral Glucose Tolerance Test (OGTT) – Part A Only

In Part A, an OGTT will be used to assess the PD effects of GSK1292263.

After the ingestion of the 75g oral glucose challenge, there is a rapid rise in insulin secretion (first phase response), which is followed by a more sustained release of the hormone (second phase). During this secretion process, C-peptide, or connecting peptide, is split from pro-insulin, the insulin precursor molecule, and is produced in equimolar amounts to insulin. In the bloodstream, C-peptide has a long half-life, because, unlike insulin, it is not subject to hepatic clearance. Blood samples will be collected for 180min so that the first and second phases of insulin secretion can be derived by modelling the C-peptide and insulin kinetics data during the OGTT. In addition, insulin sensitivity can be calculated from the rate of appearance and disappearance of the ingested glucose.

4.1.8.2. Meal Tolerance test (MTT) – Part C only

On Days -1, 7, 13 and 14 in Part C, subjects will be administered a standardized MTT at breakfast time. Blood samples will be collected up to 180min so that the first and second phases of insulin secretion can be derived by modelling the C-peptide and insulin kinetics data during the MTT. In addition, insulin sensitivity may be calculated from the rate of appearance and disappearance of the ingested glucose in the meal. Limited PD and PK sampling will be conducted on Day 7.

The composition of the meal for the MTT and timing relative to dosing are defined in the SPM.

On Day 1 (full PK sampling day), all meals will be of the same composition as those on the PD profiling days, but no PD samples will be taken.

On Days -1, 1, 7, 13 and 14 the subjects must eat all of the breakfast meal provided.

4.1.8.3. Hunger, Satiety and Caloric Intake (Part C)

A putative natural ligand for GPR119, oleoylethanolamide, is an endocannabinoid that reduces food intake in animal models. In addition, the elevations of incretins and PYY seen in the human may also affect feeding. For this reason, this study includes assessments of eating behaviors and calorie intake.

The modified Hunger, Craving and Fullness Questionnaire (HCFQ) (see Appendix 3: Hunger, Craving, and Fullness Questionnaire) is a 7-item questionnaire, designed to assess changes in eating behaviors as a result of study treatment. The original HCFQ instrument was developed by GSK in collaboration with Mapi Values and underwent psychometric evaluation by Oxford Outcomes. The modified HCFQ questionnaire is a self-administered questionnaire that will be used as an exploratory measure to understand patient-reported feelings of hunger and satiety.

The modified HCFQ is comprised of 7 items to assess hunger, craving, and fullness. Patients rate each item on a Likert scale comprising 5 responses. The modified instrument has not undergone psychometric evaluation.

The modified HCFQ will be administered after dinner on Days -1, 7, 13 and 14, (see Appendix 3 and SPM for details). Any subject who withdraws from the study should complete the modified HCFQ at withdrawal.

If feasible, on Days -1, 7, 13 and 14, calorie counts will be obtained across the meal periods and during the overall 24h (note that the subjects must eat the whole breakfast meal on these days).

4.1.8.4. Other Pharmacodynamic Profiling

The oral ingestion of a meal stimulates the release of incretins, such as GLP-1 and GIP, and other gut hormones, including PYY.

Assessment of 24-hour glucose, insulin, C-peptide, glucagon, GLP-1 (total and active), GIP, and PYY (total) may be conducted in Parts A and B to determine the effects in the prandial and post-prandial periods.

In Part C, assessment of 24-hour glucose, insulin, C-peptide, glucagon, GLP-1 (total and active), GIP (total), PYY (total) at baseline (Day -1), and on Days 7 and 13 will allow evaluation of the pharmacodynamic effect of repeat-dosing of GSK1292263 versus placebo and sitagliptin, and will permit comparison of the three dose levels (low, medium, high) of GSK1292263. In addition, the effect of adding sitagliptin to GSK1292263 will be assessed primarily by comparing the responses on Day 13 and Day 14, but further information may be forthcoming by comparing Day -1 and Day 14. Other biomarkers, such as glycerol, may be assessed at the same timepoint.

4.1.8.5. Exploratory Biomarkers

Blood sample(s) will be collected in Part C for metabolomic, lipidomic and proteomic analyses, as blood volume limits permit, to investigate how fingerprints of these analytes change during treatment and whether they provide insights into the biochemical mechanisms involved. As new data emerge, it may also be possible to probe novel aspects of the biology of T2DM or related metabolic disorders such as obesity and the metabolic syndrome, as well as the biological and clinical responses to GSK1292263. If relevant, this approach will be extended to include the identification of biomarkers associated with adverse events.

The biomarker research activities may include measurement of, (i) known biomarkers that have been associated with T2DM and/or related metabolic disorders, and (ii) novel biomarker discovery activities related toT2DM, and/or related metabolic diseases.

Biomarkers may include, but are not limited to, (i) small molecular weight metabolites, including branch chain amino acids, acylcarnitines, and lipids, (ii) blood polypeptide analytes including leptin, ghrelin, adiponectin, and (iii) novel biomarkers derived from peptidomic, lipidomic and metabolomic analysis of blood.

Performance of these biomarker investigations may be conditional on the results of the clinical trial and samples may be selected for analysis on the basis of the clinical outcome. For example, samples may be selected for analysis based on the response to intervention. Because of their exploratory nature, these investigations may be performed irrespective of whether a pharmacodynamic response is observed.

In Part C, samples will be collected on Days -1, 7, 13 and 14. Details for sample collection and processing of samples can be found in the SPM. The timing of the collections may be adjusted on the basis of emerging PK or PD data from this study or other new information in order to ensure optimal evaluation of the PD.

Samples of blood may be stored for a maximum of 15 years after the last subject completes the trial for possible future assessments of additional biomarkers.

4.2. Treatment Assignment

Subjects will be assigned to study treatments in accordance with the randomization schedule generated by Discovery Biometrics, prior to the start of the study, using validated internal software. A separate randomization schedule will be used for each part of the study.

For each Part of the study, subjects will be identified at enrollment by a unique subject number that will remain consistent for the duration of that part of the study. This number will be used for subject identification throughout that part of the study. Each site will be provided a unique set of numbers in chronological order beginning with the lowest number. Once a subject number is assigned to a subject, it cannot be re-assigned to any other subject during the study.

For Parts A and B, subjects will be randomized according to a randomization schedule provided to each site. This will permit appropriate dosing by the site pharmacist, and will be recorded on the eCRF.

A web-based central randomization method will be employed in Part C. Upon confirmation of eligibility, investigators or designated staff will use the IWRS to randomize a subject. Once a randomization number is assigned to a subject, it cannot be re-assigned to any other subject during the study.

Subjects will be randomized to receive active treatment or placebo in Parts A and C, and GSK1292263 in the fed or fasted state in Part B. If a subject is prematurely discontinued from the study and a replacement subject is to be recruited, a replacement treatment number will be used to assign treatment to the replacement subject.

An example treatment regimen (possible sequences) for Part A is provided in Table 12:

Table 12 Example Treatment Regimen (Part A)

Subjects	Period 1	Period 2	Period 3	Period 4	Period 5
n=2	GSK Lo	GSK Mid	GSK Hi	Placebo	Sitagliptin
n=2	GSK Lo	GSK Mid	GSK Hi	Sitagliptin	Placebo
n=2	Placebo	GSK Lo	GSK Mid	GSK Hi	Sitagliptin
n=2	Sitagliptin	GSK Lo	GSK Mid	GSK Hi	Placebo
n=2	Placebo	Sitagliptin	GSK Lo	GSK Mid	GSK Hi
n=2	Sitagliptin	Placebo	GSK Lo	GSK Mid	GSK Hi

GSK Lo=GSK1292263 Low dose, GSK Mid=GSK1292263 Medium dose, GSK Hi=GSK1292263 High dose, Sitagliptin=sitagliptin 100mg.

An example treatment regimen for Part B is provided in Table 13:

Table 13 Example Treatment Regimen (Part B)

Subjects	Period 1	Period 2
GSK1292263<br>n=2	Fed	Fasted
GSK1292263<br>n=2	Fasted	Fed

In Part C, subjects will be randomized to receive an active treatment in Cohort 3 (3 dose levels of GSK1292263 or sitagliptin 100mg) or placebo or GSK1292263 in a QD or BID regimen in Cohort 4 in a ratio of 1:1:1:1:1:1. Appropriate adjustment will be made to prevent an enrollment bias based on prior anti-diabetic treatment.

4.3. Investigational Product Dosage/Administration

Product name:	Investigational Product	
	GSK1292263	Placebo
Formulation description:	Immediate Release Tablet, Round, White, Film Coated	Immediate Release Tablet, Round, White, Film Coated
Dosage form:	Tablet	Tablet
Unit dose strength(s)/Dosage level(s):	25mg, 75mg and 200mg	Placebo
Route/Administration/Duration:	Oral	Oral
Dosing instructions:	Dose orally with ~250mL water per protocol	Dose orally with ~250mL water per protocol
Physical description:	Round, white, plain faced, film coated tablet	Round, white, plain faced, film coated tablet
Device:	None	None
Manufacturer/source of procurement:	GSK	GSK

Sitagliptin will be administered as open-label Januvia.

4.4. Dose Adjustment/Stopping Criteria

This protocol allows some alteration from the currently outlined dosing schedule. No dose will be administered that is projected to exceed: (i) for the cohort as a whole, a mean plasma AUC(0-24) 49,400ng.h/mL and mean Cmax of 2693ng/mL, which is 80% of the gender-averaged NOAEL mean exposure in beagle dogs at Day 14 at the highest dose of 1000mg/kg/day, or (ii) for an individual subject an AUC of 72,700ng.h/mL and Cmax of 3585ng/mL, which are 80% of the highest AUC and Cmax observed in dog at 1000mg/kg NOAEL dose group in the 14 day toxicology study. These exposures limits were approved for the conduct of the FTIH study, GPR111596, by the GSK Global Safety Board and the local IRB, and were not objected to by the FDA.

In Amendment No. 4, however, the maximum total daily dose that will be evaluated in Part C of this study is 600mg based on preliminary data from the 13-week toxicology study in the dog (see Section 1.5.2).

In Part A, the planned doses of GSK1292263 will be 25, 150 and 800mg. Doses up to and including 400mg in the fasted and fed states have been evaluated in healthy normal subjects in the FTIH study GPR111596, and found to be safe and well tolerated. Safety and tolerability will be assessed during each dosing period and the planned medium and/or high doses of GSK1292263 may be altered based on emergent safety and tolerability data obtained for the lower dose(s).

For Part B, a planned dose of 800mg GSK1292263 may be adjusted based on emergent safety and tolerability data from Part A.

For Part C, subjects in Cohort 3 will be randomized to receive planned doses of 25mg GSK1292263 QD, 150mg QD and 800mg QD. Alternatively, GSK1292263 may be administered BID in 3 treatment arms at doses that do not exceed 300mg BID, for comparison to placebo (administered BID) and open label sitagliptin administered QD. The actual doses to be administered in Cohort 3 may be modified based on preliminary PK/PD, safety and tolerability data from Parts A and B. Final doses will be selected which are predicted to maintain exposures below nonclinical toxicology limits.

Cohort 4 of Part C will be conducted to assess the PK and PD of BID or QD dosing of GSK1292263 in T2DM subjects after 14-days of dosing, as well as the safety, tolerability, PK and PD of a 100mg single dose of open-label sitagliptin co-administered with GSK1292263 on Day 14. If GSK1292263 is dosed QD in Cohort 3 of Part C, Cohort 4 will evaluate half the maximum QD dose used in Cohort 3 administered twice a day for 14 days. Alternatively, if Cohort 3 of Part C is dosed BID, then Cohort 4 will evaluate the maximum total daily dose used in Part C administered QD for 14 days.

Doses in Part C (Cohorts 3 and 4) will be confirmed or changed based on safety/tolerability data (including clinically significant abnormalities of clinical labs, vital signs, and ECGs), and PK and PD data from Parts A, B and C.

Amendment No 4 Note: The actual doses being administered in Part C are: 50mg BID, 150mg BID, 300mg BID and 600mg QD.

The actual doses to be administered or already being administered in Part C may be adjusted based on safety and tolerability and emergent preliminary pharmacokinetic and/or pharmacodynamic data. These dose adjustments may involve either an increase or a decrease in the planned dose, but all doses will be selected so as not to exceed 600mg total daily dose of GSK1292263 and/or the mean or individual plasma exposures defined by the non-clinical toxicology studies.

Study enrollment may be adjusted to include more subjects in a dosing cohort to further evaluate safety, pharmacokinetic and/or pharmacodynamic findings at a given dose level, or to add cohorts to evaluate up to 2 additional dose levels. The study procedures for these additional subject(s) or cohort(s) will be the same as those described for the other study subjects.

In all cases, ethics boards will be informed of the actual doses to be investigated prior to administration to the subjects.

4.4.1. Liver Chemistry Stopping Criteria

Liver chemistry threshold stopping criteria have been designed to assure subject safety and to evaluate liver event etiology during administration of investigational product and the follow-up period. Investigational product will be stopped if any of the following liver chemistry stopping criteria is met:

1. ALT ≥ 3xULN and bilirubin ≥ 2xULN
   NOTE: serum bilirubin fractionation should be performed if testing is available. If fractionation is unavailable, urinary bilirubin is to be measured via dipstick (a measurement of direct bilirubin, which would suggest liver injury).

2. ALT ≥ 5xULN.

3. ALT ≥ 3xULN if associated with the appearance or worsening of rash or hepatitis symptoms (fatigue, nausea, vomiting, right upper quadrant pain or tenderness, fever, rash or eosinophilia).

Subjects with ALT ≥ 3xULN **and** < 5xULN **and** bilirubin < 2xULN, who do not exhibit hepatitis symptoms or rash, can continue investigational product as long as they can be monitored weekly for 4 weeks. See Section 13 for details on weekly follow-up procedures for these subjects.

Refer to Section 13, Liver Chemistry Follow-up Procedures, for details of the required assessments if a subject meets any of the above criteria.

4.4.2. QTc Withdrawal Criteria

A subject that meets the criteria below will be withdrawn from the study:

- QTcB or QTcF > 500 msec or uncorrected QT >600msec (machine or manual over-read)
- If subject has partial right bundle branch block then criteria is QTcB or QTcF > 530msec. Subjects with left bundle branch block are not eligible for the study

These criteria are based on an average QTc value of triplicate ECGs. If an ECG demonstrates a prolonged QT interval, obtain 2 more ECGs over a brief period, and then use the averaged QTc values of the 3 ECGs to determine whether the subject should be discontinued from the study.

4.4.3. Blood Glucose Withdrawal Criteria

- If a subject experiences symptomatic hypoglycemia during the course of treatment, the relevant data should be evaluated closely and discussed with the medical monitor. A hypoglycemic episode is defined as symptoms consistent with hypoglycemia which are confirmed by glucometer result or plasma glucose sample <50mg/dL.
- If fasting plasma glucose >280mg/dL, confirmed by prompt repeat testing.

4.4.4. Additional Withdrawal Criteria

- PR interval <100 or >220msec.
- QRS duration <55 or >120msec.
- Systolic blood pressure changes by more than 30mmHg and/or diastolic blood pressure changes by more than 20mmHg from pre-dose baseline on 3 occasions 5min apart. In these circumstances, a subject may be withdrawn, in consultation with the GSK Medical Monitor, if blood pressure remains elevated 6 hours later.
- In Parts A and B, subjects who present pre-dose (D-1) with a hemoglobin < 10 g/dL will be withdrawn from the study.

Any subject with clinically significant changes in safety parameters not listed above or significant AEs thought to be drug related will be withdrawn and monitored until recovery. A full assessment by the Investigator and the GSK Medical Monitor will determine whether it is safe and ethical to continue dosing the remaining subjects.

4.5. Time and Events Table (Parts A and B)

Procedure	Screening ≤28 days prior to first dose	Washout Days -14 to Day -2	Periods 1 through 5 (Part A) Periods 1 and 2 (Part B)			Follow-Up
			Day -1	Day 1	Day 2	7 -10 days after final discharge
Clinic Visit[1]	X		------------------------In-Clinic------------------------			X
Informed Consent	X					
Inclusion/Exclusion Criteria	X					
Demographics	X					
Medical History / Current Medical Conditions	X					
Complete Physical Exam	X					X
Weight	X			X		X
BMI Calculation	X					
Waist Circumference	X		X			
Urine Drug/Cotinine Screen	X		X			
Urine/ Breath Alcohol Screen	X		X			
Vitals [2]	X		X	X		X
24-hour Holter	X			X		
12-lead ECG[3]	X			X		X
Telemetry[4]				X		
Hematology/ Clinical Chemistry[5]	X		X		X	X
Urinalysis	X		X		X	X
Urine Pregnancy Test			X			
FSH / Estradiol[6]	X					
HIV / Hep-B/ Hep-C Serology	X					
PGx				X		
Study Medication Administration [7]				X		
Glucose and Insulin Sampling[8]				X		
Additional Biomarker Samples[9]				X		
Washout / Glucometer[10]		X				
OGTT				X		
Standardized meals				X		
PK Blood Sampling[11]				X	X	
Glucometer readings[12]				X		

Procedure	Screening ≤28 days prior to first dose	Washout Days -14 to Day -2	Periods 1 through 5 (Part A) Periods 1 and 2 (Part B)			Follow-Up
			Day -1	Day 1	Day 2	7 -10 days after final discharge
Con Med Assessment	X		X	X	X	X
Adverse Event Assessment			X	X	X	X

1. Screening visit must be performed within 28 days of Day 1.
2. Assessment of vital signs (including blood pressure and heart rate) will be performed at one time point at Screening, at follow-up and on Day -1 of each period. On Day 1, they will be taken at pre-dose, 1 hour, 3, 4, 6, 10, 16 and 24hours. Assessments should be made in triplicate at each pre-dose time point, and single assessments should be made at all other times. Assessments should be performed after resting in a supine or semi-supine position for at least 10 minutes.
3. ECGs will be taken at Screening, on Day 1 at pre-dose, 1 hour, 2, 3, 4, 6, 10, 16, 24hours and at follow-up. Assessments should be made in triplicate at each pre-dose time point, and single assessments should be made at all other times. ECGs should be taken while subject is supine. Additional ECGs may be taken at the discretion of the investigator as needed based on symptoms or telemetry findings.
4. Telemetry will be performed for 24 hours on Day 1 of each period, starting 1hr prior to dosing.
5. Blood samples for safety will be collected at screening, pre-dose (Day -1), 24hr post- dose for each period, and at follow-up. When this results in multiple samples at the same time point, only one sample will be collected (e.g., 24hrs post-first-dose = pre-dose (time 0) for the second dose. Refer to Section 7.2.4 for specific laboratory parameters to be tested.
6. FSH and Estradiol tests will be performed for all post-menopausal women.
7. In Part A study drug will be administered 2h before the OGTT. In Part B study drug will be administered 30 min after breakfast and lunch and dinner will be approximately 4 and 10 hours after dosing
8. Blood samples for the determination of glucose and insulin will be collected at pre-dose on Day 1 of each dosing period,, and immediately prior to and at 10, 20, 30, 60, 90, 120, 180min after administration of the 75g glucose drink in Part A. For breakfast, lunch and evening meal in Part B and lunch and evening meal in Part A, samples will be collected just before (Part A)/after (Part B) the meal and at the following times after starting each meal: 0.5, 1, 1.5 (except breakfast in Part B), 2 and 3 hours. Samples will also be collected in Part B at 24hrs post-dose. When this results in multiple samples at the same time point, only one sample will be collected (e.g., 24hrs post-first-dose = pre-dose (time 0) for the second dose. Refer to the Study Procedures Manual for additional details on the collection of PD samples in relation to meals, and processing of PD samples.
9. If blood volume permits, a blood sample for additional biomarkers will be collected pre-dose on Day 1 of each dosing period,, and immediately prior to and at 30, 60, 90 and 120 after administration of the 75g glucose drink in Part A. For breakfast, lunch and evening meal in Part B and lunch and evening meal in Part A, samples will be collected just before (Part A)/after (Part B) the meal and at the following times after starting each meal: 0.5, 1, 1.5 (except breakfast in Part B) and 2 hours. Samples will also be collected in Part B at 24hrs post-dose. When this results in multiple samples at the same time point, only one sample will be collected (e.g., 24hrs post-first-dose = pre-dose (time 0) for the second dose. Refer to the Study Procedures Manual for additional details on the collection of samples in relation to meals, and processing of these samples.
10. Subjects washing out of fibrates should be instructed to do so beginning on Day -14, but no visit occurs at that time.
11. Blood samples for the determination of PK will be collected at the following times (PK sample times may be changed based on observed PK profile, but the total number of samples will not change) on Day 1 of each period: Immediately pre-dose (time 0) and at 0.5, 1, 2, 3, 4, 6, 8, 14 (for Part B, sample should be collected at 13hrs, not 14hrs), and 24 hours (when this results in multiple samples at the same time point, only one sample will be collected (e.g., 24hrs post-first-dose = pre-dose (time 0) for the second dose). Refer to the Study Procedures Manual for details on the collection of PK samples in relation to meals, and processing of PK samples.
12. Subjects should be checked by investigational staff during the day and night to ensure there are no findings consistent with hypoglycemia (e.g., cool, moist skin, and diaphoresis)

4.6. Time and Events Table (Part C)

Procedure	Screening ≤28 days prior to first dose	Period 1 Days -14 to Day -3	Day -2	Day -1	Day 1	Days 2-6	Day 7	Days 8-12	Day 13	Day 14	Day 15	Follow-up 7 -10 days after discharge
Clinic Visit	X	Washout[5]	---In Clinic---									X
Informed Consent	X											
Inclusion/ Exclusion Criteria	X											
Demographics	X											
Complete physical	X											
Check into Clinic			X									
Brief physical			X									X
Medical/medication/ drug /alcohol history	X											
Weight	X				X					X		
Waist Circumference					X					X		
Height / BMI Calculation	X											
12-lead ECG[1]	X			X	X		X		X	X	X	X
24-hr Holter	X											
Vitals[2]	X			X	X	X	X	X	X	X	X	X
Urine drug/alcohol screen	X		X									
Urine pregnancy test			X									X
Clinical Chemistry / Hematology / Urinalysis[3]	X		X		X		X			X	X	X
FSH/ Estradiol[4]	X											
HIV/ Hep B / Hep C	X											
Washout / Glucometer		X	X	X								
PGx[6]					X							
Dosing – Randomized study med[7]					X	X	X	X	X	X		
Mixed Meal Tolerance Test [8]				X			X		X	X		
Modified HCFQ [9]				X			X		X	X		
Calorie counts				X			X		X	X		
Standardized meals				X	X	X	X	X	X	X		
Blood samples for glucose and insulin/PD[10]				X			X		X	X	X	
PK profile blood samples[11]					X		X		X	X	X	
PK Trough Samples[11]						X	X					
Resume Washed-out Meds[12]											X	
Checkout from clinic											X	

Procedure	Screening	Period 1										Follow-up
	≤28 days prior to first dose	Days -14 to Day -3	Day -2	Day -1	Day 1	Days 2-6	Day 7	Days 8-12	Day 13	Day 14	Day 15	7 -10 days after discharge
Concomitant Medication Review[13]	X	X	X	X	X	X	X	X	X	X	X	X
AE assessment			X	X	X	X	X	X	X	X	X	X

1. ECGs will be taken at Screening, and on Day -1,1,7,13 and 14 pre-breakfast dose (fasting) and at 1,3, 6, 9,12 and 24hour and at Follow-up. Assessments should be made in triplicate at each pre-dose time point and single assessments should be made at all other times.ECGs should be taken while subject is supine. Additional ECGs may be taken at the discretion of the investigator as needed based on symptoms or ECG findings.
2. Assessment of vital signs (including blood pressure and heart rate) will be performed at Screening, on Days -1 to 14 in a fasting state early in the morning (prior to morning dosing on days 1-14), and at Follow-up. On Days 1,7,13 and 14, they will also be taken at 1,3, 6, 9,12 and 24 hours after the morning dose. At each time point, assessment should be performed after resting in a supine or semi-supine position for at least 10 minutes.
3. Blood samples for safety will be collected at screening, on Day -2 (can be non-fasting), and prior to breakfast (early in the morning, fasting) on Days 1, 7, and 14, and on Day 15 prior to checkout, (=24hrs post-dose), and at follow-up. When this results in multiple samples at the same time point, only one sample will be collected (eg.when 24hrs post-first-dose = pre-dose (time 0) for the second dose). Refer to Section 7.2.4 for specific laboratory parameters to be tested.
4. FSH and Estradiol tests will be performed for all post-menopausal women.
5. Subjects washing out of fibrates should be instructed to do so beginning on Day -14, but no visit occurs at that time.
6. PGx sample may be taken at any time after the first dose.
7. For QD dosing, study drug will be administered immediately after breakfast. For BID dosing study drug will be administered immediately after breakfast and immediately after the evening meal, but prior to the 10hr PK sample (see Study Procedures Manual for details of sampling times relative to dosing times),
8. Composition of the meal tolerance test is specified in the SPM.
9. Modified HCFQ is administered after dinner, at the same time of day, ±1 hour, on Days -1,7, 13 and 14.
10. Blood samples for the determination of glucose and insulin and other PD markers will be collected fasting pre-breakfast and then pre-morning dose (PD time 0) on Days -1,13 and 14, and then at 10,20,30,60, 90,120,180min after eating the standardised breakfast meal tolerance test. For lunch (approximately 4h post morning dose) samples will be collected just before the meal and at the following times after starting each meal: 0.5,1,1.5,2 and 3 hours. For the evening meal (approximately 10h post morning dose), BID dosing groups should follow the sequence of sampling, food and dosing as for breakfast (PD sample immediately before meal, eat and then dose), then 0.5,1,1.5,2 and 3 hours post dinner. A sample will also be collected 24 hours post-dose. On Day 7,samples for glucose and insulin and other PD markers will be collected fasting, pre-breakfast and at 1,2,4 (= pre lunch), 6,10 and 12h post morning dose, if blood volume permits. When this results in multiple samples at the same time point, only one sample will be collected (eg.,24hrs post-first-dose = pre-dose (time 0) for the second dose). Refer to the Study Procedures Manual for additional details on the collection and processing of PD samples.
11. Serial blood samples for the determination of PK will be collected on Days 1,7,13 and 14. PK sampling times may be changed based on observed PK profile, but the total number of samples will not change. When GSK1292263 is dosed QD, blood samples for PK will be collected on Days 1,13, and 14 immediately pre-dose (time 0) and at 0.5, 1, 1.5, 2, 3, 4,6,8,14 and 24 hours post-dose. When GSK1292263 is dosed BID, blood samples for PK will be collected on Days 1,13 and 14, at immediately pre-morning dose, 1,2, 4, 6, 8, 10,11,12,14,16,18,and 24hrs post-morning dose. For QD and BID dosing regimens on Day 7, blood samples for PK will be collected at predose (=post- breakfast), 1, 2, 4 (=pre lunch), 6,10 (=immediately post-dinner) and 12h. For QD and BID dosing regimens, trough samples for PK will be collected early in the morning (fasting) on Days 4, 5,and 6. When planned PK sampling results in multiple samples at the same time point, only one sample will be collected (e.g., 24hrs post-first-dose = pre-dose (time 0) for the second dose). Refer to the Study Procedures Manual for details on the collection and processing of PK samples.

Time and Events Table (Part C) (Continued)

12. If appropriate, the PI has the option of adjusting doses of washed-out medications when they are resumed. Review of glucose results should occur, and doses of oral anti-diabetic medications adjusted based on pharmacologic effects of treatment observed during the study.
13. At screening, medications taken in the 90 days prior should be collected.

5. STUDY POPULATION

5.1. Number of Subjects

Approximately 100 subjects will be enrolled (12 in Part A ([Cohort 1], 4 in Part B [Cohort 2], and 84 in Part C [72 in Cohort 3 and 12 in Cohort 4]) and complete dosing and critical assessments. Additional subjects/cohorts may be enrolled to allow for evaluation of additional dose levels.

For Cohort 3 of Part C, 12 subjects will be randomized into each of 5 treatment arms (planned QD doses 25, 150, 800mg GSK1292263 or planned BID doses not to exceed 400mg BID, placebo or open-label sitagliptin).

Amendment No 4 Note: The actual doses being administered in Part C are: 50mg BID, 150mg BID, 300mg BID and 600mg QD.

If safety, PK and PD data are satisfactory, an additional 4 subjects will be randomized into each of the three GSK1292263 treatment arms. Twelve subjects will be randomized into Cohort 4 (QD or BID regimen depending on the dosing regimen evaluated in Cohort 3). This will result in a total of 16 subjects in each of the GSK1292263 treatment arms in Cohort 3, 12 in the placebo arm, 12 in the sitagliptin alone arm, and 12 in the GSK1292263 treatment arm in Cohort 4.

If subjects prematurely discontinue the study, additional subjects may be enrolled as replacement subjects and assigned to the same treatment sequence (Parts A and B), or treatment arm (Part C), at the discretion of the Sponsor, in consultation with the Investigator.

5.2. Eligibility Criteria

5.2.1. Inclusion Criteria

A subject will be eligible for inclusion in this study only if all of the following criteria apply:

1. Male or female subjects, 18 - 60 years of age, inclusive, at the time of signing the informed consent.
2. A female subject is eligible to participate if she is of non-childbearing potential, defined as pre-menopausal females with a documented tubal ligation or hysterectomy; or postmenopausal defined as 12 months of spontaneous amenorrhea. If the clinical situation is unclear, FSH and estradiol levels may be used to confirm post-menopausal status at Screening. Simultaneous follicle stimulating hormone (FSH) > 40 mIU/ml and estradiol < 40pg/ml (<140pmol/L) is confirmatory in the absence of a clear post-menopausal history.

3. Except as noted elsewhere, subjects should have no significant known medical conditions other than T2DM, as determined by a responsible physician, based on a medical evaluation including medical history, physical examination, laboratory tests and ECGs. A subject with a clinical abnormality or laboratory parameters that meets exclusion criteria but is outside the reference range for the population being studied may be included only if the Investigator and the GSK Medical Monitor agree that the finding is unlikely to introduce additional risk factors and will not interfere with the study procedures.
4. BMI (body mass index) within the range 21.8-35.2 kg/m2, inclusive.
5. Part A: T2DM diagnosed by American Diabetes Association criteria prior to Screening:
   - Currently controlled by diet and exercise, and no anti-hyperglycemic medications used in the past 3 months.
   - Fasting plasma glucose (FPG) level ≤ 250mg/dL at the Screening visit.
   - FPG level ≤ 250mg/dL on Day -1.
   - HbA1c between 6.5 and 11%, inclusive, at Screening visit.
6. For Part B: T2DM diagnosed by American Diabetes Association criteria for at least 3 month prior to Screening:
   - Controlled by diet and exercise, or, if on medication, subjects must be treating their T2DM using one of the following regimens:
     - ❖ Metformin as monotherapy
     - ❖ Sulfonylurea as monotherapy
     - ❖ Metformin and sulfonylurea in combination, if both components are being administered at doses that are half their maximum dose or less
     - ❖ DPP-IV inhibitors, either as monotherapy or in combination with other agent(s) on this list at half maximal dose or less
     - ❖ Exenatide, either as monotherapy or in combination with other agent(s) on this list at half maximal dose or less

   For subjects that are being screened for Part B, all doses of anti-diabetic medication must have been stable for at least 3 months prior to Screening, and the subject must be willing to wash out from their anti-diabetic medications from Day -7 through post-last-dose of Period 2.

   - Fasting plasma glucose (FPG) level ≤ 220mg/dL at the Screening visit
   - FPG level ≤ 250mg/dL on Day -1
   - HbA1c between 7 and 11%, inclusive, at Screening visit
7. For Part C: T2DM diagnosed by American Diabetes Association criteria for at least 3 month prior to Screening:

- Controlled by diet and exercise, or, if on medication, subjects must be treating their T2DM using one of the following regimens:
  - ❖ Metformin as monotherapy
  - ❖ Sulfonylurea as monotherapy
  - ❖ DPP-IV inhibitors as monotherapy
  - ❖ Exenatide as monotherapy

Note: For subjects that are being screened for Part C, all doses of anti-diabetic medication must have been stable for at least 1 month prior to Screening, and the subject must be willing to wash out from their anti-diabetic medications from Day -7 through Day 15.

- Fasting plasma glucose (FPG) level ≤ 220mg/dL at the Screening visit
- FPG or fasting blood glucose level ≤ 250mg/dL on Day -2 or Day -1
- HbA1c between 7 and 11%, inclusive, at Screening visit

8. Capable of giving written informed consent, which includes compliance with the requirements and restrictions listed in the consent form.

5.2.2. Exclusion Criteria

A subject will not be eligible for inclusion in this study if any of the following criteria apply:

1. Has any of the following laboratory abnormalities:
   - Positive pre-study Hepatitis B surface antigen or positive Hepatitis C, result within 3 months of screening.
   - Positive test for HIV antibody.
   - History of uncorrected thyroid dysfunction or an abnormal thyroid function test assessed by TSH at Screening. (NOTE: subjects with hypothyroidism on a stable dose of thyroid replacement therapy for at least 3 months prior to Screening and who have a screening thyroid stimulating hormone (TSH) within the normal range may participate.)
   - ALT and/or AST > 2 times the upper limit of normal at screening.
   - Fasting triglycerides > 450mg/dL at screening.
   - Total Bilirubin > 1.5 times the upper limit of normal at screening.
   - For females a haemoglobin < 11.5 g/dL, and for males a hemoglobin < 12.5 g/dL. (A female subject with haemoglobin between 10g/dL and 11.5 g/dL, or a male subject with haemoglobin between 10g/dL and 12.5 g/dL may be enrolled only if the Investigator and the GSK Medical Monitor agree that the finding is unlikely to introduce additional risk to the subject and will not interfere with the study procedures).

- A positive pre-study drug/urine screen. A minimum list of drugs that will be screened for include amphetamines, barbiturates, cocaine, opiates, cannabinoids and benzodiazepines.
- A pre-study urine cotinine screen indicating use of tobacco/ nicotine containing products.

2. If female is pregnant or has a positive pregnancy test.
3. Significant renal disease as manifested by one or more of the following:

- Glomerular filtration rate (or creatinine clearance) <60mL/min. (estimated from serum creatinine (SCr) and demographic data using the MDRD calculation):
    - To calculate estimated GFR (mL/min/1.73m^2) manually:

      = 186 x (SCr in mg/dL)$^{-1.154}$ x (age)$^{-0.203}$ x (0.742 if female) x (1.210 if African-American)

      = exp (5.228-1.154 x ln (SCr)-0.203x ln(age)-(0.299 if female) + (0.192 if African American))

  (A link to a validated MDRD calculator on the internet is provided in the SRM.)

    - Urine protein/creatinine (mg of protein/mg of creatinine) ratio >2.5; **or** urine albumin or protein concentration >300mg/g of creatinine.
    - Known loss of a kidney either by surgical ablation, injury, or disease.

4. Significant ECG abnormalities, defined as follows:

Heart Rate	< 50 and >100bpm
PR Interval	<120 and > 220ms
QRS duration	< 70 and >120ms
QT_C Interval (Bazett)*	> 450ms

Or, has clinically significant rhythm abnormalities identified during 24-hour Screening Holter assessment. Subjects with Left Bundle Branch Block are excluded from the study. Subjects with partial Right Bundle Branch Block may be considered for inclusion following consultation with the GSK Medical Monitor. Subjects with WPW syndrome are excluded from the study.

*Note that if ECG abnormalities are identified that may be variable or reversible, the ECG should be repeated two more times (with 5 minutes between ECG readings) and the average of the 3 values used to determine eligibility.

5. Systolic pressure > 150mmHg or <80mmHg or diastolic blood pressure > 95mmHg or <60mmHg at screening. Blood pressure assessments may be repeated once if needed, allowing adequate time for subject to rest.
6. Previous use of insulin as a treatment within 3 months of Screening, or for >2 weeks when used for acute illness in the last 12 months prior to Screening, or if used for more than 1 year when associated with gestational diabetes mellitus.

7. Has a history of any of the following conditions:
   - Clinically significant symptoms of gastroparesis
   - Cholelithiasis or obstructive or inflammatory gallbladder disease within 3 months prior to Screening
   - Gastrointestinal disease that could affect fat or bile acid absorption, or the pharmacokinetics or pharmacodynamics of the study drugs, including inflammatory bowel disease, chronic diarrhea, Crohn's or malabsorption syndromes within the past year
   - Gastrointestinal surgery that may affect the pharmacokinetics or pharmacodynamics of the study drugs

     Subjects may be enrolled in the study if they have had a cholecystectomy three or more months before the time of screening and are stable and asymptomatic.
   - Chronic or acute pancreatitis
8. History of regular alcohol consumption within 6 months of the study defined as:
   - An average weekly intake of >14 drinks for males or >7 drinks for females. One drink is equivalent to 12 g of alcohol: 12 ounces (360mL) of beer, 5 ounces (150mL) of wine or 1.5 ounces (45mL) of 80 proof distilled spirits.
9. Urinary cotinine levels indicative of smoking or history or regular use of tobacco- or nicotine-containing products within 6 months prior to screening.
10. Has participated in a clinical trial and has received a drug or a new chemical entity within 30 days or 5 half-lives, or twice the duration of the biological effect of any drug (whichever is longer) prior to the first dose of current study medication.
11. Exposure to more than four new chemical entities within 12 months prior to the first dosing day.
12. Is taking prohibited medications. See Section 9.3 for a detailed list of prohibited medications. Note also:
    - The use of anti-diabetic agents other than those listed in Inclusion #6 and 7 is reason for exclusion and subjects will not be allowed to wash off of unapproved anti-diabetic medications in order to qualify for participation in this study.
    - Subjects must wash out from the following medications during the 7-day period prior to first dose, and must remain off these medications through discharge on post-last-dose of Period 2 (Part B) or Day 15 (Part C): all anti-diabetic medications specified in Inclusion #6 and 7, all statin agents, fat absorption blocking agents, bile acid sequestrants. Fibrates must be washed out for a 14-day period prior to first dose.
    - Vitamins, herbal and dietary supplements (including St John's Wort) are prohibited within 7 days or 5 half-lives (whichever is longer) prior to the first dose of study medication and through discharge.

13. Unwilling to abstain from
    - Caffeine-or xanthine-containing products for 24 hours prior to dosing until post-last-dose of Period 5 (Part A), post-last-dose of Period 2 (Part B) or Day -7 through Day 15 (Part C).
    - Use of illicit drugs or nicotine-containing products
    - Alcohol for 24 hours prior to dosing until post-last-dose of Period 5 (Part A), post-last-dose of Period 2 (Part B) or Day -7 through Day 15 (Part C).
    - Consumption of red wine, Seville oranges, grapefruit or grapefruit juice from 7 days prior to the first dose of study medication until collection of the final pharmacokinetic blood samples.
14. History of sensitivity to any of the study medications, or components thereof or a history of drug or other allergy that, in the opinion of the physician responsible, contraindicates their participation. This includes sensitivity to heparin or heparin-induced thrombocytopenia, if heparin will be used to maintain catheter patency.
15. Where participation in the study would result in donation of blood in excess of approximately 500mL within a 56 day period.
16. Subject is either an immediate family member of a participating investigator, study coordinator, employee of an investigator; or is a member of the staff conducting the study.
17. Unwillingness or inability to follow the procedures outlined in the protocol.
18. Subject is mentally or legally incapacitated.

5.2.3. Other Eligibility Criteria Considerations

To assess any potential impact on subject eligibility with regard to safety, the investigator must refer to the following document(s) for detailed information regarding warnings, precautions, contraindications, adverse events, and other significant data pertaining to the investigational product(s) being used in this study: Investigator’s Brochure for GSK1292263 and supplement [GlaxoSmithKline Document Number RM2008/00434/00 and GlaxoSmithKline Document Number RM2009/00168/00], and Januvia Prescribing Information [**Januvia** Package Insert, 2007].

6. DATA ANALYSIS AND STATISTICAL CONSIDERATIONS

6.1. Hypotheses and Treatment Comparisons

The focus of this study is to evaluate safety and tolerability of GSK1292263 and to estimate GSK1292263 PK parameters and PD effects in subjects with T2DM following single doses and repeat doses of GSK1292263. No formal statistical hypotheses will be tested. Descriptive statistics will be used to assess safety and tolerability objectives. An estimation approach will be used to address the PK and PD study objectives, where point estimates and corresponding confidence intervals will be constructed.

To assess the safety and tolerability of GSK1292263, adverse events and changes in ECGs, vital signs and laboratory values will be evaluated. Treatment comparisons with placebo and sitagliptin will be based on review of descriptive statistics.

Point estimates and 90% confidence intervals for the slope for ln(dose) from the analysis of GSK1292263 PK parameters [AUC(0-τ), AUC(0-24), AUC(0-∞) (as data permit), and Cmax following single dosing on Day 1 and AUC(0-τ), AUC(0-24), Cτ, and Cmax following repeat dosing] will be calculated to assess dose-proportionality. Dose proportionality will also be assessed by comparing the PK parameters for each of the test doses to those for the reference dose and by visual inspection (test and reference doses to be determined). Point estimates and 90% confidence intervals will be presented for each of the differences (test-reference).

Repeat-dose (QD or BID) PK parameters [AUC(0-τ)] will be compared to single-dose PK parameters to assess accumulation [AUC(0-τ)] and time-invariance [AUC(0-∞)] (as data permit). AUC(0-τ), AUC(0-24) and Cmax will be calculated on Days 13 and 14, as data permit, to assess the effect of sitagliptin on GSK1292263 PK (Day 14 versus Day 13). Point estimates and 90% confidence intervals will be presented for each of the comparisons.

AUC(0-24) and Cmax will be calculated, as data permit, for sitagliptin on Day 14 to assess the effect of GSK1292263 on sitagliptin PK (sitagliptin alone versus sitagliptin co-administered with GSK1292263). Point estimates and 90% confidence intervals will be presented for each of the comparisons.

Point estimates and 90% confidence intervals for the slope for day from the analysis of GSK1292263 trough concentration (Cτ) will be calculated to assess achievement of steady state at each dose level.

Fasted and derived PD parameters will be compared between each of the following during the repeat dose part of the study (Part C):

- GSK1292263 Day 13 versus Day -1
- GSK1292263+Sitagliptin Day 14 versus GSK1292263 alone Day 13
- GSK1292263 Day 13 versus Placebo Day 13 (change from baseline)

- Sitagliptin Day 13 versus Placebo Day 13 (change from baseline)
- Sitagliptin Day 13 and Day 14 versus Day -1
- GSK1292263+Sitagliptin Day 14 versus Placebo+Sitagliptin Day 14 (change from baseline)
- GSK1292263 Day 7 versus Placebo Day 7
- Sitagliptin Day 7 versus Placebo Day 7

In the single dose part of the study (Part A), fasted and derived PD parameters will be compared between each GSK1292263-treated group and placebo and between GSK1292263 and sitagliptin. Point estimates and 95% confidence intervals will be presented for each of the differences (GSK1292263-placebo and GSK1292263-sitagliptin).

No adjustments for multiple comparisons will be made.

6.2. Sample Size Considerations

6.2.1. Sample Size Assumptions

The sample size for all parts of this study is based primarily on feasibility considerations; however, for the repeat dose part, as a reference, provided below is an estimate of how the targeted sample size affects the precision of the estimation and the power of detecting a clinically relevant difference between active treatment and placebo for the primary PD endpoints.

Except for Part B, this study is targeted to have 12 subjects complete each treatment group. A further 4 subjects may be enrolled into each of the 3 GSK1292263 arms of Cohort 3. Based on data from study KG2104940 using an investigation GSK anti-diabetic drug [GlaxoSmithKline Document Number RM2006/00553/00] estimates of square root of the mean square errors for change from baseline fasting plasma glucose (FPG) and glucose weighted mean AUC(0-24) on Day 11 are 23.8mg/dL and 17.0mg/dL, respectively. Based on the sample size and variability estimates above, expected half-widths of the 95% CI for the mean difference between active treatment and placebo and the difference detectable with 80% or 90% power are provided in the table below.

Parameter	Common SD	Sample Size		Expected Half-Width of 95% CI	Difference detectable with power[1]	
		Active	Placebo		90%	80%
FPG (mg/dL)	23.8	12	12	20.2	32.0	28.5
Weighted Mean AUC(0-24) (mg/dL)	17.0	12	12	14.4	23.5	20.3

1. Based on a two-sided two-sample t-test at significance level (α) of 0.05 (unadjusted for multiple comparisons).

6.2.2. Sample Size Sensitivity

Based on data from study GLP106073 using an investigation GSK anti-diabetic drug [GlaxoSmithKline Document Number RM2007/00229/00] estimates of square root of the mean square errors for change from baseline FPG and glucose weighted mean AUC(0-24) on Day 9 are 25.9mg/dL and 27.1mg/dL, respectively. Effects of this increase in variability on the precision and power are shown in the Table 14.

Table 14 Sample Size Sensitivity

Parameter	Common SD	Sample Size		Expected Half-Width of 95% CI	Difference detectable with power[1]	
		Active	Placebo		90%	80%
FPG (mg/dL)	25.9	12	12	21.9	35.9	31.0
Weighted Mean AUC(0-24) (mg/dL)	27.1	12	12	22.9	37.5	32.4

1. Based on a two-sided two-sample t-test at significance level (α) of 0.05 (unadjusted for multiple comparisons).

6.2.3. Sample Size Re-estimation

No sample size re-estimation will be performed.

6.3. Data Analysis Considerations

6.3.1. Interim Analysis

There will be no formal statistical interim analysis. However, reviews of safety, tolerability, pharmacokinetic, and pharmacodynamic data may be performed by the GSK study team during the study. This analysis can include review of individual subject data and basic summaries and graphs. The GSK study team will have access to an unblinded copy of the randomization schedule. Subjects and all site personnel, with the exception of the study pharmacists, will be blinded to subject randomization throughout the trial.

The preliminary results from available safety data may be reported for the purposes of safety review by GSK, the study investigators, and where required by regulatory bodies prior to database freeze.

If safety, tolerability, PK and PD from Part C are satisfactory after completion of 12 subjects in each arm, then a further 4 subjects will be enrolled into each of the GSK1292263 arms.

6.3.2. Final Analyses

The final planned analyses will be performed after all subjects have completed each part of the study after database freeze/unblinding. Version 9.1 or higher of the SAS system will be used to analyze the data as well as to generate tables, figures, and listings. Complete details will be documented in the Reporting and Analysis Plan (RAP).

6.3.2.1. Safety Analyses

Safety data will be presented in tabular and/or graphical format and summarized descriptively according to GSK's Integrated Data Standards Library (IDSL) standards.

The GSK Division of Discovery Biometrics will conduct summary analyses of safety assessments. No formal statistical comparisons will be made for the safety data. Details of the safety analyses will be provided in the RAP.

6.3.2.2. Pharmacokinetic Analyses

Pharmacokinetic analysis will be the responsibility of the Clinical Pharmacology Modeling and Simulation Department, GlaxoSmithKline. Plasma GSK1292263 and sitagliptin concentration-time data will be analyzed by non-compartmental methods with WinNonlin Professional Version 5.2 or higher. Calculations will be based on the actual sampling times recorded during the study. From the plasma concentration-time data, the following pharmacokinetic parameters will be determined, as data permit.

Following single dosing in Parts A and B:

Maximum observed plasma concentration (Cmax), time to Cmax (tmax), area under the plasma concentration-time curve [AUC(0-τ) and AUC(0-∞)], apparent terminal phase half-life (t½), and, as data permit, the lag time before observation of drug concentrations in the plasma (tlag), the apparent clearance CL/F, and the apparent volume of distribution V/F.

Following repeat dosing in Part C:

Cmax, tmax, area under the plasma concentration-time curve over the dosing interval [AUC(0-τ), AUC(0-24)] and AUC(0-12) on Day 7, pre-dose (trough) concentration at the end of the dosing interval (Cτ), and t½ will be estimated for GSWK1292263 ,as data permit.

Cmax, tmax, and area under the plasma concentration-time curve over the dosing interval [AUC(0-24)] on Day 14, will be estimated for sitagliptin (alone and in combination with GSK1292263), as data permit.

AUC(0-∞), and Cmax following dosing on Day 1 and AUC(0-τ), Cτ, and Cmax following repeat dosing will be used for assessment of dose proportionality of GSK1292263. Trough concentration (Cτ) samples collected on Days 4, 5, 6 and 7 will be used to assess attainment of steady state for GSK1292263. To estimate the extent of

accumulation and assess time-invariance of GSK1292263 after repeat dosing, the observed accumulation ratio (Ro) and time-invariance ratio (Rs) will be determined. If AUC(0-∞) on Day 1 cannot be accurately estimated due to inadequate sampling periods and large extrapolated areas, the dose proportionality assessment will not include AUC(0-∞) and the time-invariance ratio will not be provided.

AUC(0-τ), AUC(0-24), and Cmax on Days 13 and 14 will be used to assess the effect of sitagliptin on GSK1292263 pharmacokinetics, as data permit.

AUC(0-τ), AUC(0-24), and Cmax on Days 13 and 14 for GSK1292263 will be used to assess the effect of sitagliptin on GSK1292263 pharmacokinetics, as data permit.

AUC(0-24) and Cmax on Day 14 for sitagliptin (alone and when co-administered with GSK1292263) will be used to assess the effect of GSK1292263 on sitagliptin pharmacokinetics, as data permit.

Additional or different PK parameters may be calculated if necessary.

Pharmacokinetic data will be presented in graphical and/or tabular form and will be summarized descriptively. Descriptive statistics (n, arithmetic mean, standard deviation, 95% CI, minimum, median, and maximum) will be calculated for all pharmacokinetic parameters by treatment and day. For $\log_e$-transformed variables geometric mean, 95% confidence interval and %CVb ($100 * \sqrt{(\exp(SD^2) - 1)}$) will be provided, where the SD is the standard deviation of log-transformed data. All pharmacokinetic data will be stored in the Archives, GlaxoSmithKline Pharmaceuticals, R&D.

Statistical analyses of the pharmacokinetic parameter data will be the responsibility of Discovery Biometrics, GlaxoSmithKline. Dose proportionality, accumulation, time-invariance, and achievement of steady state will be analyzed by using appropriate power, ANOVA, or linear regression models. Details of the statistical analysis of PK data will be provided in the RAP.

No formal analyses of sitagliptin pharmacokinetic data will be performed. Details will be provided in the RAP.

6.3.2.3. Pharmacokinetic/Pharmacodynamic Analyses

The relationship between the plasma concentrations of GSK1292263 and sitagliptin or the corresponding metrics of systemic exposure (Cmax and AUC) and the PD endpoints will be explored using appropriate PK/PD modelling techniques, as data permit.

6.3.2.4. Pharmacodynamic Analyses

Analyses of pharmacodynamic data will be the responsibility of Discovery Biometrics, GlaxoSmithKline. PD data will be presented in graphical and/or tabular form and will be summarized descriptively.

PD parameters, including fasting PD, maximum PD, and weighted mean AUCs, along with the change from baseline values will be derived and summarized.

In Part A, an analysis of variance (ANOVA) with a fixed effect term for treatment will be fitted with the weighted mean AUC as the dependent variable. The ANOVA model will include factors for treatment, period, and subject. Subject will be fitted as a random effect in the model. The point estimates and corresponding 95% confidence intervals for treatment ratios will be calculated for treatment comparisons versus placebo and back-transformed for reporting purposes.

In Part B, analyses will be performed similar to those used for Part A.

In Part C, an analysis of covariance (ANCOVA) with a fixed effect terms for treatment and prior anti-diabetic treatment will be fitted with the post-baseline weighted mean minus baseline (Day -1 weighted mean) as the dependent variable and the Day -1 weighted mean as a covariate. Pairwise differences in least squares means between each GSK1292263 active treatment and placebo will be calculated, and 95% confidence intervals will be constructed for these differences.

Subgroup analysis of PD data may be performed by separately analyzing drug naïve, single-treated, and combo-treated patients, if data permit. Further subgroups may be defined.

Details of the statistical analysis of PD data will be provided in the RAP.

6.3.2.5. Novel Biomarker(s) Analyses

The results of any novel biomarker investigations will be reported separately from the main clinical study report. All endpoints of interest from all comparisons will be descriptively and/or graphically summarized as appropriate to the data.

Additional exploratory analyses may be performed to further characterize the novel biomarker(s).

6.3.2.6. Hunger, Craving, and Fullness Questionnaire and Calorie Counts

The HCFQ data will be listed and summarized with frequency counts at each time point by treatment. Calorie count data will be summarized at each time point. Additional details will be provided in the RAP.

7. STUDY ASSESSMENTS AND PROCEDURES

This section lists the parameters of each planned study assessment. The exact timing of each assessment is listed in the Time and Events Tables (Section 4.5 and Section 4.6). Detailed procedures for obtaining each assessment are provided in the Study Procedures Manual (SPM). Whenever vitals signs, 12-lead ECGs and blood draws are scheduled for the same nominal time, the assessments should occur in the following order: 12-lead ECG, vital signs, blood draws. When procedures are close to dosing and meals, the

following order will be employed, as appropriate: ECGs, vitals, blood sampling, meal ingestion, dosing. The timing of the assessments should allow the blood draw to occur at the exact nominal time.

The timing and number of planned study assessments, including safety, pharmacokinetic, pharmacodynamic/biomarker or other assessments may be altered during the course of the study based on newly available data (e.g., to obtain data closer to the time of peak plasma concentrations) to ensure appropriate monitoring. The change in timing or addition of time points for any planned study assessments must be approved and documented by GSK, but this will not constitute a protocol amendment. The IRB will be informed of any safety issues that require alteration of the safety monitoring scheme. No more than approximately 500mL of blood will be collected over a 30-day period in the study, including any extra assessments that may be required.

7.1. Demographic/Medical History Assessments

The following demographic parameters will be captured: date of birth, gender, race and ethnicity.

Medical/medication/alcohol history will be assessed as related to the eligibility criteria listed in Section 5.2.

7.2. Safety

Planned timepoints for all safety assessments are listed in the Time and Events Tables (Section 4.5 and Section 4.6). Additional time points for safety tests such as vital signs, physical exams and laboratory safety tests may be added during the course of the study based on newly available data to ensure appropriate safety monitoring.

7.2.1. Physical Exams

- A complete physical examination will include assessments of the head, eyes, ears, nose, throat, skin, thyroid, neurological, lungs, cardiovascular, abdomen (liver and spleen), lymph nodes and extremities. Height, weight, waist circumference will also be measured and recorded.
- A brief physical examination will include assessments of the skin, lungs, cardiovascular system, and abdomen (liver and spleen).

7.2.2. Vital Signs

- Vital sign measurements will include systolic and diastolic blood pressure and pulse rate.
- At each time point, assessment should be performed after resting in a supine or semi-supine position for at least 10 minutes.

7.2.3. Electrocardiogram (ECG)

- 12-lead ECGs will be obtained in a supine position at each timepoint noted in the Time and Events Tables (Section 4.5 and Section 4.6) during the study using an ECG machine that automatically calculates the heart rate and measures PR, QRS, QT, and QTc intervals. Refer to Section 4.4.2 for QTc withdrawal criteria and additional QTc readings that may be necessary.
- Continuous cardiac telemetry will be performed in Parts A and B as noted in the Time and Events Table (Section 4.5). Full disclosures will be maintained as part of the subject's source documents and will be reviewed in detail.

7.2.4. Clinical Laboratory Assessments

Hematology, clinical chemistry, urinalysis and additional parameters to be tested are listed below:

Hematology

Platelet Count	*RBC Indices*:	*Automated WBC Differential:*
RBC Count	MCV	Neutrophils
WBC Count (absolute)	MCH	Lymphocytes
Reticulocyte Count	MCHC	Monocytes
Hemoglobin		Eosinophils
Hematocrit		Basophils

Clinical Chemistry

BUN	Potassium	AST (SGOT)	Total and direct bilirubin
Creatinine	Chloride	ALT (SGPT)	Uric Acid
Glucose, fasting	Total CO_2	GGT	Albumin
Sodium	Calcium	Alkaline phosphatase	Total Protein
Magnesium	Triglycerides	Total Cholesterol	LDL cholesterol
Phosphorus	FFA (NEFA)	HDL cholesterol	

Routine Urinalysis

Specific gravity
Urine protein and microalbumin
pH, glucose, protein, blood and ketones by dipstick
Microscopic examination (if blood or protein is abnormal)

Other tests

HbA1c (screening)
HIV
Hepatitis B (HBsAg)
Hepatitis C (Hep C antibody -- if second generation Hepatitis C antibody positive, a hepatitis C antibody Chiron RIBA immunoblot assay should be reflexively performed on the same sample to confirm the result)
FSH and estradiol (as needed in women of non-child bearing potential only)
Alcohol and drug screen (to include at minimum: amphetamines, barbiturates, cocaine, opiates, cannabinoids and benzodiazepines).

7.3. Pregnancy

7.3.1. Time period for collecting pregnancy information

All female participants in the study must be of non-child bearing potential, with post-menopausal status confirmed at Screening by FSH and estradiol testing. A pregnancy test will be performed on Day -1 and at the Follow-up visit.

All pregnancies in female subjects will be collected after the start of dosing and until the follow-up visit.

7.3.2. Action to be taken if pregnancy occurs

The investigator will collect pregnancy information on any female subject, who becomes pregnant while participating in this study. The investigator will record pregnancy information on the appropriate form and submit it to GSK within 2 weeks of learning of a subject's pregnancy. The subject will also be followed to determine the outcome of the pregnancy. Information on the status of the mother and child will be forwarded to GSK. Generally, follow-up will be no longer than 6 to 8 weeks following the estimated delivery date. Any premature termination of the pregnancy will be reported.

While pregnancy itself is not considered to be an AE or SAE, any pregnancy complication or elective termination of a pregnancy for medical reasons will be recorded as an AE or SAE (see AE/SAE section of the protocol and the SPM for definitions and a description of follow-up).

A spontaneous abortion is always considered to be an SAE and will be reported as such. Furthermore, any SAE occurring as a result of a post-study pregnancy and is considered reasonably related to the investigational product by the investigator, will be reported to GSK as described in section entitled, "Post-study AEs and SAEs" of the SPM. While the investigator is not obligated to actively seek this information in former study participants, he or she may learn of an SAE through spontaneous reporting.

Any female subject who becomes pregnant while participating will be withdrawn from the study.

7.4. Pharmacokinetics

7.4.1. Blood Sample Collection

Blood samples for pharmacokinetic analysis of GSK1292263 will be collected at the time points indicated in Section 4.5 and Section 4.6, Time and Events Tables. The actual date and time of each blood sample collection will be recorded. The timing of PK samples may be altered and/or PK samples may be obtained at additional time points to ensure thorough PK monitoring.

Details of PK blood sample collection (including volume to be collected), processing, storage and shipping procedures are provided in the Study Procedures Manual (SPM).

7.4.2. Sample Analysis

Plasma/serum analysis will be performed under the management of Worldwide Bioanalysis, DMPK, GlaxoSmithKline. Concentrations of GSK1292263 will be determined in plasma samples using the currently approved analytical methodology. Raw data will be stored in the GLP Archives, GlaxoSmithKline. Once the plasma has been analyzed for GSK1292263 any remaining plasma may be analyzed qualitatively for other circulating metabolites and the results reported under a separate DMPK protocol.

7.5. Biomarker(s)/Pharmacodynamic Markers

7.5.1. Type II Diabetes Biomarkers/Pharmacodynamic Markers

Blood samples for assessment of 24-hour glucose, insulin, C-peptide, glucagon, GLP-1 (total and active), total GIP, total PYY and glycerol will be collected at the timepoints indicated in the Time and Events Tables in Section 4.5and Section 4.6. The timing of the collections may be adjusted on the basis of emerging PK or PD data from this study or other new information in order to ensure optimal evaluation of the PD endpoints.

7.5.2. Exploratory Biomarkers

Blood sample(s) will be collected during this study and may be used for the purposes of measuring exploratory biomarkers (e.g., adiponectin, leptin, ghrelin, amylin, PP, CCK) or novel biomarkers to identify factors that may influence diabetes, and/or medically related conditions, as well as the biological and clinical responses to GSK1292263, including adverse events, if relevant.

Exploratory biomarkers, if analyzed, will be assessed from the PD blood samples.

7.6. Pharmacogenetics

Information regarding pharmacogenetic (PGx) research is included in Appendix 2: Pharmacogenetic research: Pharmacogenetic Research. The IRB and, where required, the applicable regulatory agency must approve the PGx assessments before these can be conducted at the site. In some cases, approval of the PGx assessments can occur after approval is obtained for the rest of the study. If so, then the written approval will clearly indicate approval of the PGx assessments is being deferred and in most cases, the study, except for PGx assessments, can be initiated. When PGx assessments are not approved, the approval for the rest of the study will clearly indicate this and therefore, PGx assessments will not be conducted.

8. LIFESTYLE AND/OR DIETARY RESTRICTIONS

Category	Dietary Guidelines and Restrictions
Exercise	Subjects will abstain from strenuous exercise for 48 hours prior to each blood collection for clinical laboratory tests.<br>Light recreational activity is permitted while subjects are in the clinic.<br>Subjects will be given instructions for exercise when not in the unit.
Fasting	Subjects should fast from all food or drink with the exception of water from midnight prior to each blood collection for clinical chemistry tests.<br>Subjects should fast from all food or drink with the exception of water from midnight prior to each morning administration of study drug. See the SPM for timing of meals in relation to dosing for each part of the study.
Water	Water is permitted until one hour prior to study drug administration when subjects are dosed in the unit in Parts A and B. In Parts A and C, subjects may consume water *ad libitum* after dosing, provided it does not adversely affect the OGTT in Part A. In Part B, subjects may consume water *ad libitum* beginning one hour after dosing.
Breakfast	Breakfast will be omitted on days when the OGTT will be administered (Part A). In Part C, on Days -1, 7, 13 and 14 the breakfast meal will be referred to as a meal tolerance test (MTT). See SPM for menu details. The composition of the breakfast meal on D1 will be the same as that of the MTT.
Lunch	In Parts A and B, a standardized lunch will be fed on Day 1 of each period. In Part C, a standardized lunch will be consumed on Days -1 through 14 at approximately 4h after the morning dosing. The same lunch will be provided on Days -1, 1, 7, 13 and 14.
Evening meal	In Parts A and B, a standardized evening meal will be fed on Day 1 of each period. In Part C, a standardized evening meal will be consumed on Days -1 through 14 at approximately 10h after the morning dose. The same evening meal will be provided on Days -1, 1, 7, 13 and 14. For Cohort 4 (BID or QD dosing), see SPM for dosing in relation to meals and blood sampling.
Evening Snack	No evening snack will be permitted on Day 1 in Part A, and Days -1, 7, 13 and 14 in Part C. On other days, an evening snack will be permitted at approximately 12 - 14 hours after dosing and up to 23:00.
Alcohol, Caffeine, Xanthine	Subjects will not be allowed to consume alcohol or caffeine- or xanthine-containing products (e.g., coffee, tea, cola drinks, chocolate) from 24-hours prior to dosing in Parts A and B or 7 days prior to dosing in Part C until final discharge from the clinic (Day 2 of Period 5 in Part A, Day 2 of Period 2 in Part B, and Day 15 in Part C).
Nicotine	Subjects will not be allowed to smoke or use nicotine-containing products during the course of the study (from Screening until after the final follow-up visit)
Grapefruit, Red Wine, Seville Oranges	Subjects will not be allowed to consume red wine, Seville oranges, grapefruit juice or grapefruit within 7 days prior to the first dose of study medication until final discharge from the clinic (Day 2 of Period 5 in Part A, Day 2 of Period 2 in Part B, and Day 15 in Part C).

8.1. Contraception requirements for Male Subjects

To prevent pregnancy in a female partner or to prevent exposure of any partner to the investigational product from a male subject's semen, male subjects must use one of the following contraceptive methods:

- Abstinence, defined as sexual inactivity consistent with the preferred and usual lifestyle of the subject. Periodic abstinence (e.g. calendar, ovulation, symptom-thermal, post-ovulation methods) and withdrawal are not acceptable methods of contraception.
- Condom *(during non-vaginal intercourse with any partner - male or female)* **OR**
- Condom and occlusive cap (diaphragm or cervical/vault caps) plus spermicidal agent (foam/gel/film/cream/suppository) *(during sexual intercourse with a female)*

9. CONCOMITANT MEDICATIONS AND NON-DRUG THERAPIES

9.1. Permitted Medications

All concomitant medications taken during the study will be recorded in the CRF. Subjects using the following medications must be on stable doses during the 3 months prior to Screening:

- Antihypertensives (e.g., beta blockers, ACE inhibitors, angiotensin II receptor antagonists, calcium channel blockers and thiazide diuretics), with the exception of diltiazem, verapamil, and other CYP3A4 inhibitors as noted below. The dosage(s) of antihypertensive medications should, if medically appropriate, remain unchanged while the subject is enrolled in the study. If a dosage change is necessary, information pertaining to the change must be documented on the concomitant medication pages in the eCRF.
- Thyroid hormone.
- Acetaminophen may be used on an as-needed basis provided the total daily dose does not exceed 2g.
- The use of non-steroidal anti-inflammatory drugs (NSAIDs), especially aspirin, is allowed only if prescribed by a physician on a regular schedule (not intermittently or PRN). Intermittent/ PRN use of NSAIDs is prohibited.

Permitted concomitant medications should be taken as prescribed, and may be taken with water prior to scheduled study clinic visits without being considered to have broken their fast.

Other medications may be permitted following consultation with the GSK Medical Monitor if they are not considered to affect subject safety or the objectives of the study.

9.1.1. Anti-Viral Therapies for Influenza

Tamiflu and Relenza are permitted for the treatment of influenza, following consultation with the GSK Medical Monitor.

9.2. Medications Permitted at Screening but Requiring Washout before Enrollment

The following medications require washout from Day -7 to final discharge from the unit of the study. Discontinuation and resumption of these medications should be documented in the eCRF.

- All antidiabetic medications permitted in Inclusion #5: metformin, sulfonylureas, DPP-IV inhibitors, exenatide
- Statins and lipid-lowering drugs, including but not limited to the following single or combination products: atorvastatin, simvastatin, lovastatin, pravastatin, ezetemide, or simvastain / ezetemide combination
- Fat absorption blocking agents
- Bile acid sequestrants

Fibrates must be washed out from Day -14 to final discharge from the unit.

Other prior medications may be permitted that require washout following consultation with the GSK Medical Monitor if they are not considered to affect subject safety of the objectives of the study.

9.3. Prohibited Medications

- The use of anti-diabetic agents other than those listed in Inclusion # 5 is reason for exclusion and subjects will not be allowed to wash off of unapproved anti-diabetic medications in order to qualify for participation in this study. Insulin use is prohibited.
- Potent inhibitors of CYP3A4, including but not limited to diltiazem, verapamil, ketoconazole, cyclosporine.
- Potent inducers of CYP3A4, including but not limited to rifampicin, carbamazepine, phenobarbital, phenytoin.
- Loop diuretics (e.g., bumetanide, torsemide, furosemide/ frusemide).
- Oral anticoagulants, including warfarin (note that aspirin and non-steroidal anti-inflammatory drugs are permitted as noted above).
- Oral or injectable corticosteroids (inhaled, intranasal and topical are permitted).
- Antiretroviral drugs.

- Methotrexate, cyclosporine or monoclonal antibodies (e.g., infliximab, adalimumab, etanercept, certolizimab pegol, rituximab) for autoimmune disease, rheumatoid arthritis or psoriasis.
- Atypical antipsychotic medications (e.g., aripiprazole, risperidone, clozapine, olanzapine, quetiapine, and ziprasidone).
- Monoamine oxidase inhibitors, selective serotonin reuptake inhibitors (SSRIs) or tricyclic antidepressants taken within the previous three months.
- Use of other drugs that may affect glucose and/or lipid metabolism such as danazol and niacin, are prohibited within 3 months prior to Day -7 and/or during the study.

9.4. Non-Drug Therapies

- Vitamins, herbal and dietary supplements (including St John's Wort) are prohibited within 7 days or 5 half-lives (whichever is longer) prior to the first dose of study medication and through final discharge from the unit.

10. COMPLETION OR EARLY WITHDRAWAL OF SUBJECTS

10.1. Subject Completion

A completed subject is one who has completed all phases of the study including the follow-up visit.

The end of the study is defined as the last subject's last visit.

10.2. Subject Withdrawal Criteria

Refer to Section 4.4 for dose adjustment/stopping criteria based on safety/PK/PD criteria.

A subject may withdraw from investigational product at any time at his/her own request, or may be withdrawn at any time at the discretion of the investigator for safety, behavioral or administrative reasons.

10.3. Subject Withdrawal Procedures

10.3.1. Subject Withdrawal from Study

Subjects may withdraw from the study at any time and for any reason. They are not obliged to state the reason for withdrawal. However, the reasons for withdrawal, or failure to provide a reason, must be documented by the physician on the eCRF. Every effort should be made by the physician to follow up subjects who withdraw from the study, by adhering to Follow-up procedures specified in Section 4.5 and Section 4.6.

10.3.2. Subject Withdrawal from Investigational Product

If a subject does not receive all doses of randomized study drug planned for that subject, the subject will be considered to have prematurely discontinued study drug. Every effort should be made by the physician to follow up subjects who withdraw from the study, by adhering to follow-up procedures specified in Section 4.5 and Section 4.6.

Decisions regarding replacement of subjects prematurely discontinued from study drug will be made by the Investigator and GSK Medical Monitor on a case-by-case basis.

10.4. Treatment After the End of the Study

For subjects who washed out of medications, consistent instructions should be provided to the subjects defining how they should resume administration of these medications after the completion of final study procedures (end of Period 5 in Part A, end of Period 2 in Part B, and Day 15 in Part C). The investigator may use his/her discretion with respect to modifying the doses of these medications when they are re-started.

Subjects will not receive any additional treatment after completion of the study because other treatment options are available.

10.5. Screen and Baseline Failures

Data for screen and baseline failures will be collected in source documentation at the site but will not be transmitted to GSK.

11. INVESTIGATIONAL PRODUCT(S)

Investigational product dosage and administration details are listed in Section 4.3.

11.1. Blinding

Subjects and all site personnel, with the exception of the study pharmacists, will be blinded to subject randomization throughout the trial.

Sitagliptin will be administered in this study in an unblinded manner.

Because the GSK study team will be assessing data on a real-time basis while the study is ongoing, the team will be unblinded during the trial to allow accurate correlation of safety and tolerability information with PK and PD values. For this reason, this study is characterized as a single-blind study.

In the case of a **medical emergency or in the event of a serious medical condition**, when knowledge of the investigational product is essential for the clinical management or welfare of the subject, **an investigator or other physician managing the subject may decide to unblind that subject’s treatment code**. The investigator will make every effort to contact the GSK Medical Monitor or appropriate GSK study personnel before

unblinding to discuss options. If the blind is broken for any reason and the investigator is unable to contact GSK prior to unblinding, the investigator must notify GSK **as soon as possible following** the unblinding incident **without revealing the subject's study treatment assignment,** unless the information is important to the safety of subjects remaining in the study. In addition, the investigator will record the date and reason for revealing the blinded treatment assignment for that subject in the appropriate data collection tool.

If a serious adverse event (SAE; as defined in Section 12.2, "Definition of an SAE") is reported to GSK, Global Clinical Safety and Pharmacovigilance (GCSP) staff may unblind the treatment assignment for the individual subject. If an expedited regulatory report to one or more regulatory agencies is required, the report will identify the subject's treatment assignment. When applicable, a copy of the regulatory report may be sent to investigators in accordance with relevant regulations, GSK policy, or both.

11.2. Packaging and Labeling

The contents of the label will be in accordance with all applicable regulatory requirements.

11.3. Preparation/Handling/Storage/Accountability

No special preparation of investigational product is required.

Investigational product must be dispensed or administered according to procedures described herein. Only subjects enrolled in the study may receive investigational product. Only authorized site staff may supply or administer investigational product. All investigational products must be stored in a secure area with access limited to the investigator and authorized site staff. Investigational product is to be stored at up to 30°C. Maintenance of a temperature log (manual or automated) is required.

The investigator, institution, or the head of the medical institution (where applicable) is responsible for investigational product accountability, reconciliation, and record maintenance. The investigator or the head of the medical institution (where applicable), or designated site staff (e.g., storage manager, where applicable) must maintain investigational product accountability records throughout the course of the study. The responsible person(s) will document the amount of investigational product received from and returned to GSK and the amount supplied and/or administered to and/or returned by subjects. The required accountability unit for this study will be a bottle. Discrepancies are to be reconciled or resolved. Procedures for final disposition of unused investigational product are listed in the SPM.

Investigational product is not expected to pose significant occupational safety risk to site staff under normal conditions of use and administration. A Material Safety Data Sheet (MSDS)/equivalent document describing occupational hazards and recommended handling precautions either will be provided to the investigator, where this is required by local laws, or is available upon request from GSK.

11.4. Assessment of Compliance

When subjects are dosed at the study site, they will receive investigational products directly from the investigator or designee, under medical supervision. The date and time of each dose administered in the clinic will be recorded in the source documents. The dose of investigational product(s) will be confirmed prior to dosing by a member of the study site staff other than the person administering the investigational product. Study site personnel will examine each subject's mouth to ensure that the investigational product was ingested.

11.5. Treatment of Investigational Product Overdose

The maximum dose planned for this study is 800mg. For this study, any dose of GSK1292263 greater than 800mg ingested in less than a 24 hour time period will be considered an overdose.

GSK does not recommend specific treatment for an overdose. The investigator will use clinical judgment to treat any overdose. Appropriate supportive treatment should be initiated according to subject's clinical signs and symptoms. Consideration should be given to fluid repletion and administration of exogenous glucose to maintain euglycemia if needed.

In the event of an overdose of sitagliptin, usual supportive measures are recommended (e.g., removal of unabsorbed materials from GI tract, employ clinical monitoring, including obtaining an ECG and initiation of supportive therapy as dictated by the subject's clinical needs).

12. ADVERSE EVENTS (AE) AND SERIOUS ADVERSE EVENTS (SAE)

The investigator or site staff is responsible for detecting, documenting and reporting events that meet the definition of an AE or SAE.

AEs will be collected from the start of the washout period and until the follow-up contact. Medical occurrences that begin prior to the start of investigational product but after obtaining informed consent may be recorded on the Medical History/Current Medical Conditions eCRF.

SAEs will be collected over the same time period as stated above for AEs. However, any SAEs assessed as related to study participation (e.g. investigational product, protocol-mandated procedures, invasive tests, or change in existing therapy) or related to a GSK concomitant medication will be recorded from the time a subject consents to participate in the study up to and including any follow-up contact. All SAEs will be recorded and reported to GSK within 24 hours, as indicated in Section 12.7.

Investigators are not obligated to actively seek AEs or SAEs in former study participants. However, if the investigator learns of any SAE, including a death, at any time after a subject has been discharged from the study, and he/she considers the event reasonably

related to the investigational product or study participation, the investigator would promptly notify GSK.

12.1. Definition of Adverse Events

An AE is any untoward medical occurrence in a patient or clinical investigation subject, temporally associated with the use of a medicinal product, whether or not considered related to the medicinal product.

Note: An AE can therefore be any unfavorable and unintended sign (including an abnormal laboratory finding), symptom, or disease (new or exacerbated) temporally associated with the use of a medicinal product.

Events meeting the definition of an AE **include**:

- Any abnormal laboratory test results (hematology, clinical chemistry, or urinalysis) or other safety assessments (e.g., ECGs, radiological scans, vital signs measurements), including those that worsen from baseline, and felt to be clinically significant in the medical and scientific judgement of the investigator.
- Exacerbation of a chronic or intermittent pre-existing condition including either an increase in frequency and/or intensity of the condition.
- New conditions detected or diagnosed after investigational product administration even though it may have been present prior to the start of the study.
- Signs, symptoms, or the clinical sequelae of a suspected interaction.
- Signs, symptoms, or the clinical sequelae of a suspected overdose of either investigational product or a concomitant medication (overdose per se will not be reported as an AE/SAE).

Events that **do not** meet the definition of an AE include:

- Any clinically significant abnormal laboratory findings or other abnormal safety assessments that are associated with the underlying disease, unless judged by the investigator to be more severe than expected for the subject’s condition.
- The disease/disorder being studied, or expected progression, signs, or symptoms of the disease/disorder being studied, unless more severe than expected for the subject’s condition.
- Medical or surgical procedure (e.g., endoscopy, appendectomy); the condition that leads to the procedure is an AE.
- Situations where an untoward medical occurrence did not occur (social and/or convenience admission to a hospital).
- Anticipated day-to-day fluctuations of pre-existing disease(s) or condition(s) present or detected at the start of the study that do not worsen.

12.2. Definition of Serious Adverse Events

If an event is not an AE per Section 12.1, then it can not be an SAE even if serious conditions are met (e.g., hospitalization for signs/symptoms of the disease under study, death due to progression of disease, etc).

An SAE is any untoward medical occurrence that, at any dose:

a. Results in death

b. Is life-threatening

   NOTE: The term 'life-threatening' in the definition of 'serious' refers to an event in which the subject was at risk of death at the time of the event. It does not refer to an event, which hypothetically might have caused death, if it were more severe.

c. Requires hospitalization or prolongation of existing hospitalization

   NOTE: In general, hospitalization signifies that the subject has been detained (usually involving at least an overnight stay) at the hospital or emergency ward for observation and/or treatment that would not have been appropriate in the physician's office or out-patient setting. Complications that occur during hospitalization are AEs. If a complication prolongs hospitalization or fulfills any other serious criteria, the event is serious. When in doubt as to whether "hospitalization" occurred or was necessary, the AE should be considered serious.

   Hospitalization for elective treatment of a pre-existing condition that did not worsen from baseline is not considered an AE.

d. Results in disability/incapacity, or

   NOTE: The term disability means a substantial disruption of a person's ability to conduct normal life functions. This definition is not intended to include experiences of relatively minor medical significance such as uncomplicated headache, nausea, vomiting, diarrhea, influenza, and accidental trauma (e.g. sprained ankle) which may interfere or prevent everyday life functions but do not constitute a substantial disruption.

e. Is a congenital anomaly/birth defect

f. Medical or scientific judgment should be exercised in deciding whether reporting is appropriate in other situations, such as important medical events that may not be immediately life-threatening or result in death or hospitalization but may jeopardize the subject or may require medical or surgical intervention to prevent one of the other outcomes listed in the above definition. These should also be considered serious. Examples of such events are invasive or malignant cancers, intensive treatment in an emergency room or at home for allergic bronchospasm, blood dyscrasias or convulsions that do not result in hospitalization, or development of drug dependency or drug abuse.

g. ALT $\geq$3xULN and bilirubin $\geq$ 2xULN (see Section 13).

12.3. Method of Detecting AEs and SAEs

Care will be taken not to introduce bias when detecting AEs and/or SAEs. Open-ended and non-leading verbal questioning of the subject is the preferred method to inquire about AE occurrence. Appropriate questions include:

- "How are you feeling?"
- "Have you had any (other) medical problems since your last visit/contact?"
- "Have you taken any new medicines, other than those provided in this study, since your last visit/contact?"

12.4. Recording of AEs and SAEs

When an AE/SAE occurs, it is the responsibility of the investigator to review all documentation (e.g., hospital progress notes, laboratory, and diagnostics reports) relative to the event. The investigator will then record all relevant information regarding an AE/SAE in the appropriate data collection tool.

It is not acceptable for the investigator to send photocopies of the subject's medical records to GSK in lieu of completion of the GSK, AE/SAE data collection tool. However, there may be instances when copies of medical records for certain cases are requested by GSK. In this instance, all subject identifiers, with the exception of the subject number, will be blinded on the copies of the medical records prior to submission of to GSK.

The investigator will attempt to establish a diagnosis of the event based on signs, symptoms, and/or other clinical information. In such cases, the diagnosis will be documented as the AE/SAE and not the individual signs/symptoms.

12.5. Evaluating AEs and SAEs

12.5.1. Assessment of Intensity

The investigator will make an assessment of intensity for each AE and SAE reported during the study and will assign it to one of the following categories:

Mild: An event that is easily tolerated by the subject, causing minimal discomfort and not interfering with everyday activities.

Moderate: An event that is sufficiently discomforting to interfere with normal everyday activities.

Severe: An event that prevents normal everyday activities.

An AE that is assessed as severe will not be confused with an SAE. Severity is a category utilized for rating the intensity of an event; and both AEs and SAEs can be assessed as severe. An event is defined as 'serious' when it meets at least one of the pre-defined outcomes as described in the definition of an SAE.

12.5.2. Assessment of Causality

The investigator is obligated to assess the relationship between investigational product and the occurrence of each AE/SAE. A "reasonable possibility" is meant to convey that there are facts/evidence or arguments to suggest a causal relationship, rather than a relationship cannot be ruled out. The investigator will use clinical judgment to determine the relationship. Alternative causes, such as natural history of the underlying diseases, concomitant therapy, other risk factors, and the temporal relationship of the event to the investigational product will be considered and investigated. The investigator will also consult the Investigator Brochure (IB) and/or Product Information, for marketed products, in the determination of his/her assessment.

There may be situations when an SAE has occurred and the investigator has minimal information to include in the initial report to GSK. However, **it is very important that the investigator always make an assessment of causality for every event prior to the initial transmission of the SAE data to GSK.** The investigator may change his/her opinion of causality in light of follow-up information, amending the SAE data collection tool accordingly. The causality assessment is one of the criteria used when determining regulatory reporting requirements.

12.6. Follow-up of AEs and SAEs

After the initial AE/SAE report, the investigator is required to proactively follow each subject at subsequent visits/contacts. All AEs and SAEs will be followed until resolution, until the condition stabilizes, until the event is otherwise explained, or until the subject is lost to follow-up.

The investigator is obligated to perform or arrange for the conduct of supplemental measurements and/or evaluations as may be indicated or as requested by GSK to elucidate as fully as possible the nature and/or causality of the AE or SAE. The investigator is obligated to assist. This may include additional laboratory tests or investigations, histopathological examinations or consultation with other health care professionals. If a subject dies during participation in the study or during a recognized follow-up period, the investigator will provide GSK with a copy of any post-mortem findings, including histopathology.

New or updated information will be recorded in the originally completed data collection tool. The investigator will submit any updated SAE data to GSK within the designated reporting time frames.

12.7. Prompt Reporting of SAEs to GSK

Once the investigator determines that an event meets the protocol definition of an SAE, the SAE will be reported to GSK **within 24 hours**. Any follow-up information on a previously reported SAE will also be reported to GSK within 24 hours.

If the investigator does not have all information regarding an SAE, he/she will not wait to receive additional information before notifying GSK of the event and completing the appropriate data collection tool. The investigator will always provide an assessment of causality at the time of the initial report as described in Section 12.5.2, Assessment of Causality.

The primary mechanism for reporting SAEs to GSK will be the electronic data collection tool (e.g., InForm system). If the electronic system is unavailable for greater than 24 hours, the site will use the paper SAE data collection tool and fax it to the GSK Medical Monitor. Then the site will enter the serious adverse event data into the electronic system as soon as it becomes available.

After the study is completed at a given site, the electronic data collection tool (e.g., InForm system) will be taken off-line to prevent the entry of new data or changes to existing data. If a site receives a report of a new SAE from a study participant or receives updated data on a previously reported SAE after the electronic data collection tool has been taken off-line, the site can report this information on a paper SAE form or to their GSK protocol contact by telephone.

GSK contacts for SAE receipt can be found at the beginning of this protocol on the Sponsor/Medical Monitor Contact Information page.

12.8. Regulatory Reporting Requirements for SAEs

Prompt notification of SAEs by the investigator to GSK is essential so that legal obligations and ethical responsibilities towards the safety of subjects are met.

GSK has a legal responsibility to notify both the local regulatory authority and other regulatory agencies about the safety of a product under clinical investigation. GSK will comply with country specific regulatory requirements relating to safety reporting to regulatory authorities, IRBs/IECs and investigators.

Investigator safety reports are prepared for suspected unexpected serious adverse reactions according to local regulatory requirements and GSK policy and are forwarded to investigators as necessary. An investigator who receives an investigator safety report describing an SAE(s) or other specific safety information (e.g., summary or listing of SAEs) from GSK will file it with the IB and will notify the IRB, if appropriate according to local requirements.

13. LIVER CHEMISTRY TESTING PROCEDURES

Refer to the diagram in Appendix 1 for a visual presentation of the procedures listed below.

The procedures listed below are to be followed if a subject meets any of the liver chemistry stopping criteria defined in Section 4.4.1:

- Immediately and permanently withdraw the subject from investigational product
- Notify the GSK medical monitor within 24 hours of learning of the abnormality to confirm the subject's investigational product cessation and follow-up.
- Complete the "Safety Follow-Up Procedures" listed below.
- Complete the liver event case report forms. If the event also meets the criteria of an SAE (see Section 12.2), the SAE data collection tool will be completed separately with the relevant details.
- Upon completion of the safety follow-up permanently withdraw the subject from the study and do not rechallenge with investigational product.

Safety Follow-Up Procedures for subjects with ALT ≥3xULN and bilirubin ≥ 2xULN (Stopping Criteria #1):

- This event is considered an SAE (see Section 12.2). Serum bilirubin fractionation should be performed if testing is available. If fractionation is unavailable, urinary bilirubin is to be measured via dipstick (a measurement of direct bilirubin, which would suggest liver injury).
- Make every reasonable attempt to have the subjects return to the clinic (within 24 hours) for repeat liver chemistries, additional testing and to be monitored closely (with specialist or hepatology consultation recommended).
- Monitor subjects twice weekly until liver chemistries (ALT, AST, alkaline phosphatase, bilirubin) resolve, stabilize or return to within baseline values.

Safety Follow-Up Procedures for subjects with ALT ≥ 5xULN or ALT ≥ 3xULN who have hepatitis symptoms or rash, can't be monitored for 4 weeks (Stopping Criteria #2 and #3):

- Make every reasonable attempt to have the subject return to the clinic within 24-72 hrs for repeat liver chemistries and additional testing.
- Monitor subjects weekly until liver chemistries (ALT, AST, alkaline phosphatase, bilirubin) resolve, stabilize or return to within baseline values.

Safety Follow-Up Procedures for subjects with ALT ≥ 3xULN and < 5xULN and bilirubin < 2xULN, who do not exhibit hepatitis symptoms or rash:

- Notify the GSK medical monitor within 24 hours of learning of the abnormality to discuss subject safety.

- Subject can continue investigational product if liver chemistries (ALT, AST, alkaline phosphatase, bilirubin) can be monitored weekly for up to 4 weeks.
- If at any point these subjects meet the liver chemistry stopping criteria (outlined in Section 4.4.1), immediately withdraw investigational product, perform additional testing and continue safety follow-up until liver chemistries resolve, stabilize or return to baseline values.
- After 4 weeks of monitoring, if ALT < 3xULN and bilirubin < 2xULN, subjects must be monitored twice monthly until liver chemistries normalize or return to within baseline values.

Additional Follow-Up Procedures for subjects who meet *any* of the stopping criteria:

- Viral hepatitis serology including:
  - Hepatitis A IgM antibody;
  - Hepatitis B surface antigen and Hepatitis B Core Antibody (IgM);
  - Hepatitis C RNA;
  - Cytomegalovirus IgM antibody;
  - Epstein-Barr viral capsid antigen IgM antibody (or if unavailable, obtain heterophile antibody or monospot testing);
  - Hepatitis E IgM antibody (if subject resides outside the USA or Canada, or has traveled outside USA or Canada in past 3 months)
- Blood sample for pharmacokinetic (PK) analysis, obtained within 24hrs of last dose. Record the date/time of the PK blood sample draw and the date/time of the last dose of investigational product prior to blood sample draw on the CRF. If the date or time of the last dose is unclear, provide the subject's best approximation. If the date/time of the last dose can not be approximated OR a PK sample can not be collected in the time period indicated above, do not obtain a PK sample. Instructions for sample handling and shipping are included in the SPM.
- Serum creatine phosphokinase (CPK) and lactate dehydrogenase (LDH).
- Fractionate bilirubin, if total bilirubin ≥ 2xULN.
- Assess eosinophilia
- Record the appearance or worsening of clinical symptoms of hepatitis (fatigue, nausea, vomiting, right upper quadrant pain or tenderness, fever, rash or eosinophilia) as relevant on the AE CRF
- Record use of concomitant medications, acetaminophen, herbal remedies, other over the counter medications, or putative hepatotoxins on the Concomitant Medications CRF.
- Record alcohol use on the Liver Events CRF.

The following are required for subjects with ALT ≥ 3xULN **and** bilirubin ≥ 2xULN but are optional for other abnormal liver chemistries:

- Anti-nuclear antibody, anti-smooth muscle antibody, and Type 1 anti-liver kidney microsomal antibodies.
- Liver imaging (ultrasound, magnetic resonance, or computerized tomography) to evaluate liver disease.
- The Liver Imaging and/or Liver Biopsy CRFs are also to be completed if these tests are performed.

14. STUDY CONDUCT CONSIDERATIONS

14.1. Regulatory and Ethical Considerations, Including the Informed Consent Process

GSK will obtain favorable opinion/approval to conduct the study from the appropriate regulatory agency in accordance with any applicable country-specific regulatory requirements prior to a site initiating the study in that country.

The study will be conducted in accordance with all applicable regulatory requirements, including a U.S. IND.

The study will also be conducted in accordance with "good clinical practice" (GCP), all applicable subject privacy requirements, and, the guiding principles of the 2004 Declaration of Helsinki. This includes, but is not limited to, the following:

- IRB/IEC review and favorable opinion/approval to conduct the study and of any subsequent relevant amended documents
- Written informed consent (and any amendments) to be obtained for each subject before participation in the study
- Investigator reporting requirements (e.g., reporting of AEs/SAEs/protocol deviations to IRB/IEC)

14.2. Quality Control (Study Monitoring)

In accordance with applicable regulations including GCP, and GSK procedures, GSK monitors will contact the site prior to the start of the study to review with the site staff the protocol, study requirements, and their responsibilities to satisfy regulatory, ethical, and GSK requirements. When reviewing data collection procedures, the discussion will also include identification, agreement and documentation of data items for which the CRF will serve as the source document.

GSK will monitor the study and site activity to verify that the:

- Data are authentic, accurate, and complete.
- Safety and rights of subjects are being protected.

- Study is conducted in accordance with the currently approved protocol and any other study agreements, GCP, and all applicable regulatory requirements.

The investigator and the head of the medical institution (where applicable) agrees to allow the monitor direct access to all relevant documents.

14.3. Quality Assurance

To ensure compliance with GCP and all applicable regulatory requirements, GSK may conduct a quality assurance audit. Regulatory agencies may also conduct a regulatory inspection of this study. Such audits/inspections can occur at any time during or after completion of the study. If an audit or inspection occurs, the investigator and institution agree to allow the auditor/inspector direct access to all relevant documents and to allocate his/her time and the time of his/her staff to the auditor/inspector to discuss findings and any relevant issues.

14.4. Study and Site Closure

Upon completion or premature discontinuation of the study, the monitor will conduct site closure activities with the investigator or site staff, as appropriate, in accordance with applicable regulations including GCP, and GSK procedures.

In addition, GSK reserves the right to temporarily suspend or prematurely discontinue this study at any time for reasons including, but not limited to, safety or ethical issues or severe non-compliance. For multicenter studies, this can occur at one or more or at all sites. If GSK determines such action is needed, GSK will discuss this with the investigator or the head of the medical institution (where applicable), including the reasons for taking such action. When feasible, GSK will provide advance notification to the investigator or the head of the medical institution, where applicable, of the impending action prior to it taking effect.

If the study is suspended or prematurely discontinued for safety reasons, GSK will promptly inform investigators or the head of the medical institution (where applicable) and the regulatory authorities of the suspension or premature discontinuation of the study and the reason(s) for the action. If required by applicable regulations, the investigator or the head of the medical institution (where applicable) must inform the IRB/IEC promptly and provide the reason for the suspension or premature discontinuation.

14.5. Records Retention

Following closure of the study, the investigator or the head of the medical institution (where applicable) must maintain all site study records, except for those required by local regulations to be maintained by someone else, in a safe and secure location. The records must be maintained to allow easy and timely retrieval, when needed (e.g., audit or inspection), and, whenever feasible, to allow any subsequent review of data in conjunction with assessment of the facility, supporting systems, and staff. Where permitted by local laws/regulations or institutional policy, some or all of these records can be maintained in a format other than hard copy (e.g., microfiche, scanned,

electronic); however, caution needs to be exercised before such action is taken. The investigator must assure that all reproductions are legible and are a true and accurate copy of the original, and meet accessibility and retrieval standards, including re-generating a hard copy, if required. Furthermore, the investigator must ensure there is an acceptable back-up of these reproductions and that an acceptable quality control process exists for making these reproductions.

GSK will inform the investigator of the time period for retaining these records to comply with all applicable regulatory requirements. The minimum retention time will meet the strictest standard applicable to that site for the study, as dictated by any institutional requirements or local laws or regulations, or GSK standards/procedures; otherwise, the retention period will default to 15 years.

The investigator must notify GSK of any changes in the archival arrangements, including, but not limited to, archival at an off-site facility or transfer of ownership of the records in the event the investigator leaves the site.

14.6. Provision of Study Results and Information to Investigators

When required by applicable regulations, the investigator signatory for the clinical study report will be determined at the time the report is written. When the clinical study report is completed, GSK will provide the investigator with a full summary of the study results. The investigator is encouraged to share the summary results with the subjects, as appropriate. In addition, the investigator will be given reasonable access to review the relevant statistical tables, figures, and reports and will be able to review the results for the entire study at a GSK site or other mutually agreeable location.

GSK will provide the investigator with the randomization codes for their site after the statistical analysis for the entire study has been completed.

14.7. Data Management

GSK Data Management will identify and implement the most effective data acquisition and management strategy for each clinical trial protocol and deliver datasets which support the protocol objectives. Subject data will be entered into GSK defined CRFs and combined with data provided from other sources (e.g., diary data, laboratory data) in a validated data system. Clinical data management will be performed in accordance with applicable GSK standards and data cleaning procedures with the objective of removing errors and inconsistencies in the data which would otherwise impact on the analysis and reporting objectives, or the credibility of the Clinical Study Report. Adverse events and concomitant medications terms will be coded using validated dictionaries. Original CRFs will be retained by GSK, while the investigator will retain a copy. In all cases, subject initials will not be collected nor transmitted to GSK.

15. REFERENCES

GlaxoSmithKline Document Number RH2008/00140/01 Study ID GPR111598. A study in type 2 diabetic subjects of single and multiple doses of orally administered GSK1292263 to investigate the safety, tolerability, pharmacokinetics and pharmacodynamics of the compound. Report Date 19-Jun-2009.

GlaxoSmithKline Document Number RM2006/00553/00 Study ID KG2104940. A double-blind, randomized, placebo-controlled, repeat-dose study to compare the safety, tolerability, pharmacokinetics and pharmacodynamics of GSK189075 with GW869682 in subjects with type 2 diabetes mellitus. Report Date 02-Jul-2007.

GlaxoSmithKline Document Number RM2007/00229/00 Study ID GLP106073. A single-blinded, randomized, placebo-controlled, staggered-parallel, escalating-dose study to investigate the safety, tolerability, pharmacokinetics and pharmacodynamics of subcutaneous injections of GSK716155 in subjects with type 2 diabetes mellitus. Report Date 27-Jul-2007.

GlaxoSmithKline Document Number RM2008/00434/00 Study ID GSK1292263. Investigator's Brochure. Report Date 2008.

GlaxoSmithKline Document Number RM2009/00168/00 Study ID GSK1292263. Investigator’s Brochure Supplement. Report Date May-2009.

GlaxoSmithKline Document Number RM2009/00168/01 Study ID GSK1292263. Investigator Brochure Supplement 2. Report Date 04-Jun-2009.

Herman GA, Bergman A, Stevens C, Kotey P, Yi B, et al. Effect of single oral doses of sitagliptin, a depeptidyl peptidase-4 inhibitor, on incretin and plasma glucose levels after an oral glucose tolerance test in patients with type 2 diabetes. *J Clin Endocrin Metab.* 2006;91(11):4612-9.

Januvia (sitagliptin) Product Information. October, 2007.

Mari A, Sallas WM, He YL, Watson C, Ligueros-Saylan M, et al. Vildagliptin, a dipeptidyl peptidase-IV inhibitor, improves model-assessed beta-cell function in patients with type 2 diabetes. *J Clin Endocrin Metab.* 2005;90(8):4888-94.

Appendices

Appendix 1: Liver Safety Algorithms

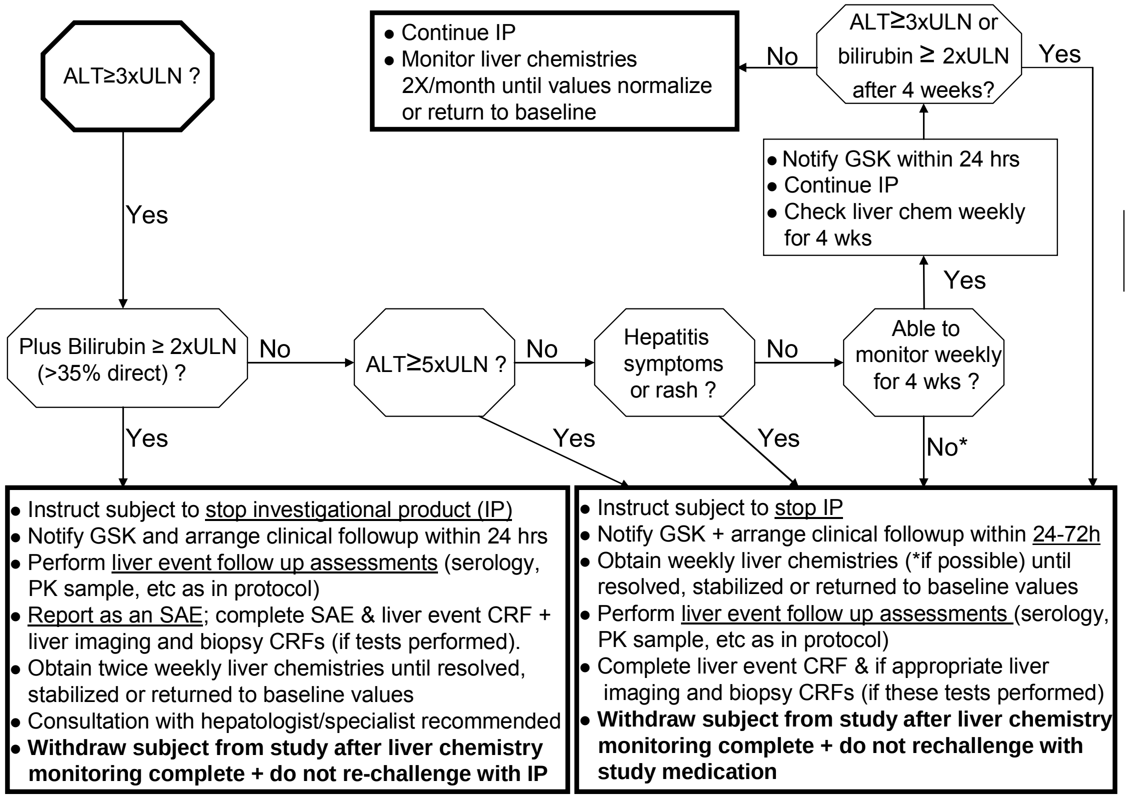

Appendix 2: Pharmacogenetic research

Pharmacogenetics - Background

Pharmacogenetics (PGx) is the study of variability in drug response due to hereditary factors in different populations. There is increasing evidence that an individual's genetic composition (i.e., genotype) may impact the pharmacokinetics (absorption, distribution, metabolism, elimination), pharmacodynamics (relationship between concentrations and pharmacologic effects or the time course of pharmacologic effects) and/or clinical outcome (in terms of efficacy and/or safety and tolerability). Collection of whole blood samples, even when no a priory hypothesis has been identified, may enable PGx analysis to be conducted if at any time it appears that there is a potential unexpected or unexplained variation in handling or response to GSK1292263.

Pharmacogenetic Research Objectives

The objective of the PGx research (if there is a potential unexpected or unexplained variation) is to investigate a possible genetic relationship to handling or response to GSK1292263. If at any time it appears there is potential variability in response in this clinical study or in a series of clinical studies with GSK1292263 that may be attributable to genetic variations of subjects, the following objectives may be investigated:

- Relationship between genetic variants and the pharmacokinetics of investigational product
- Relationship between genetic variants and safety and/or tolerability of investigational product
- Relationship between genetic variants and efficacy of investigational product

Informed Consent

Subjects who do not wish to participate in the PGx research may still participate in the clinical study. PGx informed consent must be obtained prior to any blood being taken for PGx research. Refusal to participate will involve no penalty or loss of benefits to which the subject would otherwise be entitled.

Study Population

Any subject who has given informed consent to participate in the clinical study, has met all the entry criteria for the clinical study, and receives investigational product may take part in the PGx research provided the subject has given consent to the specific collection of a PGx sample. Any subject who has received an allogeneic bone marrow transplant must be excluded from the PGx research.

Subject participation in the PGx research is voluntary and refusal to participate will not indicate withdrawal from the clinical study.

Study Assessments and Procedures

In addition to any blood samples taken for the clinical study, a whole blood sample (~10ml) will be collected for the PGx research using a tube containing EDTA. The PGx sample is labelled (or "coded") with a study specific number that can be traced or linked back to the subject by the investigator or site staff. Coded samples do not carry personal identifiers (such as name or social security number). The blood sample will be taken on a single occasion, unless a duplicate sample is required due to inability to use the original sample. It is recommended that the blood sample be taken at the first opportunity after a subject has been randomized and provided informed consent for PGx research, but the sample may be taken at any time during the subject's participation in the clinical study.

If deoxyribonucleic acid (DNA) is extracted from the blood sample, the DNA may be subjected to sample quality control analysis. This analysis will involve the genotyping of several genetic markers to confirm the integrity of individual samples. If inconsistencies are noted in the analysis, then those samples may be destroyed.

The need to conduct PGx analysis may be identified after a study (or a set of studies) of GSK1292263 has been completed and the study data reviewed. For this reason, samples may be kept for up to 15 years after the last subject completes the study or GSK may destroy the samples sooner. In special cases, the samples may not be studied, e.g., if there are not enough subjects, if the study is stopped for other reasons, or if no questions are raised about how people respond to GSK1292263. GSK or those working with GSK (for example, other researchers) will only work with samples collected from the study for the use stated in this protocol and in the informed consent form. Samples will be stored securely. Subjects can request their sample to be destroyed at any time.

Subject Withdrawal from Study

If a subject who has consented to participate in PGx research withdraws from the clinical study for any reason other than being lost to follow-up, the subject will be given a choice of one of the following options concerning the PGx sample, if already collected:

- PGx research continues per the subject's consent (i.e., the sample is retained); or,
- Any remaining sample is destroyed

If a subject withdraws consent from the PGx research or requests sample destruction, the investigator must request sample destruction by completing the appropriate documentation within the specified timeframe specified, and maintain the documentation in the site study records. In either case, GSK will only keep study information collected/generated up to that point.

Screen and Baseline Failures

If a blood sample for PGx research has been collected and it is then determined that the subject does not meet the entry criteria for participation in the clinical study, then the investigator must request sample destruction by completing the appropriate documentation within the specified timeframe, and maintain the documentation in the site study records.

Pharmacogenetics Analyses

The need to conduct PGx analysis may be identified after a study (or set of studies) has been completed. For this reason, samples may be kept for up to 15 years after the last subject completes the study. GSK may destroy the samples sooner.

Generally, GSK will utilize any of three approaches to explore genetic variation in drug response.

Specific genetic markers may be selected from “candidate genes” known to encode the drug target, drug metabolizing enzymes, molecules associated with mechanisms underlying adverse events (for example, molecules important for immune response), and those linked to drug response. Candidate genes that may be investigated in this study are genes from the GSK Absorption, Distribution, Metabolism and Excretion (ADME) panel. ADME genes play a central role in drug pharmacokinetics and pharmacodynamics (PK-PD). The GSK ADME panel contains genetic markers from one hundred and thirty-five enzymes, transporters and other genes involved in drug absorption, distribution, metabolism and excretion. The ADME panel may be used to investigate the relationship between genetic variants on the panel and pharmacokinetics, safety and efficacy of the investigational product.

Additional candidate genes that may be investigated in this study include, but are not limited to, the following:

- GPR119 receptor

- Genes altering response to DPP-IV inhibitors or GLP-1 analogues

In addition, continuing research may identify other enzymes, transporters, proteins, or receptors that may be involved in response to GSK1292263. The genes that may code for these proteins may also be studied.

Evaluate markers that comprise pre-defined “panels” for association with specified endpoints.

Examples of such panels include the GSK ADME (Absorption, Distribution, Metabolism, and Excretion) Panel and the GSK DILI (Drug Induced Liver Injury) Panel which consist of genetic markers from set of genes that are known to be related to pharmacokinetic, pharmacodynamic, immune, or adverse drug response.

Evaluate markers throughout the genome using a whole genome screen (WGS).

By evaluating large numbers of genetic markers (e.g., single nucleotide polymorphisms or SNPs) throughout the genome, sets of markers may be identified that correspond to differential drug response.

In all cases, appropriate statistical methods will be used to analyze the genetic markers in the context of other clinical data. The statistical methods for analysis may include, but are not limited to Hardy-Weinberg Equilibrium (HWE) Analysis, Linkage Disequilibrium Analysis, Evaluation of Genotypic Effects, Evaluation of Treatment by Genotype and Gene-Gene Interaction, Multiple Comparisons and Multiplicity, and/or Power and Sample Size Considerations. Detailed description of all analyses to be conducted will be documented in the Reporting and Analysis Plan.

Provision of Study Results and Confidentiality of Subject's PGx Data

GSK may summarize the cumulative PGx research results in the clinical study report.

In general, GSK will not inform the investigator, subject, or anyone else (e.g., family members, study investigators, primary care physicians, insurers, or employers) of the PGx research results under any circumstances unless required by law. This is because the information generated from PGx studies is preliminary in nature, and the significance and scientific validity of the results are undetermined at such an early stage of research.

Appendix 3: Hunger, Craving, and Fullness Questionnaire

	CONFIDENTIAL		Page 1
Protocol Identifier GPR111598	Subject Identifier		Visit Description Day __________

Hunger, Craving, and Fullness Questionnaire

This questionnaire asks you how often you were hungry, craved food, and about how full you feel when you finished meals, on average, **in the past 24-hours.**

For each question below, place a cross (✕) in the box next to the option that best describes your answer. Place a cross in only one box per question.

In the past 24 hours I was hungry

Always ☐

Often ☐

Sometimes ☐

Rarely ☐

Never ☐

In the past 24 hours I thought about food

Always ☐

Often ☐

Sometimes ☐

Rarely ☐

Never ☐

	CONFIDENTIAL		Page 2
Protocol Identifier GPR111598	Subject Identifier		Visit Description Day __________

In the past 24 hours I wanted to eat

Always ☐

Often ☐

Sometimes ☐

Rarely ☐

Never ☐

In the past 24 hours I ate more than I think I should have.

Yes definitely ☐

Yes probably ☐

I don’t know ☐

Probably not ☐

Definitely not ☐

In the past 24 hours I craved specific foods

Always ☐

Often ☐

Sometimes ☐

Rarely ☐

Never ☐

	CONFIDENTIAL		Page 3
Protocol Identifier GPR111598	Subject Identifier		Visit Description Day __________

In the past 24 hours, when I finished my meals I felt

Much too full ☐

Very full ☐

Comfortably full ☐

Slightly hungry ☐

Very hungry ☐

In the past 24 hours, when I finished my meals I felt

Extremely satisfied ☐

Satisfied ☐

Neutral ☐

Dissatisfied ☐

Extremely dissatisfied ☐

Appendix 4: Protocol Amendment Changes

AMENDMENT 4

Where the Amendment Applies

Amendment applies to all sites.

Summary of Amendment Changes with Rationale

This amendment includes preliminary information from the 13-week toxicology studies in the rat and dog and provides preliminary pharmacokinetic data from the ongoing repeat dose (Part C) of the study.

List of Specific Changes

ABBREVIATIONS

ADDED TEXT

GLDH Glutamate dehydrogenase

Section 1.2. Previous Human Experience

PREVIOUS TEXT

1.2. Previous Human Experience

REVISED TEXT

1.2. ~~**Previous**~~ Human Experience **with GSK1292263**

Section 1.2. Human Experience with GSK1292263

ADDED TEXT

1.2.2. GPR111598 - First in T2DM subjects: Preliminary Safety and PK Data from Parts A and B

- **Part A (completed): a 5-way crossover evaluating single doses of 25, 150 and 800mg of GSK1292263, placebo and open-label sitagliptin 100mg. Subjects were administered a 75g oral glucose tolerance test 2 hours after dosing.**
- **Part B (completed): a 2-way crossover evaluating a single dose of 800mg GSK1292263 administered in the fed and fasting state.**

It is important to note that the investigators are still blinded to the treatment allocation in this study, and only preliminary results without treatment allocation are presented below.

1.2.2.2 Part A

Safety

In Part A, 12 T2DM subjects received singles doses of 25mg and 150mg GSK1292263, and 11 received 800mg single doses of GSK1292263 (the twelfth subject was withdrawn before completing the 800mg period; see below). These doses of GSK1292263 were safe and well tolerated.

Points of note:

- **There was no association of study drug to significant AEs (see Table 1).**
- **There was 1 AE of asymptomatic hypoglycemia several hours after lunch on glucometer testing (48mg/dL) that was not observed on repeat laboratory testing.**
- **There were episodes of blurred vision and dizziness that were not associated with hypoglycemia and in some cases related to rapid glucose changes during the OGTT.**
- **There were no clinically significant changes in vital signs.**
- **There were no clinically significant ECG or telemetry changes, including QTc.**
- **There were no clinically significant safety lab changes, other than increased blood glucose levels typical of T2DM subjects and one instance of elevated Mg^{2+} that was considered to be a laboratory error.**

One subject was withdrawn from the study in Period 1 because of severe hyperglycemia during Day 1 after receiving sitagliptin 100mg. On further questioning it was discerned that this subject was not drug naïve, as required for enrolment into the study, and had taken oral anti-diabetic medications and insulin in the past. This subject was replaced.

Another subject was withdrawn on D-1 prior to dosing in Period 4 because of a positive cotinine test and high fasting blood glucose. The subject had completed Periods 1-3. This subject was not replaced.

Table 1 Most Frequent Adverse Events For GPR111598 (Part A)

	Single Dose		
Most Frequent Adverse Events	Placebo N=11	Sitagliptin N=12	GSK1292263 N=12
	n (%)		
Any AE	2 (18%)	2 (17%)	2 (17%)
Any AE related to investigational product	1 (9%)	0	1 (9%)
Most Common AEs:			
Headache	2 (18%)	0	2 (17%)
Blurry Vision	0	1 (9%)	2 (17%)
Hyperglycemia	0	1 (9%)	0

Pharmacokinetics

Table 2 and Table 3 show the preliminary AUC and Cmax PK data for GSK1292263 after singles doses of 25, 150 and 800mg administered in the fasted state 2h before an OGTT. Note that complete PK profiles are available from 12 subjects who received 25mg and 150mg and from 11 subjects who received 800mg.

Table 2 Preliminary AUC(0-24) following single doses ofGSK1292263 (Fasted)

Dose (mg)	AUC(0-24h), ng.h/mL (CV%)		Exposure ratio relative to mean exposure at NOAEL for 14-day toxicity study [1]	Exposure ratio relative to individual exposure at NOAEL for 14-day toxicity study [2]
25	537 (20)		92	101
150	1748 (32)		28	24
800	4109 (25)		12	13

1. Mean AUC(0-24h) limit = 49400 ng.h/mL
2. Individual AUC(0-24h) limit = 72700 ng.h/mL – Based on maximum individual exposure

Table 3 Preliminary Cmax following single doses of GSK1292263 (Fasted)

Dose (mg)	Cmax, ng/mL (CV%)		Exposure ratio relative to mean exposure at NOAEL for 14-day toxicity study [1]	Exposure ratio relative to individual exposure at NOAEL for 14-day toxicity study [2]
25	53 (17)		51	50
150	175 (32)		15	13
800	393 (27)		7	6

1. Mean Cmax limit = 2693 ng/mL
2. Individual Cmax limit = 3585 ng/mL – Based on maximum individual exposure

1.2.2.3. Part B

The four T2DM subjects in Part B received 800mg in the fasted and fed state. Three subjects were drug naïve, and the fourth was washed off metformin.

Safety

Points of note:

- **There was no association of study drug to significant AEs.**
- **There were no clinically significant changes in vital signs.**
- **There were no clinically significant ECG or telemetry changes, including QTc, except for 1 short episode of Wenckebach AV block that occurred at ~3am and resulted in 1 dropped QRS complex.**

- **There were no clinically significant safety lab changes, other than increased blood glucose levels typical of T2DM.**

Pharmacokinetics

Table 4 and Table 5 summarize the preliminary AUC and Cmax PK data for GSK1292263 after single doses of 800 mg in fasted and fed states. Note that complete PK profiles are available from all 4 subjects in this period. Across subjects, GSK1292263 AUC(0-24h) increased by 2.6- to 4.5-fold in the fed state and Cmax increased 1.3- to 4.7-fold in the fed state. Mean AUC(0-24h) and Cmax increased 3.8- and 3.1-fold, respectively, in the fed state.

Table 4 Preliminary AUC(0-24) following single doses of 800mg GSK1292263 (Fed and Fasted)

Dose (mg)	Meal	AUC(0-24h), ng.h/mL (CV%)	Exposure ratio relative to mean exposure at NOAEL for 14-day toxicity study [1]	Exposure ratio relative to individual exposure at NOAEL for 14-day toxicity study [2]
800	Fasted	3429 (22)	14	17
800	Fed	12997 (29)	4	4

1. Mean AUC(0-24h) limit = 49400 ng.h/mL
2. Individual AUC(0-24h) limit = 72700 ng.h/mL – Based on maximum individual exposure

Table 5 Preliminary Cmax following single doses of 800mg GSK1292263 (Fed and Fasted)

Dose (mg)	Meal	Cmax, ng/mL (CV%)	Exposure ratio relative to mean exposure at NOAEL for 14-day toxicity study [1]	Exposure ratio relative to individual exposure at NOAEL for 14-day toxicity study [2]
800	Fasted	351 (27)	8	8
800	Fed	1009 (37)	3	3

1. Mean Cmax limit = 2693 ng/mL
2. Individual AUC(0-24h) limit = 3585 ng/mL – Based on maximum individual exposure

1.2.2.3. Doses of GSK1292263 selected for evaluation in Part C

- **Based on the safety, tolerability and PK from Parts A and B, the actual doses of GSK1292263 being administered in Part C are: 50mg BID, 150mg BID, 300mg BID and 600mg QD.**

Section 1.2.1.2. Safety Overview ~~(Data Currently Blinded to Principal Investigator)~~

Section 1.4.1. Study Rationale

PREVIOUS TEXT

The maximum allowable total daily dose in the current study is 800mg administered as single or divided doses because the drug substance used in this study may contain a trichloromethyloxadiazole process impurity, GSK2116107A, which was mutagenic in a mini-Ames test (See Section 1.5.2).

REVISED TEXT

While ~~T~~the maximum allowable total daily dose in the current study **was** ~~is~~ 800mg **based on** ~~administered as single or divided doses because~~ the drug substance used in this study that may contain a trichloromethyloxadiazole process impurity, GSK2116107A, which was mutagenic in a mini-Ames test **(Amendment No. 2), Amendment No. 4 sets the new maximum total daily dosage for Part C in this protocol at 600mg based on the preliminary data from the 13 week toxicology study in the dog.** (See Section 1.5.2).

Section 1.4.2.2. Predicted ~~Maximal~~ Maximum Expsoures Relative to Exposure Limits

PREVIOUS TEXT

Based on the results of non-clinical toxicology studies See Investigator Brochure [GlaxoSmithKline Document Number RM2008/00434/00], exposures in the current study will not exceed a mean steady-state AUC(0-24h) of 49,400ng.h/mL and mean Cmax of 2693ng/mL which are 80% of the gender-averaged no-observed-adverse-effect-level (NOAEL) mean exposure in beagle dogs at Day 14 at the highest dose of 1000mg/kg/day. Furthermore, no individual will exceed an AUC(0-24h) of 72,700ng.h/mL or Cmax of 3585ng/mL, which are 80% of the highest AUC and Cmax observed in dog at 1000mg/kg NOAEL dose group. These exposures are consistent with limits that were approved for the conduct of the FTIH study, GPR111956, by the GSK Global Safety Board and the local IRB, and were not objected to by the FDA

REVISED TEXT

Based on the results of non-clinical toxicology studies See Investigator Brochure [GlaxoSmithKline Document Number RM2008/00434/00], exposures in the current study **were** ~~will~~ **not to** exceed a mean steady-state AUC(0-24h) of 49,400ng.h/mL and mean Cmax of 2693ng/mL which are 80% of the gender-averaged no-observed-adverse-effect-level (NOAEL) mean exposure in beagle dogs at Day 14 at the highest dose of 1000mg/kg/day. Furthermore, no individual **was to** ~~will~~ exceed an AUC(0-24h) of 72,700ng.h/mL or Cmax of 3585ng/mL, which are 80% of the highest AUC and Cmax observed in dog at 1000mg/kg NOAEL dose group **in the 14-day toxicology study**. These exposures **were** ~~are~~ consistent with limits that were approved for the conduct of

the FTIH study, GPR111956, by the GSK Global Safety Board and the local IRB, and were not objected to by the FDA.

ADDED TEXT

In this Amendment No. 4, preliminary data from the 13 week toxicology study in the dog are summarized in Section 1.5.2.1 and Section 1.5.2.3. Briefly, three of four males given 1000 mg/kg/day and one male given 20 mg/kg/day had minimal to mild degeneration/depletion of seminiferous epithelium in the testes. The AUC(0-24h) and Cmax associated with the no effect threshold for testicular findings were 36772 ng.h/mL and 2537ng/mL, respectively following 13 weeks of dosing. The lowest individual AUC(0-24h) and Cmax for the dog testicular effect were 48,863 ng.h/mL and 2757ng/mL, respectively, following 13 weeks of dosing.

Adverse GSK1292263-related changes were also observed in the liver. Moderate to marked increases in ALT (3.3X to 11.3X in males, 4.3X to 11.7X in females based on individual animal values relative to pretest) and marked increases in GLDH (4.4X to 22.3X in males, 5.8X to 15X in females based on individual animal values relative to pretest) were observed at 1000 mg/kg/day. There was no hepatocellular necrosis evident in any of the dogs given 1000 mg/kg/day so there was no clear histopathology correlate explaining the ALT and GLDH elevations. While the maximum allowable total daily dose in the current study was 800mg based on the potential impurity in the drug substance (Amendment No. 2; see Section 1.5.2), Amendment No. 4 sets the new maximum total daily dosage for Part C in this protocol at 600mg based on the preliminary data from the 13 week toxicology study in the dog. A dose of 300 mg BID, or a dosing regimen resulting in equivalent exposure, given for up to 14 days does not pose a significant clinical risk from the perspective of either mean or individual exposures to date.

PREVIOUS TEXT

The maximum allowable total daily dose in the current study is 800mg administered as single or divided doses (See Section 1.5.2). Modelling and simulation was performed to project maximum exposures for various scenarios following total daily doses of 800mg.

REVISED TEXT

While ~~**T**~~**t**he maximum allowable total daily dose in the current study **was** ~~**is**~~ 800mg ~~**administered as single or divided doses**~~ **based on the potential impurity in the drug substance** (See Section 1.5.2), ~~**Modelling and simulation was performed to project maximum exposures for various scenarios following total daily doses of 800mg.**~~ The new maximum total daily dosage for Part C in this protocol is set at 600mg based on the preliminary data from the 13 week toxicology study in the dog.

ADDED TEXT

Amendment No. 4 Note: For both of the following simulation Scenarios, please refer to Section 1.4.2.4 and Section 1.5.2.3 for preliminary exposure data from Part C of this study and the 13 week toxicology studies in the rat and dog.

… Further, no individual is predicted to exceed the individual AUC(0-24) ~~**limit**~~ of 72,700ng.h/mL, which is 80% of the highest AUC and Cmax observed in dog at 1000mg/kg NOAEL dose group **in the 14 day toxicology study.** . Following administration of 400mg BID at steady-state a small percentage of subjects (~2%) are predicted to slightly exceed the individual Cmax ~~**limit**~~ of 3585 ng/mL.

Section 1.4.2.3. Preliminary Doses and Predicted Exposures

PREVIOUS TEXT

Table 6

Fold Cover Relative to NOAEL Limits[2,3]

REVISED TEXT

Exposure ratio relative to exposure at NOAEL for 14-day toxicity study [2,3]

PREVIOUS TEXT

Table 7

Fold Cover Relative to NOAEL Limits[2,3]

REVISED TEXT

Exposure ratio relative to exposure at NOAEL for 14-day toxicity study [2,3]

PREVIOUS TEXT

Any adjustments to doses in Part C will not exceed 800mg once daily, which is predicted to maintain exposures below non-clinical toxicology limits as summarized in Table 8.

REVISED TEXT

Based on the preliminary toxicokinetic data from the 13 week toxicology study in the dog (Section 1.5.2.3), any adjustments to **QD** doses in Part C will not exceed ~~**800mg** once daily~~ **600mg total daily dose, or a dosing regimen resulting in equivalent exposure.**~~**as summarizedf in Table 8 (for the 14 day toxicology studies) and Table 11 (for the 13 week toxicology studies).**~~

PREVIOUS TEXT

Table 8

Fold Cover Relative to NOAEL Limits[2,3]

REVISED TEXT

Exposure ratio relative to exposure at NOAEL for 14-day toxicity study [2,3]

ADDED TEXT

Part C - Twice-Daily (BID) Dosing (Cohort 3 or 4) **(See Section 1.4.2.4 for Preliminary Exposure Data from Part C)**

PREVIOUS TEXT

The planned maximum dose level for twice daily dosing is 400mg BID (total daily dose – 800mg). However, based on safety, tolerability and PK/PD analysis from Part A, the BID dose level may be reduced.

REVISED TEXT

Based on the preliminary data from the 13 week toxicology study in the dog, ~~T~~**t**he maximum dose level for twice daily dosing is 300mg BID (total daily dose – ~~8~~**600mg**). However, based on safety, tolerability and PK/PD analysis from Part A**, B or C,** the BID dose level may be reduced. **A dose of 300mg BID, or a dosing regimen resulting in equivalent exposure, given for up to 14 days does not pose a significant clinical risk from the perspective of either mean or individual exposures to date [see Table 8 (PK data from Part C) and Table 11 (PK for the 13-week toxicology studies)].**

ADDED TEXT

Part C – Once Daily (QD) Dosing **(See Section 1.4.2.4 for Preliminary Exposure Data from Part C)**

ADDED TEXT

Predicted steady-state exposures following a dose of 400mg BID for 14 days in the fed state are summarized in Table 9. For co-administration of GSK1292263 with sitagliptin on Day 14 it is assumed that steady-state AUC(0-24h) and Cmax will increase by ~56% and ~35%, respectively, compared to GSK1292263 alone on Day 13. These assumptions are based on increases in exposure observed in the FTIH study following single-dose co-administration of GSK1292263 and sitagliptin. Predicted co-dosing exposures are conservative (highest exposure) given that GSK1292263 and sitagliptin will only be administered as a single dose on Day 14 after GSK1292263 has been administered alone for 13 days. It should be noted that GSK1292263 did not affect the pharmacokinetics of sitagliptin in the FTIH study. All exposures are predicted to remain below the group mean exposure limits based on non-clinical toxicology data. No subjects are predicted to exceed the individual exposure limits based on non-clinical **14 day** toxicology data **(see Section 1.4.2.4 and Section 1.5.2.3 for preliminary exposure data from Part C and the 13 week toxicology studies in the rat and dog).**

PREVIOUS TEXT

Based on emergent data from Parts A and B, BID dosing of GSK1292263 may be investigated in Cohort 3 by administering the doses of GSK1292263 or placebo as a BID regimen, keeping the exposure limits outlined above. In this case Cohort 4 will evaluate a QD dose of GSK1292263 that will not exceed 800mg, and will match the total daily dose of the highest BID regimen in Cohort 3.

REVISED TEXT

Based on emergent data from Parts A and B, BID dosing of GSK1292263 may be investigated in Cohort 3 by administering the doses of GSK1292263 or placebo as a BID regimen, keeping the **dose and** exposure limits outlined above. In this case Cohort 4 will evaluate a QD dose of GSK1292263 that will ~~**not exceed 800mg, and will**~~ match the total daily dose of the highest BID regimen in Cohort 3.

PREVIOUS TEXT

Table 9

Fold Cover Relative to NOAEL Limits[2,3]

REVISED TEXT

Exposure ratio relative to exposure at NOAEL for 14-day toxicity study [2,3]

Section 1.4.2. Dose Rationale

ADDED TEXT

1.4.2.4. Preliminary Human Exposures from the On-Going Part C and the toxicokinetics from the 13 Week Toxicology Study in the Dog

In the 13-week toxicology study in the dog, three of four males given 1000 mg/kg/day and one male given 20 mg/kg/day had minimal to mild degeneration/depletion of seminiferous epithelium in the testes. The AUC(0-24h) and Cmax values in an individual animal associated with the no effect threshold for testicular findings were 36772 ng.h/mL and 2537ng/mL, respectively. The lowest individual AUC(0-24h) and Cmax for the dog testicular effect were 48,863 ng.h/mL and 2757ng/mL, respectively.

The maximum GSK1292263 exposures observed in the current study have been associated with steady-state during Part C. The final doses of GSK1292263 selected in Part C were 50 mg BID, 150 mg BID, 300 mg BID and 600 mg QD. A summary of preliminary exposures of GSK1292263 (n=6) at steady-state (Day 13 and Day 14) are presented in Table 10.

Table 10. Summary of Preliminary GSK1292263 Exposure at Steady-State in Part C

Dose	Day 13		Day 14	
	AUC(0-24h) (ng.h/mL) Mean ±SD [min, max]	Cmax (ng/mL) Mean ±SD [min,max]	AUC(0-24h) (ng.h/mL) Mean ±SD [min, max]	Cmax (ng/mL) Mean ±SD [min, max]
50 mg BID	7944 ± 1362 [5357, 9203]	429 ± 58 [396, 536]	8153 ± 1240 [5758, 9331]	452 ± 53 [369, 515]
150 mg BID	16986 ± 5863 [12449, 27137]	985 ± 312 [691, 1517]	17257 ± 5656 [12789, 26895]	952 ± 304 [717, 1479]
300 mg BID	23101 ± 7900 [15606, 35959]	1258 ± 376 [912, 1885]	23810 ± 8517 [15585, 38771]	1222 ± 343 [863, 1834]
600 mg QD	9330 ± 1847 [6555, 11964]	866 ± 145 [653, 1098]	10221 ± 2519 [7333, 13870]	795 ± 207 [601, 1221]

The maximum observed group mean AUC(0-24h) and Cmax values to date are 23810 and 1258 ng/mL, respectively. Of note, (i) the highest exposures have been observed in the 300mg BID group, and (ii) there is no significant effect of sitagliptin on GSK1292263 exposures in these T2DM subjects.

Section 1.4.3. Stopping Criteria

PREVIOUS TEXT

The target range of exposures for this study has been selected to allow for a robust characterization of safety, tolerability, PK and PD of GSK1292263 in T2DM volunteers. The highest daily dose of GSK1292263 to be administered in the study will be 800mg (administered as single or divided doses).

REVISED TEXT

The target range of exposures for this study has been selected to allow for a robust characterization of safety, tolerability, PK and PD of GSK1292263 in T2DM volunteers. **Based on the preliminary data from the 13 week toxicology study in the dog, t**~~T~~he highest **total** daily dose of GSK1292263 to be administered in the study will be ~~8~~**6**00mg (administered as single or divided doses). **A dose of 300 mg BID, or a dosing regimen resulting in equivalent exposure, given for up to 14 days does not pose a significant clinical risk from the perspective of either mean or individual exposures to date.**

Section 1.5.2. Risks Related to GSK1292263

PREVIOUS TEXT

As indicated in Supplement 2 of the IB ([GlaxoSmithKline Document Number RM2009/00168/01]), the maximum allowable total daily dose in the current study is 800mg (administered as single or divided doses) because the drug substance used in this study may contain a trichloromethyloxadiazole process impurity, GSK2116107A, which was mutagenic in a mini-Ames test. Quantitative assessments indicate that GSK2116107A is not present in the final drug substance at a limit of detection of 50 ppm (which based on a clinical dose of 800 mg/day, equates to a total oral dose of less than 40 µg/day). There are no safety concerns for the conduct of a clinical trial at doses up to 800 mg/day for up to 28 days because the level of this impurity is below the staged TTC (Threshold of Toxicological Concern) limit of 60 µg/day for clinical trials of up to one month duration.

REVISED TEXT

As indicated in Supplement 2 of the IB ([GlaxoSmithKline Document Number RM2009/00168/01]), the maximum allowable total daily dose in the current study is 800mg (administered as single or divided doses) **based on the possibility that** **~~because~~** the drug substance used in this study may contain a trichloromethyloxadiazole process impurity, GSK2116107A, which was mutagenic in a mini-Ames test. Quantitative assessments indicate that GSK2116107A is not present in the final drug substance at a limit of detection of 50 ppm (which based on a clinical dose of 800 mg/day, equates to a total oral dose of less than 40 µg/day). There are no safety concerns for the conduct of a clinical trial at doses up to 800 mg/day for up to 28 days because the level of this impurity is below the staged TTC (Threshold of Toxicological Concern) limit of 60 µg/day for clinical trials of up to one month duration. **Amendment No. 4, however, limits the maximum total daily dose allowed in the current study to 600mg based on the preliminary data from the 13 week toxicology study in the dog (Section 1.5.2).**

ADDED TEXT

- The likelihood of the plasma exposures exceeding NOAEL limits is small based on FTIH **and preliminary Part C** PK data (see Section 1.4.2 **and Section 1.5.2**).

REVISED TEXT

As indicated in Supplement 2 of the IB ([GlaxoSmithKline Document Number RM2009/00168/01]), **Amendment No. 2 set** the maximum allowable total daily dose in the current study **is ~~at~~** 800mg …

ADDED TEXT

- The likelihood of the plasma exposures exceeding NOAEL limits is small based on FTIH and preliminary Part**s** A**, B and C** PK data **from the current study** (see Section 1.4.2 and Section 1.5.2.

Section 1.5.2.3. Toxicokinetic Parameters from the 13-week Toxicology Studies in the Rat and Dog

DELETED TEXT

~~Preliminary 14 day Phase IIa clinical exposure (GPR111598)~~		
~~Dose~~	~~C_{max} (ng/mL)~~	~~AUC_{0-24} (ng.h/mL)~~
~~300 mg BID*~~	~~1222~~	~~23,810 (15584-38771)~~

~~Note: *based on data from 6 of 12 subjects scheduled to be enrolled.~~

ADDED SECTION

Section 1.5.2.4. Risk assessment based on the preliminary observations from the 13 week toxicology studies in rats and dogs

There were no treatment-related effects in liver or testes in the rat and dog up to 2000 mg/kg/day and 1000 mg/kg/day GSK1292263, respectively, at systemic exposures of 58,405 ng·hr/mL (male rats) and 49,505 ng·hr/mL (male dogs) on Day 14. In addition, stage-dependent qualitative evaluation of spermatogenesis demonstrated normal progression up to the limit dose in both species indicating there were no subtle morphological changes in the testes following 14 days of dosing. In the 13-week rat study (10, 150, 2000 mg/kg/day) there were no treatment-related findings in liver or testes up to 43,551 ng·hr/mL (mean male rat AUC0-24hr).

For single dose administration of GSK1292263 in clinical subjects, preclinical data support mean clinical exposures up to 49,400 ng·hr/mL (as described in the current Investigator Brochure, [GlaxoSmithKline Document Number RM2008/00434/00]).

<u>Testis</u>: In the ongoing clinical trials there is no means to readily monitor for the testicular effect observed in the 13-week dog study. The mean clinical AUC achieved at 300 mg BID (23,810 ng.h/mL), the highest intended dose, is below the no-effect AUC for the testicular effect in any dog in the 3-month study (36,772 ng.h/mL), and is less than 50% of the mean no-effect AUC in the dog 14-day study (49,505 ng.h/mL).

The highest individual clinical AUC achieved at 300 mg BID (38,771 ng.h/mL) is well below the highest exposure achieved in an individual dog at the NOAEL following 14 days of dosing (78,414 ng·hr/mL), and is also less than the lowest individual dog AUC for a testicular effect following 3 months of dosing (48,863 ng.h/mL). Although 38,771 ng.mL does exceed the highest individual no-effect exposure in the 3-month dog study (36,772 ng.h/mL), the dog testicular change is

regarded as a time-dependant effect and the intended clinical dose duration is 1/6 of the duration that caused testicular toxicity (14 versus 90 days) in a single species.

<u>Liver</u>: There are safety margins based on systemic exposure (AUC) for the liver effects observed in the 13-week dog study (4.5-fold at the effect dose of 1000 mg/kg/day; 1.7-fold at the no-effect dose of 20 mg/kg/day, compared to the clinical AUC at 300 mg BID) (Table 11). Importantly, liver effects are readily monitorable and liver function monitoring and withdrawal criteria are already in place in this study.

In summary, the doses of GSK1292263 being evaluated in Part C of this study (top BID dose limited to 300 mg BID , or a dosing regimen resulting in equivalent exposure) do not pose a significant clinical risk from the perspective of either mean or individual exposures to date.

Section 4.1.3. Part C: Repeat Dosing (Cohorts 3 and 4)

PREVIOUS TEXT

If GSK1292263 is dosed QD in Cohort 3 of Part C, the planned dosed are 25, 150, 800mg QD. In the event of a dose adjustment, the maximum dose to be administered QD will not exceed 800mg. Cohort 4 will evaluate half the maximal QD dose used in Cohort 3 administered twice a day for 14 days.

If Cohort 3 of Part C is dosed BID, then the planned doses will not exceed 400mg BID (total daily dose of 800mg), and Cohort 4 will evaluate the maximum total daily dose used in Part C administered QD for 14 days.

REVISED TEXT

If GSK1292263 is dosed QD in Cohort 3 of Part C, the planned dosed are 25, 150, 800mg QD. In the event of a dose adjustment, the maximum **total daily** dose to be administered ~~**QD**~~ will not exceed ~~**8**~~**6**00mg. Cohort 4 will evaluate half the maximal QD dose used in Cohort 3 administered twice a day for 14 days.

If Cohort 3 of Part C is dosed BID, then the planned doses will not exceed **43**00mg BID (total daily dose of ~~**8**~~**6**00mg), and Cohort 4 will evaluate the maximum total daily dose used in Part C administered QD for 14 days.

REVISED TEXT

… doses will not exceed **43**00mg BID, and Cohort 4 will evaluate the maximum total daily dose used in Part C administered QD for 14 days.

ADDED TEXT

Amendment No 4 Note: The actual doses being administered in Part C are: 50mg BID, 150mg BID, 300mg BID and 600mg QD.

Section 4.1.6.3. Part C

ADDED TEXT

Amendment No 4 Note: The actual doses being administered in Part C are: 50mg BID, 150mg BID, 300mg BID and 600mg QD.

Section 4.4. Dose Adjustment/Stopping Criteria

ADDED TEXT

This protocol allows some alteration from the currently outlined dosing schedule. No dose will be administered that is projected to exceed: (i) for the cohort as a whole, a mean plasma AUC(0-24) 49,400ng.h/mL and mean Cmax of 2693ng/mL, which is 80% of the gender-averaged NOAEL mean exposure in beagle dogs at Day 14 at the highest dose of 1000mg/kg/day, or (ii) for an individual subject an AUC of 72,700ng.h/mL and Cmax of 3585ng/mL, which are 80% of the highest AUC and Cmax observed in dog at 1000mg/kg NOAEL dose group **in the 14 day toxicology study**. These exposures limits were approved for the conduct of the FTIH study, GPR111596, by the GSK Global Safety Board and the local IRB, and were not objected to by the FDA.

In Amendment No. 4, however, the maximum total daily dose that will be evaluated in Part C of this study is 600mg based on preliminary data from the 13 week toxicology study in the dog (see Section 1.5.2).

REVISED TEXT

…Alternatively, GSK1292263 may be administered BID in 3 treatment arms at doses that do not exceed ~~**4**~~**3**00mg BID…

ADDED TEXT

Doses in Part C (Cohorts 3 and 4) will be confirmed or changed based on safety/tolerability data (including clinically significant abnormalities of clinical labs, vital signs, and ECGs), and PK and PD data from Part**s** A**, B and C**.

Amendment No 4 Note: The actual doses being administered in Part C are: 50mg BID, 150mg BID, 300mg BID and 600mg QD.

REVISED TEXT

… so as not to exceed ~~**8**~~**6**00mg…

Section 5.1. Number of Subjects

ADDED TEXT

Amendment No 4 Note: The actual doses being administered in Part C are: 50mg BID, 150mg BID, 300mg BID and 600mg QD.

REVISED TEXT

> Note: For subjects that are being screened for Part C, all doses of anti-diabetic medication must have been stable for at least ~~**3**~~ 1 month**s** prior to Screening, and the subject must be willing to wash out from their anti-diabetic medications from Day -7 through Day 15.

ADDED TEXT

Amendment No 4 Note: The actual doses being administered in Part C are: 50mg BID, 150mg BID, 300mg BID and 600mg QD.

Section 5.2.2. Exclusion Criteria

ADDED TEXT

*Note that if ECG abnormalities are identified **that may be variable or reversible**, the ECG should be repeated two more times (with 5 minutes between ECG readings) and the average of the 3 values used to determine eligibility.

AMENDMENT 3

Where the Amendment Applies

Amendment applies to all sites.

Summary of Amendment Changes with Rationale

This amendment allows for venous blood or blood plasma glucose for glucose monitoring and selection criteria, clarifies that Part C subjects can only be on monotherapy, clarifies timing of HCFQ, clarifies that glycerol is a PD marker, updates language regarding the central randomization system., updates the T&E Table for Part C, clarifies inclusion of non-childbearing potential women, expands the BMI range, clarifies creatinine clearance, adds details on GI surgery inclusion criterion, changes pre-dose ECGs in Part 3 from single to triplicate on specified days, clarifies that telemetry was only in Parts A and B, and adds some details around meals and dosing in Part C.

List of Specific Changes

Section 1.4.2.3. Planned Doses and Predicted Exposures

ADDED TEXT

Part C – **Once** Daily **(QD)** Dosing

ADDED TEXT

Based on emergent data from Parts A and B, BID dosing of GSK1292263 may be investigated in Cohort 3 by administering the doses of GSK1292263 or placebo as a BID regimen, keeping the exposure limits outlined above. In this case Cohort 4 will evaluate a QD dose of GSK1292263 that will not exceed 800mg**, and will match the total daily dose of the highest BID regimen in Cohort 3.**

Section 1.5.1. Risks Related to Washout of Anti-Diabetic Medications

PREVIOUS TEXT

The risk that glycemic control will deteriorate in Part C is partially mitigated by the fact that one cohort of subjects will receive sitagliptin at the dose approved for the treatment of T2DM (100mg)

REVISED TEXT

The risk that glycemic control will deteriorate in Part C is partially mitigated by the fact that one **group** ~~cohort~~ of subjects will receive sitagliptin at the dose approved for the treatment of T2DM (100mg)

Section 1.5.1. Risks Related to Washout of Anti-Diabetic Medciations

ADDED TEXT

Glucose levels will be monitored by capillary blood glucose (CBG) **or venous or blood plasma glucose** at least twice daily (fasting and pre-evening meal) when the subject is in the unit and during washout of anti-diabetic medications prior to dosing a study drug. In addition, at the blood draws after dosing in Parts A and B:

Section 1.5.3. Capillary Blood Glucose (CBG) Monitoring

ADDED TEXT

While in-clinic, fasting glucose and pre-evening meal glucose will be monitored daily by CBG**, venous blood** or plasma glucose.

Section 4.1.3. Part C: Repeat Dosing (Cohorts 3 and 4)

REVISED TEXT

~~T2DM subjects on monotherapy or sub-maximal anti-diabetic medications will wash-off these medications for 1 week prior to receiving study drug.~~ Part C (Cohorts 3 and 4) will be conducted to assess safety, tolerability, PK and PD of GSK1292263 and open-label sitagliptin in T2DM subjects after 14-days of dosing, as well as the safety, tolerability, PK and PD of (i) a 100mg single dose of open-label sitagliptin co-administered with GSK1292263 or placebo (placebo arm), and (ii) sitagliptin alone (sitagliptin arm) on Day 14.

Section 4.1.6.3. Treatment Regimen Part C

REVISED TEXT

Subjects will be randomized to 14 days of dosing with three dose levels of GSK1292263 (planned QD doses: 25, 150, ~~400mg~~ **800mg**; planned BID doses not to exceed 400mg BID) or matching placebo, or open-label sitagliptin 100mg.

Section 4.1.8.2. Meal Tolerance test (MTT) – Part C only

ADDED TEXT

On Day 1 (full PK sampling day), all meals will be of the same composition as those on the PD profiling days, but no PD samples will be taken.

On Days -1, 1, 7, 13 and 14 the subjects must eat all of the breakfast meal provided.

Section 4.1.8.3. Hunger, Satiety and Caloric Intake (Part C)

ADDED TEXT

The modified HCFQ will be administered **after dinner on Days -1, 7, 13 and 14, (see Appendix 3 and SPM for details)**. Any subject who withdraws from the study should complete the modified HCFQ at withdrawal.

If feasible, on Days -1, 7, 13 and 14, calorie counts will be obtained across the meal periods and during the overall 24h **(note that the subjects must eat the whole breakfast meal on these days)**.

Section 4.1.8.4. Other Pharmacodynamic Profiling

ADDED TEXT

In Part C, assessment of 24-hour glucose, insulin, C-peptide, glucagon, GLP-1 (total and active), GIP **(total),** PYY **(total)** at baseline (Day -1), and on Days 7 and 13 will allow evaluation of the pharmacodynamic effect of repeat-dosing of GSK1292263 versus placebo and sitagliptin, and will permit comparison of the three dose levels (low, medium, high) of GSK1292263. In addition, the effect of adding sitagliptin to GSK1292263 will be assessed primarily by comparing the responses on Day 13 and Day 14, but further information may be forthcoming by comparing Day -1 and Day 14. **Other biomarkers, such as glycerol, may be assessed at the same timepoint.**

Section 4.2. Treatment Assignment

REVISED TEXT

A **web-based** central randomization method will be employed in Part C. Upon confirmation of eligibility, investigators or designated staff will **use** the I**W**RS to randomize a subject. Once a randomization number is assigned to a subject, it cannot be re-assigned to any other subject during the study.

Section 4.6. Time and Events Table (Part C)

PREVIOUS TEXT

Procedure	Screening ≤28 days prior to first dose	Period 1										Follow-up
		Days -14 to Day -3	Day -2	Day -1	Day 1	Days 2-6	Day 7	Days 8-12	Day 13	Day 14	Day 15	7 -10 days after discharge
Clinic Visit	X	Washout[5]	---------------------In Clinic---------------------									X
Informed Consent	X											
Inclusion/ Exclusion Criteria	X											
Demographics	X											
Complete physical	X											
Check into Clinic			X									
Brief physical			X									X
Medical/medication/ drug /alcohol history	X											
Weight	X				X					X		
Waist Circumference					X					X		
Height / BMI Calculation	X											
12-lead ECG[1]	X			X	X		X		X	X	X	X
24-hr Holter	X											
Vitals[2]	X			X	X	X	X	X	X	X	X	X
Urine drug/alcohol screen	X		X									
Urine pregnancy test			X									X
Clinical Chemistry / Hematology / Urinalysis[3]	X		X		X		X			X	X	X
FSH/ Estradiol[4]	X											
HIV/ Hep B / Hep C	X											
Washout / Glucometer		X	X	X								
PGx[6]					X							
Dosing – Randomized study med[7]					X	X	X	X	X	X		
Mixed Meal Tolerance Test [8]				X			X		X	X		
Modified HCFQ [9]				X			X		X	X		
Calorie counts				X			X		X	X		
Standardized meals				X	X	X	X	X	X	X		
Blood samples for glucose and insulin/PD[10]				X			X		X	X	X	

Procedure	Screening ≤28 days prior to first dose	Period 1										Follow-up
		Days -14 to Day -3	Day -2	Day -1	Day 1	Days 2-6	Day 7	Days 8-12	Day 13	Day 14	Day 15	7 -10 days after discharge
PK profile blood samples[11]					X		X		X	X	X	
PK Trough Samples[11]						X	X					
Resume Washed-out Meds[12]											X	
Checkout from clinic											X	
Concomitant Medication Review[13]	X	X	X	X	X	X	X	X	X	X	X	X
AE assessment			X	X	X	X	X	X	X	X	X	X

Continued

1. Single ECGs will be taken at Screening, on Day -1 before breakfast, on Days 1, 7, 13 and 14 at pre-dose (time 0), 1, 3, 9, 12 and 24hour, and at Follow-up. ECGs should be taken while subject is supine. Additional ECGs may be taken at the discretion of the investigator as needed based on symptoms or ECG findings.
2. Assessment of vital signs (including blood pressure and heart rate) will be performed at Screening, on Day -1, on each in-house day pre-dose, and at Follow-up. On Days 1, 7, 13 and 14, they will be taken at pre-dose (time 0), 1, 3, 9, 12 and 24 hours. At each time point, assessment should be performed after resting in a supine or semi-supine position for at least 10 minutes.
3. Blood samples for safety will be collected at screening, pre-dose (Day -1, time 0), Day 7, Day 14 and Day 15 (prior to checkout, =24hrs post-dose), and at follow-up. When this results in multiple samples at the same time point, only one sample will be collected (e.g., when 24hrs post-first-dose = pre-dose (time 0) for the second dose). Refer to Section 7.2.4 for specific laboratory parameters to be tested.
4. FSH and Estradiol tests will be performed for all post-menopausal women.
5. Subjects washing out of fibrates should be instructed to do so beginning on Day -14, but no visit occurs at that time.
6. PGx sample may be taken at any time after the first dose.
7. For QD dosing, study drug will be administered immediately after breakfast. For BID dosing study drug will be administered immediately after breakfast and immediately after the evening meal but prior to the 10hr PK sample (see Study Procedures Manual for details of sampling times relative to dosing times),
8. Composition of the meal tolerance test is specified in the SPM.
9. Modified HCFQ is administered at the same time of day, ±1 hour.
10. Blood samples for the determination of glucose and insulin and other PD markers will be collected at pre-dose (time 0) on Days -1, 13 and 14, and then immediately prior to and at 10, 20, 30, 60, 90, 120, 180min after eating the standardised breakfast meal tolerance test. For lunch (approximately 4h post morning dose) and the evening meal (approximately 10h post morning dose), samples will be collected just before the meal and at the following times after starting each meal: 0.5, 1, 1.5, 2 and 3 hours. A sample will also be collected 24 hours post-dose. On Day 7, samples for glucose and insulin and other PD markers will be collected at predose (= pre- breakfast), 1, 2, 4 (= pre lunch), 6, 10 and 12h, if blood volume permits. When this results in multiple samples at the same time point, only one sample will be collected (e.g., 24hrs post-first-dose = pre-dose (time 0) for the second dose). Refer to the Study Procedures Manual for additional details on the collection and processing of PD samples.
11. Blood samples for the determination of PK will be collected at the following times (PK sample times may be changed based on observed PK profile, but the total number of samples will not change) on Days 1, 13 and 14: Immediately pre-dose (time 0) and at 0.5, 1, 1.5, 2, 3, 4, 6, 8, 14 and 24 hours post-dose, (when this results in multiple samples at the same time point, only one sample will be collected (e.g., 24hrs post-first-dose = pre-dose (time 0) for the second dose). On Day 7, samples for PK will be collected at predose (=pre- breakfast), 1, 2, 4 (= pre lunch), 6, 10 (= immediately post-dinner) and 12h. Trough samples for PK will be collected immediately pre-dose on Days 4, 5, 6 and 7 . Refer to the Study Procedures Manual for details on the collection and processing of PK samples. When GSK1292263 is dosed BID in Cohorts 3 or 4 blood samples for PK sampling will be collected at immediately pre-dose, 1, 2, 4, 6, 8, 10, 11, 12, 14, 16, 18, and 24hrs post-dose.
12. If appropriate, the PI has the option of adjusting doses of washed-out medications when they are resumed. Review of glucose results should occur, and doses of oral anti-diabetic medications adjusted based on pharmacologic effects of treatment observed during the study.

REVISED TEXT

Procedure	Screening ≤28 days prior to first dose	Period 1										Follow-up
		Days -14 to Day -3	Day -2	Day -1	Day 1	Days 2-6	Day 7	Days 8-12	Day 13	Day 14	Day 15	7 -10 days after discharge
Clinic Visit	X	Washout[5]	------------------------------In Clinic------------------------------									X
Informed Consent	X											
Inclusion/ Exclusion Criteria	X											
Demographics	X											
Complete physical	X											
Check into Clinic			X									
Brief physical			X									X
Medical/medication/ drug /alcohol history	X											
Weight	X				X					**X**		~~X~~
Waist Circumference					X					**X**		
Height / BMI Calculation	X											
12-lead ECG[1]	X			X	X		X		X	X	X	X
24-hr Holter	X											
Vitals[2]	X			X	X	X	X	X	X	X	X	X
Urine drug/alcohol screen	X		**X**	~~X~~								
Urine pregnancy test			**X**	~~X~~								X
Clinical Chemistry / Hematology / Urinalysis[3]	X		**X**	~~X~~	**X**		X			X	X	X
FSH/ Estradiol[4]	X											
HIV/ Hep B / Hep C	X											
Washout / Glucometer		X	X	X								
PGx[6]					X							
Dosing – Randomized study med[7]					X	X	X	X	X	X		
Mixed Meal Tolerance Test [8]				X			X		X	X		
Modified HCFQ [9]				X			X		**X**	X		
Calorie counts				X			X		X	X		
Standardized meals				X	X	X	X	X	X	X		
Blood samples for glucose and insulin/PD[10]				X			X		X	X	X	
PK profile blood samples[11]					X		X		X	X	X	
PK Trough Samples[11]						X	X					

Procedure	Screening	Period 1										Follow-up
	≤28 days prior to first dose	Days -14 to Day -3	Day -2	Day -1	Day 1	Days 2-6	Day 7	Days 8-12	Day 13	Day 14	Day 15	7 -10 days after discharge
Resume Washed-out Meds[12]											X	
Checkout from clinic											X	
Concomitant Medication Review[13]	X	X	X	X	X	X	X	X	X	X	X	X
AE assessment			X	X	X	X	X	X	X	X	X	X

Continued

1. ECGs will be taken at Screening, and on Day -1,,1, 7, 13 and 14 pre-breakfast dose (fasting) and at 1, 3, 6, 9, 12 and 24hour, and at Follow-up. Assessments should be made in triplicate at each pre-dose time point, and single assessments should be made at all other times. ECGs should be taken while subject is supine. Additional ECGs may be taken at the discretion of the investigator as needed based on symptoms or ECG findings.
2. Assessment of vital signs (including blood pressure and heart rate) will be performed at Screening, on Days -1 to 14 in a fasting state early in the morning (prior to morning dosing on days 1-14), and at Follow-up. On Days 1, 7, 13 and 14, they will also be taken at 1, 3, 6, 9, 12 and 24 hours after the morning dose. At each time point, assessment should be performed after resting in a supine or semi-supine position for at least 10 minutes.
3. Blood samples for safety will be collected at screening, on Day -2 (can be non-fasting), and prior to breakfast (early in the morning, fasting) on Days 1, 7, and 14, and on Day 15 prior to checkout, (=24hrs post-dose), and at follow-up. When this results in multiple samples at the same time point, only one sample will be collected (e.g., when 24hrs post-first-dose = pre-dose (time 0) for the second dose). Refer to Section 7.2.4 for specific laboratory parameters to be tested.
4. FSH and Estradiol tests will be performed for all post-menopausal women.
5. Subjects washing out of fibrates should be instructed to do so beginning on Day -14, but no visit occurs at that time.
6. PGx sample may be taken at any time after the first dose.
7. For QD dosing, study drug will be administered immediately after breakfast. For BID dosing study drug will be administered immediately after breakfast and immediately after the evening meal, but prior to the 10hr PK sample (see Study Procedures Manual for details of sampling times relative to dosing times),
8. Composition of the meal tolerance test is specified in the SPM.
9. Modified HCFQ is administered after dinner, at the same time of day, ±1 hour, on Days -1, 7, 13 and 14.
10. Blood samples for the determination of glucose and insulin and other PD markers will be collected fasting pre-breakfast and then pre-morning dose (PD time 0) on Days -1, 13 and 14, and then iat 10, 20, 30, 60, 90, 120, 180min after eating the standardised breakfast meal tolerance test. For lunch (approximately 4h post morning dose) samples will be collected just before the meal and at the following times after starting each meal: 0.5, 1, 1.5, 2 and 3 hours. For the evening meal (approximately 10h post morning dose), BID dosing groups should follow the sequence of sampling, food and dosing as for breakfast (PD sample immediately before meal, eat and then dose), then 0.5, 1, 1.5, 2 and 3 hours post dinner. A sample will also be collected 24 hours post-dose. On Day 7, samples for glucose and insulin and other PD markers will be collected fasting, pre-breakfast and at 1, 2, 4 (= pre lunch), 6, 10 and 12h post morning dose, if blood volume permits. When this results in multiple samples at the same time point, only one sample will be collected (e.g., 24hrs post-first-dose = pre-dose (time 0) for the second dose). Refer to the Study Procedures Manual for additional details on the collection and processing of PD samples.
11. Serial blood samples for the determination of PK will be collected on Days 1, 7, 13 and 14. PK sampling times may be changed based on observed PK profile, but the total number of samples will not change. When GSK1292263 is dosed QD, blood samples for PK will be collected on Days 1, 13, and 14 immediately pre-dose (time 0) and at 0.5, 1, 1.5, 2, 3, 4, 6, 8, 14 and 24 hours post-dose. When GSK1292263 is dosed BID, blood samples for PK will be collected on Days 1, 13 and 14, at immediately pre-morning dose, 1, 2, 4, 6, 8, 10, 11, 12, 14, 16,18, and 24hrs post-morning dose. For QD and BID dosing regimens on Day 7, blood samples for PK will be collected at predose (=post- breakfast), 1, 2, 4 (= pre lunch), 6, 10 (= immediately post-dinner) and 12h. For QD and BID dosing regimens, trough samples for PK will be collected early in the morning (fasting) on Days 4, 5, and 6. When planned PK sampling results in multiple samples at the same time point, only one sample will be collected (e.g., 24hrs post-first-dose = pre-dose (time 0) for the second dose). Refer to the Study Procedures Manual for details on the collection and processing of PK samples.
12. If appropriate, the PI has the option of adjusting doses of washed-out medications when they are resumed. Review of glucose results should occur, and doses of oral anti-diabetic medications adjusted based on pharmacologic effects of treatment observed during the study.
13. At screening, medications taken in the 90 days prior should be collected.

Section 5.2.1. Inclusion Criteria

PREVIOUS TEXT

2. A female subject is eligible to participate if she is of non-childbearing potential, defined as pre-menopausal females with a documented tubal ligation or hysterectomy; or postmenopausal defined as 12 months of spontaneous amenorrhea. FSH and estradiol levels will be checked at Screening for postmenopausal women. Simultaneous follicle stimulating hormone (FSH) > 40 mIU/ml and estradiol < 40pg/ml (<140pmol/L) is confirmatory.

REVISED TEXT

2. A female subject is eligible to participate if she is of non-childbearing potential, defined as pre-menopausal females with a documented tubal ligation or hysterectomy; or postmenopausal defined as 12 months of spontaneous amenorrhea. **If the clinical situation is unclear,** FSH and estradiol levels **may be used to confirm post-menopausal status** ~~will be checked~~ at Screening ~~for postmenopausal women~~. Simultaneous follicle stimulating hormone (FSH) > 40 mIU/ml and estradiol < 40pg/ml (<140pmol/L) is confirmatory **in the absence of a clear post-menopausal history.**

PREVIOUS TEXT

4. BMI (body mass index) within the range 22-35 kg/m^2, inclusive.

REVISED TEXT

4. BMI (body mass index) within the range **21.8-35.2** kg/m^2, inclusive.

ADDED TEXT

Inclusion 7…

- FPG **or fasting blood glucose** level ≤ 250mg/dL on Day **-2 or Day -1**

Section 5.2.2. Exclusion Criteria

ADDED TEXT

Significant renal disease as manifested by one or more of the following:

- **Glomerular filtration rate (or c**reatinine clearance) <60mL/min. (estimated from serum creatinine (SCr) and demographic data using the MDRD calculation):

PREVIOUS TEXT

7. Has a history of any of the following conditions:

- Clinically significant symptoms of gastroparesis
- Cholelithiasis or obstructive or inflammatory gallbladder disease within 3 months prior to Screening
- Gastrointestinal disease that could affect fat or bile acid absorption, including inflammatory bowel disease, chronic diarrhea, Crohn's or malabsorption syndromes within the past year

Gastrointestinal surgery

- Chronic or acute pancreatitis

REVISED TEXT

7. Has a history of any of the following conditions:

- Clinically significant symptoms of gastroparesis
- Cholelithiasis or obstructive or inflammatory gallbladder disease within 3 months prior to Screening
- Gastrointestinal disease that could affect fat or bile acid absorption**, or the pharmacokinetics or pharmacodynamics of the study drugs**, including inflammatory bowel disease, chronic diarrhea, Crohn's or malabsorption syndromes within the past year
- Gastrointestinal surgery **that may affect the pharmacokinetics or pharmacodynamics of the study drugs**

Subjects may be enrolled in the study if they have had a cholecystectomy three or more months before the time of screening and are stable and asymptomatic.

- Chronic or acute pancreatitis

Section 6.2.3.6. Hunger, Craving, and Fullness Questionnaire and Calorie Counts (Revised Header)

ADDED TEXT

The HCFQ data will be listed and summarized with frequency counts at each time point by treatment. **Calorie count data will be summarized at each time point.** Additional details will be provided in the RAP.

Section 7. Study Assessments and Procedures

ADDED TEXT

…**When procedures are close to dosing and meals, the following order will be employed, as appropriate: ECGs, vitals, blood sampling, meal ingestion, dosing.** The timing of the assessments should allow the blood draw to occur at the exact nominal time.

Section 7.2.3. Electrocardiogram (ECG)

PREVIOUS TEXT

- Single 12-lead ECGs will be obtained in a supine position at each timepoint noted in the Time and Events Tables (Section 4.5 and Section 4.6) during the study using an ECG machine that automatically calculates the heart rate and measures PR, QRS, QT, and QTc intervals. Refer to Section 4.4.2. for QTc withdrawal criteria and additional QTc readings that may be necessary.
- Continuous cardiac telemetry will be performed as noted in the Time and Events Tables (Section 4.5 and Section 4.6). Full disclosures will be maintained as part of the subject's source documents and will be reviewed in detail.

REVISED TEXT

- ~~Single~~ 12-lead ECGs will be obtained in a supine position at each timepoint noted in the Time and Events Tables (Section 4.5 and Section 4.6) during the study using an ECG machine that automatically calculates the heart rate and measures PR, QRS, QT, and QTc intervals. Refer to Section 4.4.2 for QTc withdrawal criteria and additional QTc readings that may be necessary.
- Continuous cardiac telemetry will be performed **in Parts A and B** as noted in the Time and Events Table~~s~~ (Section 4.5 ~~and Section 4.6~~). Full disclosures will be maintained as part of the subject's source documents and will be reviewed in detail.

Section 7.2.4. Clinical Laboratory Assessments

DELETED TEXT

Clinical Chemistry

BUN	Potassium	AST (SGOT)	Total and direct bilirubin
Creatinine	Chloride	ALT (SGPT)	Uric Acid
Glucose, fasting	Total CO_2	GGT	Albumin
Sodium	Calcium	Alkaline phosphatase	Total Protein
Magnesium	Triglycerides	Total Cholesterol	LDL cholesterol
Phosphorus	FFA (NEFA)	HDL cholesterol	~~glycerol~~

Section 7.5.1. Type II Diabetes Biomarkers/Pharmacodynamic Markers

ADDED TEXT

Blood samples for assessment of 24-hour glucose, insulin, C-peptide, glucagon, GLP-1 (total and active), total GIP, total PYY **and glycerol** will be collected at the timepoints indicated in the Time and Events Tables

Section 7.5.2. Exploratory Biomarkers

ADDED TEXT

Exploratory biomarkers, if analyzed, will be assessed from the PD blood samples.

Section 8. Lifestyle And/Or Dietary Restrictions

PREVIOUS TEXT

Water	Water is permitted until one hour prior to study drug administration when subjects are dosed in the unit. In Parts A and C, subjects may consume water *ad libitum* after dosing, provided it does not adversely affect the OGTT in Part A. In Part B, subjects may consume water *ad libitum* beginning one hour after dosing.
Breakfast	Breakfast will be omitted on days when the OGTT will be administered (Part A). On Days -1, 7, 13 and 14 the breakfast meal will be referred to as a meal tolerance test (MTT). See SPM for menu details.
Lunch	In Parts A and B, a standardized lunch will be fed on Day 1 of each period. In Part C, a standardized lunch will be consumed on Days -1 through 14 at approximately 4h after the morning dosing.
Evening meal	In Parts A and B, a standardized evening meal will be fed on Day 1 of each period. In Part C, a standardized evening meal will be consumed on Days -1 through 14 at approximately 10h after the morning dose. For Cohort 4 (BID or QD dosing), see SPM for dosing in relation to meals and blood sampling.
Evening Snack	No evening snack will be permitted on Day 1 in Part A, and Days -1, 7 and 14 in Part C. On other days, an evening snack will be permitted at approximately 12 - 14 hours after dosing and up to 23:00.

REVISED TEXT

Water	Water is permitted until one hour prior to study drug administration when subjects are dosed in the unit in Parts A and B. In Parts A and C, subjects may consume water *ad libitum* after dosing, provided it does not adversely affect the OGTT in Part A. In Part B, subjects may consume water *ad libitum* beginning one hour after dosing.
Breakfast	Breakfast will be omitted on days when the OGTT will be administered (Part A). In Part C, on Days -1, 7, 13 and 14 the breakfast meal will be referred to as a meal tolerance test (MTT). See SPM for menu details. The composition of the breakfast meal on D1 will be the same as that of the MTT.
Lunch	In Parts A and B, a standardized lunch will be fed on Day 1 of each period. In Part C, a standardized lunch will be consumed on Days -1 through 14 at approximately 4h after the morning dosing. The same lunch will be provided on Days -1, 1, 7, 13 and 14.
Evening meal	In Parts A and B, a standardized evening meal will be fed on Day 1 of each period. In Part C, a standardized evening meal will be consumed on Days -1 through 14 at approximately 10h after the morning dose. The same evening meal will be provided on Days -1, 1, 7, 13 and 14. For Cohort 4 (BID or QD dosing), see SPM for dosing in relation to meals and blood sampling.
Evening Snack	No evening snack will be permitted on Day 1 in Part A, and Days -1, 7, 13 and 14 in Part C. On other days, an evening snack will be permitted at approximately 12 - 14 hours after dosing and up to 23:00.

AMENDMENT 2

Where the Amendment Applies

Amendment applies to all sites.

Summary of Amendment Changes with Rationale

The changes in this amendment are (i) adds co-dosing of GSK1292263 with sitagliptin on Day 14 of Part C. (ii) Cohort 4 will no longer be optional and will evaluate QD or BID doses of GSK1292263, depending on the dosing regimen chosen for Cohort 3, (iii) the maximum daily dose of GSK1292263 that may be administered in this study is set at 800mg total daily dose, (iv) the prior anti-diabetic therapy inclusion criteria for Part C are modified, (v) modifications to sampling times, (vi) adds HCFQ on Day 13.

List of Specific Changes

Section 1.3. Sitagliptin

ADDED TEXT

In this study, open-label sitagliptin is being used as a comparator to allow estimation of the relative efficacy of GSK1292263 on glycemic parameters. **A single dose of open-label sitagliptin will be co-administered with GSK1292263 or placebo (placebo arm) on Day 14 in Part C (Cohorts 3 and 4) to determine the extent of pharmacokinetic and pharmacodynamic interactions in subjects with T2DM.**

ADDED TEXT

By blocking DPP-IV, sitagliptin increases the systemic levels of the active form of the incretins, GLP-1 and GIP, and reduces the level of $PYY_{3\text{-}36}$, a form that has anorexigenic properties. At the same time it reduces the levels of total GLP-1, GIP and PYY via a putative negative feedback loop at the secretory L and K cells in the gut. **In the FTIH study (GPR111596), a reduction in total GLP-1, GIP and PYY was observed when 250mg GSK1292263 was co-administered with 100mg sitagliptin to normal healthy subjects, compared to the levels observed with GSK1292263 alone.**

Section 1.4. Rationale

ADDED TEXT

Data from this study will be used to assess the potential of the GPR119 agonist GSK1292263 as a treatment for T2DM, and will aid the design and dose selection of future studies of longer duration in T2DM subjects that will evaluate GSK1292263 alone or in combination with other anti-diabetic drugs**, such as a DPP-IV inhibitor or metformin.**

Section 1.4.1. Study Rationale

PREVIOUS TEXT

This will be the first use of GSK1292263 in T2DM subjects, and is intended to assess the safety, tolerability, pharmacokinetic and pharmacodynamic profile of this investigational drug following single doses (Parts A [Cohort 1] and B [Cohort 2]), and subsequently 14 days of once-daily dosing (Part C, Cohort 3). In Parts A and C, open-label sitagliptin is included as a comparator to provide data that will aid the assessment of the therapeutic potential of GSK1292263. Part B is being conducted to provide an estimate, in T2DM, of the magnitude of the effect of food on the PK of GSK1292263 at the highest dose that will be used in the study. If necessary, this will allow refinement of the doses selected for Part C. (See the SPM for timings of meals in relation to dosing.)

If the PK/PD evaluations in Parts A and B indicate that there may be loss of efficacy at the evening meal, then an optional Cohort 4 may be included in Part C to investigate the PK and PD of BID dosing of GSK1292263. Based on emergent data, there may also be an adjustment of the dosing regimen in the GSK1292263 arms of Cohort 3 in Part C to allow more extensive evaluation of BID doses. See Figure 1.

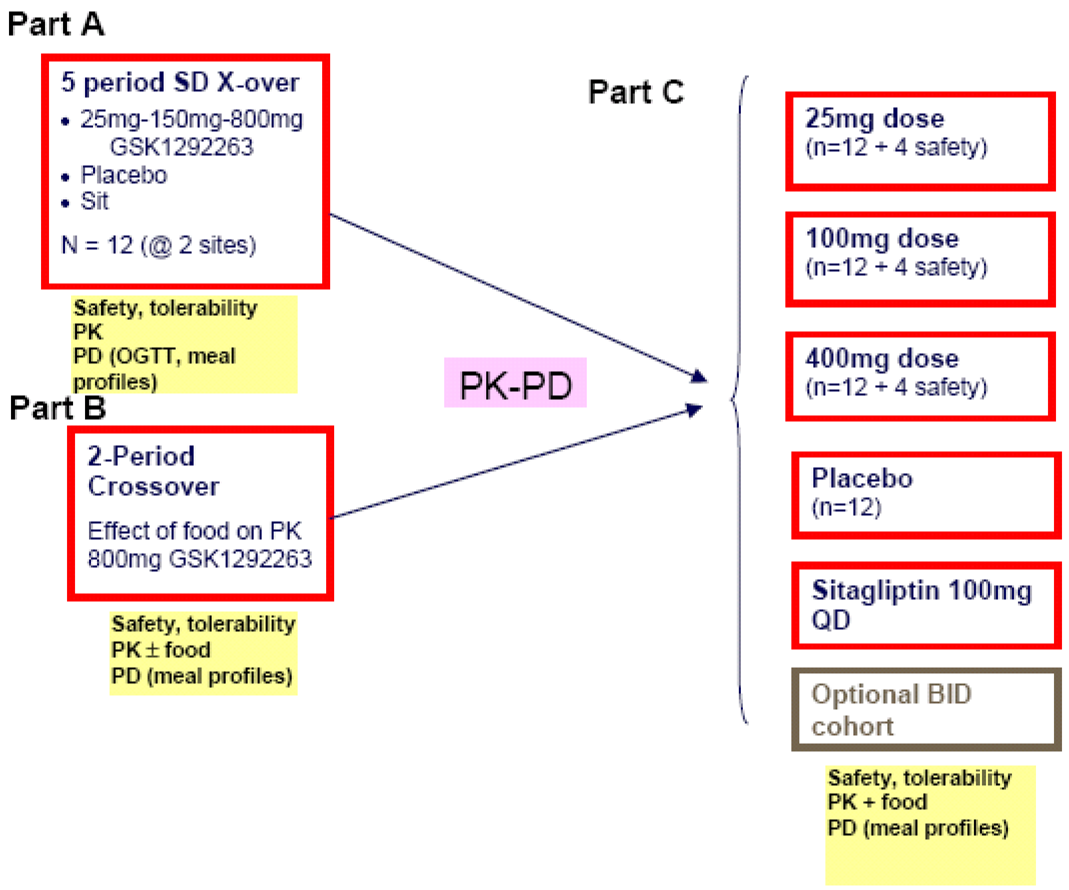

REVISED TEXT

This will be the first use of GSK1292263 in T2DM subjects, and is intended to assess the safety, tolerability, pharmacokinetic and pharmacodynamic profile of this investigational drug following single doses (Parts A [Cohort 1] and B [Cohort 2]), and subsequently 14 days of ~~repeat~~ dosing **in Part C**.

There are two scenarios for frequency of dosing in Part C of this study, summarized below:

Scenario 1: QD dosing Part C, Cohort 3 and BID dosing in Part C, Cohort 4

Scenario 2: BID dosing in Part C, Cohort 3 and QD dosing in Part C, Cohort 4

The actual scenario of dosing (QD or BID) in Part C will be determined based on preliminary PK/PD, safety and tolerability data in Parts A and B.

In Parts A and C, open-label sitagliptin is included as a comparator to provide data that will aid the assessment of the therapeutic potential of GSK1292263. Part B is being conducted to provide an estimate, in T2DM, of the magnitude of the effect of food on the PK of GSK1292263 at the highest dose that will be used in the study. If necessary, this will allow refinement of the doses selected for Part C. (See the SPM for timings of meals in relation to dosing.)

In Part C, subjects randomized to GSK1292263 or placebo will be co-administered **as a single dose of** 100mg of sitagliptin on Day 14 to determine the extent of pharmacokinetic and pharmacodynamic interactions in subjects with T2DM. **For BID dosing regimens, sitagliptin will be co-administered with the first daily dose of GSK1292263 on Day 14.** In the FTIH study GPR111596, sitagliptin increased the exposure of GSK1292263 by ~50%, but GSK1292263 did not alter the exposure of sitagliptin. The pharmacokinetics of both GSK1292263 and sitagliptin will be assessed on Day 14 in Part C.

Inhibition of DPP-IV by sitagliptin increases the circulating concentrations of active GLP-1 after nutrient stimulation and this may result in a further augmentation of the levels of active incretins when co-administered with GSK1292263. On the other hand, the DPP-IV inhibitor may reduce the levels of circulating $PYY_{[3\text{-}36]}$ and $GLP_{[9\text{-}36]}$ when co-administered with GSK1292263 because DPP-IV cleaves inactive $PYY_{[1\text{-}36]}$ to form active $PYY_{[3\text{-}36]}$, as well as active $GLP_{[7\text{-}36]}$ to form $GLP_{[9\text{-}36]}$, which also has biological effects.

It is important to note that the current study GPR111598 with T2DM subjects offers the opportunity to safely investigate doses higher than 400mg, the highest dose evaluated in the FTIH study GPR111596.

This is important for 3 reasons:

1. **To establish the safety and tolerability of a dose (800mg) that is a substantial multiple of the predicted therapeutic dose (currently approximately 100mg). This is relevant to the conduct of an adequate ‘Thorough ECG’ study in the future.**

2. **It provides an opportunity to investigate the pharmacological effects, if any, of a high dose that is predicted to increase systemic exposure by an amount that is less than dose proportional in the fed and fasted states (See Figure 2). This may allow further understanding of the relative importance of systemic exposure versus local exposure in the gastrointestinal tract for the pharmacological effects of a GPR119 agonist, and will aid in the development of future non-absorbable molecules if the efficacy is mediated primarily by mechanisms in the gut lumen.**

   The data from the FTIH study indicate that the effect of food on systemic exposure is dose-dependent. The 800mg dose administered with and without food in Part B will allow further understanding of the effect of food and safety/tolerability at a dose that may be evaluated in Part C when subjects will be administered the study drug with food.

~~If the PK/PD evaluations in Parts A and B indicate that there may be loss of efficacy at the evening meal, then an optional Cohort 4 may be included in Part C to investigate the PK and PD of BID dosing of GSK1292263~~. Based on emergent data, there may also be an adjustment of the dosing regimen in the GSK1292263 arms of Cohort 3 in Part C to allow more extensive evaluation of BID doses. See Figure 1.

The maximum allowable total daily dose in the current study is 800mg administered as single or divided doses because the drug substance used in this study may contain a trichloromethyloxadiazole process impurity, GSK2116107A, which was mutagenic in a mini-Ames test (See Section1.5.2).

Figure 1 Study Design

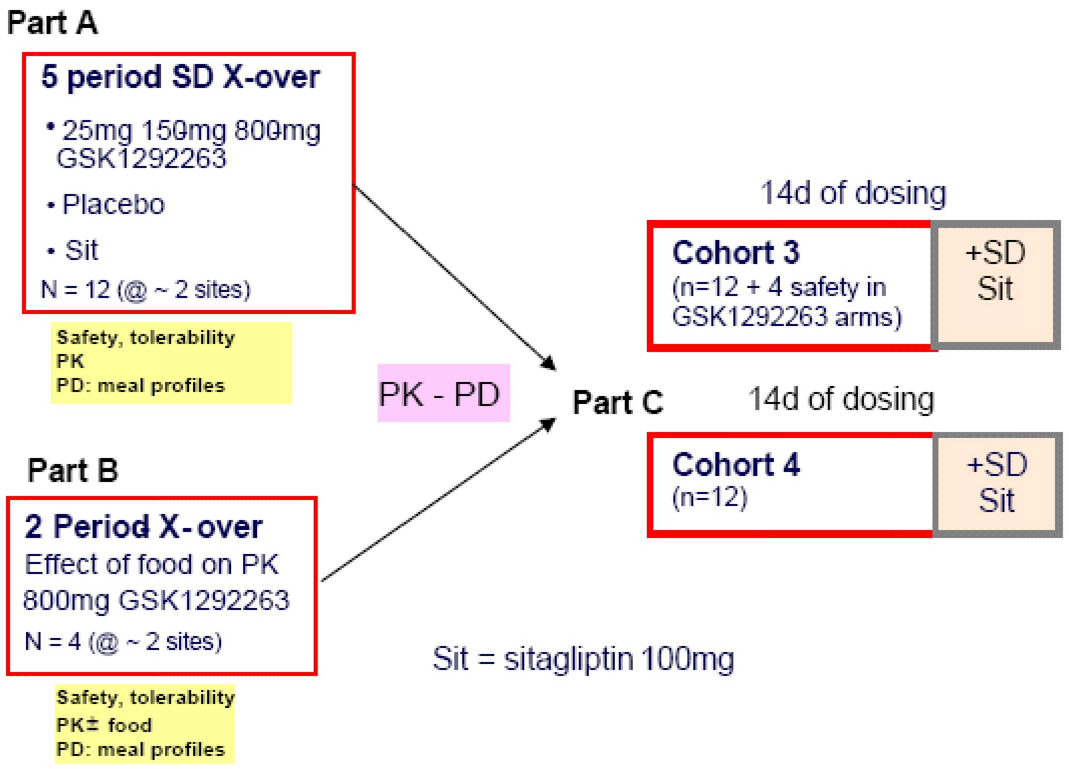

PART C	Example of QD/BID Dosing Scenario	Example of BID/QD Dosing Scenario
Cohort 3	25mg QD<br>(n=12 + 4 safety)	25mg BID<br>(n=12 + 4 safety)
	150mg QD<br>(n=12 + 4 safety)	150mg BID<br>(n=12 + 4 safety)
	800mg QD<br>(n=12 + 4 safety)	400mg BID<br>(n=12 + 4 safety)
	Placebo QD<br>(n=12)	Placebo BID<br>(n=12)
	Sitagliptin 100mg QD<br>(n=12)	Sitagliptin 100mg QD<br>(n=12)
Cohort 4	400mg BID<br>(n=12)	800mg QD<br>(n=12)

Section 1.4.2.3. Planned Doses and Predicted Exposures

PREVIOUS TEXT

Part C - Daily Dosing

The planned doses for Part C are 25, 100, and 400mg administered once daily for 14 days in the fed state. The predicted steady-state exposures relative to non-clinical toxicology limits for these doses are summarized in Table 8. PK/PD analysis will be performed on preliminary data from Parts A and B to characterize the dose/exposure-response relationship for GSK1292263. Along with safety and tolerability data, this analysis will be used to confirm the planned doses for Part C. Any adjustments to doses in Part C will not exceed 800mg once daily, which is predicted to maintain exposures below non-clinical toxicology limits as summarized in Table 8.

Table 8 Predicted Steady-State Exposures for GSK1292263 Daily Dosing (Part C)

Dose (mg)[4]	Predicted Steady-State Exposure[1]		Fold Cover Relative to NOAEL Limits[2,3]	
	AUC(0-24h) (ng.h/mL)	Cmax (ng/mL)	AUC(0-24h)	Cmax
25	1951	215	25.0	12.5
100	5911	562	8.4	4.8
400	11999	940	4.0	2.9
800	14485	1058	3.4	2.5

1. Predictions based on fasted exposure in FTIH study (GPR111596)
2. AUC(0-24h) limit = 49,400 ng.h/mL, Cmax limit = 2,693 ng/mL 80% of NOAEL in dogs at 1000mg/kg/day
3. No individuals predicted to exceed AUC(0-24h) limit of 72,700 ng.h/mL and Cmax of 3585ng/mL (80% of the highest AUC and Cmax observed in dog at 1000mg/kg NOAEL dose group), assuming plateau of exposures
4. Predictions based on PK in the fed state

Part C - Twice-Daily (BID) Dosing

PK/PD analysis will be performed on preliminary data from Parts A to characterize the dose/exposure-response relationship for GSK1292263. If PK/PD analysis indicates a potential pharmacodynamic advantage to BID dosing, an optional cohort (Cohort 4) may used to evaluate a BID regimen. The planned dose level for BID dosing is 400mg. However, based on PK/PD analysis from Part A, the BID dose level may be reduced. Predicted steady-state exposures following a dose of 400mg BID for 14 days in the fed state are summarized in Table 4. All exposures are predicted to remain below the group mean exposure limits based on non-clinical toxicology data. No subjects are predicted to exceed the individual exposure limits based on non-clinical toxicology data.

Based on emergent data, BID dosing of GSK1292263 may be investigated by conducting optional Cohort 4, or transitioning QD doses of GSK1292263 or placebo in Cohort 3 to a BID regimen, keeping the exposure limits outlined above.

Table 9 Predicted Steady-State Exposures for GSK1292263 BID Dosing (Part C)

BID Dose (mg)[4]	Predicted Steady-State Exposure[1]		Fold Cover Relative to NOAEL Limits[2,3]	
	AUC(0-24h) (ng.h/mL)	Cmax (ng/mL)	AUC(0-24h)	Cmax
400	25789	1602	1.9	1.7

1. Predictions based on fasted exposure in FTIH study (GPR111596)
2. Mean AUC(0-24h) limit = 49,400 ng.h/mL, Cmax limit = 2,693 ng/mL 80% of NOAEL in dogs at 1000mg/kg/day
3. No subjects predicted to exceed individual AUC(0-24h) limit of 72,700 ng.h/mL (80% of the highest AUC and Cmax observed in dog at 1000mg/kg NOAEL dose group). Approximately 2% of subjects predicted to slightly exceed individual Cmax limit of 3585 ng/mL
4. Predictions based on PK in the fed state

REVISED TEXT

Part C - Daily Dosing

If GSK1292263 is dosed QD, the planned doses for **Cohort 3 of** Part C are 25, 150, and 800mg administered once daily for 14 days in the fed state**, and Cohort 4 will evaluate half the** maximum **QD dose administered twice daily (see below)**. The predicted steady-state exposures **for the planned doses** relative to non-clinical toxicology limits for these doses are summarized in Table 3. **The final doses to be administered may be modified to ensure that exposures do not exceed exposure limits based on nonclinical toxicology data and based on PK/PD, safety and tolerability data from Parts A and B. For co-administration of GSK1292263 with sitagliptin on Day 14 it is assumed that steady-state AUC(0-24h) and Cmax will increase by ~56% and ~35%, respectively, compared to GSK1292263 alone on Day 13. These assumptions are based on increases in exposure observed in the FTIH study following single-dose co-administration of GSK1292263 and sitagliptin. Predicted co-dosing exposures are conservative (highest exposure) given that GSK1292263 and sitagliptin will only be administered as a single dose on Day 14 after GSK1292263 has been administered alone for 13 days. It should be noted that GSK1292263 did not affect the pharmacokinetics of sitagliptin in the FTIH study.** PK/PD analysis will be performed on preliminary data from Parts A and B to characterize the dose/exposure-response relationship for GSK1292263. Along with safety and tolerability data, this analysis will be used to confirm the planned doses for Part C. Any adjustments to doses in Part C will not exceed 800mg once daily, which is predicted to maintain exposures below non-clinical toxicology limits as summarized in Table 3.

Table 10 Predicted Steady-State Exposures for GSK1292263 Daily Dosing (Part C)

Dose (mg)[4]	Predicted Steady-State Exposure[1]		Fold Cover Relative to NOAEL Limits[2,3]	
	AUC(0-24h) (ng.h/mL)	Cmax (ng/mL)	AUC(0-24h)	Cmax
25	1951	215	25.0	12.5
100	5911	562	8.4	4.8
400	11999	940	4.0	2.9
800	14485	1058	3.4	2.5
400 + Sitagliptin	18718	1268	2.6	2.1
800 + Sitagliptin	22598	1429	2.2	1.9

1. Predictions based on fasted exposure in FTIH study (GPR111596)
2. AUC(0-24h) limit = 49,400 ng.h/mL, Cmax limit = 2,693 ng/mL 80% of NOAEL in dogs at 1000mg/kg/day
3. No individuals predicted to exceed AUC(0-24h) limit of 72,700 ng.h/mL and Cmax of 3585ng/mL (80% of the highest AUC and Cmax observed in dog at 1000mg/kg NOAEL dose group), assuming plateau of exposures
4. Predictions based on PK in the fed state. Co-administration with sitagliptin (100mg) is assumed to increase steady-state AUC(0-24h) and Cmax by 56% and 35%, respectively, based on single-dose PK in FTIH study

Part C - Twice-Daily (BID) Dosing (Cohort 3 or 4)

PK/PD analysis will be performed on preliminary data from Parts A to characterize the dose/exposure-response relationship for GSK1292263. **If Cohort 3 in Part C is dosed QD, then** Cohort 4 **will be conducted** to evaluate **half the** maximum **QD dose used in Cohort 3 administered twice a day for 14 days**. The planned **maximum** dose level for **twice daily** dosing is 400mg **BID (total daily dose – 800mg)**. However, based on **safety,tolerability and** PK/PD analysis from Part A, the BID dose level may be reduced. Predicted steady-state exposures following a dose of 400mg BID for 14 days in the fed state are summarized in Table 4. **For co-administration of GSK1292263 with sitagliptin on Day 14 it is assumed that steady-state AUC(0-24h) and Cmax will increase by ~56% and ~35%, respectively, compared to GSK1292263 alone on Day 13. These assumptions are based on increases in exposure observed in the FTIH study following single-dose co-administration of GSK1292263 and sitagliptin. Predicted co-dosing exposures are conservative (highest exposure) given that GSK1292263 and sitagliptin will only be administered as a single dose on Day 14 after GSK1292263 has been administered alone for 13 days. It should be noted that GSK1292263 did not affect the pharmacokinetics of sitagliptin in the FTIH study.** All exposures are predicted to remain below the group mean exposure limits based on non-clinical toxicology data. No subjects are predicted to exceed the individual exposure limits based on non-clinical toxicology data.

Based on emergent data **from Parts A and B,** BID dosing of GSK1292263 may be investigated **in Cohort 3** by **administering the** doses of GSK1292263 or placebo **as** a BID regimen, keeping the exposure limits outlined above. **In this case Cohort 4 will evaluate a QD dose of GSK1292263 that will not exceed 800mg.**

Table 11 Predicted Steady-State Exposures for GSK1292263 BID Dosing (Part C)

BID Dose (mg)[4]	Predicted Steady-State Exposure[1]		Fold Cover Relative to NOAEL Limits[2,3]	
	AUC(0-24h) (ng.h/mL)	Cmax (ng/mL)	AUC(0-24h)	Cmax
400	25789	1602	1.9	1.7
400mg + Sitagliptin	40230	2163	1.2	1.2

1. Predictions based on fasted exposure in FTIH study (GPR111596)
2. Mean AUC(0-24h) limit = 49,400 ng.h/mL, Cmax limit = 2,693 ng/mL 80% of NOAEL in dogs at 1000mg/kg/day
3. No subjects predicted to exceed individual AUC(0-24h) limit of 72,700 ng.h/mL (80% of the highest AUC and Cmax observed in dog at 1000mg/kg NOAEL dose group). Approximately 2% of subjects predicted to slightly exceed individual Cmax limit of 3585 ng/mL
4. Predictions based on PK in the fed state. Co-administration with sitagliptin assumed to increase steady-state AUC(0-24h) and Cmax by 56% and 35%, respectively, based on single-dose PK in FTIH study

Section 1.4.2.4. Sitagliptin

PREVIOUS TEXT

Subjects in Part C (Cohort 3) randomized to the sitagliptin-alone arm will receive 14 consecutive days of dosing with open-label 100mg sitagliptin.

REVISED TEXT

Subjects in Part C (Cohort 3 **and Cohort 4**) randomized to **GSK1292263 or placebo will be co-administered with a single open-label dose of 100mg sitagliptin on Day 14. Subjects in** the sitagliptin-alone arm will receive 14 consecutive days of dosing with open-label 100mg sitagliptin.

Section 1.4.3 Stopping Criteria

PREVIOUS TEXT

The target range of exposures for this study has been selected to allow for a robust characterization of safety, tolerability, PK and PD of GSK1292263 in T2DM volunteers. The highest single dose of GSK1292263 to be administered in the study will be 800mg.

REVISED TEXT

The target range of exposures for this study has been selected to allow for a robust characterization of safety, tolerability, PK and PD of GSK1292263 in T2DM volunteers. The highest **daily** dose of GSK1292263 to be administered in the study will be 800mg. The highest daily dose of GSK1292263 to be administered in the study will be 800mg **(administered as single or divided doses).**

Section 1.5.2. Risks Related to GSK1292263

PREVIOUS TEXT

- GSK1292263 increases glucagon secretion, and in animals studies this was enhanced during insulin-induced hypoglycemia.
- No fasting hypoglycemia was observed 24h after dosing in the FTIH study.
- T2DM subjects should be less prone than healthy normal volunteers to hypoglycemia associated with an OGTT or Meal Tolerance Test.

Details of any hypoglycemic episodes will be captured in the Case Report Form.

Overall, the potential risk to subjects who receive GSK1292263 for 14 days is likely to be very small because:

- The likelihood of the plasma exposures exceeding NOAEL limits is small based on FTIH PK data.
- So far, GSK1292263 has been safe and well tolerated in normal subjects.

There is no indication from the mechanism of action of this drug that would predict

REVISED TEXT

- GSK1292263 increases glucagon secretion **during insulin-induced hypoglycaemia** in animals studies ~~this was enhanced during insulin-induced hypoglycemia~~.
- No fasting hypoglycemia was observed 24h after dosing in the FTIH study.
- T2DM subjects should be less prone than healthy normal volunteers to hypoglycemia associated with an OGTT or Meal Tolerance Test.

Details of any hypoglycemic episodes will be captured in the Case Report Form.

As indicated in Supplement 2 of the IB ([GlaxoSmithKline Document Number RM2009/00168/01]), the maximum allowable total daily dose in the current study is 800mg (administered as single or divided doses) because the drug substance used in this study may contain a trichloromethyloxadiazole process impurity, GSK2116107A, which was mutagenic in a mini-Ames test. Quantitative assessments indicate that GSK2116107A is not present in the final drug substance at a limit of detection of 50 ppm (which based on a clinical dose of 800 mg/day, equates to a total oral dose of less than 40 μg/day). There are no safety concerns for the conduct of a clinical trial at doses up to 800 mg/day for up to 28 days because the level of this impurity is below the staged TTC (Threshold of Toxicological Concern) limit of 60 μg/day for clinical trials of up to one month duration.

Overall, the potential risk to subjects who receive GSK1292263 is likely to be very small because:

- **The likelihood of the plasma exposures exceeding NOAEL limits is small based on FTIH PK data (see Section 1.4.2).**
- **So far, GSK1292263 has been safe and well tolerated in normal human subjects at doses ≤ 400mg, with no clinically significant changes in vital signs, ECGs, telemetry and lab parameters related to the study drug.**
- **The subjects are dosed in the clinical unit and observed closely for 24h.**
- **There is no indication that the mechanism of action of this drug would predict an increased risk of hypoglycaemia in subjects with T2DM. In addition:**
  - **Part A uses the dosing-OGTT paradigm that reduced hypoglycemic episodes in the FTIH study GPR111596**
  - **At the request of the IRB, in Part A and B the staff at the clinical site will be monitoring blood glucose in ‘real time’ using a glucometer, as indicated in Amendment 1 of the protocol GPR111598 ([GlaxoSmithKline Document Number RH2008/00140/01])**
  - **Subjects will be asked ‘how are you feeling’ to provide an assessment of neuroglycopenia.**

- **The drug substance used in this study has a lower level of impurities than that used in the FTIH study.**

~~The likelihood of the plasma exposures exceeding NOAEL limits is small based on FTIH PK data.~~

Section 1.5.3. Capillary Blood Glucose (CBG) Monitoring

PREVIOUS TEXT

During the wash-out period prior to Parts B and C all subjects, even if not changing previous medications, must check fasting capillary glucose at least twice daily (fasting and prior to the evening meal), and at any time symptoms of hypoglycemia are experienced. A written diary card should be kept for each subject.

REVISED TEXT

~~During the wash-out period~~ **Subjects who are washed off prior anti-diabetic medications prior to dosing in** Parts B and C ~~all subjects, even if not changing previous medications,~~ must check fasting capillary glucose at least twice daily (fasting and prior to the evening meal) **during the washout period**, and at any time symptoms of hypoglycemia **or hyperglycemia** are experienced. A written diary card should be kept for each **of these** subjects. **Subjects who are drug naïve are not required to measure CBG and maintain a diary card prior to dosing in Parts B and C.**

Section 2.1 Primary Objectives

PREVIOUS TEXT

- To investigate in subjects with T2DM the safety and tolerability of GSK1292263 administered as single ascending (Part A) and repeat oral doses (Part C).
- To determine the pharmacokinetic parameters of GSK1292263 in subjects with T2DM following single ascending (Part A) and repeat oral doses (Part C).
- To evaluate in T2DM subjects the pharmacodynamic effects of GSK1292263 following single ascending doses (Part A) and repeated doses (Part C), and the pharmacokinetic/pharmacodynamic relationships.

REVISED TEXT

- To investigate in subjects with T2DM the safety and tolerability of GSK1292263 administered as single ascending (Part A) and repeat oral doses **administered QD or BID** (Part C).
- To determine the pharmacokinetic parameters of GSK1292263 in subjects with T2DM following single ascending (Part A) and repeat oral doses **administered QD or BID** (Part C).
- To evaluate in T2DM subjects the pharmacodynamic effects of GSK1292263 following single ascending doses (Part A) and repeated **oral** doses **administered QD or BID** (Part C), and the pharmacokinetic/pharmacodynamic relationships.

Section 2.2. Secondary Objectives

PREVIOUS TEXT

- To investigate in subjects with T2DM the safety and tolerability of GSK1292263 when administered as a single dose with food (Part B).
- To determine the pharmacokinetic parameters of GSK1292263 in subjects with T2DM when administered as a single dose with food (Part B).
- To establish the pharmacodynamic effects of GSK1292263 in subjects with T2DM when administered as a single dose with food (Part B).

REVISED TEXT

- To investigate in subjects with T2DM the safety and tolerability of GSK1292263 when administered as a single dose with food (Part B).
- To determine the pharmacokinetic parameters of GSK1292263 in subjects with T2DM when administered as a single dose with food (Part B).
- To establish the pharmacodynamic effects of GSK1292263 in subjects with T2DM when administered as a single dose with food (Part B).
- **To investigate the safety and tolerability of GSK1292263 co-administered with a single dose of sitagliptin (Part C).**
- **To determine the pharmacokinetic parameters of (i) GSK1292263 and sitagliptin when co-administered with a single dose of sitagliptin on Day 14, and (ii) sitagliptin alone (sitagliptin arm) on Day 14. (Part C)**
- **To evaluate the pharmacodynamic effects GSK1292263 when co-administered with a single dose of sitagliptin (Part C).**
- **To estimate the relative bioavailability of GSK1292263 and sitagliptin when administered together, compared to sitagliptin alone and GSK1292263 alone (Part C).**

Section 3.1. Primary Endpoints

PREVIOUS TEXT

- Safety and tolerability parameters following single and repeat doses of GSK1292263 including adverse events, and assessments of clinical laboratory, ECGs and vital signs.
- Pharmacokinetic parameters following single and repeat doses of GSK1292263: Cmax, Tmax, t½, tlag, CL/F, V/F, AUC(0-τ), AUC(0-∞) for single dose and repeat doses, accumulation ratio (Ro), time invariance ratio (Rs), as data permit.

REVISED TEXT

- Safety and tolerability parameters following single and repeat doses of GSK1292263 **administered QD or BID,** including adverse events, and assessments of clinical laboratory, ECGs and vital signs.
- Pharmacokinetic parameters following single and repeat doses of GSK1292263 **administered QD or BID:** Cmax, Tmax, t½, tlag, CL/F, V/F, AUC(0-τ), AUC(0-∞) for single dose and repeat doses, accumulation ratio (Ro), time invariance ratio (Rs), as data permit.

Section 3.2. Secondary Endpoints

ADDED TEXT

- **Pharmacokinetic parameters: AUC(0-τ), AUC(0-24), AUC(0-12), Cmax, and tmax of GSK1292263 and sitagliptin when co-administered, and sitagliptin alone (sitagliptin arm) (Part C), as data permit.**

Section 4.1.2. Part B: GSK1292263 with Food (Cohort 2)

PREVIOUS TEXT

Part B (Cohort 2) is a single-blind, randomized, 2-period study in which T2DM subjects will receive a single dose of GSK1292263, fasted or fed. T2DM subjects on monotherapy or sub-maximal anti-diabetic medications will wash-off these medications for 1 week prior to study receiving study drug. This will commence after dosing in Part A is completed.

In the FTIH study, GPR111596, food increased the exposure of GSK1292263 by up 2-to 3-fold in normal volunteers. Part B will comprise a two-period crossover of a single tablet dose of GSK1292263 administered in the fed or fasted state. The planned dose in Part B will be 800mg and will be administered after eating a high fat breakfast meal (see the SPM for specifics). This dose may be changed based on emergent safety, tolerability and PD data. Results from Part B will be used to confirm the 'food effect' seen in study GPR111596, and will allow assessment of the extent of the change in PK exposure in the presence of food in T2DM patients. This will aid in the selection of doses in Part C, where the subjects will eat a standardised meal tolerance test (MTT) at breakfast. Part B will be conducted in parallel with the last two periods of Part A.

REVISED TEXT

Part B (Cohort 2) is a single-blind, randomized, 2-period study in which T2DM subjects will receive a single dose of GSK1292263, fasted or fed. T2DM subjects on monotherapy or sub-maximal anti-diabetic medications will wash-off these medications for 1 week prior to study receiving study drug. **Before subjects in Part B are dosed, the safety and tolerability of 800mg GSK1292263 administered in the fasted state to 9**~~This will commence after~~ **subjects** ~~dosing in Part A~~ **will be reviewed to ensure that it is safe to proceed.**~~ is completed.~~

In the FTIH study, GPR111596, food increased the exposure of GSK1292263 by up 2-to 3-fold in normal volunteers. Part B will comprise a two-period crossover of a single tablet dose of GSK1292263 administered in the fed or fasted state. The planned dose in Part B will be 800mg and will be administered after eating a high fat breakfast meal (see the SPM for specifics). This dose may be changed based on emergent safety, tolerability and PD data. Results from Part B will be used to confirm the 'food effect' seen in study GPR111596, and will allow assessment of the extent of the change in PK exposure in the presence of food in T2DM patients. This will aid in the selection of doses in Part C, where the subjects will eat a standardised meal tolerance test (MTT) at breakfast. ~~Part B will be conducted in parallel with the last two periods of Part A.~~

Section 4.1.3. Part C: Repeat Dosing (Cohorts 3 and 4)

PREVIOUS TEXT

Part C (Cohort 3, optional Cohort 4) is a single-blind, randomized, placebo-controlled, 5-arm study of 14 days of dosing with GSK1292263, placebo or open-label sitagliptin. An optional Cohort 4 may be enrolled to investigate the safety, tolerability, pharmacokinetics and pharmacodynamics of GSK1292263 when dosed in a BID regimen. Based on emergent data, there may also be an adjustment of the dosing regimen in other GSK1292263 arms of Part C from QD dosing to BID dosing, to allow more extensive evaluation of BID doses.

The planned doses of GSK1292263 in Part C will be 25, 100 and 400mg, but these doses may be changed based on emergent safety, tolerability, PK and PD data from Parts A and B. Ethics boards will be advised of the final doses chosen for Part C prior to the dosing of subjects. Safety, tolerability, PK and PD data from Parts A and B will be assessed prior to initiation of Part C.

T2DM subjects on monotherapy or sub-maximal anti-diabetic medications will wash-off these medications for 1 week prior to receiving study drug.

Part C (Cohort 3) will be conducted to assess safety, tolerability, PK and PD of GSK1292263 and open-label sitagliptin in T2DM subjects after 14-days of dosing.

Subjects will be randomized into one of 5 treatment arms: 25mg dose of GSK1292263 once-daily, 100mg dose of GSK1292263 once-daily, 400mg dose of GSK1292263 once-daily, placebo or sitagliptin.

Figure 3 Part C Schematic of Design

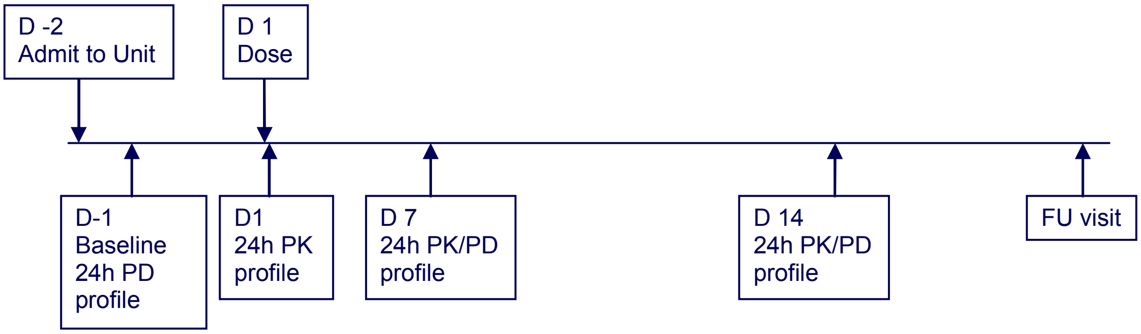

As shown in Figure 3, subjects will be admitted to the research facility within 48 hours (Day -2) of first dose (Day 1). The 24-hour time period prior to dosing (Day-1) will be defined as the baseline, and the treatment period will be defined as Days 1-14. Full 24hr profiles for PK will be conducted on Days 1, 7 and 14, and 24hr profiles for PD will be conducted on Days -1, 7 and 14.

REVISED TEXT

Part C (Cohort 3 **and optional Cohort** 4) is a single-blind, randomized, placebo-controlled, **6**~~5~~-arm study of 14 days of dosing with GSK1292263, placebo or open-label sitagliptin. **If Cohort 3 is dosed QD,** Cohort 4 **will** ~~may~~ be enrolled to investigate the safety, tolerability, pharmacokinetics and pharmacodynamics of GSK1292263 when dosed in a BID regimen. **Alternatively, if Cohort 3 is dosed** BID**, then Cohort 4 will be enrolled to investigate the safety, tolerability, pharmacokinetics and pharmacodynamics of GSK1292263 when dosed in a** QD **regimen**. Based on emergent data, there may also be an adjustment of the **dose or** dosing regimen in **the** ~~other~~ GSK1292263 arms of Part C ~~from QD dosing to BID dosing,~~ to allow more extensive evaluation of **QD or** BID doses.

If GSK1292263 is dosed QD ~~The planned doses of GSK1292263 in~~ Cohort 3 of Part C, the planned dosed are ~~will be~~ 25, 1**5**0, and **8**00mg QD. **In the event of a dose adjustment, the maximum dose to be administered QD will not exceed 800mg. Cohort 4 will evaluate half the maximal QD dose used in Cohort 3 administered twice a day for 14 days.**

~~, and Cohort 4 will evaluate half the maximum QD dose used in Cohort 3 administered twice a day for 14 days. Alternatively, i~~

If Cohort 3 of Part C is dosed BID, then the planned doses will not exceed 400mg BID (total daily dose of 800mg), and Cohort 4 will evaluate the maximum total daily dose used in Part C administered QD for 14 days.

These doses may be changed based on emergent safety, tolerability, PK and PD data from Parts A, ~~and~~ B and C. Ethics boards will be advised of the final doses chosen for Part C prior to the dosing of subjects. Safety, tolerability, PK and PD data from Parts A and B will be assessed prior to initiation of Part C.

T2DM subjects on monotherapy or sub-maximal anti-diabetic medications will wash-off these medications for 1 week prior to receiving study drug.

Part C (Cohort**s 3 and 4**) will be conducted to assess safety, tolerability, PK and PD of GSK1292263 and open-label sitagliptin in T2DM subjects after 14-days of dosing, **as well as the safety, tolerability, PK and PD of (i) a 100mg single dose of open-label sitagliptin co-administered with GSK1292263 or placebo (placebo arm), and (ii) sitagliptin alone (sitagliptin arm) on Day 14.**

Subjects **in Cohort 3** will be randomized into one of 5 treatment arms. **Planned doses for Cohort 3 are as follows:** 25mg dose of GSK1292263 once-daily, 1**5**0mg dose of GSK1292263 once-daily, **8**00mg dose of GSK1292263 once-daily, placebo or sitagliptin. **Alternatively, GSK1292263 may be administered BID in 3 treatment arms, for comparison to placebo (administered BID) and open label sitagliptin administered QD.**

Cohort 4 of Part C will be conducted to assess the PK and PD of BID or QD dosing of GSK1292263 in T2DM subjects after 14-days of dosing, as well as the safety, tolerability, PK and PD of a 100mg single dose of open-label sitagliptin co-administered with GSK1292263 on Day 14. If GSK1292263 is dosed QD in Cohort 3 of Part C, Cohort 4 will evaluate half the maximum QD dose used in Cohort 3 administered twice a day for 14 days. Alternatively, if Cohort 3 of Part C is dosed BID, then the planned doses will not exceed 400mg BID, and Cohort 4 will evaluate the maximum total daily dose used in Part C administered QD for 14 days.

Figure 3 Part C Schematic of Design

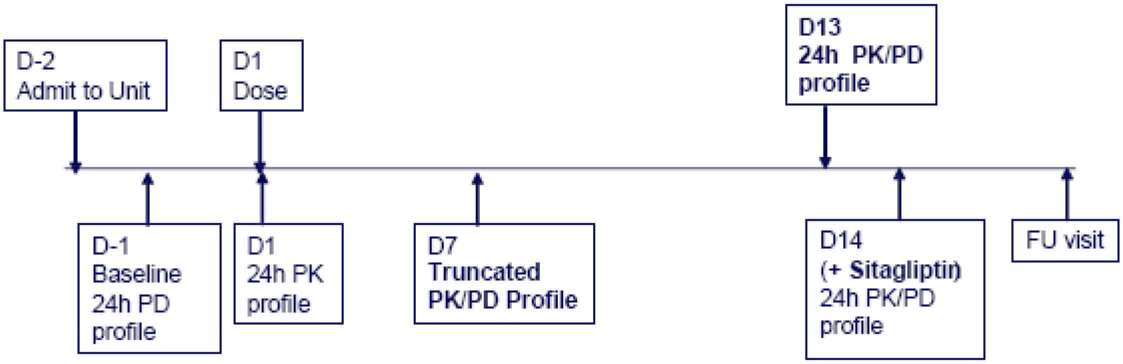

As shown in Figure 3, subjects will be admitted to the research facility within 48 hours (Day -2) of first dose (Day 1). The 24-hour time period prior to dosing (Day-1) will be defined as the baseline, and the treatment period will be defined as Days 1-14. **On Day 14, subjects randomized to GSK1292263 or placebo (QD or BID) will also receive a single dose of 100mg sitagliptin.** Full 24hr profiles for PK will be conducted on Days 1**,**

13 and 14 **for GSK1292263. A full 24 hr PK profile will be conducted on Day 14 for sitagltipin. Full** 24hr profiles for PD will be conducted on Days -1**, 13** and 14. **Truncated PK and PD profiles will be obtained on Day 7 to evaluate the time-course of the PD changes.**

Section 4.1.6.3 Part C (Treatment Regimen)

PREVIOUS TEXT

Subjects will be randomized to 14 days of dosing with three once-daily dose levels of GSK1292263 (planned doses: 25, 150, 400mg) or matching placebo, or open-label sitagliptin 100mg. Subjects will check into the unit on Day -2, followed by 24h PK assessments on Days 1, 7 and 14, and PD assessments on Days -1, 7 and 14.

REVISED TEXT

Subjects will be randomized to 14 days of dosing with three ~~once-daily~~ dose levels of GSK1292263 (planned **QD** doses: 25, 150, 400mg**; planned BID doses not to exceed 400mg BID)** or matching placebo, or open-label sitagliptin 100mg. **On Day 14, subjects randomized to GSK1292263 or placebo will also receive a single dose of 100mg sitagliptin.** Subjects will check into the unit on Day -2, followed by 24h PK assessments on Days 1, 13 and 14, and PD assessments on Days -1**, 13** and 14. **Truncated PK and PD profiles will also be obtained on Day 7. PK samples will be collected for all subjects and will be analyzed for GSK1292263 concentrations on Days 1, 7 and 13. PK samples will be analyzed for both sitagliptin and GSK1292263 concentrations on Day 14... Sitagliptin PK will be measured only on Day 14 for subjects in the sitagliptin arm**.

If GSK1292263 is dosed in a BID regimen in Part C (Cohort 3 or 4), on Day 14 sitagliptin will be dosed QD with the morning dose of GSK1292263.

Section 4.1.8.2. Meal Tolerance test (MTT) – Part C only

ADDED TEXT

On Days -1**, 7, 13** and 14 in Part C, subjects will be administered a standardized MTT at breakfast time. Blood samples will be collected up to 180min so that the first and second phases of insulin secretion can be derived by modelling the C-peptide and insulin kinetics data during the MTT. In addition, insulin sensitivity may be calculated from the rate of appearance and disappearance of the ingested glucose in the meal. **Limited PD and PK sampling will be conducted on Day 7.**

Section 4.1.8.3. Hunger, Satiety and Caloric Intake (Part C)

ADDED TEXT

The modified HCFQ will be administered on Days -1, 7**, 13** and 14 (at the same time of day, ±1 hour). Any subject who withdraws from the study should complete the modified HCFQ at withdrawal.

If feasible, on Days -1, 7**, 13** and 14, calorie counts will be obtained across the meal periods and during the overall 24h.

Section 4.1.8.4. Other Pharmacodynamic Profiling

PREVIOUS TEXT

In Part C, assessment of 24-hour glucose, insulin, C-peptide, glucagon, GLP-1 (total and active), GIP, and PYY (total) at baseline (Day -1), and on Days and 14 will allow evaluation of the pharmacodynamic effect of repeat-dosing of GSK1292263 versus placebo and sitagliptin, and will permit comparison of the three dose levels (low, medium, high) of GSK1292263.

REVISED TEXT

In Part C, assessment of 24-hour glucose, insulin, C-peptide, glucagon, GLP-1 (total and active), GIP, and PYY (total) at baseline (Day -1), and on Days **7** and 1**3** will allow evaluation of the pharmacodynamic effect of repeat-dosing of GSK1292263 versus placebo and sitagliptin, and will permit comparison of the three dose levels (low, medium, high) of GSK1292263. **In addition, the effect of adding sitagliptin to GSK1292263 will be assessed primarily by comparing the responses on Day 13 and Day 14, but further information may be forthcoming by comparing Day -1 and Day 14.**

Section 4.1.8.5. Exploratory Biomarkers

ADDED TEXT

In Part C, samples will be collected on Days -1, 7**, 13** and 14. Details for sample collection and processing of samples can be found in the SPM. The timing of the collections may be adjusted on the basis of emerging PK or PD data from this study or other new information in order to ensure optimal evaluation of the PD.

Section 4.2. Treatment Assignment

PREVIOUS TEXT

In Part C, subjects will be randomized to receive an active treatment (GSK1292263 25, 100 or 400mg or sitagliptin 100mg) or placebo in a 1:1:1:1:1 ratio, or a ratio of 1:1:1:1:1:1 if optional Cohort 4 is used to test a BID dosing regimen of GSK1292263.

REVISED TEXT

In Part C, subjects will be randomized to receive an active treatment **in Cohort 3 (3 dose levels of** GSK1292263 ~~25, 100 or 400mg~~ or sitagliptin 100mg) or placebo **or GSK1292263 in a QD or BID regimen in Cohort 4** in a ~~1:1:1:1:1 ratio, or a~~ ratio of 1:1:1:1:1:1 ~~if optional Cohort 4 is used to test a BID dosing regimen of GSK1292263~~. **Appropriate adjustment will be made to prevent an enrollment bias based on prior anti-diabetic treatment.**

Section 4.4. Dose Adjustment/Stopping Criteria

PREVIOUS TEXT

For Part C, the doses of GSK1292263 will be 25, 100 and 400mg, and these doses will be confirmed or changed based on safety/tolerability data (including clinically significant abnormalities of clinical labs, vital signs, and ECGs), and PK and PD data from Part A and B. The actual doses to be administered or already being administered in Part C may be adjusted based on safety and tolerability and emergent preliminary pharmacokinetic and/or pharmacodynamic data. These dose adjustments may involve either an increase or a decrease in the planned dose, but all doses will be selected so as not to exceed the mean or individual plasma exposures as noted above.

REVISED TEXT

For Part C, ~~the doses of GSK1292263 will be 25, 100 and 400mg, and~~ **subjects in Cohort 3 will be randomized to received planned doses of 25mg GSK1292263 QD, 150mg QD and 800mg QD. Alternatively, GSK1292263 may be administered BID in 3 treatment arms at doses that do not exceed 400mg BID, for comparison to placebo (administered BID) and open label sitagliptin administered QD. The actual doses to be administered in Cohort 3 may be modified based on preliminary PK/PD, safety and tolerability data from Parts A and B. Final doses will be selected which are predicted to maintain exposures below nonclinical toxicology limits.**

Cohort 4 of Part C will be conducted to assess the PK and PD of BID or QD dosing of GSK1292263 in T2DM subjects after 14-days of dosing, as well as the safety, tolerability, PK and PD of a 100mg single dose of open-label sitagliptin co-administered with GSK1292263 on Day 14. If GSK1292263 is dosed QD in Cohort 3 of Part C, Cohort 4 will evaluate half the maximum QD dose used in Cohort 3 administered twice a day for 14 days. Alternatively, if Cohort 3 of Part C is dosed BID, then Cohort 4 will evaluate the maximum total daily dose used in Part C administered QD for 14 days.

Doses in Part C (Cohorts 3 and 4) will be confirmed or changed based on safety/tolerability data (including clinically significant abnormalities of clinical labs, vital signs, and ECGs), and PK and PD data from Part A and B. The actual doses to be administered or already being administered in Part C may be adjusted based on safety and tolerability and emergent preliminary pharmacokinetic and/or pharmacodynamic data. These dose adjustments may involve either an increase or a decrease in the planned dose, but all doses will be selected so as not to exceed **800mg total daily dose of GSK1292263 and/or** the mean or individual plasma exposures **defined by the non-clinical toxicology studies** ~~as noted above~~.

Section 4.6. Time and Events Table (Parts A and B)

PREVIOUS TEXT

1. Screening visit must be performed within 28 days of Day 1.
2. Assessment of vital signs (including blood pressure and heart rate) will be performed at one time point at Screening, at follow-up and on Day -1 of each period. On Day 1, they will be taken at pre-dose, 1 hour, 3, 4, 6, 10, 16 and 24hours. Assessments should be made in triplicate at each pre-dose time point, and single assessments should be made at all other times. Assessments should be performed after resting in a supine or semi-supine position for at least 10 minutes.
3. ECGs will be taken at Screening, on Day 1 at pre-dose, 1 hour, 2, 3, 4, 6, 10, 16, 24hours and at follow-up. Assessments should be made in triplicate at each pre-dose time point, and single assessments should be made at all other times. ECGs should be taken while subject is supine. Additional ECGs may be taken at the discretion of the investigator as needed based on symptoms or telemetry findings.
4. Telemetry will be performed for 24 hours on Day 1 of each period, starting 1hr prior to dosing.
5. Blood samples for safety will be collected at screening, pre-dose (Day -1), 24hr post- dose for each period, and at follow-up. When this results in multiple samples at the same time point, only one sample will be collected (e.g., 24hrs post-first-dose = pre-dose (time 0) for the second dose. Refer to Section 7.2.4 for specific laboratory parameters to be tested.
6. FSH and Estradiol tests will be performed for all post-menopausal women.
7. In Part A study drug will be administered 2h before the OGTT. In Part B study drug will be administered 30 min after breakfast.
8. Blood samples for the determination of glucose and insulin will be collected at pre-dose on Day 1 of each dosing period,, and immediately prior to and at 10, 20, 30, 60, 90, 120, 180min after administration of the 75g glucose drink in Part A. For breakfast, lunch and evening meal in Part B and lunch and evening meal in Part A, samples will be collected just before the meal and at the following times after starting each meal: 0.5, 1, 1.5, 2 and 3 hours. When this results in multiple samples at the same time point, only one sample will be collected (e.g., 24hrs post-first-dose = pre-dose (time 0) for the second dose. Refer to the Study Procedures Manual for additional details on the collection and processing of PD samples.
9. If blood volume permits, a blood sample for additional biomarkers will be collected pre-dose on Day 1 of each dosing period,, and immediately prior to and at 30, 60, 90 and 120 after administration of the 75g glucose drink in Part A. For breakfast, lunch and evening meal in Part B and lunch and evening meal in Part A, samples will be collected just before the meal and at the following times after starting each meal: 0.5, 1, 1.5 and 2 hours. When this results in multiple samples at the same time point, only one sample will be collected (e.g., 24hrs post-first-dose = pre-dose (time 0) for the second dose. Refer to the Study Procedures Manual for additional details on the collection and processing of these samples.
10. Subjects washing out of fibrates should be instructed to do so beginning on Day -14, but no visit occurs at that time.
11. Blood samples for the determination of PK will be collected at the following times (PK sample times may be changed based on observed PK profile, but the total number of samples will not change) on Day 1 of each period: Immediately pre-dose (time 0) and at 0.5, 1, 2, 3, 4, 6, 8, 14, and 24 hours (when this results in multiple samples at the same time point, only one sample will be collected (e.g., 24hrs post-first-dose = pre-dose (time 0) for the second dose). Refer to the Study Procedures Manual for details on the collection and processing of PK samples.
12. Subjects should be checked by investigational staff during the day and night to ensure there are no findings consistent with hypoglycemia (e.g., cool, moist skin, and diaphoresis)

REVISED TEXT

1. Screening visit must be performed within 28 days of Day 1.
2. Assessment of vital signs (including blood pressure and heart rate) will be performed at one time point at Screening, at follow-up and on Day -1 of each period. On Day 1, they will be taken at pre-dose, 1 hour, 3, 4, 6, 10, 16 and 24hours. Assessments should be made in triplicate at each pre-dose time point, and single assessments should be made at all other times. Assessments should be performed after resting in a supine or semi-supine position for at least 10 minutes.
3. ECGs will be taken at Screening, on Day 1 at pre-dose, 1 hour, 2, 3, 4, 6, 10, 16, 24hours and at follow-up. Assessments should be made in triplicate at each pre-dose time point, and single assessments should be made at all other times. ECGs should be taken while subject is supine. Additional ECGs may be taken at the discretion of the investigator as needed based on symptoms or telemetry findings.
4. Telemetry will be performed for 24 hours on Day 1 of each period, starting 1hr prior to dosing.
5. Blood samples for safety will be collected at screening, pre-dose (Day -1), 24hr post- dose for each period, and at follow-up. When this results in multiple samples at the same time point, only one sample will be collected (e.g., 24hrs post-first-dose = pre-dose (time 0) for the second dose. Refer to Section 7.2.4 for specific laboratory parameters to be tested.
6. FSH and Estradiol tests will be performed for all post-menopausal women.
7. In Part A study drug will be administered 2h before the OGTT. In Part B study drug will be administered 30 min after breakfast and lunch and dinner will be approximately 4 and 10 hours after dosing
8. Blood samples for the determination of glucose and insulin will be collected at pre-dose on Day 1 of each dosing period,, and immediately prior to and at 10, 20, 30, 60, 90, 120, 180min after administration of the 75g glucose drink in Part A. For breakfast, lunch and evening meal in Part B and lunch and evening meal in Part A, samples will be collected just before (Part A)/after (Part B) the meal and at the following times after starting each meal: 0.5, 1, 1.5, 2 and 3 hours. Samples will also be collected in Part B at 24hrs post-dose. When this results in multiple samples at the same time point, only one sample will be collected (e.g., 24hrs post-first-dose = pre-dose (time 0) for the second dose. Refer to the Study Procedures Manual for additional details on the collection of PD samples in relation to meals, and processing of PD samples.
9. If blood volume permits, a blood sample for additional biomarkers will be collected pre-dose on Day 1 of each dosing period,, and immediately prior to and at 30, 60, 90 and 120 after administration of the 75g glucose drink in Part A. For breakfast, lunch and evening meal in Part B and lunch and evening meal in Part A, samples will be collected just before (Part A)/after (Part B) the meal and at the following times after starting each meal: 0.5, 1, 1.5 and 2 hours. Samples will also be collected in Part B at 24hrs post-dose. When this results in multiple samples at the same time point, only one sample will be collected (e.g., 24hrs post-first-dose = pre-dose (time 0) for the second dose. Refer to the Study Procedures Manual for additional details on the collection of samples in relation to meals, and processing of these samples.
10. Subjects washing out of fibrates should be instructed to do so beginning on Day -14, but no visit occurs at that time.
11. Blood samples for the determination of PK will be collected at the following times (PK sample times may be changed based on observed PK profile, but the total number of samples will not change) on Day 1 of each period: Immediately pre-dose (time 0) and at 0.5, 1, 2, 3, 4, 6, 8, 14 (for Part B, sample should be collected at 13hrs, not 14hrs), and 24 hours (when this results in multiple samples at the same time point, only one sample will be collected (e.g., 24hrs post-first-dose = pre-dose (time 0) for the second dose). Refer to the Study Procedures Manual for details on the collection of PK samples in relation to meals, and processing of PK samples.
12. Subjects should be checked by investigational staff during the day and night to ensure there are no findings consistent with hypoglycemia (e.g., cool, moist skin, and diaphoresis)

Section 4.6. Time and Events Table (Part C)

PREVIOUS TEXT

Procedure	Screening ≤28 days prior to first dose	Period 1								Follow-up
		Days -14 to Day -3	Day -2	Day -1	Day 1	Days 2-6	Day 7	Days 8-13	Day 14	7 -10 days after discharge
Clinic Visit	X	Washout	---In Clinic---							X
Informed Consent	X									
Inclusion/ Exclusion Criteria	X									
Demographics	X									
Complete physical	X									
Check into Clinic			X							
Brief physical			X							X
Medical/medication/ drug /alcohol history	X									
Weight	X				X					X
Waist Circumference					X					
Height / BMI Calculation	X									
12-lead ECG[1]	X				X		X		X	X
24-hr Holter	X									
Vitals[2]	X				X	X	X	X	X	X
Urine drug/alcohol screen	X			X						
Urine pregnancy test				X						X
Clinical Chemistry / Hematology / Urinalysis[3]	X			X			X		X	X
FSH/ Estradiol[4]	X									
HIV/ Hep B / Hep C	X									
Washout / Glucometer		X	X	X						
PGx[6]					X					
Dosing – Randomized study med[7]					X	X	X	X	X	
Mixed Meal Tolerance Test [8]				X			X		X	
Modified HCFQ [9]				X			X		X	
Calorie counts				X			X		X	
Standardized meals				X	X	X	X	X	X	
Blood samples for glucose and insulin[10]				X			X		X	
Blood samples for peptides.[10]				X			X		X	
Additional biomarker blood samples[11]				X			X		X	
PK profile blood samples[12]					X		X		X	

Procedure	Screening ≤28 days prior to first dose	Period 1								Follow-up
		Days -14 to Day -3	Day -2	Day -1	Day 1	Days 2-6	Day 7	Days 8-13	Day 14	7 -10 days after discharge
PK Trough Samples[12]						X				
Resume Washed-out Meds[13]									X	
Concomitant Medication Review	X	X	X	X	X	X	X	X	X	X
AE assessment			X	X	X	X	X	X	X	X

1. Single ECGs will be taken at Screening, on Day -1 before breakfast, on Days 1, 7 and 14 at pre-dose, 1, 3, 9, 12 and 24hour, and at Follow-up. ECGs should be taken while subject is supine. Additional ECGs may be taken at the discretion of the investigator as needed based on symptoms or ECG findings.
2. Assessment of vital signs (including blood pressure and heart rate) will be performed at Screening, on Day -1, on each in-house day pre-dose, and at Follow-up. On Days 1, 7 and 14, they will be taken at pre-dose, 1, 3, 9, 12 and 24 hours. At each time point, assessment should be performed after resting in a supine or semi-supine position for at least 10 minutes.
3. Blood samples for safety will be collected at screening, pre-dose (Day -1), Day 7, Day 14, and at follow-up. When this results in multiple samples at the same time point, only one sample will be collected (e.g., 24hrs post-first-dose = pre-dose (time 0) for the second dose). Refer to Section 7.2.4 for specific laboratory parameters to be tested.
4. FSH and Estradiol tests will be performed for all post-menopausal women.
5. Subjects washing out of fibrates should be instructed to do so beginning on Day -14, but no visit occurs at that time.
6. PGx sample may be taken at any time after the first dose.
7. For QD dosing, study drug will be administered 30 min before breakfast. If optional Cohort 4 is conducted, dosing will occur 30min before breakfast and 30min before the evening meal.
8. Composition of the meal tolerance test is specified in the SPM.
9. Modified HCFQ is administered at the same time of day, ±1 hour.
10. Blood samples for the determination of glucose and insulin will be collected at pre-dose on Days -1, 7 and 14, and then immediately prior to and at 10, 20, 30, 60, 90, 120, 180min after eating the standardised breakfast meal tolerance test. For lunch and the evening meal, samples will be collected just before the meal and at the following times after starting each meal: 0.5, 1, 1.5, 2 and 3 hours. When this results in multiple samples at the same time point, only one sample will be collected (e.g., 24hrs post-first-dose = pre-dose (time 0) for the second dose). Refer to the Study Procedures Manual for additional details on the collection and processing of PD samples.
11. If blood volume permits, blood samples for the determination of additional biomarkers will be collected at pre-dose on Days -1, 7 and 14, and then immediately prior to and at 30, 60, 90 and 120miin after eating the standardised breakfast meal tolerance test. For lunch and the evening meal, samples will be collected just before the meal and at the following times after starting each meal: 0.5, 1, 1.5 and 2 hours. When this results in multiple samples at the same time point, only one sample will be collected (e.g., 24hrs post-first-dose = pre-dose (time 0) for the second dose). Refer to the Study Procedures Manual for additional details on the collection and processing of PD samples.
12. Blood samples for the determination of PK will be collected at the following times (PK sample times may be changed based on observed PK profile, but the total number of samples will not change) on Days 1, 7 and 14: Immediately pre-dose (time 0) and at 0.5, 1, 1.5, 2, 3, 4, 6, 8, 14 and 24 hours post-dose, (when this results in multiple samples at the same time point, only one sample will be collected (e.g., 24hrs post-first-dose = pre-dose (time 0) for the second dose). Trough samples for PK will be collected immediately pre-dose on Days 3, 4, 5 and 6. Refer to the Study Procedures Manual for details on the collection and processing of PK samples. If optional Cohort 4 is conducted, blood samples for PK sampling will be collected at immediately pre-dose, 1, 2, 4, 6, 8, 12, 13, 14, 16, 18, 20 and 24hrs post-dose.
13. If appropriate, the PI has the option of adjusting doses of washed-out medications when they are resumed. Review of glucose results should occur, and doses of oral anti-diabetic medications adjusted based on pharmacologic effects of treatment observed during the study.

REVISED TEXT

Procedure	Screening ≤28 days prior to first dose	Period 1										Follow-up
		Days -14 to Day -3	Day -2	Day -1	Day 1	Days 2-6	Day 7	Days 8-12	Day 13	Day 14	Day 15	7 -10 days after discharge
Clinic Visit	X	Washout[5]	---In Clinic---									X
Informed Consent	X											
Inclusion/ Exclusion Criteria	X											
Demographics	X											
Complete physical	X											
Check into Clinic			X									
Brief physical			X									X
Medical/medication/ drug /alcohol history	X											
Weight	X				X							X
Waist Circumference					X							
Height / BMI Calculation	X											
12-lead ECG[1]	X			**X**	X		X		**X**	X	**X**	X
24-hr Holter	X											
Vitals[2]	X			**X**	X	X	X	X	**X**	X	**X**	X
Urine drug/alcohol screen	X			X								
Urine pregnancy test				X								X
Clinical Chemistry / Hematology / Urinalysis[3]	X			X			X			X	**X**	X
FSH/ Estradiol[4]	X											
HIV/ Hep B / Hep C	X											
Washout / Glucometer		X	X	X								
PGx[6]					X							
Dosing – Randomized study med[7]					X	X	X	X	**X**	X		
Mixed Meal Tolerance Test [8]				X			X		**X**	X		
Modified HCFQ [9]				X			X		**X**	X		
Calorie counts				X			X		**X**	X		
Standardized meals				X	X	X	X	X	**X**	X		
Blood samples for glucose and insulin/**PD**[10]				X			X		**X**	X	**X**	
PK profile blood samples[11]					X		X		**X**	X	**X**	
PK Trough Samples[11]						**X**	**X**					
Resume Washed-out Meds[12]											**X**	

Procedure	Screening ≤28 days prior to first dose	Period 1										Follow-up
		Days -14 to Day -3	Day -2	Day -1	Day 1	Days 2-6	Day 7	Days 8-12	Day 13	Day 14	Day 15	7 -10 days after discharge
**Checkout from clinic**											**X**	
Concomitant Medication Review	X	X	X	X	X	X	X	X	**X**	X	**X**	X
AE assessment			X	X	X	X	X	X	**X**	X	**X**	X

1. Single ECGs will be taken at Screening, on Day -1 before breakfast, on Days 1, 7, **13** and 14 at pre-dose **(time 0),** 1, 3, 9, 12 and 24hour, and at Follow-up. ECGs should be taken while subject is supine. Additional ECGs may be taken at the discretion of the investigator as needed based on symptoms or ECG findings.
2. Assessment of vital signs (including blood pressure and heart rate) will be performed at Screening, on Day -1, on each in-house day pre-dose, and at Follow-up. On Days 1, 7, **13** and 14, they will be taken at pre-dose **(time 0),** 1, 3, 9, 12 and 24 hours. At each time point, assessment should be performed after resting in a supine or semi-supine position for at least 10 minutes.
3. Blood samples for safety will be collected at screening, pre-dose (Day -1**, time 0**), Day 7**, Day 14** and Day 1**5 (prior to checkout, =24hrs post-dose)**, and at follow-up. When this results in multiple samples at the same time point, only one sample will be collected (e.g., **when** 24hrs post-first-dose = pre-dose (time 0) for the second dose). Refer to Section 7.2.4 for specific laboratory parameters to be tested.
4. FSH and Estradiol tests will be performed for all post-menopausal women.
5. Subjects washing out of fibrates should be instructed to do so beginning on Day -14, but no visit occurs at that time.
6. PGx sample may be taken at any time after the first dose.
7. For QD dosing, study drug will be administered **immediately after** breakfast. **For BID dosing study drug will be administered immediately after breakfast and immediately after the evening meal but prior to the 10hr PK sample (see Study Procedures Manual for details of sampling times relative to dosing times)**,
8. Composition of the meal tolerance test is specified in the SPM.
9. Modified HCFQ is administered at the same time of day, ±1 hour.
10. Blood samples for the determination of glucose and insulin **and other PD markers** will be collected at pre-dose **(time 0)** on Days -1**, 13** and 14, and then immediately prior to and at 10, 20, 30, 60, 90, 120, 180min after eating the standardised breakfast meal tolerance test. For lunch **(approximately 4h post morning dose)** and the evening meal **(approximately 10h post morning dose),** samples will be collected just before the meal and at the following times after starting each meal: 0.5, 1, 1.5, 2 and 3 hours. **A sample will also be collected 24 hours post-dose. On Day 7, samples for glucose and insulin and other PD markers will be collected at predose (= pre- breakfast), 1, 2, 4 (= pre lunch), 6, 10 and 12h, if blood volume permits.** When this results in multiple samples at the same time point, only one sample will be collected (e.g., 24hrs post-first-dose = pre-dose (time 0) for the second dose). Refer to the Study Procedures Manual for additional details on the collection and processing of PD samples.
11. Blood samples for the determination of PK will be collected at the following times (PK sample times may be changed based on observed PK profile, but the total number of samples will not change) on Days 1, **13** and 14: Immediately pre-dose (time 0) and at 0.5, 1, 1.5, 2, 3, 4, 6, 8, 14 and 24 hours post-dose, (when this results in multiple samples at the same time point, only one sample will be collected (e.g., 24hrs post-first-dose = pre-dose (time 0) for the second dose). **On Day 7, samples for PK will be collected at predose (=pre- breakfast), 1, 2, 4 (= pre lunch), 6, 10 (= immediately post-dinner) and 12h**. Trough samples for PK will be collected immediately pre-dose on Days 4, 5, 6 **and 7** . Refer to the Study Procedures Manual for details on the collection and processing of PK samples. **When GSK1292263 is dosed BID in Cohorts 3 or** 4 blood samples for PK sampling will be collected at immediately pre-dose, 1, 2, 4, 6, 8, **10, 11, 12, 14, 16,**18, and 24hrs post-dose.
12. If appropriate, the PI has the option of adjusting doses of washed-out medications when they are resumed. Review of glucose results should occur, and doses of oral anti-diabetic medications adjusted based on pharmacologic effects of treatment observed during the study.

Section 5.1. Number of Subjects

PREVIOUS TEXT

Approximately 88 subjects will be enrolled (12 in Part A ([Cohort 1], 4 in Part B [Cohort 2], and 72 in Part C [Cohort 3]) and complete dosing and critical assessments. If optional Cohort 4 is employed, then a further 12 subjects will be enrolled. Additional subjects/cohorts may be enrolled to allow for evaluation of additional dose levels.

For Part C, 12 subjects will be randomized into each of 5 treatment arms (planned doses 25, 100, 400mg GSK1292263, placebo or open-label sitagliptin). If safety, PK and PD data are satisfactory, an additional 4 subjects will be randomized into each of the three GSK1292263 treatment arms. If justified by emergent PK-PD data from Parts A, B or C, 12 subjects will be randomized into an additional optional Cohort 4 (BID regimen). This will result in a total of 16 subjects in each of the once-daily GSK1292263 treatment arms, 12 in the placebo arm, 12 in the sitagliptin alone arm, and 12 in the BID GSK1292263 treatment arm, if the latter is conducted.

REVISED TEXT

Approximately **100** subjects will be enrolled (12 in Part A ([Cohort 1], 4 in Part B [Cohort 2], and **84** in Part C **[72 in** Cohort 3 **and 12 in Cohort 4**]) and complete dosing and critical assessments. Additional subjects/cohorts may be enrolled to allow for evaluation of additional dose levels.

For **Cohort 3 of** Part C, 12 subjects will be randomized into each of 5 treatment arms (planned **QD** doses 25, 1**5**0, **8**00mg GSK1292263 **or planned BID doses not to exceed 400mg BID**, placebo or open-label sitagliptin). If safety, PK and PD data are satisfactory, an additional 4 subjects will be randomized into each of the three GSK1292263 treatment arms. **Twelve** subjects will be randomized into Cohort 4 (**QD or** BID regimen **depending on the dosing regimen evaluated in Cohort 3**). This will result in a total of 16 subjects in each of the GSK1292263 treatment arms **in Cohort 3,** 12 in the placebo arm, 12 in the sitagliptin alone arm, and 12 in the GSK1292263 treatment arm **in Cohort 4.**

Section 5.2.1. Inclusion Criteria

PREVIOUS TEXT

6. For Parts B and C: T2DM diagnosed by American Diabetes Association criteria for at least 3 month prior to Screening:
   - Controlled by diet and exercise, or, if on medication, subjects must be treating their T2DM using one of the following regimens:
     - Metformin as monotherapy
     - Sulfonylurea as monotherapy
     - Metformin and sulfonylurea in combination, if both components are being administered at doses that are half their maximum dose or less

- ❖ DPP-IV inhibitors, either as monotherapy or in combination with other agent(s) on this list at half maximal dose or less
- ❖ Exenatide, either as monotherapy or in combination with other agent(s) on this list at half maximal dose or less

For subjects that are being screened for Parts B and C, all doses of anti-diabetic medication must have been stable for at least 3 months prior to Screening, and the subject must be willing to wash out from their anti-diabetic medications from Day -7 through post-last-dose of Period 2 (Part B) or Day -7 through Day 15 (Part C).

- Fasting plasma glucose (FPG) level ≤ 220mg/dL at the Screening visit
- FPG level ≤ 250mg/dL on Day -1
- HbA1c between 7 and 11%, inclusive, at Screening visit

For subjects that are being screened for Parts B and C, all doses of anti-diabetic medication must have been stable for at least 3 months prior to Screening, and the subject must be willing to wash out from their anti-diabetic medications from Day -7 through post-last-dose of Period 2 (Part B) or Day -7 through Day 15 (Part C).

REVISED TEXT

6. For Part~~s~~ B ~~and C~~: T2DM diagnosed by American Diabetes Association criteria for at least 3 month prior to Screening:
   - Controlled by diet and exercise, or, if on medication, subjects must be treating their T2DM using one of the following regimens:
     - ❖ Metformin as monotherapy
     - ❖ Sulfonylurea as monotherapy
     - ❖ Metformin and sulfonylurea in combination, if both components are being administered at doses that are half their maximum dose or less
     - ❖ DPP-IV inhibitors, either as monotherapy or in combination with other agent(s) on this list at half maximal dose or less
     - ❖ Exenatide, either as monotherapy or in combination with other agent(s) on this list at half maximal dose or less

   For subjects that are being screened for Part B, all doses of anti-diabetic medication must have been stable for at least 3 months prior to Screening, and the subject must be willing to wash out from their anti-diabetic medications from Day -7 through post-last-dose of Period 2.

   - **Fasting plasma glucose (FPG) level ≤ 220mg/dL at the Screening visit**
   - **FPG level ≤ 250mg/dL on Day -1**
   - **HbA1c between 7 and 11%, inclusive, at Screening visit**

   For Part C: T2DM diagnosed by American Diabetes Association criteria for at least 3 month prior to Screening:

- **Controlled by diet and exercise, or, if on medication, subjects must be treating their T2DM using one of the following regimens:**
  - ❖ **Metformin as monotherapy**
  - ❖ **Sulfonylurea as monotherapy**
  - ❖ **DPP-IV inhibitors as monotherapy**
  - ❖ **Exenatide as monotherapy**

Note: For subjects that are being screened for Part~~s B and~~ C, all doses of anti-diabetic medication must have been stable for at least 3 months prior to Screening, and the subject must be willing to wash out from their anti-diabetic medications from ~~Day -7 through post-last-dose of Period 2 (Part B) or~~ Day -7 through Day 15 ~~(Part C)~~.

Section 5.2.2. Exclusion Criteria

ADDED TEXT

3. …

- Urine protein/creatinine (mg of protein/mg of creatinine) ratio >2.5; **or** urine albumin **or protein** concentration >300mg/g of creatinine.

Section 6.1. Hypotheses and Treatment Comparisons

PREVIOUS TEXT

Repeat-dose PK parameters [AUC(0-τ)] will be compared to single-dose PK parameters to assess accumulation [AUC(0-τ)] and time-invariance [AUC(0-∞)] (as data permit). Point estimates and 90% confidence intervals will be presented for each of the comparisons.

Point estimates and 90% confidence intervals for the slope for day from the analysis of GSK1292263 trough concentration (Cτ) will be calculated to assess achievement of steady state at each dose level.

Fasted and derived PD parameters will be compared between each GSK1292263-treated group and placebo and between GSK1292263 and sitagliptin in both single dose and repeat dose parts of the study. Point estimates and 95% confidence intervals will be presented for each of the differences (GSK1292263-placebo and GSK1292263-sitagliptin).

REVISED TEXT

Repeat-dose **(QD or BID)** PK parameters [AUC(0-τ)] will be compared to single-dose PK parameters to assess accumulation [AUC(0-τ)] and time-invariance [AUC(0-∞)] (as data permit). **AUC(0-τ), AUC(0-24) and Cmax will be calculated on Days 13 and 14, as data permit, to assess the effect of sitagliptin on GSK1292263 PK (Day 14 versus Day 13). Point estimates and 90% confidence intervals will be presented for each of the comparisons.**

AUC(0-24) and Cmax will be calculated, as data permit, for sitagliptin on Day 14 to assess the effect of GSK1292263 on sitagliptin PK (sitagliptin alone versus sitagliptin co-administered with GSK1292263). Point estimates and 90% confidence intervals will be presented for each of the comparisons.

Point estimates and 90% confidence intervals for the slope for day from the analysis of GSK1292263 trough concentration (Cτ) will be calculated to assess achievement of steady state at each dose level.

Fasted and derived PD parameters will be compared between each **of the following during the repeat dose part of the study (Part C):**

- GSK1292263 **Day 13 versus Day -1**
- **GSK1292263+Sitagliptin Day 14 versus GSK1292263 alone Day 13**
- **GSK1292263 Day 13 versus Placebo Day 13 (change from baseline)**
- **Sitagliptin Day 13 versus Placebo Day 13 (change from baseline)**
- **Sitagliptin Day 13 and Day 14 versus Day -1**
- **GSK1292263+Sitagliptin Day 14 versus Placebo+Sitagliptin Day 14 (change from baseline)**
- **GSK1292263 Day 7 versus Placebo Day 7**
- **Sitagliptin Day 7 versus Placebo Day 7**

In the single dose part of the study (Part A), fasted and derived PD parameters will be compared between each GSK1292263-treated group and placebo and between GSK1292263 and sitagliptin Point estimates and 95% confidence intervals will be presented for each of the differences (GSK1292263-placebo and GSK1292263-sitagliptin).

Section 6.2.1. Sample Size Assumptions

ADDED TEXT

…Except for Part B, this study is targeted to have 12 subjects complete each treatment group. **A further 4 subjects may be enrolled into each of the 3 GSK1292263 arms of Cohort 3…**

Section 6.3.2.2. Pharmacokinetic Analyses

PREVIOUS TEXT

Following repeat dosing in Part C:

Cmax, tmax, area under the plasma concentration-time curve over the dosing interval [AUC(0-τ)], pre-dose (trough) concentration at the end of the dosing interval (Cτ), and t½.

AUC(0-∞), and Cmax following single dosing on Day 1 and AUC(0-τ), Cτ, and Cmax following repeat dosing will be used for assessment of dose proportionality of GSK1292263. Trough concentration (Cτ) samples collected on Days 3, 4, 5, 6 will be used to assess attainment of steady state for GSK1292263. To estimate the extent of accumulation and assess time-invariance of GSK1292263 after repeat dosing, the observed accumulation ratio (Ro) and time-invariance ratio (Rs) will be determined. If AUC(0-∞) on Day 1 can not be accurately estimated due to inadequate sampling periods and large extrapolated areas, the dose proportionality assessment will not include AUC(0-∞) and the time-invariance ratio will not be provided.

REVISED TEXT

Following repeat dosing in Part C:

Cmax, tmax, area under the plasma concentration-time curve over the dosing interval **[AUC(0-τ), AUC(0-24)] and AUC(0-12) on Day 7**, pre-dose (trough) concentration at the end of the dosing interval (Cτ), and t½ **will be estimated for GSK1292263, as data permit**.

Cmax, tmax, and area under the plasma concentration-time curve over the dosing interval [AUC(0-24)] on Day 14, will be estimated for sitagliptin (alone and in combination with GSK1292263), as data permit.

AUC(0-∞), and Cmax following ~~single~~ dosing on Day 1 and AUC(0-τ), Cτ, and Cmax following repeat dosing will be used for assessment of dose proportionality of GSK1292263. Trough concentration (Cτ) samples collected on Days ~~3,~~ 4, 5, 6 **and 7** will be used to assess attainment of steady state for GSK1292263. To estimate the extent of accumulation and assess time-invariance of GSK1292263 after repeat dosing, the observed accumulation ratio (Ro) and time-invariance ratio (Rs) will be determined. If AUC(0-∞) on Day 1 can not be accurately estimated due to inadequate sampling periods and large extrapolated areas, the dose proportionality assessment will not include AUC(0-∞) and the time-invariance ratio will not be provided.

AUC(0-τ), AUC(0-24), and Cmax on Days 13 and 14 will be used to assess the effect of sitagliptin on GSK1292263 pharmacokinetics, as data permit.

ADDED TEXT

AUC(0-τ), AUC(0-24), and Cmax on Days 13 and 14 for GSK1292263 will be used to assess the effect of sitagliptin on GSK1292263 pharmacokinetics, as data permit.

AUC(0-24) and Cmax on Day 14 for sitagliptin (alone and when co-administered with GSK1292263) will be used to assess the effect of GSK1292263 on sitagliptin pharmacokinetics, as data permit.

Section 6.3.2.4. Pharmacodynamic Analyses

ADDED TEXT

In Part C, an analysis of covariance (ANCOVA) with a fixed effect terms for treatment **and prior anti-diabetic treatment** will be fitted with the post-baseline weighted mean minus baseline (Day -1 weighted mean) as the dependent variable and the Day -1 weighted mean as a covariate. Pairwise differences in least squares means between each GSK1292263 active treatment and placebo will be calculated, and 95% confidence intervals will be constructed for these differences.

Section 8. LIFESTYLE AND/OR DIETARY RESTRICTIONS

PREVIOUS TEXT

Breakfast	Breakfast will be omitted on days when the OGTT will be administered (Part A). On Days -1, 7 and 14 the breakfast meal will be referred to as a meal tolerance test (MTT). See SPM for menu details.
Lunch	In Parts A and B, a standardized lunch will be fed on Day 1 of each period. In Part C, a standardized lunch will be consumed on Days -1 through 14.
Evening meal	In Parts A and B, a standardized evening meal will be fed on Day 1 of each period. In Part C, a standardized evening meal will be consumed on Days -1 through 14.
Evening Snack	No evening snack will be permitted on Day 1 in Part A, and Days -1, 7 and 14 in Part C. On other days, an evening snack will be permitted at approximately 12 - 14 hours after dosing and up to 23:00.
Alcohol, Caffeine, Xanthine	Subjects will not be allowed to consume alcohol or caffeine- or xanthine-containing products (e.g., coffee, tea, cola drinks, chocolate) for 24-hours prior to dosing until final discharge from the clinic (Day 2 of Period 5 in Part A, Day 2 of Period 2 in Part B, and Day 15 in Part C).
Nicotine	Subjects will not be allowed to smoke or use nicotine-containing products during the course of the study (Day -7 until after the final follow-up visit)

REVISED TEXT

Breakfast	Breakfast will be omitted on days when the OGTT will be administered (Part A). On Days -1, 7, 13 and 14 the breakfast meal will be referred to as a meal tolerance test (MTT). See SPM for menu details.
Lunch	In Parts A and B, a standardized lunch will be fed on Day 1 of each period. In Part C, a standardized lunch will be consumed on Days -1 through 14 at approximately 4h after the morning dosing.
Evening meal	In Parts A and B, a standardized evening meal will be fed on Day 1 of each period. In Part C, a standardized evening meal will be consumed on Days -1 through 14 at approximately 10h after the morning dose. For Cohort 4 (BID or QD dosing), see SPM for dosing in relation to meals and blood sampling.
Evening Snack	No evening snack will be permitted on Day 1 in Part A, and Days -1, 7 and 14 in Part C. On other days, an evening snack will be permitted at approximately 12 - 14 hours after dosing and up to 23:00.
Alcohol, Caffeine, Xanthine	Subjects will not be allowed to consume alcohol or caffeine- or xanthine-containing products (e.g., coffee, tea, cola drinks, chocolate) from 24-hours prior to dosing in Parts A and B or 7 days prior to dosing in Part C until final discharge from the clinic (Day 2 of Period 5 in Part A, Day 2 of Period 2 in Part B, and Day 15 in Part C).
Nicotine	Subjects will not be allowed to smoke or use nicotine-containing products during the course of the study (from Screening until after the final follow-up visit)

Section 12.2. Definition of Serious Adverse Events

ADDEDTEXT

h. ALT ≥3xULN and bilirubin ≥ 2xULN (see Section 13).

AMENDMENT 1

Where the Amendment Applies

All sites.

Summary of Amendment Changes with Rationale

This amendment adds criteria recommended by the IRB and alters Selection Criteria based on comments from the Investigator, in respons to recruitment problems.

List of Specific Changes

Section 1.2.1.2. Safety Overview (Data Currently Blinded to Principal Investigator)

PREVIOUS TEXT

Some episodes of dizziness were reported by the subjects during and after the OGTT, and on occasions these were associated with low blood glucose levels either on finger stick capillary blood measurement or formal laboratory analysis. Blood glucose levels <60mg/dL were defined by the Investigator as ‘hypoglycemia’ AEs. The episodes of ‘hypoglycemia’ were mild in all cases and resolved spontaneously without dose alteration or glucose rescue. These episodes we generally more frequent 2-4h after the start of the OGTT, and when the OGTT was administered 4h after dosing. The hypoglycemic events were reduced by (i) dosing the study drug earlier, (ii) administering the OGTT 2h after dosing, and (iii) feeding the subjects lunch at ~12:30. Some episodes of hypoglycemia occurred after the subjects had received placebo. Of note, there was no increased propensity to hypoglycemia when GSK1292263 250mg was co-administered with sitagliptin 100mg.

REVISED TEXT

Some episodes of dizziness were reported by the subjects during and after the OGTT, and on occasions these were associated with low blood glucose levels either on finger stick capillary blood measurement or formal laboratory analysis. Blood glucose levels <60mg/dL were defined by the Investigator as ‘hypoglycemia’ AEs. The episodes of ‘hypoglycemia’ were mild in all cases and resolved spontaneously without dose alteration or glucose rescue. These episodes we generally more frequent 2-4h after the start of the OGTT, and when the OGTT was administered 4h after dosing. The hypoglycemic events were reduced by (i) dosing the study drug earlier, (ii) administering the OGTT 2h after dosing, and (iii) feeding the subjects lunch at ~12:30. **There was no clear relationship to the dose of GSK1292263.** Some episodes of hypoglycemia occurred after the subjects had received placebo. Of note, there was no increased propensity to hypoglycemia when GSK1292263 250mg was co-administered with sitagliptin 100mg. ***[Note: there is error in Table 9 in the Investigator Brochure supplement that emerged during QC of the preliminary data – the number of adverse events of hypoglycemia in Part E should be 0, and not 5 events as in the table]***

Section 1.4.1. Study Rationale

PREVIOUS TEXT

This will be the first use of GSK1292263 in T2DM subjects, and is intended to assess the safety, tolerability, pharmacokinetic and pharmacodynamic profile of this investigational drug following single doses (Parts A [Cohort 1] and B [Cohort 2]), and subsequently 14 days of once-daily dosing (Part C, Cohort 3). In Parts A and C, open-label sitagliptin is included as a comparator to provide data that will aid the assessment of the therapeutic potential of GSK1292263. Part B is being conducted to provide an estimate, in T2DM, of the magnitude of the effect of food on the PK of GSK1292263 at the highest dose that

will be used in the study. If necessary, this will allow refinement of the doses selected for Part C, where the subjects will be receiving the study drug approximately 30min before a meal tolerance test (MTT) at breakfast time.

REVISED TEXT

This will be the first use of GSK1292263 in T2DM subjects, and is intended to assess the safety, tolerability, pharmacokinetic and pharmacodynamic profile of this investigational drug following single doses (Parts A [Cohort 1] and B [Cohort 2]), and subsequently 14 days of once-daily dosing (Part C, Cohort 3). In Parts A and C, open-label sitagliptin is included as a comparator to provide data that will aid the assessment of the therapeutic potential of GSK1292263. Part B is being conducted to provide an estimate, in T2DM, of the magnitude of the effect of food on the PK of GSK1292263 at the highest dose that will be used in the study. If necessary, this will allow refinement of the doses selected for Part C~~, where the subjects will be receiving the study drug approximately 30min before a meal tolerance test (MTT) at breakfast time~~. **(See the SPM for timings of meals in relation to dosing.)**

Section 1.5.2. Risks Related to GSK1292263

PREVIOUS TEXT

Glucose levels will be monitored by capillary blood glucose (CBG) at least twice daily (fasting and pre-evening meal) when the subject is in the unit and during washout of anti-diabetic medications prior to dosing a study drug.

REVISED TEXT

Glucose levels will be monitored by capillary blood glucose (CBG) at least twice daily (fasting and pre-evening meal) when the subject is in the unit and during washout of anti-diabetic medications prior to dosing a study drug. **In addition, at the blood draws after dosing in Parts A and B:**

A drop of blood will be used to provide real-time glucometer readings of capillary glucose levels (no additional blood required)

Subjects will be asked ‘how are you feeling’ to provide an assessment of neuroglycopenia

Subjects will have IV access post-dosing for rapid treatment of hypoglycemia, if required.

Section 4.1.1. Part A: Crossover Study Comparing a Low, Mid and High Dose of GSK1292263, Placebo and Sitagliptin (Cohort 1)

ADDED TEXT

For Part A, folate and iron supplementation may be started for 2 weeks after completion of Period 5 procedures, as necessary (see also Section 4.1.4).

Section 4.1.2. Part B: GSK1292263 with Food (Cohort 2)

PREVIOUS TEXT

Part B (Cohort 2) is a single-blind, randomized, placebo-controlled, 2-period study in which T2DM subjects will receive a single dose of GSK1292263, fasted or fed. T2DM subjects on monotherapy or sub-maximal anti-diabetic medications will wash-off these medications for 1 week prior to study receiving study drug.

In the FTIH study, GPR111596, food increased the exposure of GSK1292263 by up 2-to 3-fold in normal volunteers. Part B will comprise a two-period crossover of a single tablet dose of GSK1292263 administered in the fed or fasted state. The planned dose in Part B will be 800mg and will be administered 30min after eating a high fat breakfast meal. This dose may be changed based on emergent safety, tolerability and PD data. Results from Part B will be used to confirm the 'food effect' seen in study GPR111596, and will allow assessment of the extent of the change in PK exposure in the presence of food in T2DM patients. This will aid in the selection of doses in Part C, where the subjects will eat a standardised meal tolerance test (MTT) at breakfast within 30min of dosing with study drug. Part B will be conducted in parallel with the last two periods of Part A.

REVISED TEXT

Part B (Cohort 2) is a single-blind, randomized, ~~placebo-controlled,~~ 2-period study in which T2DM subjects will receive a single dose of GSK1292263, fasted or fed. T2DM subjects on monotherapy or sub-maximal anti-diabetic medications will wash-off these medications for 1 week prior to study receiving study drug. **This will commence after dosing in Part A is completed.**

In the FTIH study, GPR111596, food increased the exposure of GSK1292263 by up 2-to 3-fold in normal volunteers. Part B will comprise a two-period crossover of a single tablet dose of GSK1292263 administered in the fed or fasted state. The planned dose in Part B will be 800mg and will be administered ~~30min~~ after eating a high fat breakfast meal **(see the SPM for specifics)**. This dose may be changed based on emergent safety, tolerability and PD data. Results from Part B will be used to confirm the 'food effect' seen in study GPR111596, and will allow assessment of the extent of the change in PK exposure in the presence of food in T2DM patients. This will aid in the selection of doses in Part C, where the subjects will eat a standardised meal tolerance test (MTT) at breakfast ~~within 30min of dosing with study drug~~. Part B will be conducted in parallel with the last two periods of Part A.

Section 4.1.4. Screening

ADDED TEXT

For Part A, subjects who are eligible for enrollment in the study after the Screening procedure will be counselled on dietary methods and may be prescribed folate and iron, if appropriate, from the Screening visit to 7 days before randomization to minimize the effects of blood draws on haemoglobin levels.

Section 4.1.8.2. Meal Tolerance test (MTT) – Part C only

PREVIOUS TEXT

On Days -1, 7 and 14 in Part C, subjects will be administered a standardized MTT 30min after administration of the study drug. Blood samples will be collected up to 180min so that the first and second phases of insulin secretion can be derived by modelling the C-peptide and insulin kinetics data during the MTT. In addition, insulin sensitivity may be calculated from the rate of appearance and disappearance of the ingested glucose in the meal.

The composition of the meal for the MTT is defined in the SPM.

REVISED TEXT

On Days -1, 7 and 14 in Part C, subjects will be administered a standardized MTT ~~30min after administration of the study drug~~ **at breakfast time**. Blood samples will be collected up to 180min so that the first and second phases of insulin secretion can be derived by modelling the C-peptide and insulin kinetics data during the MTT. In addition, insulin sensitivity may be calculated from the rate of appearance and disappearance of the ingested glucose in the meal.

The composition of the meal for the MTT **and timing relative to dosing are** ~~is~~ defined in the SPM.

Section 4.1.8.3. Hunger, satiety and caloric intake (Part C) [header modified]

Section 4.4. Dose Adjustment/Stopping Criteria

PREVIOUS TEXT

For Part B, a planned dose of 800mg GSK1292263 may be adjusted based on emergent safety and tolerability data from the first 3 periods of Part A (Part B will run in parallel with Periods 4 and 5 of Part A).

REVISED TEXT

For Part B, a planned dose of 800mg GSK1292263 may be adjusted based on emergent safety and tolerability data from ~~the first 3 periods of~~ Part A ~~(Part B will run in parallel with Periods 4 and 5 of Part A)~~.

Section 4.4.3. Blood Glucose Withdrawal Criteria

PREVIOUS TEXT

- If fasting plasma glucose >270mg/dL, confirmed by prompt repeat testing.

REVISED TEXT

- If fasting plasma glucose >2**8**0mg/dL, confirmed by prompt repeat testing.

Section 4.4.4. Additional Withdrawal Criteria

ADDED TEXT

- **In Parts A and B, subjects who present pre-dose (Day -1) with a hemoglobin < 10 g/dL will be withdrawn from the study.**

Section 5.2.1. Inclusion Criteria

PREVIOUS TEXT

4. BMI (body mass index) within the range 25-35 kg/m2, inclusive.
5. T2DM diagnosed by American Diabetes Association criteria at least 3 months prior to Screening with:
   - Fasting plasma glucose (FPG) level ≤ 220mg/dL at the Screening visit
   - FPG level ≤ 250mg/dL on Day -1
   - HbA1c between 7 and 11%, inclusive, at Screening visit
6. For Part A, T2DM currently controlled by diet and exercise.
7. For Parts B and C, T2DM currently controlled by diet and exercise, or, if on medication, subjects must be treating their T2DM using one of the following regimens:
   - Metformin as monotherapy
   - Sulfonylurea as monotherapy
   - Metformin and sulfonylurea in combination, if both components are being administered at doses that are half their maximum dose or less
   - DPP-IV inhibitors, either as monotherapy or in combination with other agent(s) on this list at half maximal dose or less
   - Exenatide, either as monotherapy or in combination with other agent(s) on this list at half maximal dose or less

   For subjects that are being screened for Parts B and C, all doses of anti-diabetic medication must have been stable for at least 3 months prior to Screening, and the subject must be willing to wash out from their anti-diabetic medications from Day -7 through post-last-dose of Period 2 (Part B) or Day -7 through Day 15 (Part C).
8. Capable of giving written informed consent, which includes compliance with the requirements and restrictions listed in the consent form.

REVISED TEXT

4. BMI (body mass index) within the range 2**2**-35 kg/m^2, inclusive.
5. Part A: T2DM diagnosed by American Diabetes Association criteria prior to Screening:
   - **Currently controlled by diet and exercise, and no anti-hyperglycemic medications used in the past 3 months.**
   - Fasting plasma glucose (FPG) level ≤ 250mg/dL at the Screening visit.
   - FPG level ≤ 250mg/dL on Day -1.
   - HbA1c between 6.5 and 11%, inclusive, at Screening visit.
6. For Parts B and C: T2DM diagnosed by American Diabetes Association criteria for at least 3 month prior to Screening:
   - Controlled by diet and exercise, or, if on medication, subjects must be treating their T2DM using one of the following regimens:
     - ❖ Metformin as monotherapy
     - ❖ Sulfonylurea as monotherapy
     - ❖ Metformin and sulfonylurea in combination, if both components are being administered at doses that are half their maximum dose or less
     - ❖ DPP-IV inhibitors, either as monotherapy or in combination with other agent(s) on this list at half maximal dose or less
     - ❖ Exenatide, either as monotherapy or in combination with other agent(s) on this list at half maximal dose or less

   For subjects that are being screened for Parts B and C, all doses of anti-diabetic medication must have been stable for at least 3 months prior to Screening, and the subject must be willing to wash out from their anti-diabetic medications from Day -7 through post-last-dose of Period 2 (Part B) or Day -7 through Day 15 (Part C).

   - Fasting plasma glucose (FPG) level ≤ 220mg/dL at the Screening visit
   - FPG level ≤ 250mg/dL on Day -1
   - HbA1c between 7 and 11%, inclusive, at Screening visit
7. Capable of giving written informed consent, which includes compliance with the requirements and restrictions listed in the consent form.

Section 5.2.2. Exclusion Criteria

PREVIOUS TEXT

- Hemoglobin < 11g/dL (A subject with hemoglobin < 11 g/dL, but > 10 g/dL, may be enrolled only if the Investigator and the GSK Medical Monitor agree that the finding is unlikely to introduce additional risk to the subject and will not interfere with the study procedures).

REVISED TEXT

- **For females a haemoglobin < 11.5 g/dL, and for males a hemoglobin < 12.5 g/dL.** ~~Hemoglobin < 11g/dL~~**(A female subject with haemoglobin between 10g/dL and 11.5 g/dL, or a male subject with haemoglobin between 10g/dL and 12.5 g/dL** ~~(A subject with hemoglobin < 11 g/dL, but > 10 g/dL,~~ may be enrolled only if the Investigator and the GSK Medical Monitor agree that the finding is unlikely to introduce additional risk to the subject and will not interfere with the study procedures).

Section 7.2.4. Clinical Laboratory Assessments

ADDED TEXT

Glycerol (Part C only)

Section 8 Lifestyle and/or Dietary Restrictions

PREVIOUS TEXT

Fasting	Subjects should fast from all food or drink with the exception of water from midnight prior to each blood collection for clinical chemistry tests.<br>Subjects should fast from all food or drink with the exception of water from midnight prior to each morning administration of study drug, except for Part B when the study drug will be administered within 30min of consuming a high fat breakfast.
Water	Water is permitted until one hour prior to study drug administration when subjects are dosed in the unit. In Parts A and C, subjects may consume water *ad libitum* after dosing, provided it does not adversely affect the OGTT in Part A. In Part B, subjects may consume water *ad libitum* beginning one hour after dosing.
Breakfast	Breakfast will be omitted on days when the OGTT will be administered (Part A). In Part B, subjects will be fed breakfast 30 minutes prior to dosing or they will be dosed in a fasted state and will not eat until lunchtime. On Days -1 through 14 in Part C, a standardized breakfast will be consumed within 30 min of dosing. On Days -1, 7 and 14 the breakfast meal will be referred to as a meal tolerance test (MTT). See SPM for menu details.

REVISED TEXT

Fasting	Subjects should fast from all food or drink with the exception of water from midnight prior to each blood collection for clinical chemistry tests.<br>Subjects should fast from all food or drink with the exception of water from midnight prior to each morning administration of study drug~~, except for Part B when the study drug will be administered within 30min of consuming a high fat breakfast~~ See the SPM for timing of meals in relation to dosing for each part of the study.
Water	Water is permitted until one hour prior to study drug administration when subjects are dosed in the unit. In Parts A and C, subjects may consume water *ad libitum* after dosing, provided it does not adversely affect the OGTT in Part A. In Part B, subjects may consume water *ad libitum* beginning one hour after dosing.
Breakfast	Breakfast will be omitted on days when the OGTT will be administered (Part A). ~~In Part B, subjects will be fed breakfast 30 minutes prior to dosing or they will be dosed in a fasted state and will not eat until lunchtime. On Days -1 through 14 in Part C, a standardized breakfast will be consumed within 30 min of dosing.~~ On Days -1, 7 and 14 the breakfast meal will be referred to as a meal tolerance test (MTT). See SPM for menu details.
