# Supplementary material for: Gut Hormone Pharmacology of a Novel GPR119 Agonist (GSK1292263), Metformin, and Sitagliptin in Type 2 Diabetes Mellitus: Results from Two Randomized Studies
Source: PLoS One. 2014 Apr 3;9(4):e92494. doi: 10.1371/journal.pone.0092494 (PMC3974707; doi:10.1371/journal.pone.0092494)
Supplement: Protocol S2 — Trial Protocol 2. (PDF) [file pone.0092494.s003.pdf]

Division: Worldwide Development
Retention Category: GRS019
Information Type: Protocol Amendment

**Title:**	A study in type 2 diabetic subjects on stable metformin therapy to investigate the safety, tolerability, pharmacokinetics and pharmacodynamics of co-administering single and multiple oral doses of GSK1292263

Compound Number: GSK1292263

Effective Date: 08-FEB-2010

Protocol Amendment Number: 02

Description: This amendment adds (i) an extra arm in Part B to evaluate the safety, tolerability, PK and PD of 600mg QD when co-administered with metformin, and (ii) apolipoprotein and CRP measurements to the fasting laboratory analyses. The addition of the QD arm will allow comparison of the PK and PD of 600mg QD GSK1292263 when administered as monotherapy and as an add-on to metformin.

Subject: safety, tolerability, pharmacokinetics, glucose, pharmacodynamics, male, female, type II diabetes, metformin

Author:

[redacted] EE Discovery Medicine, Metabolic Pathways CEDD
CPSSO, Metabolic Pathways
CPMS, Clinical Pharmacology Modeling and Simulation
Discovery Biometrics - Metabolic

Copyright 2010 the GlaxoSmithKline group of companies. All rights reserved. Unauthorised copying or use of this information is prohibited.

Revision Chronology:

RM2009/00552/00	2009-NOV-09	Original
RM2009/00552/01	2010-JAN-15	Amendment No. 01: This amendment includes (i) preliminary safety, tolerability and PK information from Part C of study GPR111598, (ii) preliminary data from 13-week toxicology studies in the rat and dog, (iv) sets the maximum daily dose to be evaluated in Part B at 600mg, and (v) allows up to 8 subjects to be enrolled in Part A if required to define the PK of single dose GSK1292263 when co-administered with metformin.
RM2009/00552/02	2010-FEB-08	Amendment No. 02: This amendment adds (i) an extra arm in Part B to evaluate the safety, tolerability, PK and PD of 600mg QD when co-administered with metformin, and (ii) apolipoprotein and CPR measurements to the fasting laboratory analyses.

CONFIDENTIAL RM2009/00552/02
GPR113132

SPONSOR SIGNATORY:

[redacted]

8 February 2010
Date

[redacted] MD, FRCP
Director and Head
EnteroEndocrine Discovery Medicine
Metabolic Pathways CEDD

SPONSOR/MEDICAL MONITOR INFORMATION PAGE

Medical Monitor and Sponsor Contact Information:

Role	Name	Day Time Phone Number & email	After-hours Phone/Cell/ Pager Number	Fax Number	GSK Address
Primary Medical Monitor	[redacted]	[redacted]	[redacted]	[redacted]	[redacted]
Secondary Medical Monitor	[redacted]	[redacted]	[redacted]	[redacted]	[redacted]
Tertiary Medical Monitor	[redacted]	[redacted]	[redacted]	[redacted]	[redacted]

Sponsor Registered Address:

GlaxoSmithKline Research & Development Limited
980 Great West Road
Brentford
Middlesex, TW8 9GS
UK

In some countries, the clinical trial sponsor may be the local GlaxoSmithKline affiliate company (or designee). If applicable, the details of the alternative Sponsor and contact person in the territory will be provided to the relevant regulatory authority as part of the clinical trial application.

Regulatory Agency Identifying Number(s): IND 103,221

INVESTIGATOR PROTOCOL AGREEMENT PAGE

I confirm agreement to conduct the study in compliance with the protocol, as amended by this protocol amendment.

Investigator Name:	
Investigator Address:	
Investigator Phone Number:	
Investigator Signature	Date

TABLE OF CONTENTS

Page

ABBREVIATIONS 10

1. INTRODUCTION 14
   1.1. Background 14
      1.1.1. Type 2 Diabetes Mellitus Pathophysiology and Treatment . . . 14
      1.1.2. GSK1292263 14
   1.2. Previous Human Experience with GSK1292263 18
      1.2.1. First Time in Human (Healthy Subjects) 18
      1.2.2. First Time in T2DM subjects (Single and Multiple Dose) 19
   1.3. Metformin 27
      1.3.1. Metformin Pharmacology 27
      1.3.2. Absorption, Metabolism and Elimination of Metformin 28
   1.4. Sitagliptin 28
   1.5. Rationale 29
      1.5.1. Study Rationale 29
      1.5.2. Dose Rationale 29
   1.6. Summary of Risk Management 32
      1.6.1. Risks Related to Washout of Anti-Diabetic Medications 32
      1.6.2. Risks Related to GSK1292263 32
      1.6.3. Risks related to GSK12922263 and Metformin Co-Administration 35
      1.6.4. Risks Related to Sitagliptin and Sitagliptin-Metformin Co-administration 36
      1.6.5. Capillary Blood Glucose (CBG) Monitoring 37

2. OBJECTIVE(S) 38
   2.1. Primary 38
   2.2. Exploratory 38

3. ENDPOINT(S) 38
   3.1. Primary 38
   3.2. Exploratory 39

4. INVESTIGATIONAL PLAN 40
   4.1. Study Design/Schematic 40
   4.2. Discussion of Design 40
      4.2.1. Part A: Assessment of Single Dose PK of GSK1292263 in T2DM Subjects Taking Metformin 40

4.2.2. Part B: Repeat Dosing of GSK1292263 to T2DM Subjects Taking Metformin 41
4.2.3. Screening 43
4.2.4. Washout 43
4.2.5. Treatment regimen 43
4.2.6. Follow-up Visit 44
4.2.7. Pharmacodynamic Testing 44
4.3. Treatment Assignment 46
4.4. Investigational Product Dosage/Administration 47
4.5. Dose Adjustment Criteria 47
4.6. Safety Criteria 49
4.6.1. Liver Chemistry Stopping Criteria 49
4.6.2. QTc Withdrawal Criteria 49
4.6.3. Additional Withdrawal Criteria 50
4.6.4. Blood Glucose Withdrawal Criteria 50
4.7. Time and Events Table (Part A) 51
4.8. Time and Events Table (Part B) 53

5. STUDY POPULATION 56
5.1. Number of Subjects 56
5.2. Eligibility Criteria 56
5.2.1. Inclusion Criteria 56
5.2.2. Exclusion Criteria 57
5.2.3. Other Eligibility Criteria Considerations 60

6. DATA ANALYSIS AND STATISTICAL CONSIDERATIONS 61
6.1. Hypotheses and Treatment Comparisons 61
6.2. Sample Size Considerations 62
6.2.1. Sample Size Assumptions 62
6.2.2. Sample Size Sensitivity 62
6.2.3. Sample Size Re-estimation 63
6.3. Data Analysis Considerations 63
6.3.1. Interim Analysis 63
6.3.2. Final Analyses 63

7. STUDY ASSESSMENTS AND PROCEDURES 66
7.1. Demographic/Medical History Assessments 66
7.2. Safety 66
7.2.1. Physical Exams 66
7.2.2. Vital Signs 67

7.2.3. Electrocardiogram (ECG) 67
7.2.4. Clinical Laboratory Assessments 67
7.3. Pregnancy 68
7.3.1. Time period for collecting pregnancy information 68
7.3.2. Action to be taken if pregnancy occurs 68
7.3.3. Action to be taken if pregnancy occurs in a female partner of a male study subject 69
7.4. Pharmacokinetics 69
7.4.1. Blood Sample Collection 69
7.4.2. Sample Analysis 69
7.5. Biomarker(s)/Pharmacodynamic Markers 69
7.5.1. Type II Diabetes Biomarkers/Pharmacodynamic Markers 69
7.5.2. Exploratory Biomarkers 70
7.6. Pharmacogenetics 70

8. LIFESTYLE AND/OR DIETARY RESTRICTIONS 70
8.1. Contraception Requirements 70
8.1.1. Male Subjects 70
8.2. Meals and Dietary Restrictions 71

9. CONCOMITANT MEDICATIONS AND NON-DRUG THERAPIES 72
9.1. Permitted Medications 72
9.1.1. Anti-Viral Therapies for Influenza 72
9.2. Medications Permitted at Screening but Requiring Washout before Enrollment 72
9.3. Prohibited Medications 73
9.4. Non-Drug Therapies 73

10. COMPLETION OR EARLY WITHDRAWAL OF SUBJECTS 74
10.1. Subject Completion 74
10.2. Subject Withdrawal Criteria 74
10.3. Subject Withdrawal Procedures 74
10.3.1. Subject Withdrawal from Study 74
10.3.2. Subject Withdrawal from Investigational Product 74
10.4. Treatment After the End of the Study 74
10.5. Screen and Baseline Failures 75

11. INVESTIGATIONAL PRODUCT(S) 75
11.1. Blinding 75
11.2. Packaging and Labeling 75
11.3. Preparation/Handling/Storage/Accountability 76

11.4. Assessment of Compliance . . . 76
11.5. Treatment of Investigational Product Overdose . . . 76
12. ADVERSE EVENTS (AE) AND SERIOUS ADVERSE EVENTS (SAE) . . 77
12.1. Definition of Adverse Events . . . 77
12.2. Definition of Serious Adverse Events . . . 78
12.3. Laboratory and Other Safety Assessment Abnormalities Reported as AEs and SAEs . . . 80
12.4. Disease-Related Events and/or Disease-Related Outcomes Not Qualifying as SAEs . . . 80
12.4.1. Pregnancy . . . 80
12.5. Method of Detecting AEs and SAEs . . . 80
12.6. Recording of AEs and SAEs . . . 81
12.7. Evaluating AEs and SAEs . . . 81
12.7.1. Assessment of Intensity . . . 81
12.7.2. Assessment of Causality . . . 81
12.8. Follow-up of AEs and SAEs . . . 82
12.9. Prompt Reporting of SAEs and Other Events to to GSK . . . 82
12.10. Regulatory Reporting Requirements for SAEs . . . 85
13. LIVER CHEMISTRY FOLLOW-UP PROCEDURES . . . 85
14. STUDY CONDUCT CONSIDERATIONS . . . 87
14.1. Posting of Information on clinicaltrials.gov . . . 87
14.2. Regulatory and Ethical Considerations, Including the Informed Consent Process . . . 87
14.3. Quality Control (Study Monitoring) . . . 88
14.4. Quality Assurance . . . 88
14.5. Study and Site Closure . . . 88
14.6. Records Retention . . . 89
14.7. Provision of Study Results to Investigators, Posting to the Clincal Trials Register and Publication . . . 89
14.8. Data Management . . . 90
15. REFERENCES . . . 91
Appendix 1: Liver Safety Algorithms . . . 92
Appendix 2: Pharmacogenetic research . . . 93
Appendix 3: Hunger, Craving, and Fullness Questionnaire . . . 97
Appendix 4: Protocol Amendment Changes . . . 100

ABBREVIATIONS

μM	Micromolar
ADME	Absorption, Distribution, Metabolism and Excretion
AE	Adverse Event
ALT	Alanine aminotransferase (SGPT)
ANCOVA	Analysis of Co-variance
ANOVA	Analysis of Variance
Apo	Apolipoprotein
AST	Aspartate aminotransferase (SGOT)
AUC	Area under concentration-time curve
AUC(0-∞)	Area under the concentration-time curve from time zero (pre-dose) extrapolated to infinite time
AUC(0-τ)	Area under the concentration-time curve over the dosing interval
AUC(0-t)	Area under the concentration-time curve from time zero (pre-dose) to last time of quantifiable concentration within a subject across all treatments
AUC(0-x)	Area under the concentration-time curve from zero (pre-dose) to some fixed nominal time x
BID	Twice daily
BMI	Body mass index
BP	Blood pressure
BPM	Beat Per Minute
BUN	Blood urea nitrogen
Cτ	Trough concentration
CBG	Capillary blood glucose
CCK	Cholecystokinin
CI	Confidence Interval
CL/F	Apparent clearance following oral dosing
Cmax	Maximum observed concentration
CO_2	Carbon dioxide
CPDS	Clinical Pharmacology Data Sciences
CPK	Creatine phosphokinase
CPKMS	Clinical Pharmacokinetics Modelling & Simulation
CRF	Case Report Form
CRP	C-Reactive Protein
CPSSO	Clinical Pharmacology Science and Study Operations
CV	Coefficient of variance
Cτ	Pre-dose (trough) concentration at the end of the dosing interval
D	Day
DILI	Drug Induced Liver Injury
dL	Deciliters
DMPK	Drug Metabolism and Pharmacokinetics
DNA	Deoxyribonucleic acid
DPP-IV	Dipeptidyl peptidase-IV
ECG	Electrocardiogram
eCRF	Electronic Case Report Form
FDA	Food and Drug Administration

FFA	Free fatty acids
FPG	Fasting plasma glucose
FSH	Follicle Stimulating Hormone
FTIH	First time in humans
FU	Follow-up
g	Grams
GCP	Good Clinical Practice
GCSP	Global Clinical Safety and Pharmacovigilence
GGT	Gamma glutamyltransferase
GI	Gastrointestinal
GIP	Glucose-dependent insulinotropic peptide
GLP	Good Laboratory Practice
GLDH	Glutamate dehydrogenase
GLP-1	Glucagon-like peptide-1
GSK	GlaxoSmithKline
h/hr	Hour(s)
HbA1c	Glycosylated hemoglobin
HBsAg	Hepatitis B surface antigen
HCFQ	Hunger, Craving and Fullness Questionaire
HDL	High-density lipoprotein
Hep B	Hepatitis B
Hep C	Hepatitis C
HIV	Human Immunodeficiency Virus
HR	Heart rate
HWE	Hardy-Weinberg Equilibrium
IB	Investigator's Brochure
IEC	Independent Ethics Committee
IgM	Immunoglobulin
IGT	Impaired glucose tolerance
IND	Investigational New Drug
IRB	Institutional Review Board
IWRS	Interactive Web-based Response System
Kg	Kilogram
L	Liter
LDH	Lactate dehydrogenase
LDL	Low-density lipoprotein
MCH	Mean corpuscular hemoglobin
MCHC	Mean corpuscular hemoglobin concentration
MCV	Mean corpuscular volume
MDRD	Modification of diet in renal disease
mg	Milligrams
min	Minutes
mL	Milliliter
mmHg	Millimeters of Mercury
ms	Milliseconds
MSDS	Material Safety Data Sheet
msec	Milliseconds
MTT	Meal Tolerance Test

ng.h/mL	Nanogram hours per milliliter
ng/mL	Nanograms per milliliter
NOAEL	No observed adverse event level
NSAID	Non-steroidal anti-inflammatory drug
OGTT	Oral glucose tolerance test
PD	Pharmacodynamic
PGx	Pharmacogenetics
PI	Primary Investigator
PIB	Powder in bottle
PK	Pharmacokinetic
PP	Pancreatic polypeptide
PRN	As needed
PYY	Peptide Tyrosine-Tyrosine
QD	Once daily
QTc	Corrected QT interval
QTcB	QT duration corrected for heart rate by Bazett's formula
QTcF	QT duration corrected for heart rate by Fridericia's formula
RAP	Reporting and Analysis Plan
RBC	Red blood cells
RNA	Ribonucleic acid
Ro	Accumulation ratio
Rs	Time invariance ration
SAE	Serious adverse event(s)
SAS	Statistical Analysis Software
SCr	Serum creatinine
SD	Standard deviation
SGOT	Serum glutamic oxaloacetic transaminase
SGPT	Serum gluatmic pyruvic transaminase
SNP	Single nucleotide polymorphism
SPM	Study Procedures Manual
SSRI	Selective serotonin receptor inhibitor
t½	Terminal phase half-life
T2DM	Type 2 diabetes mellitus
tlag	Lag time before observation of drug concentrations in sampled matrix
tmax	Time of occurrence of Cmax
TSH	Thyroid stimulating hormone
ULN	Upper limit of normal
USA	United States of America
V/F	Volume of distribution
WBC	White blood cells
WGS	Whole genome screen
WPW	Wolf-Parkinson-White Syndrome
X-over	Crossover

Trademark Information

Trademarks of the GlaxoSmithKline group of companies
RELENZA

Trademarks not owned by the GlaxoSmithKline group of companies
Byetta
Chiron RIBA
Glucophage
Janumet
Januvia
SAS
Tamiflu
WinNonlin

1. INTRODUCTION

GSK1292263 is a potent and selective agonist of GPR119, a receptor that represents a novel approach to target the enteroinsular axis and other target tissues to improve glucose homeostasis. This investigational agent is being developed as a potential treatment for Type 2 Diabetes Mellitus (T2DM), either as a monotherapy or in combination with other marketed oral anti-diabetic agents.

1.1. Background

1.1.1. Type 2 Diabetes Mellitus Pathophysiology and Treatment

T2DM is a metabolic disorder primarily characterized by insulin resistance and relative insulin deficiency. Insulin resistance alone is insufficient to produce T2DM, but when accompanied by progressive dysfunction of pancreatic islet ß-cells, the result is prandial and fasting hyperglycemia. Characteristically, there is worsening hyperglycemia that requires step-up therapy, often culminating in combination therapy using 2 or more oral antidiabetic agents with complementary mechanisms, and even progressing to the use of insulin when islet function is greatly compromised.

By altering nutrient-mediated insulin and glucagon secretion, the incretin hormones, glucagon like peptide-1 (GLP-1) and glucose-dependent insulinotropic peptide (GIP), are both major determinants of glucose disposal following a meal. In addition, gut-derived peptides, such as peptide YY (PYY), control food intake by affecting hunger and satiety. Interestingly, PYY and GLP-1 are secreted by the L cells present mainly in the colon, whilst GIP is secreted by K cells present in the small intestine.

In T2DM, plasma levels of GIP appear to be normal or possibly increased, while the ß-cell response to GIP is diminished. It has been shown that ß-cell responses to GIP increase with improved glycemic control. Conversely, although ß-cells remain responsive to the insulinotropic action of GLP-1, the meal-stimulated GLP-1 plasma increases are diminished in patients with impaired glucose tolerance and also in subjects with T2DM. Therefore, therapeutic strategies have focused on increasing GLP-1, either by administration of exogenous GLP-1 analogues, such as exenatide (Byetta) or by preserving endogenous GLP-1 with inhibitors of dipeptidyl peptidase-IV (DPP-IV), the protease responsible for the rapid degradation of incretins following their release from intestinal L-cells. Recently, activation of the G-coupled protein receptor, GPR119, has been found to increase incretin and gut hormone release, as well as insulin secretion.

1.1.2. GSK1292263

Below is a brief summary of the relevant non-clinical data obtained with GSK1292263 relevant to this protocol.

Further information is available in the Investigator Brochure and supplements [GlaxoSmithKline Document Number RM2008/00434/00; GlaxoSmithKline Document Number RM2009/00168/00; GlaxoSmithKline Document Number RM2009/00168/01].

1.1.2.1. Pharmacology

In preclinical studies, GSK1292263 was noted to have two principal mechanisms of action that produce lowering of blood glucose: (i) nutrient-independent and nutrient-augmented release of incretin (GLP-1 and GIP) and gastrointestinal (GI) hormones (e.g., PYY), (ii) increased glucose-stimulated insulin release.

The durability of GPR119 agonism has been demonstrated in a rodent model in which the glucose lowering effect of GSK1292263 during an OGTT was maintained for 10 weeks.

1.1.2.2. Metabolism and Elimination of GSK1292263

GSK1292263 is poorly soluble in water, and absorption decreases with increasing dose in the fasted state, probably due to the low solubility.

The oral bioavailability of micronized GSK1292263 generally ranged between ~20-30% in fed rats (10 and 30 mg/kg) and 7 to 15% in fasted dogs (3 and 28 mg/kg). A formal oral bioavailability study has not been completed in fed dogs; however a relative bioavailability study comparing fasted vs. fed dogs showed a 4-fold increase in the exposure for GSK1292263 with food. Plasma protein binding is ~99% in humans, rats and dogs, and binding was independent of GSK1292263 concentration (range 0.15 to 15 ug/mL).

The metabolism and elimination of GSK1292263 has not been fully characterized. Based on preliminary metabolism results from healthy volunteers demonstrating the presence of multiple metabolites in plasma and urine, along with the observation that there is little parent compound detected in urine, it is expected that GSK1292263 is extensively metabolized and the resulting metabolites are eliminated via biliary and urinary routes.

GSK1292263 has no direct inhibitory effect on CYP1A2, 2C9, 2C19, 2D6 and 3A4, and no metabolism-dependent inhibition of CYP3A4. It is a very weak activator of human PXR (<5% of maximal response), and thus is not expected to cause induction of human PXR target genes in vivo.

In vitro studies have demonstrated that in human liver microsomes, CYP3A4 is the main CYP enzyme involved in the oxidative metabolism of GSK1292263. In contrast, in human hepatocytes and in human plasma, there are a number of reductive and oxidative metabolites identified, suggesting that there are multiple routes of metabolism.

GSK1292263 is not a Pgp inhibitor or OATP1B3 inhibitor, but it has a modest inhibitory effect on OATP1B1 (43% at 1μM). Of relevance to this study, it is an inhibitor of OCT1 (69% at 30μM), but not of OCT2 (23% at 30μM) (see Section 1.3.2 and Section 1.6.3).

1.1.2.3. Non-clinical Toxicology

There were no test article-related adverse findings in rats or dogs administered GSK1292263 orally for 14 days at doses up to 2000 and 1000mg/kg/day, respectively. No other significant biological or adverse events were observed in these 14 day repeat dose safety studies.

Amendment No. 1 summarizes preliminary data from the 13-week toxicology studies in the dog and rat.

1.1.2.3.1. Preliminary Data from 13-week Toxicology Study in the Dog

In the 13-week dog study (4, 20, 1000mg/kg/day), preliminary histopathological evaluation revealed adverse GSK1292263-related changes in (i) the testes (minimal to slight tubular degeneration/depletion and Leydig cell hypertrophy) at ≥ 20mg/kg/day, and (ii) the liver (elevated ALT and GLDH in both sexes; minimal inflammation in males) at 1000mg/kg/day. There were no treatment-related findings in the liver or testes in the dog at 4mg/kg/day.

Testis:

Three of four males given 1000mg/kg/day and one male given 20mg/kg/day had minimal to mild degeneration/depletion of seminiferous epithelium in the testes. The change primarily affected spermatids in the superficial layers of the seminiferous epithelium indicating the test article effect was occurring in the later steps of the spermatogenic process while sparing the early spermatogonial stem cells. This suggests reversibility is likely to occur on cessation of treatment. However, no recovery groups were included in this study.

Liver:

Adverse GSK1292263-related changes were observed in the liver (increased ALT and GLDH in both sexes; minimal inflammation in males) at 1000mg/kg/day. No recovery groups were included on this study. Moderate to marked increases in ALT (3.3X to 11.3X in males, 4.3X to 11.7X in females based on individual animal values relative to pre-test) and marked increases in GLDH (4.4X to 22.3X in males, 5.8X to 15X in females based on individual animal values relative to pre-test) were observed at 1000mg/kg/day, with changes increasing in severity between Week 4 and Week 13. Although minimal inflammation with a mixed inflammatory cell population was present in males given 1000mg/kg/day, it was not present in females although females had higher enzyme elevations. There was no hepatocellular necrosis evident in any of the dogs given 1000mg/kg/day so there was no clear histopathology correlate explaining the ALT and GLDH elevations.

1.1.2.3.2. Preliminary Data from 13-week Toxicology Study in the Rat

In the 13-week rat study (10, 150, 2000mg/kg/day) there were no treatment-related findings in liver or testes up to 2000mg/kg/day.

1.1.2.4. Toxicokinetics

Based on the results of non-clinical toxicology studies [*GlaxoSmithKline Document Numbe RM2008/00434/00*], exposures in the current study were not to exceed a mean steady-state AUC(0-24h) of 49,400ng.h/mL and mean Cmax of 2693ng/mL which are 80% of the gender-averaged no-observed-adverse-effect-level (NOAEL) mean exposure in beagle dogs at Day 14 at the highest dose of 1000mg/kg/day.

Furthermore, no individual was to exceed an AUC(0-24h) of 72,700ng.h/mL or Cmax of 3585ng/mL, which are 80% of the highest AUC and Cmax observed in dog at 1000mg/kg NOAEL dose group in the 14 day toxicology study.

These exposures were consistent with limits that were approved for the conduct of the previous studies, GPR111956 and GPR111598, by the GSK Global Safety Board and the local IRBs.

1.1.2.4.1. Toxicokinetic Parameters from the 13-Week Toxicology Studies in Rat and Dog

The GSK1292263 concentrations in these studies are shown in Table 1.

Table 1 Mean Systemic Exposures Following Oral Administration of GSK1292263 in the Rat and Dog 13-week studies

Duration	Dose (mg/kg/day)	Sex	C_{max} (ng/mL)		AUC_{0-24} (ng.h/mL)	
			1st TK	End of Study[1]	1st TK	End of Study[1]
Rat (13 week)	10	M<br>F	488<br>712	395<br>995	4429<br>7002	3702<br>10868
	150	M<br>F	1696<br>2390	1081<br>2991	22016<br>33178	16285<br>42869
	2000	M<br>F	3394<br>(2899-3684)<br>6779<br>(5818-7372)	2543<br>(2332-2755)<br>5749<br>(4977-6521)	55601<br>(49322-63416)<br>92517<br>(83695-103185)	43551<br>(30628-56474)<br>89976<br>(82895-97057)
Dog (13 week)	4	M<br>F	870<br>702	1087<br>969	14034<br>9541	17517<br>12860
	20	M<br>F	2100<br>(1824-2467)<br>1716<br>(1402-1956)	2573<br>(1885-3145)<br>2377<br>(2237-2478)	32536<br>(28785-38282)<br>23153<br>(20103-25929)	43504<br>(27838-59195)<br>36930<br>(32808-39672)
	1000	M<br>F	5091<br>(2801-6846)<br>5323<br>(4857-5816)	5453<br>(2757-7304)<br>5743<br>(4628-6900)	86890<br>(38952-124997)<br>94643<br>(81316-108038)	107751<br>(48863-144523)<br>106938<br>(89998-124774)

1. First TK was Day 14 in rat and Day 1 in dog. End of study was Day 87 for rat and Day 90 for dog.

The AUC(0-24h) and Cmax associated with the no effect threshold for testicular findings in the dog were 36,772ng.h/mL and 2,537ng/mL, respectively, following 13 weeks of dosing. The lowest individual AUC(0-24h) and Cmax for the dog testicular effect were 48,863ng.h/mL and 2,757 ng/mL, respectively, following 13 weeks of dosing.

Protocol Amendment 1 now limits the maximum total daily dose in this study to 600mg (300mg BID) based on the results of the 13 week toxicology study in the dog (Section 1.1.2.3.1). A dose of 300mg BID, or a dosing regimen resulting in equivalent exposure, given for up to 14 days does not pose a significant clinical risk from the perspective of either mean or individual exposures to date (see summary of human pharmacokinetics in Section 1.2.2.3).

It is important to note that there were no treatment-related effects in liver or testes in the rat and dog up to 2000mg/kg/day and 1000mg/kg/day GSK1292263, respectively, at mean systemic exposures of 58,405ng·hr/mL (male rats) and 49,505ng·hr/mL (male dogs) on Day 14 of the 14-day toxicology study. In addition, stage-dependent qualitative evaluation of spermatogenesis demonstrated normal progression up to the limit dose in both species indicating there were no subtle morphological changes in the testes following 14 days of dosing in the 14-day toxicology study. In the 13-week rat study (10, 150, 2000mg/kg/day) there were no treatment-related findings in liver or testes up to 43,551ng.hr/mL (mean male rat AUC0-24hr).

1.2. Previous Human Experience with GSK1292263

1.2.1. First Time in Human (Healthy Subjects)

Information from the FTIH study, GPR111596, is summarized in Supplement 1 to the Investigator Brochure [GlaxoSmithKline Document Number RM2009/00168/00]. Single doses of 10, 40, 50, 80, 200 and 400mg, and 2-day and 5-day repeat doses of 250mg were evaluated in healthy subjects.

Briefly,

- Single doses of GSK1292263 were generally well-tolerated over the dose range of 10mg to 400mg. There were no significant safety issues. The majority of AEs were mild in intensity; no events were severe in intensity. No deaths were reported. One subject was withdrawn because of an SAE labelled as “viral gastroenteritis” with associated AEs of syncope, hypotension, orthostatic hypotension, hyperhydrosis, cold sweat, diarrhea, nausea, vomiting, viral gastroenteritis and pyrexia (this subject had received a single dose of 400mg GSK1292263). These events were not attributed to study drug. The most common AEs reported in the subjects who received GSK1292263 were: hypoglycemia (7 events; see below for further information), headache (6 events), dizziness (5 events) and hyperhidrosis (2 events).
- Two- and 5-day repeat doses of 250mg GSK1292263 were generally well-tolerated. There were no significant safety issues. The majority of AEs were mild in intensity; no events were severe in intensity. No deaths were reported. One subject was withdrawn because of a series of AEs that were labelled as “gastroenteritis” (this subject had received 2 doses of 250mg GSK1292263). These events were not attributed to study drug. The most common AEs reported in the subjects who received GSK1292263 were: hypoglycemia (5 events, see below for further information) and headache (3 events).

- No clinically significant, study drug-related changes from baseline were noted in routine blood and urine chemistry panels, complete blood count, vital signs, ECGs and physical examinations during these parts of the study. In particular, a thorough review of QTc intervals during the study did not identify a clinically significant effect of GSK1292263.
- There was no clear relationship between dose and hypoglycemia AEs in the FTIH study (GPR111596) and some events occurred in the subjects who received placebo. It is important to note that there is an error in Table 9 of the IB Supplement 1 – there were 0 episodes of hypoglycemia in Part E (5 days dosing with 250mg GSK1292263), rather than 5 as shown in the table.

1.2.2. First Time in T2DM subjects (Single and Multiple Dose)

GSK1292263 is being investigated in one Phase II study, GPR111598 "A study in type 2 diabetic subjects of single and multiple doses of orally administered GSK1292263 to investigate the safety, tolerability, pharmacokinetics and pharmacodynamics of the compound".

This study is being conducted in 3 parts:

- Part A (completed): a 5-way crossover evaluating single doses of 25, 150 and 800mg of GSK1292263, placebo and open-label sitagliptin 100mg. This was conducted to compare the safety, tolerability and pharmacodynamics of GSK1292263 to sitagliptin after single doses. Subjects were administered a 75g oral glucose tolerance test 2 hours after dosing.
- Part B (completed): a 2-way crossover evaluating a single dose of 800mg GSK1292263 administered in the fed and fasting state. This was done to determine whether food would produce a similar increase in systemic exposure in T2DM to that seen in healthy volunteers.
- Part C (on-going): 14 days of repeat dose evaluating 50mg BID, 150mg BID and 300mg BID and 600mg QD of GSK1292263, placebo and open-label sitagliptin 100mg. On Day 14, subjects randomized to the GSK1292263 and placebo groups also receive sitagliptin 100mg. This part is designed to compare the safety, tolerability and pharmacodynamics of GSK1292263 to sitagliptin after multiple doses and to examine the effects of co-administering the two drugs.

It is important to note that the investigators are still blinded to the treatment allocation in study GPR111598, and only preliminary results without treatment allocation are presented below because some investigators may also be participating in the present study, GPR113132.

1.2.2.1. Part A

Safety

In Part A, 12 T2DM subjects received singles doses of 25mg and 150mg GSK1292263, and 11 received 800mg single doses of GSK1292263 (the twelfth subject was withdrawn

before completing the 800mg period; see below). These doses of GSK1292263 were safe and well tolerated.

Points of note:

- There was no association of study drug to significant AEs (see Table 2).
- There was 1 AE of asymptomatic hypoglycemia several hours after lunch on glucometer testing (48mg/dL) that was not observed on repeat laboratory testing.
- There were episodes of blurred vision and dizziness that were not associated with hypoglycemia and in some cases related to rapid glucose changes during the OGTT.
- There were no clinically significant changes in vital signs.
- There were no clinically significant ECG or telemetry changes, including QTc.
- There were no clinically significant safety lab changes, other than increased blood glucose levels typical of T2DM subjects and one instance of elevated Mg^{2+} that was considered to be a laboratory error.

One subject was withdrawn from the study in Period 1 because of severe hyperglycemia during Day 1 after receiving sitagliptin 100mg. On further questioning it was discerned that this subject was not drug naïve, as required for enrolment into the study, and had taken oral anti-diabetic medications and insulin in the past. This subject was replaced.

Another subject was withdrawn on D-1 prior to dosing in Period 4 because of a positive cotinine test and high fasting blood glucose. The subject had completed Periods 1-3. This subject was not replaced.

Table 2 Most Frequent Adverse Events For GPR111598 (Part A)

	Single Dose		
Most Frequent Adverse Events	Placebo N=11	Sitagliptin N=12	GSK1292263 N=12
	n (%)		
Any AE	2 (18%)	2 (17%)	2 (17%)
Any AE related to investigational product	1 (9%)	0	1 (9%)
Most Common AEs:			
Headache	2 (18%)	0	2 (17%)
Blurry Vision	0	1 (9%)	2 (17%)
Hyperglycemia	0	1 (9%)	0

Pharmacokinetics

Table 3 and Table 4 show the preliminary AUC and Cmax PK data for GSK1292263 after singles doses of 25, 150 and 800mg administered in the fasted state 2h before an OGTT. Note that complete PK profiles are available from 12 subjects who received 25mg and 150mg and from 11 subjects who received 800mg.

Table 3 Preliminary AUC(0-24) for GSK1292263 (Fasted) in Part A of study GPR111598

Dose (mg)	AUC(0-24h), ng.h/mL (CV%)	Exposure Ratio Relative to Mean Exposure at NOAEL for 14-day Toxicity Study [1]	Exposure Ratio Relative to Individual Exposure at NOAEL for 14-day Toxicity Study [2]
25	537 (20)	92	101
150	1748 (32)	28	24
800	4109 (25)	12	13

1. Mean AUC(0-24h) limit = 49400 ng.h/mL
2. Individual AUC(0-24h) limit = 72700 ng.h/mL – Based on maximum individual exposure

Table 4 Preliminary Cmax for GSK1292263 (Fasted) in Part A of study GPR111598

Dose (mg)	Cmax, ng/mL (CV%)	Exposure Ratio Relative to mean Exposure at NOAEL for 14-day Toxicity Study [1]	Exposure Ratio Relative to Individual Exposure at NOAEL for 14-day Toxicity Study [2]
25	53 (17)	51	50
150	175 (32)	15	13
800	393 (27)	7	6

1. Mean Cmax limit = 2693 ng/mL
2. Individual Cmax limit = 3585 ng/mL – Based on maximum individual exposure

Pharmacodynamics

Figure 1 shows the incremental AUC(0-3h) for glucose and insulin during the 75g OGTT. There was a trend in the reduction of glucose incremental AUC that was related to an increase in dose of GSK1292263. At the highest dose of 800mg, the reduction in glucose incremental AUC was equivalent to that seen with sitagliptin 100mg. The high variability in the insulin responses to GSK1292263 compromises the interpretation of these data.

Figure 1 Incremental AUC(0-3h) for Glucose and Insulin During OGTT

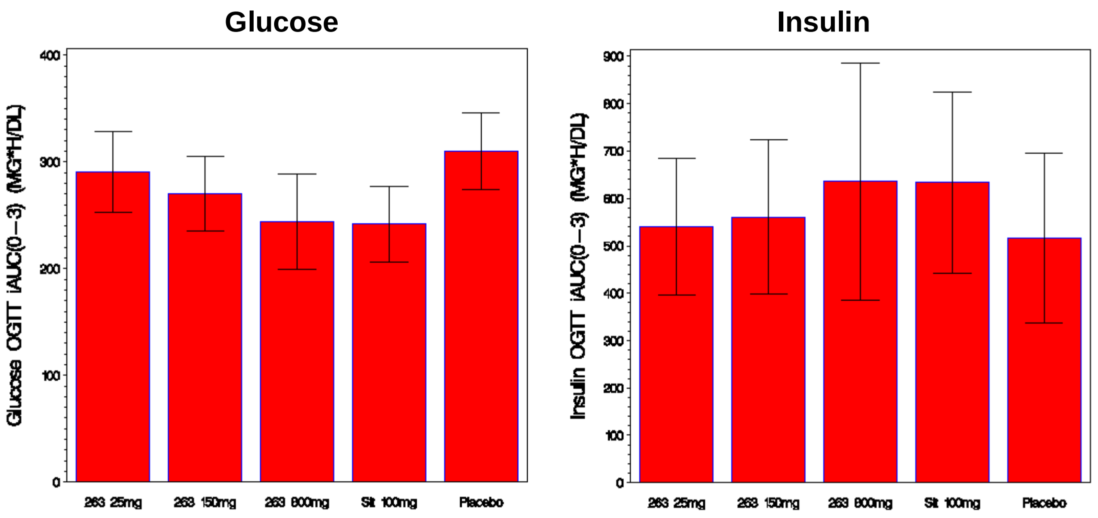

Error bars are the 95% confidence intervals

For GSK1292263, this reduction in glucose was accompanied by an elevation of total GLP-1, total GIP and total PYY relative to placebo, with no detectable change in active GLP-1. Sitagliptin, on the other hand, increased active GLP-1, but reduced levels of total GLP-1, GIP and PYY.

1.2.2.2. Part B

The four T2DM subjects in Part B received 800mg in the fasted and fed state. Three subjects were drug naïve, and the fourth was washed off metformin.

Safety

Points of note:

- There was no association of study drug to significant AEs.
- There were no clinically significant changes in vital signs.
- There were no clinically significant ECG or telemetry changes, including QTc, except for 1 short episode of Wenckebach AV block that occurred at ~3am and resulted in 1 dropped QRS complex.
- There were no clinically significant safety lab changes, other than increased blood glucose levels typical of T2DM.

Pharmacokinetics

Table 5 and Table 6 summarize the preliminary AUC and Cmax PK data for GSK1292263 after single doses of 800 mg in fasted and fed states. Note that complete PK profiles are available from all 4 subjects in this period. Across subjects,

GSK1292263 AUC(0-24h) increased by 2.6- to 4.5-fold in the fed state and Cmax increased 1.3- to 4.7-fold in the fed state. Mean AUC(0-24h) and Cmax increased 3.8- and 3.1-fold, respectively, in the fed state.

Table 5 Preliminary AUC(0-24) Following Single Doses of 800mg GSK1292263 (Fed and Fasted) in Part B of study GPR111598

Dose (mg)	Meal	AUC(0-24h), ng.h/mL (CV%)	Exposure Ratio Relative to Mean Exposure at NOAEL for 14-day Toxicity Study [1]	Exposure Ratio Relative to Individual Exposure at NOAEL for 14-day Toxicity Study [2]
800	Fasted	3429 (22)	14	17
800	Fed	12997 (29)	4	4

1. Mean AUC(0-24h) limit = 49400 ng.h/mL
2. Individual AUC(0-24h) limit = 72700 ng.h/mL – Based on maximum individual exposure

Table 6 Preliminary Cmax Following Single Doses of 800mg GSK1292263 (Fed and Fasted) in Part B of study GPR111598

Dose (mg)	Meal	Cmax, ng/mL (CV%)	Exposure Ratio Relative to Mean Exposure at NOAEL for 14-day Toxicity Study [1]	Exposure Ratio Relative to Individual Exposure at NOAEL for 14-day Toxicity Study [2]
800	Fasted	351 (27)	8	8
800	Fed	1009 (37)	3	3

1. Mean Cmax limit = 2693 ng/mL
2. Individual AUC(0-24h) limit = 3585 ng/mL – Based on maximum individual exposure

Individual trends for AUC(0-24h) and Cmax in the fasted and fed states are presented in the Figure 2:

Figure 2 Individual Trends in AUC(0-24h) and Cmax (Fed and Fasted)

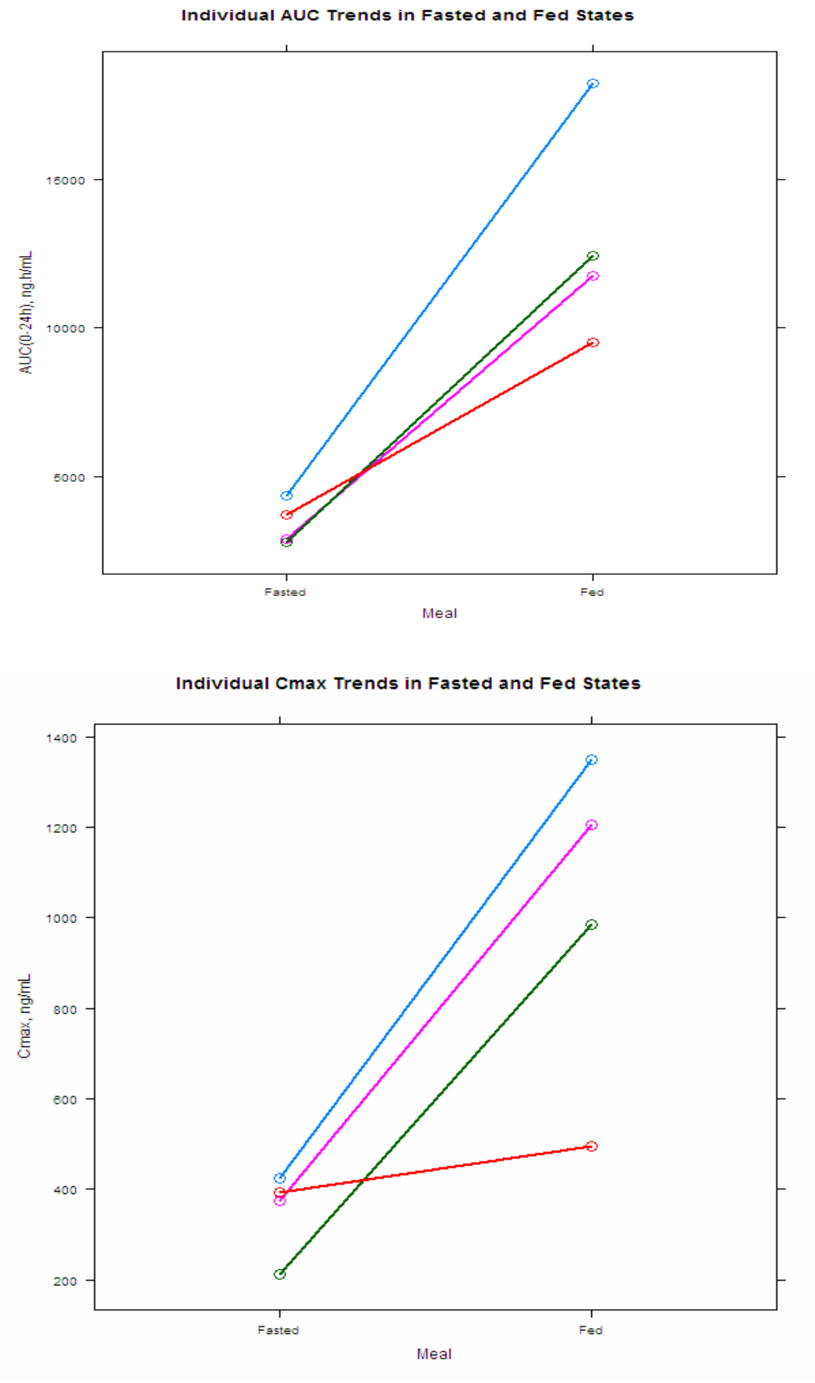

Red lines: subject washed off metformin for 7 days.

In Figure 2, the subject with the lowest AUC and Cmax in the fed state had been washed off metformin. As discussed in Section 1.3.1, metformin reduces bile acid reuptake and may reduce the bile acid pool released during meals. Given the low solubility in water of GSK1292263, bile acids may promote solubilisation and absorption of the drug. Therefore, it is possible that reduced levels of bile acids in the gut may explain the lower exposure seen in the metformin washout subject.

1.2.2.3. Part C (Ongoing)

Seventy four subjects have been enrolled in Part C as of February 4, 2010.

Safety

Points of note:

- Ten subjects have been withdrawn from the study.
    - [redacted]
    - [redacted]
    - [redacted]
    - [redacted]
    - [redacted]
    - [redacted]
    - [redacted]
    - [redacted]
    - [redacted]
    - [redacted]
- One additional subject was randomized but not dosed because the blood pressure and QTc parameters were outside of the protocol permitted limits.
- There have been no clinically significant AEs or changes in vital signs other than those indicated above.
- There have been no clinically significant ECG changes, including QTc.
- There have been no clinically significant safety lab changes, other than increased blood glucose levels typical of T2DM.

Pharmacokinetics

The maximum GSK1292263 exposures observed in the GPR111598 study have been associated with steady-state during the on-going Part C. The final doses of GSK1292263 selected in Part C were 50mg BID, 150mg BID, 300mg BID and 600mg QD. A

summary of preliminary exposures of GSK1292263 (n=6) at steady-state (Day 13 and Day 14) are presented in Table 7 (n=5-7 in each group).

Table 7 Summary of Preliminary GSK1292263 Exposure at Steady-State in Part C of study GPR111598

Dose	Day 13		Day 14	
	AUC(0-24h) (ng.h/mL) Mean ±SD [min, max]	Cmax (ng/mL) Mean ±SD [min,max]	AUC(0-24h) (ng.h/mL) Mean ±SD [min, max]	Cmax (ng/mL) Mean ±SD [min, max]
50mg BID	7944 ± 1362 [5357, 9203]	429 ± 58 [396, 536]	8153 ± 1240 [5758, 9331]	452 ± 53 [369, 515]
150mg BID	16986 ± 5863 [12449, 27137]	985 ± 312 [691, 1517]	17257 ± 5656 [12789, 26895]	952 ± 304 [717, 1479]
300mg BID	23101 ± 7900 [15606, 35959]	1258 ± 376 [912, 1885]	23810 ± 8517 [15585, 38771]	1222 ± 343 [863, 1834]
600mg QD	9330 ± 1847 [6555, 11964]	866 ± 145 [653, 1098]	10221 ± 2519 [7333, 13870]	795 ± 207 [601, 1221]

The maximum observed group mean AUC(0-24h) and Cmax values to date are 23,810ng.h/mL and 1,258ng/mL, respectively. The maximum individual AUC(0-24h) and Cmax values to date are 38,771ng.h/mL and 1,885ng/mL, respectively. Of note, (i) the highest exposures have been observed in the 300mg BID group, and (ii) there is no significant effect of sitagliptin on GSK1292263 exposures in these T2DM subjects.

1.2.2.3.1. PK Simulations based on available Part C data

PK simulations were performed using preliminary data from Part C (Table 7, n=5-7 in each group) to characterize the dose/exposure relationship for GSK1292263 in T2DM subjects and relate them to the toxicokinetic data from the 13-week dog study.

The probabilities of the steady state exposure in a T2DM subject exceeding the testicular no-effect threshold (AUC(0-24h) = 36,772 ng.h/mL and Cmax = 2,537 ng/mL) are shown in Table 8, and the probabilities of the steady state exposure in a T2DM subject exceeding the testicular effect threshold (AUC(0-24h) = 48,863 ng.h/mL and Cmax = 2,757 ng/mL) are shown in Table 9.

Table 8 Probability of Exceeding NoEeffect Threshold for Testicular Findings

Treatment	No Effect Cmax, ng/mL	No Effect AUC(0-24h), ng.h/mL
50 mg BID	~0%	~0%
150 mg BID	0.15%	0.7%
300 mg BID	1.1%	5.9%
600 mg QD	~0%	~0%

Table 9 Probability of Exceeding Exposures Associated with Testicular Findings

Treatment	Effect Cmax, ng/mL	Effect AUC(0-24h), ng.h/mL
50 mg BID	~0%	~0%
150 mg BID	0.2%	0.1%
300 mg BID	1.1%	0.8%
600 mg QD	~0%	~0%

Table 8 indicates that there is a small probability (<6%) that an AUC will exceed the no-effect threshold and Table 9 shows that there is a probability of <1% that an AUC will exceed the lowest exposure associated with the testicular effect in dogs following 13 weeks of dosing.

1.3. Metformin

1.3.1. Metformin Pharmacology

Metformin is an orally active drug that has been used for over 30 years for the treatment of T2DM. The actions of metformin are complex, and have been reviewed extensively in the literature [Correia, 2008; Kirpichnikov, 2002]. Metformin reduces hepatic glucose output and may increase peripheral insulin sensitivity. Although the precise biochemical mechanisms involved are not well characterized, these involve suppression of the

gluconeogenic and glycogenolytic pathways in the liver, and AMP kinase activation in skeletal muscle and other tissues.

More recently, metformin has been shown to increase circulating GLP-1 [Mannucci, 2004] , and this may play a significant role in controlling blood glucose in T2DM patients treated with this drug.

Metformin also reduces bile acid reabsorption in the distal ileum [Carter, 2003] , and this is thought to explain some of the gastrointestinal side-effects of the drug, as well as the lowering of LDL cholesterol seen in T2DM patients treated with this agent.

1.3.2. Absorption, Metabolism and Elimination of Metformin

Metformin is absorbed slowly from the upper gastrointestinal tract within ~6h (tmax ~2-3h), and has a bioavailability of ~40-60%. About 20-30% of a dose appears in the feces. The absorption of metformin is reduced by food. Metformin shows non-linear pharmacokinetics due to decreased bioavailability at higher doses. It is minimally bound to plasma proteins and it has a volume of distribution of ~1-4L; accumulating in the GI tract, salivary glands, kidneys and blood cells.

The oral half-life is ~2-6h.

Metformin is eliminated by glomerular filtration and active secretion (CLr -333-600 mL/min) of parent drug. Renal secretion involves a number of drug transporters, including the organic cation transporter-2 (OCT2) in human renal tubules. No metabolites of metformin have been identified in human plasma.

OCT1 in the liver is involved in the uptake of metformin into hepatocytes. However, OCT1 does not have a major effect on systemic pharmacokinetics of the drug, although it may play a role in determining metformin's effects on hepatic glucose output. Indeed, OCT1 knockout mice are resistant to the metformin-induced lactic acidosis and have markedly lower liver concentrations of metformin.

In general, only modest increases in metformin AUC and Cmax have been observed in clinical practice, even with inhibitors of OCTs such as cimetidine [**GLUCOPHAGE** Package Insert, 2009].

1.4. Sitagliptin

In this study, open-label sitagliptin is being used as a comparator to allow estimation of the relative efficacy of GSK1292263 on glycemic parameters when co-administered with metformin.

Sitagliptin (Januvia) is a marketed DPP-IV inhibitor that is approved for use as monotherapy or in combination with metformin for the treatment of T2DM. The recommended dose is 100mg once daily, which can be taken without regard to food, or 50mg BID when co-administered with metformin as Janumet.

The pharmacokinetics of sitagliptin are generally similar between healthy volunteers and T2DM subjects, with tmax occurring at 1-4 h, t½ approximately 12 hours, and AUC and Cmax approximately 3468ng.h/mL and 387ng/mL, respectively following a single 100mg dose.

Sitagliptin is mainly excreted unchanged in the urine (79%) with limited metabolism.

Additional information may be found in the sitagliptin Prescribing Information [**JANUVIA** Package Insert, 2009; **JANUMET** Package Insert, 2009].

By blocking DPP-IV, sitagliptin increases the systemic levels of the active form of the incretins, GLP-1 and GIP, and reduces the level of PYY_{3-36}, a form that has anorexigenic properties. At the same time, relative to placebo, it reduces the levels of total GLP-1, GIP and PYY via a putative negative feedback loop at the secretory L and K cells in the gut. In the FTIH study (GPR111596), a reduction in total GLP-1, GIP and PYY was observed when 100mg sitagliptin was administered to normal healthy subjects, compared to the levels observed with 250mg GSK1292263 alone.

1.5. Rationale

Data from this study will be used to assess the potential of the GPR119 agonist GSK1292263 as a treatment for T2DM, and will aid the design and dose selection of future studies of longer duration in T2DM subjects that will evaluate GSK1292263 alone or in combination with other anti-diabetic drugs, such as a DPP-IV inhibitor or metformin.

1.5.1. Study Rationale

This will be the first study of GSK1292263 in T2DM subjects who are taking metformin (≥1000mg/day), and is intended to assess the safety, tolerability, pharmacokinetic and pharmacodynamic profile of this investigational drug following single doses (Parts A), and subsequently 14 days of dosing in Part B.

In Part B, open-label sitagliptin (co-administered with metformin) is included as a comparator to provide data that will aid the assessment of the therapeutic potential of GSK1292263 when co-administered with metformin.

1.5.2. Dose Rationale

1.5.2.1. GSK1292263

Simulation of Exposures (Parts A and B)

Modeling and simulation were performed to predict exposures of GSK1292263 when co-administered with metformin for 1 (Part A) or 14 days (Part B). Key factors considered for these simulations were: accumulation upon repeat-dosing and increased exposure in the fed state. Given that lower exposures were observed in the metformin washout subject in Part B of study GPR111598 (Figure 2), these simulations provide conservative estimates of systemic exposure (higher exposure) and cover relative to exposure limits

based on non-clinical toxicology noted in Section 1.1.2.3. Currently, there are no data to suggest that combination of GSK1292263 with metformin would increase systemic exposure to GSK1292263 or metformin.

A nonlinear-mixed effects pharmacokinetic model was developed based on data generated in the fed state from study GPR111596 (healthy subjects) and available PK data from GPR111598.

The final model was used to simulate a range of possible doses of 25mg to 800mg QD and 25mg to 400mg BID with food. Simulations provided predictions of exposures [AUC(0-24h), and Cmax] after a single dose in Part A and on Day 14 in Part B. In addition, the probability of exceeding group mean and individual limits for AUC(0-24h) and Cmax were simulated.

Considerations for Part B Dose Selection

As outlined in the protocol, the initial planned doses of GSK1292263 were 75mg BID and 300mg BID. In Amendment 1, the maximum allowable total daily dose of 600mg (300mg BID) was based on preliminary data from the 13 week toxicology study in the dog (Section 1.1.2.3 and Section 1.1.2.4). In Amendment 2, a 5th arm is added to Part B to evaluate the safety, tolerability, PK and PD of 600mg QD GSK1292263 when co-administered with metformin.

The following factors have been considered to arrive at the doses and regimens for Part B:

- BID dosing is used to coincide with metformin dosing.
- QD dosing is added in Amendment 2 to allow comparison of the effects of 600mg QD of GSK1292263 when administered as monotherapy to T2DM subjects washed off prior anti-diabetic medications (Part C of study GPR111598) and as an add-on to metformin (Part B of this study GPR113132).
- The broadest spectrum of doses should be investigated within the safety limits available:
    - The maximal dose should maintain exposures within the toxicokinetic boundaries specified in the protocol (see Section 1).
        - The simulations account for the effect of food on exposure, accumulation over multiple days of dosing.
    - GSK1292263 exposure estimates are conservative given the potential for reduced exposures when administered with metformin.
    - In addition, the probability of exceeding the safety exposure limits was set at <5% (<1:20 subjects exceed the limit). Note that the cohorts will enroll approximately 12-16 subjects, so it is not anticipated that a subject will cross the conservative toxicokinetic limits.
- Luminal drug levels may need to be high to stimulate gut hormone secretion.

Proposed Doses to be Investigated

Predicted GSK1292263 exposures and the ratios of the toxicology limits to predicted exposures are summarized in Table 10 and Table 11.

Table 10 Simulated Exposures (Mean ± SD) in Part A*

Dose (QD)	AUC(0-24h), ng.h/mL	Cmax, ng/mL
300 mg	4906 ± 2591	436 ± 249

*Simulations predict ~0% probability that subjects will exceed group mean or individual exposure limits following administration of a 300mg single dose based on 14 day toxicology studies in the dog and rat.

Table 11 Simulated Exposures (Mean ± SD) in Part B*

Dose (BID)	AUC(0-24h), ng.h/mL		Cmax, ng/mL	
	Day 1	Steady-State	Day 1	Steady-State
75 mg	3859 ± 1998	6018 ± 3078	305 ± 166	430 ± 223
300 mg	8316 ± 4219	13436 ± 6875	646 ± 347	945 ± 490

*Simulations predict ~0% probability that subjects will exceed group mean or individual exposure limits based on the 14-day and 13-week toxicology studies in the dog and rat.

The dose of GSK1292263 to be tested in Part A is 300mg and is considered adequate to characterize any potential effect of metformin on the pharmacokinetics of GSK1292263. The planned doses of GSK1292263 for Part B are 75mg BID and 300mg BID co-administered with metformin, and Amendment 2 adds a dose of 600mg QD co-administered with metformin. Doses for Part B were selected to (1) provide a range of exposures to allow estimation of the exposure/response relationship for GSK1292263, and (2) to investigate the PD of GSK1292263 at the highest allowable exposures, while maintaining exposures within limits established by non-clinical toxicology studies. Based on data from Part A, the doses to be administered in Part B may be modified up to a maximum total daily dose of 600mg. A dose of 300mg BID, or a dosing regimen resulting in equivalent exposure, given for up to 14 days does not pose a significant clinical risk from the perspective of either mean or individual exposures to date.

1.5.2.2. Metformin

The dose of metformin to be administered in the current study will be ≥1000mg administered as divided doses (BID) with GSK1292263, sitagliptin, or placebo. Seven days before enrollment in Part B, subjects taking metformin TID or using an extended-release formulation will be converted to the equivalent total daily dose of immediate release metformin administered BID. The actual dose of metformin may vary across subjects but will be consistent with the dose at which each subject was stabilized prior to enrolment in the study.

1.5.2.3. Sitagliptin

Subjects in Part B will be randomized to GSK1292263, placebo, or open label 50mg sitagliptin, all administered BID with open label metformin (at the subject's usual dose).

This is the recommended dose of sitagliptin when co-administered with metformin for the treatment of T2DM in subjects without contraindications.

1.6. Summary of Risk Management

1.6.1. Risks Related to Washout of Anti-Diabetic Medications

GSK1292263 has only been evaluated in conjunction with one approved anti-diabetic medication, sitagliptin (as a single 100mg dose). This will be the first assessment of the safety, tolerability, PK and PD of GSK1292263 in T2DM subjects taking established metformin therapy.

Subjects in Part B will be allowed to enter the study on their usual total daily dose of metformin. By enrolling subjects previously maintained only on metformin monotherapy, it is anticipated that this will not cause significant deterioration of glycemic control during the approximately 2 weeks' duration of Part B.

The risk that glycemic control will deteriorate in Part B is partially mitigated by the fact that one group of subjects will receive sitagliptin at the dose approved for the treatment of T2DM (50mg BID as Janumet). In addition, in the T2DM study GPR111598, a single dose of 800mg GSK1292263 reduced glucose AUC during an OGTT similarly to 100mg sitagliptin.

1.6.2. Risks Related to GSK1292263

GSK1292263 was generally safe and well-tolerated when administered in single doses up to 800mg in the fasted and fed states in studies GPR111596 (healthy volunteers) and GPR111598 (T2DM subjects), and when administered at a dose of 250mg for 2 or 5 days to healthy volunteers (GPR111596), and doses of 50mg BID, 150mg BID and 300mg BID to T2DM subjects (GPR111598).

In this study, subjects will be carefully evaluated prior to, during and after dosing. In Part A, 4 subjects will be dosed in the unit under close supervision, and will remain there until all 24hr post-last-dose assessments, including safety, have been completed. Adverse event data, including clinically significant abnormalities of clinical labs, vital signs and ECGs will be reviewed by the GSK Medical Monitor and study team, and the Investigator prior to dosing in each period. Amendment 1 allows up to 8 subjects to complete study assessments in Part A, if required to define the PK of single dose GSK1292263 when co-administered with metformin.

In Part B, subjects will remain in-unit under close supervision from Day -2 through the morning of Day 15 (final checkout). Clinical chemistry, hematology, urinalysis, vital signs, and ECGs will be monitored at regular intervals throughout this part of study.

After a subject has passed successfully through screening, glucose levels will be evaluated by capillary blood glucose (CBG) or venous or blood plasma glucose monitoring at least once daily (fasting pre-breakfast meal) prior to dosing a study drug, and at least twice daily (fasting and pre-evening meal) when the subject is in the unit. In addition, subjects will be asked ‘how are you feeling’ to provide an early indication of potential of neuroglycopenia.

At the discretion of the Investigator, subjects will have IV access during the dosing period for rapid treatment of hypoglycemia, if required while in the clinical unit.

Stopping criteria for these parameters are specified in Section 1.6.5.

The risk in T2DM subjects of clinically-significant hypoglycemia due to GSK1292263 is believed to be low because:

- The mechanism relies on glucose-dependent insulin secretion.
- GSK1292263 increases glucagon secretion during insulin-induced hypoglycemia in animal studies.
- No fasting hypoglycemia was observed 24h after dosing in the 2 previous studies (including a single day of co-administration of GSK1292263 and sitagliptin 100mg to healthy volunteers and subjects with T2DM).
- T2DM subjects who are not at glycemic goal should be at little risk of hypoglycemia.

Details of any hypoglycemic episodes will be captured in the Case Report Form.

While the maximum allowable total daily dose in the current study was 800mg based on a number of impurities in the drug substance, (current IB [GlaxoSmithKline Document Number RM2008/00434/00] and Supplement2 of the IB [GlaxoSmithKline Document Number RM2009/00168/01]), Amendment 1 limited the maximum total daily dosage for Part B in this protocol to 600mg (300mg BID) based on the preliminary data from the 13-week toxicology study in the dog. (See Section 1.6.2 and Section 1.6.3).

In Amendment 2, a 5th arm is added to Part B to evaluate the safety, tolerability, PK and PD of 600mg QD GSK1292263 when co-administered with metformin. A single dose of 800mg QD GSK1292263 was generally safe and well tolerated in Part A of study GPR111598. In addition, based on data from Part C of study GPR111598 (Table 7), the systemic exposures of GSK1292263 expected from 600mg QD GSK1292263 co-administered with metformin are anticipated to be well below the exposures achieved with 300mg BID.

1.6.2.1. Risk Assessment based on the Preliminary Observations from the 13-week Toxicology Studies in Rats and Dogs

There were no treatment-related effects in liver or testes in the rat and dog up to 2000mg/kg/day and 1000mg/kg/day GSK1292263, respectively, at systemic mean exposures of 58,405ng·hr/mL (male rats) and 49,505ng·hr/mL (male dogs) on Day 14 of the 14-day toxicology study. In addition, stage-dependent qualitative evaluation of spermatogenesis demonstrated normal progression up to the limit dose in both species indicating there were no subtle morphological changes in the testes following 14 days of dosing in the 14-day toxicology study. In the 13-week rat study (10, 150, 2000mg/kg/day) there were no treatment-related findings in liver or testes up to 43,551ng.hr/mL (mean male rat AUC0-24hr).

For single dose administration of GSK1292263 in clinical subjects, preclinical data support mean clinical exposures up to 49,400ng.hr/mL (as described in the current Investigator Brochure, [GlaxoSmithKline Document Number RM2008/00434/00]).

Testis: In the ongoing clinical trials there are no means to readily monitor for the testicular effect observed in the 13-week dog study. The mean clinical AUC achieved at 300 mg BID (23,810ng.h/mL), the highest intended dose, is below the no-effect AUC for the testicular effect in any dog in the 3-month study (36,772ng.h/mL), and is less than 50% of the mean no-effect AUC in the dog 14-day study (49,505ng.h/mL).

The highest individual clinical AUC achieved at 300mg BID (38,771ng.h/mL) is well below the highest exposure achieved in an individual dog at the NOAEL following 14 days of dosing (78,414ng·hr/mL), and is also less than the lowest individual dog AUC for a testicular effect following 3 months of dosing (48,863ng.h/mL). Although 38,771ng.mL does exceed the highest individual no-effect exposure in the 3-month dog study (36,772ng.h/mL), the dog testicular change is regarded as a time-dependant effect and the intended clinical dose duration is 1/6 of the duration that caused testicular toxicity (14 versus 90 days) in a single species.

Liver: There are safety margins based on systemic exposure (AUC) for the liver effects observed in the 13-week dog study 4.5-fold at the effect dose of 1000mg/kg/day (107,344ng.hr/mL); 1.7-fold at the no-effect dose of 20mg/kg/day (40,217ng.hr/mL), compared to the clinical AUC at 300 mg BID) (Table 1). Importantly, liver effects are readily monitorable and liver function monitoring and withdrawal criteria are already in place in this study.

In summary, the doses of GSK1292263 being evaluated in Part B of this study (top BID dose limited to 300mg BID, or a dosing regimen resulting in equivalent exposure) do not pose a significant clinical risk from the perspective of either mean or individual exposures to date.

1.6.2.2. Overall Assessment of GSK1292263 Risk

Overall, the potential risk to subjects who receive GSK1292263 is considered low because:

- The likelihood that plasma exposures will reach levels of toxicological concern is small based on prior data from healthy volunteers and T2DM subjects (see Section 1.2.1 and Section 1.2.2). Furthermore, PK data from Part A of the current study will be used to refine the exposure simulations that may be observed in Part B with the planned doses of GSK1292263 (75mg BID and 300mg BID).
- So far, GSK1292263 has been generally well tolerated in healthy subjects at doses ≤ 400mg and T2DM subjects at single doses up to 800mg and multiple doses up to 300mg BID, with no clinically significant changes in vital signs, ECGs, telemetry and lab parameters related to the study drug.
- The subjects will be dosed in the clinical unit and observed closely for 24h for the single dose and remain in house for two weeks during the dosing period with appropriate monitoring of clinical status, labs, vital signs, etc to decrease risk.
    - There is no indication that the mechanism of action of this drug would predict an increased risk of hypoglycemia in subjects with T2DM, and this was confirmed in Parts A and B of study GPR111598 in T2DM subjects.
- The drug substance purity levels comply with current guidelines for short-term studies.

1.6.3. Risks related to GSK12922263 and Metformin Co-Administration

1.6.3.1. Potential for PK Interaction

As discussed in Section 1.1.2.2 and Section 1.3.2, GSK1292263 is extensively metabolized, whilst metformin undergoes renal excretion via OCT2 and is eliminated as parent drug.

GSK1292263 is not expected to have a significant inhibitory effect on OCT2 at the exposures predicted for this study, so there is a low probably that this drug will alter the PK of metformin.

However, there is a theoretical possibility that metformin may reduce GSK1292263 exposure because (i) metformin inhibits bile acid reuptake and may reduce bile acid secretion with food, and (ii) bile acids secreted with food may help to solubilize GSK1292263 and increase its exposure when taken with food.

1.6.3.2. Potential for PD Interaction

OCT1 in the liver is involved in the uptake of metformin into hepatocytes. Because GSK1292263 is a weak inhibitor of OCT1 (69% at 30µM), there is a theoretical possibility that this drug will reduce the uptake of metformin into the liver and hence its efficacy at reducing hepatic glucose output. However, following a 800mg QD dose, the Cmax of GSK1292263 is 1009ng/mL (2.2µM; Table 6), which is over 10-fold lower than the concentration used in the OCT1 inhibition assay. The plasma exposure, in conjunction with the high protein binding of ~99%, suggest that GSK1292263 is unlikely to inhibit OCT1 *in vivo*, but liver concentrations may be higher.

1.6.4. Risks Related to Sitagliptin and Sitagliptin-Metformin Co-administration

In this study, sitagliptin (Januvia) is being used as an open label comparator to GSK1292263. Sitagliptin is approved as monotherapy and as a fixed-dose combination with metformin (Janumet) for the treatment of T2DM. The most common side effects are described in the Januvia and Janumet Package Inserts [**JANUVIA** Package Insert, 2009; **JANUMET** Package Insert, 2009] .

Recently the FDA has issued the following information for healthcare professionals relating to 88 post-marketing cases of acute pancreatitis (including two cases of hemorrhagic or necrotizing pancreatitis) in patients using sitagliptin that were reported between October 16, 2006 and February 9, 2009:

http://www.fda.gov/Drugs/DrugSafety/PostmarketDrugSafetyInformationforPatientsandProviders/DrugSafetyInformationforHeathcareProfessionals/ucm183764.htm

Thirteen of the 88 cases occurred within 30 days of starting sitagliptin treatment.

The FDA has issued the following considerations for healthcare professionals:

- Be aware of the possibility for and monitor for the emergence of the signs and symptoms of pancreatitis such as nausea, vomiting, anorexia, and persistent severe abdominal pain, sometimes radiating to the back.
- Discontinue sitagliptin or sitagliptin/metformin if pancreatitis is suspected.
- Understand that if pancreatitis is suspected in a patient, supportive medical care should be instituted. The patient should be monitored closely with appropriate laboratory studies such as serum and urine amylase, amylase/creatinine clearance ratio, electrolytes, serum calcium, glucose, and lipase.
- Inform patients of the signs and symptoms of acute pancreatitis so they are aware of and able to notify their healthcare professional if they experience any unusual signs or symptoms.

1.6.5. Capillary Blood Glucose (CBG) Monitoring

Subjects who complete successfully the screening procedures prior to dosing in Part B must check fasting capillary glucose at least once daily (fasting pre-breakfast), and at any time symptoms of hypoglycemia or hyperglycemia are experienced. A written diary card should be kept for each of these subjects.

Fasting capillary glucose values >270mg/dL or <70mg/dL must be reported to the site at once. If fasting glucose levels are >270mg/dL or <70mg/dL on any two consecutive days, the subject will be discontinued and appropriate anti-diabetic therapy reinstated. All subjects will be supplied with glucometers and strips to use during washout and the dosing period, if not already available.

While in-clinic, fasting glucose and pre-evening meal glucose will be monitored daily by CBG, venous blood or plasma glucose.

When subjects are in the unit they should alert study staff any time they experience symptoms that might be related to hypoglycemia. Study staff should then test their blood glucose values. Hypoglycemia documented by a blood glucose ≤ 50mg/dL (capillary or venous), or any hypoglycemia requiring assistance should be assessed and a decision made, in consultation with the GSK Medical Monitor, whether or not to withdraw the subject from the study.

Subjects are required to call the study center while not in the unit:

- When they have CBG values that are >270mg/dL or < 70mg/dL (when self-monitoring CBG outside of the study center)
- When they have any concerns relating to their CBG levels
- When they have rapid, unexplained changes in their blood glucose levels

Subjects are required to alert site staff while in the unit, or to call the study center while not in the unit:

- When they have symptomatic hypoglycemia, even if not confirmed by their blood glucose values

For the purpose of this protocol, ‘hypoglycemia’ is defined as an AE for any confirmed blood glucose value ≤50mg/dL, which may or may not be symptomatic. Episodes of hypoglycemia unconfirmed by blood glucose measurement (≥50mg/dL or not checked) may be recorded as an AE if the subject experiences typical symptoms and signs consistent with their usual hypoglycemic episodes.

2. OBJECTIVE(S)

2.1. Primary

- To investigate the safety and tolerability of GSK1292263 as single dose (Part A) and repeat oral BID or QD doses (Part B) when co-administered in subjects with T2DM already taking metformin.
- To determine the pharmacokinetic parameters of GSK1292263 in subjects with T2DM already taking metformin following single dose (Part A) and repeat oral doses administered BID or QD (Part B).
- To evaluate in T2DM subjects already taking metformin the pharmacodynamic effects of GSK1292263 following single dose (Part A) and repeated oral doses administered BID or QD (Part B), and the pharmacokinetic/pharmacodynamic relationships.

2.2. Exploratory

- To determine the pharmacokinetic parameters of sitagliptin and metformin in subjects with T2DM taking metformin following repeat oral doses administered BID (Part B).
- To investigate the mechanism of action of GSK1292263.
- To compare, in T2DM subjects already taking metformin, the PD effects of GSK1292263 to those of sitagliptin (Part B).

3. ENDPOINT(S)

3.1. Primary

- Safety and tolerability parameters following single and repeat doses of GSK1292263 administered BID or QD, including adverse events, and assessments of clinical laboratory, ECGs and vital signs.
- Pharmacokinetic parameters following a single dose of GSK1292263 in Part A: Cmax, Tmax, t½, tlag, CL/F, V/F, AUC(0-24h), and AUC(0-∞). For repeat-dosing (BID or QD) in Part B the following PK parameters will be calculated: Cmax, Tmax, tlag (Day 1 only),AUC(0-10h) (BID regimen only), AUC(0-24h), and t1/2 (Day 14 only) accumulation ratio (Ro), as data permit.
- Pharmacodynamic/biomarker endpoints will include fasting and meal-related incremental and weighted mean AUC for glucose and insulin, as well as insulin secretion and insulin sensitivity parameters, as data permit. Relationships between GSK1292263 drug exposures and pharmacodynamic parameters (e.g., glucose, insulin), safety (e.g., QTc), and tolerability will be evaluated, as appropriate.

3.2. Exploratory

- In Part B the following sitagliptin and metformin PK parameters will be calculated: Cmax and AUC(0-24h).
- Pharmacodynamic/biomarker endpoints will include fasting and meal-related weighted mean AUC for glucose, insulin, glucagon, GLP-1 (active and total), C-peptide, total GIP, and total PYY, as data permit.
- Correlation between drug dose or exposure and parameters of pharmacodynamics, safety, and tolerability.
- Pharmacodynamic/biomarker endpoints including measures of insulin sensitivity, beta cell function, GI peptide profiles and other exploratory lipidomic, peptidomic and metabolomic biomarkers, as data permit.
- Exploratory analyses relating to biomarkers of T2DM or related metabolic diseases, biomarkers of GSK1292263, sitagliptin pharmacodynamics or safety may be performed using (i) small molecular weight metabolites, including, but not limited to, branch chain amino acids, acylcarnitines, and lipids, (ii) blood polypeptide analytes including, but not limited to, leptin, ghrelin, adiponectin, and (iii) novel biomarkers derived from peptidomic, lipidomic and metabolomic analysis of blood.

4. INVESTIGATIONAL PLAN

4.1. Study Design/Schematic

Figure 3 Study Schematic

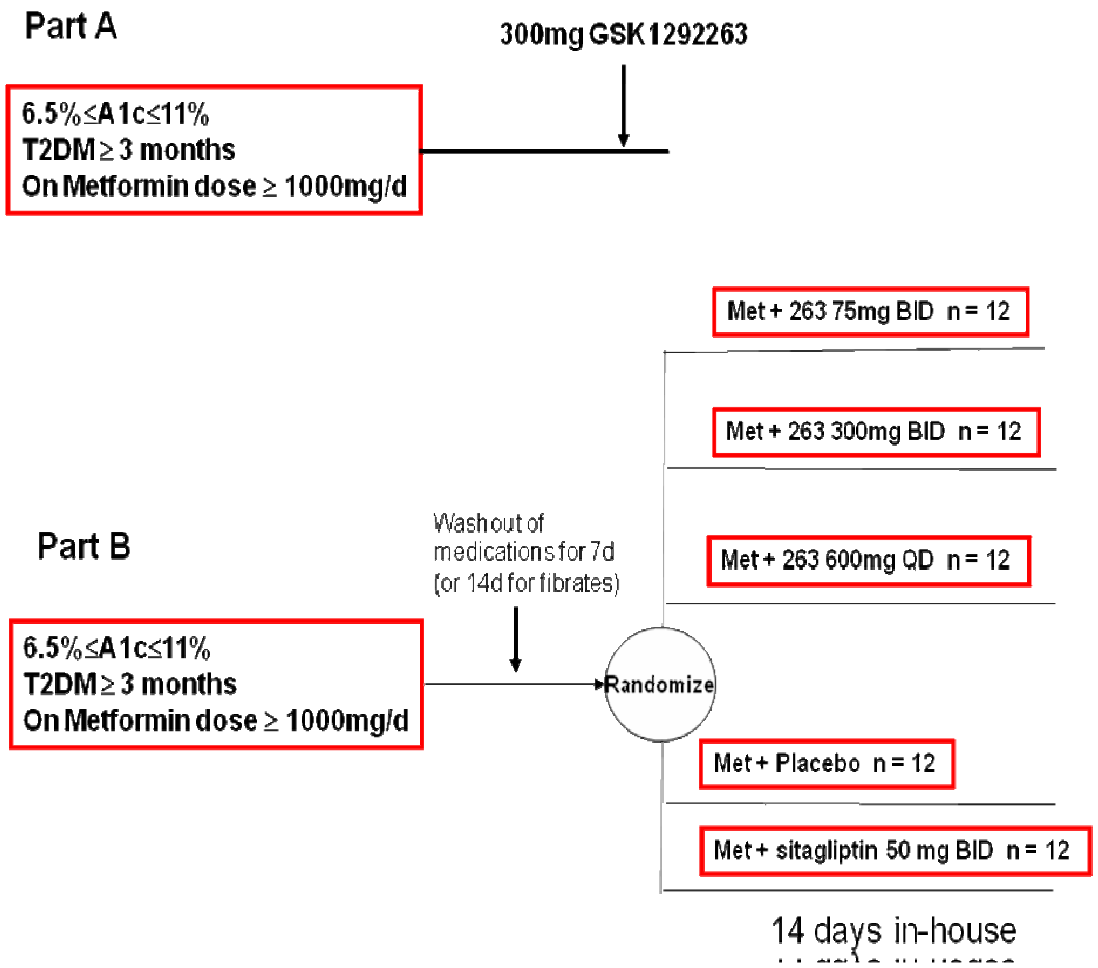

4.2. Discussion of Design

4.2.1. Part A: Assessment of Single Dose PK of GSK1292263 in T2DM Subjects Taking Metformin

Part A is open label, in 4 T2DM subjects on established metformin monotherapy. Subjects will receive a single dose of 300mg of GSK1292263 with food. This will permit a comparison of GSK1292263 exposures in this cohort with those observed in study GPR111598 in which T2DM subjects were drug naïve or washed off prior anti-diabetic medications. Amendment 1 allows up to 8 subjects to complete study assessments in Part A, if required to define the PK of single dose GSK1292263 when co-administered with metformin.

The planned 300mg dose selected for Part A will allow for comparison to prior studies to evaluate evidence of any pharmacokinetic interaction with metformin. This dose may be changed based on emergent safety, tolerability and PD data from the on-going Part C of study GPR111598.

A limited assessment of pharmacodynamics will be preformed to allow PK-PD analyses that will inform the dose selection for Part B.

Subjects will undergo screening procedures within 28 days of the first dosing period.

As shown in Figure 3, subjects will be admitted to the research facility and dosed with GSK1292263 after successfully completing all screening and baseline assessments.

Subjects will be admitted to the research facility within 24 hours prior to the dose period and will remain in unit for 24hr post dosing. They will return to the unit 48 hr after dosing to provide a blood sample for analysis of GSK1292263 and metformin concentration. A follow-up visit will occur between 7 and 10 days after the last dose.

The maximum amount of time a subject can expect to spend in this part of the study is approximately 6 weeks, including the 28-day period allotted for screening assessments.

4.2.2. Part B: Repeat Dosing of GSK1292263 to T2DM Subjects Taking Metformin

Part B is a single-blind, randomized, placebo-controlled, 5-arm cohort of 60 subjects dosed for 14 days with one of 3 doses of GSK1292263, placebo or open-label sitagliptin 50mg BID. It is being conducted to assess safety, tolerability, PK and PD of GSK1292263 and open-label sitagliptin after 14-days of dosing in T2DM subjects already taking metformin monotherapy.

The planned doses of GSK1292263 in Part B are 75mg BID, 300mg BID, and 600mg QD. Modeling and simulation indicates these doses will provide an estimate of the safety, tolerability, PK and PD of GSK1292263 in T2DM subjects taking metformin and will not exceed exposure limits based on non-clinical toxicology studies. These doses may be changed based on emergent safety, tolerability, PK and PD data from Part A of this study, as well as the on-going Part C of study GPR111598, but the maximum total daily dose will not exceed 600mg (300mg BID or 600mg QD).

Ethics boards will be advised of the final doses chosen for Part B prior to the dosing of subjects. Safety, tolerability, PK and PD data from Part A will be assessed prior to initiation of Part B.

Figure 4 Schematic of Part B

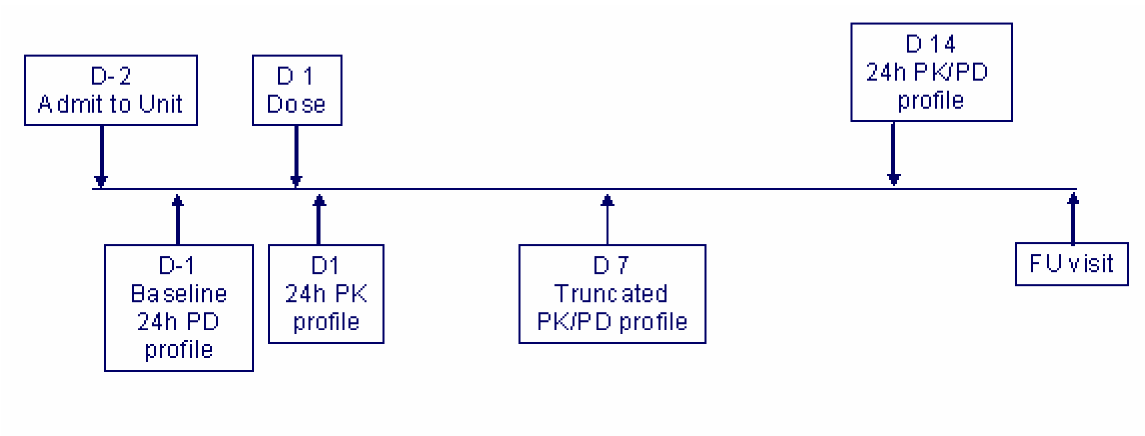

Subjects enrolled into Part B will have been on a stable dose of metformin monotherapy for at least 1 month.

Subjects from Part A may also participate in Part B if, (i) they meet all inclusion/exclusion criteria, (ii) there is a washout period for GSK1292263 of ≥2 weeks between Part A and participation in Part B (based on the t½ of GSK1292263 observed in T2DM subjects of ~12 - 17h), and, (iii) the Investigator considers the subject will not be compromised by the additional blood sampling in Part B.

Subjects will undergo screening procedures within 28 days of first dose. Subjects will be randomized upon admission to the research facility after successfully completing all screening and baseline assessments.

As shown in Figure 4, subjects will be admitted to the research facility within 48 hours (Day -2) prior to the first dose (Day 1). The 24-hour time period prior to dosing (Day-1) will be defined as the baseline, and the treatment period will be defined as Days 1-14. Full profiles for PK will be conducted on Days 1 and 14, for GSK1292263, but only on Day 14 for sitagliptin and metformin concentrations. Full 24hr profiles for PD will be conducted on Days -1 and 14. For GSK1292263, truncated PK profiles will be obtained on Day 7. Truncated PD profiles will be obtained for all dosing arms on Day 7.

On Day 14, subjects will have PK and PD assessments, and will remain in the unit for 24hr post dosing. Subjects may be discharged from the unit on the morning of Day 15, following all post-last dose safety, PK and PD assessments. The subjects will return 48hr after the last morning dose to provide a sample of PK analysis.

A follow-up visit will occur between 7 and 10 days after the last dose in each cohort.

The maximum amount of time a subject can expect to spend in this part of the study is approximately 8 weeks, including the 28-day period allotted for screening assessments.

Subjects will be administered doses that are expected to be safe and well-tolerated based on data from studies GPR11956 and GPR111598.

During the dosing period, subjects will eat standardized meals. See the Study Procedure Manual (SPM) for specific instructions.

4.2.3. Screening

To determine subject eligibility for enrollment in the study, a screening visit will be performed within 28 days of first dose administration. For the purposes of subject eligibility for enrollment in the study, screening assessments are defined as any assessments performed prior to the first dose of study drug, including baseline assessments on Day -1 (Part A) or Day -2 (Part B) that are used to qualify the subject for enrollment.

If a subject is not eligible for the study based on the Inclusion and Exclusion Criteria at the initial attempt, but becomes eligible at a later date during the enrollment period, the investigator should contact the GSK Medical Monitor to discuss the possibility of re-screening the subject.

4.2.4. Washout

4.2.4.1. Part A

Subjects enrolled in Part A must be on metformin monotherapy as their only anti-diabetic medication.

4.2.4.2. Part B

In order to evaluate the effect of GSK1292263 on plasma lipids, subjects who qualify for Part B of the trial will begin washout of fibrate medications 14 days prior to Day 1 (Day -14). All subjects who will be washing off of approved medications will return to the clinic 7 days prior to Day 1 (Day -7). If relevant, at this visit, (i) subjects will discontinue use of concurrent lipid lowering agents such as statins, fat absorption blocking agents, and bile acid sequestrants, and will remain off of these medications until discharged from the clinic after the final post-last-dose study assessments (Day 15 in Part B), and/or (ii) TID or extended release metformin will be converted to the equivalent BID total daily dose of immediate release metformin.

4.2.5. Treatment regimen

4.2.5.1. Part A

T2DM subjects on metformin monotherapy will enroll into Part A, check into the unit on Day -1 and receive a single dose of 300mg of GSK1292263 immediately after eating the breakfast meal.

4.2.5.2. Part B

Subjects will be randomized to 14 days of dosing with one of three dosing regimens of GSK1292263 (doses: 75mg BID, 300mg BID and 600mg QD) or matching placebo BID, or open-label sitagliptin 50mg BID. Subjects will check into the unit on Day -2, followed by 24h PD assessments on Days -1 and 14, and PK assessments on Days 1 and 14. Truncated PK and PD profiles will also be obtained on Day 7. PK samples will be collected for all subjects and will be analyzed for GSK1292263 concentrations on Days 1, 7 and 14. Sitagliptin PK will be measured only on Day 14 for subjects in the sitagliptin arm. Metformin PK will be measured on Day 14 for all subjects.

After completion of Part B, the prior medications to be restarted may be modified as needed by the Investigator, who should review laboratory results obtained during the 14-day dosing period to determine whether adjustments to these prior medication doses should be made.

4.2.6. Follow-up Visit

A follow-up visit will occur 7 to 10 days following discharge from the clinic from either Part A or Part B. Any subject withdrawing from the trial prematurely should also be asked to complete follow-up procedures 7-10 days after withdrawal.

See Section 4.7 and Section 4.8 for details regarding all study procedures performed during the course of this trial.

4.2.7. Pharmacodynamic Testing

4.2.7.1. Meal Tolerance Test (MTT) – Part B only

On Days -1, 7 and 14 in Part B, subjects will be administered a standardized MTT at breakfast, lunch and evening meal time. Blood samples will be collected up to 3h post each meal to define the profiles of glucose, insulin and related biomarkers indicated in Section 4.2.7.3. Limited PD and PK sampling will be conducted on Day 7.

The composition of the meals for the MTTs and timing relative to dosing are defined in the SPM.

On Day 1 (full PK sampling day), all meals will be of the same composition as those on the PD profiling days, but no PD samples will be taken.

4.2.7.2. Hunger, Craving and Fullness Questionaire and Caloric Intake (Part B)

GPR119 agonists elevate incretins and PYY in the human and these may affect food intake. For this reason, this study includes assessments of eating behaviors and calorie intake.

The modified Hunger, Craving and Fullness Questionnaire (HCFQ) (see Appendix 3) is designed to assess changes in eating behaviors as a result of study treatment. The original HCFQ instrument was developed by GSK in collaboration with Mapi Values and underwent psychometric evaluation by Oxford Outcomes. The modified HCFQ

questionnaire is a self-administered questionnaire that will be used as an exploratory measure to understand patient-reported feelings of hunger and satiety.

The modified HCFQ is comprised of 7 items to assess hunger, craving, and fullness. Patients rate each item on a Likert scale comprising 5 responses. The modified instrument has not undergone psychometric evaluation.

The modified HCFQ will be administered after dinner on Days -1, 7 and 14, (see SPM for details). Any subject who withdraws early from the study should complete the modified HCFQ at withdrawal.

If feasible, on Days -1, 7 and 14, calorie counts will be obtained across the meal periods and during the prior 24h (note that the subjects are not required to eat all of the meals on these days).

4.2.7.3. Other Pharmacodynamic Profiling

The oral ingestion of a meal stimulates the release of incretins, such as GLP-1 and GIP, and other gut hormones, including PYY.

Assessment of baseline and 24-hour post-dose glucose, insulin, C-peptide, glucagon, GLP-1 (total and active), GIP, and PYY (total) may be conducted in Part A if blood volume permits.

In Part B, assessment of 24-hour glucose, insulin, C-peptide, glucagon, GLP-1 (total and active), GIP (total), PYY (total) at baseline (Day -1), and on Days 7 and 14 will allow evaluation of the pharmacodynamic effect of repeat-dosing of GSK1292263 versus placebo and sitagliptin, and will permit comparison of the two dose levels of GSK1292263. Other biomarkers, such as glycerol, may be assessed at the same timepoint.

4.2.7.4. Exploratory Biomarkers

Pharmacodynamic blood sample(s) also may be analyzed in Part B for metabolomic, lipidomic and proteomic analyses, as blood volume limits permit, to investigate how fingerprints of these analytes change during treatment and whether they provide insights into the biochemical mechanisms involved. As new data emerge, it may also be possible to probe novel aspects of the biology of T2DM or related metabolic disorders such as obesity and the metabolic syndrome, as well as the biological and clinical responses to GSK1292263. If relevant, this approach will be extended to include the identification of biomarkers associated with adverse events.

The biomarker research activities may include measurement of, (i) known biomarkers that have been associated with T2DM and/or related metabolic disorders, and (ii) novel biomarker discovery activities related toT2DM, and/or related metabolic diseases.

Biomarkers may include, but are not limited to, (i) small molecular weight metabolites, including branch chain amino acids, acylcarnitines, and lipids, (ii) blood polypeptide

analytes including leptin, ghrelin, adiponectin, and (iii) novel biomarkers derived from peptidomic, lipidomic and metabolomic analysis of blood.

Performance of these biomarker investigations may be conditional on the results of the clinical trial and samples may be selected for analysis on the basis of the clinical outcome. For example, samples may be selected for analysis based on the response to intervention. Because of their exploratory nature, these investigations may be performed irrespective of whether a pharmacodynamic response is observed.

Details of PD sample collection and processing are found in the SPM. The timings of all the PD collections may be adjusted on the basis of emerging PK or PD data from this study or other new information in order to ensure optimal evaluation of the PD endpoints.

Samples of blood may be stored for a maximum of 15 years after the last subject completes the trial for possible future assessments of additional biomarkers.

4.3. Treatment Assignment

All subjects in Part A will receive a single dose of 300mg GSK1292263.

Subjects will be assigned to study treatments in accordance with the randomization schedule generated by Discovery Biometrics, prior to the start of the study, using validated internal software.

Sites may assign screening numbers using their own schema. Screening numbers are not collected by GSK. For each part of the study, subjects will be identified at enrollment by a unique Subject Number that will remain consistent for the duration of that part of the study. This number will be used for subject identification throughout that part of the study. Each site will be provided a unique set of numbers in chronological order beginning with the lowest number. Once a subject number is assigned to a subject, it cannot be re-assigned to any other subject during the study. A list of subjects and their subject numbers will be provided by the sites for subjects who participate in both parts of the study.

A central randomization method will be employed in Part B. Upon confirmation of eligibility, investigators or designated staff will use a central system (e.g., phone or web-based) to randomize a subject. Once a randomization number is assigned to a subject, it cannot be re-assigned to any other subject during the study.

Subjects will be randomized to receive active treatment or placebo in Part B. If a subject is prematurely discontinued from the study and a replacement subject is to be recruited, a related replacement treatment number will be used to assign treatment to the replacement subject.

In Part B, subjects were randomized to receive an active treatment (one of two dose levels of GSK1292263 BID or sitagliptin 50mg BID) or placebo BID regimen in a ratio of 1:1:1:1. However, with the addition of GSK1292263 600mg QD arm, the ratio will change to 1:1:1:1:6 after 10 subjects per arm are randomized per the original randomization schedule. This will allow for a constant block size once the GSK1292263

600mg QD arm is added. Appropriate stratification will be made to prevent an enrolment bias based on screening BMI (≥ 30 and <30 kg/m^2).

4.4. Investigational Product Dosage/Administration

Product name:	Investigational Product	
	GSK1292263	Placebo
Formulation description:	Immediate Release Tablet, Round, White, Film Coated	Immediate Release Tablet, Round, White, Film Coated
Dosage form:	Tablet	Tablet
Unit dose strength(s)/Dosage level(s):	25mg, 75mg and 200mg	Placebo
Route/Administration/Duration:	Oral	Oral
Dosing instructions:	Dose orally with ~250mL water per protocol	Dose orally with ~250mL water per protocol
Physical description:	Round, white, plain faced, film coated tablet	Round, white, plain faced, film coated tablet
Device:	None	None
Manufacturer/source of procurement:	GSK	GSK

Metformin and sitagliptin will be administered as open label drug, and subjects will continue their usual metformin dose. The dose and time of administration of metformin and sitagliptin will be recorded.

The doses in Part B are planned to be administered as 14 days of dosing in conjunction with metformin, randomized into one of 5 groups:

- 75mg GSK1292263 BID
  as 1 x 75mg GSK1292263+ 2 placebo (AM and PM)
- 300mg GSK1292263 BID
  as 1 x 200mg + 1 x 75mg +1 x 25mg all GSK1292263 (AM and PM)
- 600mg GSK1292263 QD
  as 3 x 200mg GSK1292263 (AM) + 3 x Placebo (PM)
- Placebo BID
  as 3 x placebo tablets (AM and PM)
- Sitagliptin
  50mg BID (open label, unblinded)

4.5. Dose Adjustment Criteria

The planned doses in this study are 300mg as a single dose in Part A, and 75 mg BID 300mg BID, and 600mg QD in Part B. These doses were selected to allow for a robust characterization of safety, tolerability, PK and PD of GSK1292263 when co-administered with metformin in T2DM volunteers, while keeping within this toxicokinetics limits indicated in Section 1.1.2.4. If modification of doses is necessary, the highest total daily dose of GSK1292263 to be administered in the study will be 600mg (administered as 300mg BID or 600mg QD) based on preliminary data from the 13-week toxicology study in the dog (see Section 1.1.2.3 and Section 1.1.2.4).

This protocol allows some alteration from the currently outlined dosing schedule with the following limitations:

- Doses may be adjusted based on emergent safety, tolerability and preliminary pharmacokinetic and/or pharmacodynamic data from Part C of study GPR111598 in T2DM subjects, and well as safety, tolerability and preliminary pharmacokinetic and/or pharmacodynamic data from Part A or B of the current study that indicate that the exposure limits presented in Section 1.1.2.4 may be exceeded.. These dose adjustments may involve either an increase or a decrease in the planned dose, but all doses will be selected so as not to exceed a 600mg total daily dose of GSK1292263 and/or the mean or individual plasma exposures defined by the non-clinical toxicology studies. A maximum 6-fold exposure differential between the low and high doses in Part B will be allowed if modeling and simulation indicates that exposure limits based on the preclinical toxicology studies are unlikely to be exceeded even assuming a conservative approach using dose proportional increase in exposure.

The actual doses in Part B administered may be changed based on safety/tolerability data (including clinically significant abnormalities of clinical laboratory results, vital signs, and ECGs), and PK and PD data from study GPR111598 and Part A and B of the current study.

Study enrolment may be adjusted to include more subjects in a dosing group to further evaluate safety, pharmacokinetic and/or pharmacodynamic findings at a given dose level, or to add cohorts to evaluate up to 2 additional dose levels, not to exceed a total daily dose of 600mg. In the latter case, enrolment would be suspended to allow implementation of a new randomization schedule in the central system. The study procedures for these additional subject(s) or cohort(s) will be the same as those described for the other study subjects.

In all cases, ethics boards will be informed of the actual doses to be investigated or dosage changes prior to administration to the subjects.

4.6. Safety Criteria

4.6.1. Liver Chemistry Stopping Criteria

Liver chemistry threshold stopping criteria have been designed to assure subject safety and to evaluate liver event etiology during administration of investigational product and the follow-up period. Investigational product will be stopped if any of the following liver chemistry stopping criteria is met:

- ALT ≥ 3xULN and bilirubin ≥ 2xULN (>35% direct bilirubin) (or ALT ≥ 3xULN and INR>1.5, if INR measured).

  NOTE: serum bilirubin fractionation should be performed if testing is available. If fractionation is unavailable, urinary bilirubin is to be measured via dipstick (a measurement of direct bilirubin in urine, which would suggest liver injury).
- ALT ≥ 5xULN.
- ALT ≥ 3xULN if associated with the appearance or worsening of hepatitis or hypersensitivity such as fatigue, nausea, vomiting, right upper quadrant pain or tenderness, fever, rash or eosinophilia.

Subjects with ALT ≥ 3xULN **and** < 5xULN **and** bilirubin < 2xULN, who do not exhibit hepatitis symptoms or rash, can continue investigational product as long as they can be monitored weekly for 4 weeks, and after consultation with the GSK Medical Monitor.

Refer to Section 13, Liver Chemistry Follow-up Procedures, for details of the required assessments if a subject meets any of the above criteria.

4.6.2. QTc Withdrawal Criteria

A subject that meets the criteria below will be withdrawn from the study.

- QTcB or QTcF > 500 msec (machine or manual overread)
- If subject has bundle branch block then criteria is QTcB or QTcF > 530 msec. Subjects with left bundle branch block are not eligible for the study
- Prolongation of QTcB or QTcF by > 60 msec as compared to baseline

These criteria are based on an average QTc value of triplicate ECGs. If an ECG demonstrates a prolonged QT interval, obtain 2 more ECGs over a brief period, and then use the averaged QTc values of the 3 ECGs to determine whether the subject should be discontinued from the study.

4.6.3. Additional Withdrawal Criteria

- PR interval <100 or >220msec.
- QRS duration <55 or >120msec.
- Systolic blood pressure changes by more than 30mmHg and/or diastolic blood pressure changes by more than 20mmHg from pre-dose baseline on 3 occasions 5min apart. In these circumstances, a subject may be withdrawn, in consultation with the GSK Medical Monitor, if blood pressure remains elevated 6 hours later.

4.6.4. Blood Glucose Withdrawal Criteria

- If a subject experiences symptomatic hypoglycemia during the course of treatment, the relevant data should be evaluated closely and the clinical situation should be discussed with the GSK Medical Monitor. A hypoglycemic episode that requires withdrawal is defined as symptoms consistent with hypoglycemia which are confirmed by glucometer result or blood glucose sample <50mg/dL (consider withdrawal for any severe or serious adverse event of hypoglycaemia).
- If fasting plasma or blood glucose >280mg/dL, confirmed by repeat testing within 2 hours (in a fasting or non-fasting state).

Any subject with clinically significant changes in safety parameters not listed above or significant AEs thought to be drug related will be withdrawn and monitored until recovery. A full assessment by the Investigator and the GSK Medical Monitor will determine whether it is safe and ethical to continue dosing the remaining subjects.

4.7. Time and Events Table (Part A)

Procedure	Screening ≤28 days prior to first dose	Washout Days -14 to Day -2	Dosing Period				Follow-Up
			Day -1	Day 1	Day 2	Day 3	7 -10 days after discharge
Clinic Visit[1]	X		--------In-Clinic--------			X	X
Informed Consent	X						
Inclusion/Exclusion Criteria	X						
Demographics	X						
Medical History / Current Medical Conditions	X						
Complete Physical Exam	X						X
Weight	X			X			X
BMI Calculation	X						
Waist Circumference	X		X				
Urine Drug/Cotinine Screen	X		X				
Urine/ Breath Alcohol Screen	X		X				
Vitals [2]	X		X	X	X		X
24-hour Holter	X			X			
12-lead ECG[3]	X		X	X	X		X
Telemetry[4]				X			
Hematology/ Clinical Chemistry[5]	X		X		X		X
Urinalysis	X		X		X		X
Urine Pregnancy Test			X				
FSH / Estradiol[6]	X						
HIV / Hep-B/ Hep-C Serology	X						
PGx				X			
Study Medication Administration [7]				X			
Glucose and Insulin/PD Sampling[8]				X	X		
Washout / Glucometer[9]		X					
Standardized meals				X			
PK Blood Sampling[10]				X	X	X	
Glucometer readings[11]			X	X			
Con Med Assessment	X		X	X	X	X	X

Procedure	Screening ≤28 days prior to first dose	Washout Days -14 to Day -2	Dosing Period				Follow-Up
			Day -1	Day 1	Day 2	Day 3	7 -10 days after discharge
Adverse Event Assessment		X	X	X	X	X	X

1. Screening visit must be performed within 28 days of Day 1.
2. Assessment of vital signs (including blood pressure and heart rate) will be performed at one time point at Screening, at follow-up and pre-breakfast on Day -1. On Day 1, they will be taken at pre-breakfast, 1 hour, 3, 4, 6, 10, 16 and 24hours post-dose. Assessments should be made in triplicate at the pre-breakfast time point, and single assessments should be made at all other times. Assessments should be performed after resting in a supine or semi-supine position for at least 10 minutes.
3. ECGs will be taken at Screening, pre-breakfast on Day -1, on Day 1 (pre-breakfast, 1 hour, 2, 3, 4, 6, 8, 13, 24hours post-dose), and at follow-up. Assessments should be made in triplicate on Day 1 at the pre-breakfast time point, and single assessments should be made at all other times. ECGs should be taken while subject is supine. Additional ECGs may be taken at the discretion of the investigator as needed based on symptoms or telemetry findings.
4. Telemetry will be performed for 24 hours on Day 1, starting 1hr prior to breakfast.
5. Blood samples for safety will be collected at screening, fasting (Day -1), at 24hr post- dose (morning of Day 2), and at follow-up. Refer to Section 7.2.4 for specific laboratory parameters to be tested.
6. FSH and Estradiol tests will be performed for post-menopausal women if the clinical history is unclear.
7. Study drug will be administered immediately after eating breakfast, and lunch and dinner will be approximately 4 and 10 hours after dosing.
8. Blood samples for the determination of glucose and insulin will be collected at pre-breakfast on Day 1 and 24h post-dose. Refer to the Study Procedures Manual for additional details on the collection and processing of PD samples.
9. Subjects washing out of fibrates should be instructed to do so beginning on Day -14, but no visit occurs at that time.
10. Blood samples for the determination of PK will be collected at the following times (PK sample times may be changed based on observed PK profile, but the total number of samples will not change) on Day 1 Immediately pre-dose (time 0) and at 0.5, 1, 2, 3, 4, 6, 8, 13, 24 and 48 hours post-dose. Refer to the SPM for details on the collection and processing of PK samples.
11. Subjects should be checked by investigational staff during the day and night to ensure there are no findings consistent with hypoglycemia (e.g., cool, moist skin, and diaphoresis).

4.8. Time and Events Table (Part B)

Procedure	Screening	Period 1										Follow-up
	≤28 days prior to first dose	Days -14 to Day-3	Day -2	Day -1	Day 1	Days 2-6	Day 7	Days 8-13	Day 14	Day 15	Day 16	7 -10 days after discharge
Clinic Visit	X	Washout[5]	-----In Clinic-----								X	X
Informed Consent	X											
Inclusion/ Exclusion Criteria	X											
Demographics	X											
Complete physical	X											
Check into Clinic			X									
Brief physical			X									X
Medical/medication/ drug /alcohol history	X											
Weight	X				X				X			
Waist Circumference					X				X			
Height / BMI Calculation	X											
12-lead ECG[1]	X			X	X		X		X	X		X
24-hr Holter	X											
Vitals[2]	X			X	X	X	X	X	X	X		X
Urine drug/alcohol screen	X		X									
Urine pregnancy test			X									X
Clinical Chemistry / Hematology / Urinalysis[3]	X		X		X	X	X	X	X	X		X
FSH/ Estradiol[4]	X											
HIV/ Hep B / Hep C	X											
Washout / Glucometer		X	X	X								
PGx[6]					X							
Dosing – Randomized study med[7]					X	X	X	X	X			
Mixed Meal Tolerance Test [8]				X			X		X			
Modified HCFQ [9]				X			X		X			
Calorie counts				X			X		X			
Standardized meals				X	X	X	X	X	X			
Blood samples for glucose and insulin/PD[10]				X			X		X	X		
PK profile blood samples[11]					X		X		X	X	X	
PK Trough Samples[11]						X						
Resume Washed-out Meds[12]										X		

Procedure	Screening	Period 1										Follow-up
	≤28 days prior to first dose	Days -14 to Day-3	Day -2	Day -1	Day 1	Days 2-6	Day 7	Days 8-13	Day 14	Day 15	Day 16	7 -10 days after discharge
Checkout from clinic										X		
Concomitant Medication Review[13]	X	X	X	X	X	X	X	X	X	X	X	X
AE assessment			X	X	X	X	X	X	X	X	X	X

1. Single ECGs will be taken at Screening, pre-breakfast on Day -1 and at Follow-up. On Days 1, 7 and 14 ECGS will be taken pre-breakfast (fasting) and at 1, 2, 4, 6, 8, 12 and 24hours post-dose. Triplicate ECGs will be taken at the pre-breakfast time point, and single assessments should be taken at all other times. ECGs should be taken while subject is supine. Additional ECGs may be taken at the discretion of the investigator as needed based on symptoms or ECG findings.
2. Assessment of vital signs (including blood pressure and heart rate) will be performed at Screening, pre-breakfast on Days -1 to 14 in a fasting state early in the morning (prior to morning dosing on Days 1-14), and at Follow-up. On Days 1, 7 and 14, they will also be taken at 1, 3, 6, 9, 12 and 24 hours after the morning dose. At each time point, assessment should be performed after resting in a supine or semi-supine position for at least 10 minutes.
3. Blood samples for safety will be collected at screening, on Day -2 (can be non-fasting), and prior to breakfast (early in the morning, fasting) on Days 1, 7, and on Day 15 prior to checkout, (=24hrs post-dose), and at follow-up. In addition, blood samples for safety may be taken on Days 3 and 10 depending on the results from Part A. When this results in multiple samples at the same time point, only one sample will be collected (e.g., when 24hrs post-dose = pre-dose (time 0) for the next dose). Refer to Section 7.2.4 for specific laboratory parameters to be tested.
4. FSH and Estradiol tests will be performed for post-menopausal women where the clinical history in unclear.
5. Subjects washing out of fibrates should be instructed to do so beginning on Day -14, but no visit occurs at that time.
6. PGx sample may be taken at any time after the first dose.
7. Study drug will be administered immediately after breakfast and immediately after the evening meal, but prior to the 10hr PK sample (see SPM for details of sampling times relative to dosing times).
8. Composition of the meal tolerance tests is specified in the SPM.
9. Modified HCFQ is administered after dinner, at the same time of day, ±1 hour, on Days -1, 7 and 14.
10. Blood samples for the determination of glucose and insulin and other PD markers will be collected fasting pre-breakfast on Days -1 and 14, and then at 0.5, 1, 1.5, 2 and 3 hours post dose. For lunch (approximately 4h post morning dose) samples will be collected just before the meal and at the following times after starting each meal: 0.5, 1, 1.5, 2 and 3 hours.. For the evening meal (approximately 10h post morning dose), sampling should follow the sequence of sampling: PD sample immediately before meal, eat and then dose> Further samples are then taken at 0.5, 1, 1.5, 2 and 3 hours post dinner. A sample will also be collected 24 hours post-dose. On Day 7, samples for glucose and insulin and other PD markers will be collected fasting, pre-breakfast and at 1, 2, 4 (= pre lunch), 6, 10 and 12h post morning dose, if blood volume permits. When this results in multiple samples at the same time point, only one sample will be collected (e.g., 24hrs post-dose = pre-dose (time 0) for the next dose). Refer to the SPM for additional details on the collection and processing of PD samples.
11. Serial blood samples for the determination of the PK of GSK1292263 will be collected on Days 1, 7 and 14. Metformin and sitagliptin concentrations will also be measured using Day 14 samples. PK sampling times may be changed based on observed PK profile, but the total number of samples will not change. Blood samples for PK will be collected on Days 1 and 14, at immediately pre-morning dose, 1, 2, 4, 6, 8, 10, 11, 12, 14, 16, 18, 24 and 48 hrs post-morning dose. On Day 7, blood samples for PK will be collected at predose (=post- breakfast), 1, 2, 4 (= pre lunch), 6 and 10 (= immediately post-dinner, predose for BID regimen). Trough samples for PK will be collected early in the morning (fasting) on Days 4, 5, and 6. When planned PK sampling results in multiple samples at the same time point, only one sample will be collected (e.g., 24hrs post-dose = pre-dose (time 0) for the next dose). Refer to the Study Procedures Manual for details on the collection and processing of PK samples.

4.8 Time and Events Table (Part B) (Continued)

12. If appropriate, the PI has the option of adjusting prior medications when they are resumed. Review of laboratory results should occur, and doses of medications adjusted as appropriate.
13. At screening, medications taken in the 90 days prior should be collected.

5. STUDY POPULATION

5.1. Number of Subjects

Approximately 68 subjects may be enrolled (up to 8 subjects in Part A, 60 in Part B) and complete dosing and study assessments. Additional subjects/cohorts may be enrolled to allow for evaluation of additional dose levels, with notification of IRBs.

For Part B, 12 subjects will be randomized into each of 3 GSK1292263 treatment arms (BID doses 75mg and 300mg GSK1292263 and QD dose 600mg), the open-label sitagliptin arm and the placebo arm.

If subjects prematurely discontinue the study, additional subjects may be enrolled as replacement subjects and assigned to the same treatment (Part A), or treatment arm (Part B), at the discretion of the Sponsor, in consultation with the Investigator.

5.2. Eligibility Criteria

5.2.1. Inclusion Criteria

A subject will be eligible for inclusion in this study only if all of the following criteria apply:

1. Male or female subjects, 18 - 65 years of age, inclusive, at the time of signing the informed consent.
2. A female subject is eligible to participate if she is of non-childbearing potential, defined as pre-menopausal females with a documented tubal ligation or hysterectomy; or postmenopausal defined as 12 months of spontaneous amenorrhea. If the clinical situation is unclear, FSH and estradiol levels may be used to confirm post-menopausal status at Screening. Simultaneous follicle stimulating hormone (FSH) > 40 mIU/ml and estradiol < 40pg/ml (<140pmol/L) is confirmatory in the absence of a clear post-menopausal history.
3. Male subjects are willing to employ the contraceptive methods outlined in Section 8.1.1.
4. Except as noted elsewhere, subjects should have no significant known medical conditions other than T2DM that would affect the safety of the subject or the objectives of the study. This will be determined by a responsible physician, based on a medical evaluation including medical history, physical examination, laboratory tests and ECGs. Subjects with treated and stable hypertension and dyslipidemia are eligible if they fulfill the entry criteria for the study (see exclusion criteria #1 and 5). A subject with a clinical abnormality or laboratory parameters that meets inclusion criteria, but is outside the reference range for the population being studied may be included only if the Investigator and the GSK Medical Monitor agree that the finding is unlikely to introduce additional risk factors and will not interfere with the study procedures.
5. BMI (body mass index) within the range 21.8-37.5 kg/m^2, inclusive.

6. Parts A and B: T2DM diagnosed by American Diabetes Association criteria for at least 3 month prior to screening:
   - If currently on stable metformin monotherapy (≥ 1000mg/d for at least 1 month).
   - Fasting plasma glucose (FPG) level ≤ 250mg/dL at the Screening visit.
   - FPG or fasting blood glucose level ≤ 250mg/dL on Day -1 (Part A) or Day -2 (Part B).
   - HbA1c between 6.5 and 11.0%, inclusive, at screening visit.

   For subjects that are being screened for Part B, the anti-diabetic management must not have been altered by introduction of metformin within 3 months prior to screening.

7. Capable of giving written informed consent, which includes compliance with the requirements and restrictions listed in the consent form.
8. Average QTcB or QTcF < 450 msec; or QTc < 480 msec in subjects with right bundle branch block. Subjects with left bundle branch block are not eligible.
9. AST and ALT < 2xULN; alkaline phosphatase and bilirubin ≤ 1.5xULN (isolated bilirubin >1.5xULN is acceptable if bilirubin is fractionated and direct bilirubin <35%). Subjects with Gilbert's syndrome are allowed to participate in the study.

5.2.2. Exclusion Criteria

A subject will not be eligible for inclusion in this study if any of the following criteria apply:

1. Has any of the following laboratory abnormalities:
   - Positive pre-study Hepatitis B surface antigen or positive Hepatitis C, result within 3 months of screening.
   - Positive test for HIV antibody.
   - History of uncorrected thyroid dysfunction or an abnormal thyroid function test assessed by TSH at Screening. (NOTE: subjects with hypothyroidism on a stable dose of thyroid replacement therapy for at least 3 months prior to Screening and who have a screening thyroid stimulating hormone (TSH) within the normal range may participate.).
   - History of ketoacidosis or lactic acidosis.
   - Fasting triglycerides > 450mg/dL at screening.
   - For females a hemoglobin < 11.5g/dL, and for males a hemoglobin < 12.5g/dL. (A female subject with hemoglobin between 10g/dL and 11.5 g/dL, or a male subject with hemoglobin between 10g/dL and 12.5g/dL may be enrolled only if the Investigator and the GSK Medical Monitor agree that the finding is unlikely to introduce additional risk to the subject and will not interfere with the study procedures).

- A positive pre-study drug/urine screen. A minimum list of drugs that will be screened for include amphetamines, barbiturates, cocaine, opiates, cannabinoids and benzodiazepines.
- A pre-study urine cotinine screen indicating use of tobacco/ nicotine containing products.

2. If female is pregnant or has a positive pregnancy test or is lactating.
3. Significant renal disease as manifested by one or more of the following:
   - Glomerular filtration rate (or creatinine clearance) <60mL/min. (estimated from serum creatinine (SCr) and demographic data using the MDRD calculation):
     - To calculate estimated GFR (mL/min/1.73m^2) manually:

       = 186 x (SCr in mg/dL)$^{-1.154}$ x (age)$^{-0.203}$ x (0.742 if female) x (1.210 if African-American)

       = exp (5.228-1.154 x ln (SCr)-0.203x ln(age)-(0.299 if female) + (0.192 if African American))

     (A validated MDRD calculator is on the internet at www.mdrd.com.)

   - Urine protein/creatinine (mg of protein/mg of creatinine) ratio >2.5; **<u>or</u>** urine albumin or protein concentration >300mg/g of creatinine.
   - Known loss of a kidney either by surgical ablation, injury, or disease.
4. Significant ECG abnormalities, defined as follows:

Heart Rate	< 50 and >100bpm
PR Interval	<120 and > 220ms
QRS duration	< 70 and >120ms
QT_C Interval (Bazett)*	> 450ms

Or, has clinically significant rhythm abnormalities identified during 24-hour Screening Holter assessment. Subjects with left bundle branch block are excluded from the study. Subjects with partial right bundle branch block may be considered for inclusion following consultation with the GSK Medical Monitor. Subjects with Wolf-Parkinson-White (WPW) syndrome are excluded from the study.

*Note that if ECG abnormalities are identified, the ECG should be repeated two more times (with 5 minutes between ECG readings) and the average of the 3 values used to determine eligibility.

5. Systolic blood pressure > 150mmHg or <80mmHg or diastolic blood pressure > 95mmHg or <60mmHg at screening. Blood pressure assessments may be repeated once if needed, allowing adequate time for subject to rest.
6. Previous use of insulin as a treatment within 3 months of screening, or for >2 weeks when used for acute illness in the last 12 months prior to screening, or if used for more than 1 year when associated with gestational diabetes mellitus.

7. Has a history of any of the following conditions:
   - Clinically significant symptoms of gastroparesis.
   - Symptomatic cholelithiasis or obstructive or inflammatory gallbladder disease within 3 months prior to screening.
   - Gastrointestinal disease that could affect fat or bile acid absorption, or the pharmacokinetics or pharmacodynamics of the study drugs, including inflammatory bowel disease, chronic diarrhea, Crohn's or malabsorption syndromes within the past year.
   - Gastrointestinal surgery that may affect the pharmacokinetics or pharmacodynamics of the study drugs.

     Note: Subjects may be enrolled in the study if they have had a cholecystectomy three or more months before the time of screening and are stable and asymptomatic.
   - Chronic or acute pancreatitis.
8. History of regular alcohol consumption within 6 months of the study defined as:
   - An average weekly intake of >14 drinks for males or >7 drinks for females. One drink is equivalent to 12 g of alcohol: 12 ounces (360mL) of beer, 5 ounces (150mL) of wine or 1.5 ounces (45mL) of 80 proof distilled spirits.
9. Urinary cotinine levels indicative of smoking or history or regular use of tobacco- or nicotine-containing products within 6 months prior to screening. If quantification of cotinine is available, this may be utilized to distinguish 'active' from 'passive' inhalation of tobacco smoke.
10. Has participated in a clinical trial and has received a drug or a new chemical entity within 30 days or 5 half-lives, or twice the duration of the biological effect of any drug (whichever is longer) prior to the first dose of current study medication.
11. Exposure to more than four new chemical entities within 12 months prior to the first dosing day.
12. Is taking prohibited medications. See Section 9.3 for a detailed list of prohibited medications. A list of permitted medications is provided in Section 9.1. Note also:
    - The use of non-metformin anti-diabetic agents. In Parts A and B, subjects will not be allowed to wash-off of unapproved anti-diabetic medications in order to qualify for participation in this study.
    - Subjects must wash out from the following medications during the 7-day period prior to first dose, and must remain off these medications through discharge on Day 2 (Part A) or Day 15 (Part B): all statin agents, fat absorption blocking agents, bile acid sequestrants. Fibrates must be washed out for a 14-day period prior to first dose.

- Use of prescription or non-prescription drugs, including vitamins, herbal and dietary supplements (including St John's Wort) within 7 days (or 14 days if the drug is a potential enzyme inducer) or 5 half-lives (whichever is longer) prior to the first dose of study medication, unless in the opinion of the Investigator and GSK Medical Monitor the medication will not interfere with the study procedures or compromise subject safety.

13. Unwilling to abstain from
    - Caffeine-or xanthine-containing products from Day -7 until D2 (Part A) or Day -7 through Day 15 (Part B).
    - Use of illicit drugs or nicotine-containing products.
    - Alcohol from Day -7 prior to dosing until D2 (Part A) or Day -7 through Day 15 (Part B).
    - Consumption of red wine, Seville oranges, grapefruit or grapefruit juice from 7 days prior to the first dose of study medication until collection of the final pharmacokinetic blood samples.
14. History of sensitivity to any of the study medications, or components thereof, or a history of drug or other allergy that, in the opinion of the physician responsible, contraindicates their participation. This includes sensitivity to heparin or heparin-induced thrombocytopenia, if heparin will be used to maintain catheter patency.
15. Where participation in the study would result in donation of blood in excess of approximately 500mL within a 56 day period.
16. Subject is either an immediate family member of a participating investigator, study coordinator, employee of an investigator; or is a member of the staff conducting the study.
17. Unwillingness or inability to follow the procedures outlined in the protocol.
18. Subject is mentally or legally incapacitated.

5.2.3. Other Eligibility Criteria Considerations

To assess any potential impact on subject eligibility with regard to safety, the investigator must refer to the following document(s) for detailed information regarding warnings, precautions, contraindications, adverse events, and other significant data pertaining to the investigational product(s) being used in this study: Investigator's Brochure for GSK1292263 and supplements 1 and 2 [GlaxoSmithKline Document Number RM2008/00434/00; GlaxoSmithKline Document Number RM2009/00168/00; GlaxoSmithKline Document Number RM2009/00168/01] , the metformin package insert (for example [**GLUCOPHAGE** Package Insert, 2009]), and sitagliptin prescribing Information [**JANUVIA** Package Insert, 2009; **JANUMET** Package Insert, 2009] .

6. DATA ANALYSIS AND STATISTICAL CONSIDERATIONS

6.1. Hypotheses and Treatment Comparisons

The focus of this study is to evaluate safety and tolerability of GSK1292263 when co-administered with metformin, and to estimate GSK1292263 PK parameters and PD effects in subjects with T2DM taking metformin following single doses and repeat doses of GSK1292263. No formal statistical hypotheses will be tested. Descriptive statistics will be used to assess safety and tolerability objectives. An estimation approach will be used to address the PK and PD study objectives, where point estimates and corresponding confidence intervals will be constructed.

To assess the safety and tolerability of GSK1292263, adverse events and changes in ECGs, vital signs and laboratory values will be evaluated. Treatment comparisons with placebo and sitagliptin will be based on review of descriptive statistics.

In Part B point estimates and 90% confidence intervals for the slope for ln(dose) from the analysis of GSK1292263 PK parameters [AUC(0-10) (BID regimens only), AUC(0-24), AUC(0-∞) (as data permit), and Cmax following single dosing on Day 1 and AUC(0-10) (BID regimens only), AUC(0-24), and Cmax following repeat dosing] will be calculated to assess dose-proportionality. Dose proportionality will also be assessed by comparing the PK parameters for each of the test doses to those for the reference dose and by visual inspection (test and reference doses to be determined). Point estimates and 90% confidence intervals will be presented for each of the differences (test-reference).

Repeat-dose AUC(0-10h) (BID regimens only), AUC(0-24h) and Cmax on Days 1 and 14 will be compared to assess accumulation (as data permit). Point estimates and 90% confidence intervals will be presented for each of the comparisons.

Point estimates and 90% confidence intervals for the slope for day from the analysis of GSK1292263 trough concentration ($C\tau$) will be calculated to assess achievement of steady state at each dose level.

PK data from Part A will be compared to data obtained in the on-going study in T2DM, GPR111598.

Fasted and derived PD parameters will be compared between each of the following during the repeat dose part of the study (Part B):

- GSK1292263 Day 14 versus Day -1
- GSK1292263 Day 14 versus placebo Day 14 (change from baseline)
- Sitagliptin Day 14 versus placebo Day 14 (change from baseline)
- GSK1292263 Day 14 versus sitagliptin Day 14 (change from baseline)
- GSK1292263 Day 7 versus placebo Day 7 (change from baseline)
- Sitagliptin Day 7 versus placebo Day 7 (change from baseline)

No adjustments for multiple comparisons will be made.

6.2. Sample Size Considerations

6.2.1. Sample Size Assumptions

The sample size for all parts of this study is based primarily on feasibility considerations; however, for the repeat dose part, as a reference, provided below is an estimate of how the targeted sample size affects the precision of the estimation and the power of detecting a clinically relevant difference between active treatment and placebo for the primary PD endpoints.

In Part B, this study is targeted to have 12 subjects complete each 'active' treatment group and 12 subjects complete the placebo treatment. Based on Period 1 data from [Brazg, 2007] estimates of the change from baseline (CFB) fasting plasma glucose and glucose weighted mean AUC(0-24) standard deviations on Day 14 for the sitagliptin+metformin treatment were 13.5 and 21.9mg/dL, respectively. Based on the sample size and variability estimates above, expected half-widths of the 95% CI for the mean difference between active treatment and placebo and the difference detectable with 80% or 90% power are provided in Table 12.

Table 12 Sample Size Assumptions

Parameter (mg/dL)	Common SD	Sample Size		Expected Half-Width of 95% CI	Difference detectable with power[1]	
		Active	Placebo		90%	80%
CFB FPG	13.5	12	12	11.4	16.7	14.1
CFB Glucose Wt. Mean AUC(0-24)	21.9	12	12	18.5	20.8	17.7

1. Based on a one-sided two-sample t-test at significance level (α) of 0.05 (unadjusted for multiple comparisons).

6.2.2. Sample Size Sensitivity

Based on the assumption of a 25% increase in the standard deviation of CFB FPG and glucose weighted mean AUC(0-24) on Day 14 for the sitagliptin+metformin treatment, the expected half-widths of the 95% CI for the mean difference between active treatment and placebo and the difference detectable with 80% or 90% power are provided in Table 13.

Table 13 Sample Size Sensitivity

Parameter (mg/dL)	Common SD	Sample Size		Expected Half-Width of 95% CI	Difference detectable with power[1]	
		Active	Placebo		90%	80%
CFB FPG	16.9	12	12	14.3	27.0	22.9
CFB Glucose Wt. Mean AUC(0-24)	27.4	12	12	23.2	33.8	28.7

1. Based on a one-sided two-sample t-test at significance level (α) of 0.05 (unadjusted for multiple comparisons).

6.2.3. Sample Size Re-estimation

No sample size re-estimation will be performed.

6.3. Data Analysis Considerations

6.3.1. Interim Analysis

There will be no formal statistical interim analysis. However, reviews of safety, tolerability, pharmacokinetic, and pharmacodynamic data may be performed by the GSK study team during the study. This analysis can include review of individual subject data and basic summaries and graphs. The GSK study team will have access to an unblinded copy of the randomization schedule. Subjects and all site personnel, with the exception of the study pharmacists, will be blinded to subject randomization throughout the trial.

The preliminary results from available safety data may be reported for the purposes of safety review by GSK, the study investigators, and where required by regulatory bodies prior to database freeze.

6.3.2. Final Analyses

The final planned analyses will be performed after all subjects have completed each part of the study after database freeze/unblinding. Version 9.1 or higher of the SAS system will be used to analyze the data as well as to generate tables, figures, and listings. Complete details will be documented in the Reporting and Analysis Plan (RAP).

6.3.2.1. Safety Analyses

Safety data will be presented in tabular and/or graphical format and summarized descriptively according to GSK's Integrated Data Standards Library (IDSL) standards.

The GSK Division of Discovery Biometrics will conduct summary analyses of safety assessments. No formal statistical comparisons will be made for the safety data. Details of the safety analyses will be provided in the RAP.

6.3.2.2. Pharmacokinetic Analyses

Pharmacokinetic analysis will be the responsibility of the Clinical Pharmacology Modeling and Simulation Department, GlaxoSmithKline. Plasma GSK1292263 and sitagliptin concentration-time data will be analyzed by non-compartmental methods with WinNonlin Professional Version 5.2 or higher. Calculations will be based on the actual sampling times recorded during the study. From the plasma concentration-time data, the following pharmacokinetic parameters will be determined, as data permit.

Following single dosing in Part A:

- Maximum observed plasma concentration (Cmax), time to Cmax (tmax), the time prior to first quantifiable plasma concentration (tlag), area under the plasma concentration-time curve [AUC(0-24h), and AUC(0-∞)], apparent terminal phase half-life (t½), and, as data permit, the apparent clearance CL/F, and the apparent volume of distribution V/F.

Following repeat dosing in Part B:

- Cmax, tmax, tlag (Day 1 only), area under the plasma concentration-time curve over the dosing interval [AUC(0-10) (BID regimens only), AUC(0-24)] and AUC(0-10) on Day 7 (all regimens), pre-dose (trough) concentration at the end of the dosing interval (Cτ) (Days 4, 5, 6 and 7), and t½ (Day 14 only) will be estimated for GSK1292263, as data permit.
- Cmax, tmax, and area under the plasma concentration-time curve over the dosing interval [AUC(0-24)] on Day 14, will be estimated for sitagliptin.
- AUC(0-24h), AUC(0-10h) (BID regimens only), and Cmax following dosing on Days 1 and 14 will be used for assessment of dose proportionality of GSK1292263. Trough concentration (Cτ) samples collected on Days 4, 5, 6 and 7 will be used to assess attainment of steady state for GSK1292263. To estimate the extent of accumulation after repeat dosing, the observed accumulation ratio (Ro) will be determined.

Additional or different PK parameters may be calculated if necessary based on observed data.

Pharmacokinetic data will be presented in graphical and/or tabular form and will be summarized descriptively. Descriptive statistics (n, arithmetic mean, standard deviation, 95% CI, minimum, median, and maximum) will be calculated for all pharmacokinetic parameters by treatment and day. For $\log_e$-transformed variables geometric mean, 95% confidence interval and %CVb $(100 * \sqrt{(\exp(SD^2) - 1)})$ will be provided, where the SD is the standard deviation of log-transformed data. All pharmacokinetic data will be stored in the Archives, GlaxoSmithKline Pharmaceuticals, R&D.

Statistical analyses of the pharmacokinetic parameter data will be the responsibility of Discovery Biometrics, GlaxoSmithKline. Dose proportionality, accumulation, time-invariance, and achievement of steady state will be analyzed by using appropriate power, ANOVA, or linear regression models. Details of the statistical analysis of PK data will be provided in the RAP.

No formal analyses of sitagliptin pharmacokinetic data will be performed. Details will be provided in the RAP.

6.3.2.3. Pharmacokinetic/Pharmacodynamic Analyses

The relationship between the plasma concentrations of GSK1292263 and sitagliptin or the corresponding metrics of systemic exposure (Cmax and AUC) and relevant PD, safety, and tolerability endpoints will be explored using appropriate PK/PD modelling techniques, as data permit.

6.3.2.4. Pharmacodynamic Analyses

Analyses of pharmacodynamic data will be the responsibility of Discovery Biometrics, GlaxoSmithKline. PD data will be presented in graphical and/or tabular form and will be summarized descriptively.

PD parameters, including fasting PD, maximum PD, incremental and weighted mean AUCs, along with the change from baseline values will be derived and summarized.

In Part B, an analysis of covariance (ANCOVA) with a fixed effect terms for treatment will be fitted with the post-baseline AUC minus baseline (Day -1 AUC) as the dependent variable and the Day -1 AUC as a covariate. Pairwise differences in least squares means between each GSK1292263 active treatment and placebo will be calculated, and 95% confidence intervals will be constructed for these differences.

Subgroup analysis of PD data may be performed by separately analyzing baseline BMI and other subject characteristics.

Details of the statistical analysis of PD data will be provided in the RAP.

6.3.2.5. Novel Biomarker(s) Analyses

The results of any novel biomarker investigations, if performed, will be reported separately from the main clinical study report. All endpoints of interest from all comparisons will be descriptively and/or graphically summarized as appropriate to the data.

Additional exploratory analyses may be performed to further characterize the novel biomarker(s).

6.3.2.6. Hunger, Craving, and Fullness Questionnaire and Calorie Counts

The HCFQ data will be listed and summarized with frequency counts at each time point by treatment. Calorie count data will be summarized at each time point. Additional details will be provided in the RAP.

7. STUDY ASSESSMENTS AND PROCEDURES

This section lists the parameters of each planned study assessment. The exact timing of each assessment is listed in the Time and Events Tables (Section 4.7 and Section 4.8). Detailed procedures for obtaining each assessment are provided in the Study Procedures Manual (SPM). Whenever vitals signs, 12-lead ECGs and blood draws are scheduled for the same nominal time, the assessments should occur in the following order: 12-lead ECG, vital signs, blood draws. When procedures are close to dosing and meals, the following order will be employed, as appropriate: ECGs, vitals, blood sampling, meal ingestion, dosing. The timing of the assessments should allow the blood draws to occur at the exact nominal time.

The timing and number of planned study assessments, including safety, pharmacokinetic, pharmacodynamic/biomarker or other assessments may be altered during the course of the study based on newly available data (e.g., to obtain data closer to the time of peak plasma concentrations) to ensure appropriate monitoring. The change in timing or addition of time points for any planned study assessments must be approved and documented by GSK, but this will not constitute a protocol amendment. The IRB will be informed of any safety issues that require alteration of the safety monitoring scheme. No more than approximately 500mL of blood will be collected over a 30-day period in the study, including any extra assessments that may be required.

7.1. Demographic/Medical History Assessments

The following demographic parameters will be captured: date of birth, gender, race and ethnicity.

Medical/medication/alcohol history will be assessed as related to the eligibility criteria listed in Section 5.2.

7.2. Safety

Planned timepoints for all safety assessments are listed in the Time and Events Tables (Section 4.7 and Section 4.8). Additional time points for safety tests such as vital signs, physical exams and laboratory safety tests may be added during the course of the study based on newly available data to ensure appropriate safety monitoring.

7.2.1. Physical Exams

- A complete physical examination will include assessments of the head, eyes, ears, nose, throat, skin, thyroid, neurological, lungs, cardiovascular, abdomen (liver and spleen), lymph nodes and extremities. Height, weight, waist circumference will also be measured and recorded.
- A brief physical examination will include assessments of the skin, lungs, cardiovascular system, and abdomen (liver and spleen).

7.2.2. Vital Signs

- Vital sign measurements will include systolic and diastolic blood pressure and pulse rate.
- At each time point, assessment should be performed after resting in a supine or semi-supine position for at least 10 minutes.

7.2.3. Electrocardiogram (ECG)

- 12-lead ECGs will be obtained in a supine position at each timepoint noted in the Time and Events Tables (Section 4.7 and Section 4.8) during the study using an ECG machine that automatically calculates the heart rate and measures PR, QRS, QT, and QTc intervals. Refer to Section 4.6.2 for QTc withdrawal criteria and additional QTc readings that may be necessary.
- Continuous cardiac telemetry will be performed in Part A as noted in the Time and Events Table (Section 4.7). Full disclosures will be maintained as part of the subject's source documents and will be reviewed in detail.

7.2.4. Clinical Laboratory Assessments

Hematology, clinical chemistry, urinalysis and additional parameters to be tested are listed below:

Hematology

Platelet Count	*RBC Indices*:	*Automated WBC Differential:*
RBC Count	MCV	Neutrophils
WBC Count (absolute)	MCH	Lymphocytes
Reticulocyte Count	MCHC	Monocytes
Hemoglobin		Eosinophils
Hematocrit		Basophils

Clinical Chemistry

BUN	Potassium	AST (SGOT)	Total and direct bilirubin
Creatinine	Chloride	ALT (SGPT)	Uric Acid
Glucose, fasting	Total CO_2	GGT	Albumin
Sodium	Calcium	Alkaline phosphatase	Total Protein
Magnesium	Triglycerides	Total Cholesterol	LDL cholesterol
Phosphorus	FFA (NEFA)	HDL cholesterol	
Apolipoprotein A1	Apolipoprotein B	Apolipoproteiin E (if test is available from local lab)	C-Reactive Protein

Routine Urinalysis

Specific gravity
Urine protein or albumin (quantitative analysis)
pH, glucose, protein, blood and ketones by dipstick
Microscopic examination (if blood or protein is abnormal)

Other tests

HbA1c (screening)
HIV
Hepatitis B (HBsAg)
Hepatitis C (Hep C antibody -- if second generation Hepatitis C antibody positive, a hepatitis C antibody Chiron RIBA immunoblot assay should be reflexively performed <u>on the same sample</u> to confirm the result)
FSH and estradiol (as needed in women of non-child bearing potential only)
Alcohol and drug screen (to include at minimum: amphetamines, barbiturates, cocaine, opiates, cannabinoids and benzodiazepines).
Urine cotinine

7.3. Pregnancy

7.3.1. Time period for collecting pregnancy information

All female participants in the study must be of non-child bearing potential, with post-menopausal status confirmed at screening by FSH and estradiol testing. A pregnancy test will be performed on Day -1 and at the follow-up visit.

All pregnancies in female subjects will be collected after the start of dosing and until the follow-up visit.

7.3.2. Action to be taken if pregnancy occurs

The investigator will collect pregnancy information on any female subject, who becomes pregnant while participating in this study. The investigator will record pregnancy information on the appropriate form and submit it to GSK within 2 weeks of learning of a subject's pregnancy. The subject will also be followed to determine the outcome of the pregnancy. Information on the status of the mother and child will be forwarded to GSK. Generally, follow-up will be no longer than 6 to 8 weeks following the estimated delivery date. Any premature termination of the pregnancy will be reported.

While pregnancy itself is not considered to be an AE or SAE, any pregnancy complication or elective termination of a pregnancy for medical reasons will be recorded as an AE or SAE.

A spontaneous abortion is always considered to be an SAE and will be reported as such. Furthermore, any SAE occurring as a result of a post-study pregnancy and is considered reasonably related to the investigational product by the investigator, will be reported to GSK as described in Section 12. While the investigator is not obligated to actively seek this information in former study participants, he or she may learn of an SAE through spontaneous reporting.

Any female subject who becomes pregnant while participating will be withdrawn from the study.

7.3.3. Action to be taken if pregnancy occurs in a female partner of a male study subject

The investigator will attempt to collect pregnancy information on any female partner of a male study subject who becomes pregnant while participating in this study. The investigator will record pregnancy information on the appropriate form and submit it to GSK within 2 weeks of learning of the partner's pregnancy. The partner will also be followed to determine the outcome of the pregnancy. Information on the status of the mother and child will be forwarded to GSK. Generally, follow-up will be no longer than 6 to 8 weeks following the estimated delivery date. Any premature termination of the pregnancy will be reported.

7.4. Pharmacokinetics

7.4.1. Blood Sample Collection

Blood samples for pharmacokinetic analysis of GSK1292263, sitagliptin (Part B only) and metformin will be collected at the time points indicated in Section 4.7 and Section 4.8, Time and Events Tables. The actual date and time of each blood sample collection will be recorded. The timing of PK samples may be altered and/or PK samples may be obtained at additional time points to ensure thorough PK monitoring.

Details of PK blood sample collection (including volume to be collected), processing, storage and shipping procedures are provided in the Study Procedures Manual (SPM).

7.4.2. Sample Analysis

Plasma/serum analysis will be performed under the management of Worldwide Bioanalysis, DMPK, GlaxoSmithKline. Concentrations of GSK1292263, sitagliptin (Part B only) and metformin will be determined in plasma samples using the currently approved analytical methodology. Raw data will be stored in the GLP Archives, GlaxoSmithKline. Once the plasma has been analyzed for GSK1292263, any remaining plasma may be analyzed qualitatively for other circulating metabolites and the results reported under a separate DMPK protocol.

7.5. Biomarker(s)/Pharmacodynamic Markers

7.5.1. Type II Diabetes Biomarkers/Pharmacodynamic Markers

Blood samples for assessment of 24-hour glucose, insulin, C-peptide, glucagon, GLP-1 (total and active), total GIP, total PYY and glycerol will be collected at the timepoints indicated in the Time and Events Tables in Section 4.7 and Section 4.8. The timing of the collections may be adjusted on the basis of emerging PK or PD data from this study or other new information in order to ensure optimal evaluation of the PD endpoints.

7.5.2. Exploratory Biomarkers

Blood sample(s) will be collected during this study as indicated in Section 4.2.7.3 and Section 4.2.7.4, and may be used for the purposes of measuring exploratory biomarkers (e.g., adiponectin, leptin, ghrelin, amylin, PP, CCK) or novel biomarkers to identify factors that may influence diabetes, and/or medically related conditions, as well as the biological and clinical responses to GSK1292263, including adverse events, if relevant.

Exploratory biomarkers, if analyzed, will be assessed from the PD blood samples.

7.6. Pharmacogenetics

Information regarding pharmacogenetic (PGx) research is included in Appendix 2: Pharmacogenetic research. The IRB and, where required, the applicable regulatory agency must approve the PGx assessments before these can be conducted at the site. In some cases, approval of the PGx assessments can occur after approval is obtained for the rest of the study. If so, then the written approval will clearly indicate approval of the PGx assessments is being deferred and in most cases, the study, except for PGx assessments, can be initiated. When PGx assessments are not approved, the approval for the rest of the study will clearly indicate this and therefore, PGx assessments will not be conducted.

8. LIFESTYLE AND/OR DIETARY RESTRICTIONS

8.1. Contraception Requirements

8.1.1. Male Subjects

To prevent pregnancy in a female partner or to prevent exposure of any partner to the investigational product from a male subject’s semen, male subjects must use one of the following contraceptive methods:

- Abstinence, defined as sexual inactivity consistent with the preferred and usual lifestyle of the subject. Periodic abstinence (e.g., calendar, ovulation, symptom-thermal, post-ovulation methods) and withdrawal are not acceptable methods of contraception.
- Condom *(during non-vaginal intercourse with any partner - male or female)* **OR**
- Condom and occlusive cap (diaphragm or cervical/vault caps) plus spermicidal agent (foam/gel/film/cream/suppository) *(during sexual intercourse with a female).*

8.2. Meals and Dietary Restrictions

Category	Dietary Guidelines and Restrictions
Exercise	Subjects will abstain from strenuous exercise for 48 hours prior to each blood collection for clinical laboratory tests.<br>Light recreational activity is permitted while subjects are in the clinic.<br>Subjects will be given instructions for exercise when not in the unit.
Fasting	Subjects should fast from all food or drink with the exception of water from midnight prior to each blood collection for clinical chemistry tests.<br>Subjects should fast from all food or drink with the exception of water from midnight prior to each morning administration of study drug. See the SPM for timing of meals in relation to dosing for each part of the study.
Water	Water is permitted *ad libitum*.
Breakfast	A standardized breakfast will be administered on Day 1 (Part A), and dosing will occur after this meal is completely eaten. In Part B, on Days -1, 7 and 14 standardized breakfast meals will be consumed. See SPM for menu details. The composition of the breakfast meal on D1 will be the same as that of D-1/7/14. The start and completion time of the meal will be recorded on Days -1, 1, 7 and 14.
Lunch	In Part A, a standardized lunch will be fed on Day 1. In Part B, a standardized lunch will be consumed on Days -1 through 14 at approximately 4h after the morning dosing. The exact same lunch will be provided on Days -1, 1, 7 and 14. See SPM for dosing in relation to meals and blood sampling. The start and completion time of the meal will be recorded on Days -1, 1, 7 and 14.
Evening meal	In Part A, a standardized evening meal will be fed on Day 1. In Part B, a standardized evening meal will be consumed on Days -1 through 14 at approximately 10h after the morning dose. The evening dose will be taken immediately after eating the meal. The same evening meal will be provided on Days -1, 1, 7 and 14. See SPM for dosing in relation to meals and blood sampling. The start and completion time of the meal will be recorded on Days -1, 1, 7 and 14.
Evening Snack	No evening snack will be permitted on Day 1 in Part A, and Days -1, 7 and 14 in Part B. On other days, an evening snack will be permitted at approximately 12 - 14 hours after dosing and up to 23:00.
Alcohol, Caffeine, Xanthine	Subjects will not be allowed to consume alcohol or caffeine- or xanthine-containing products (e.g., coffee, tea, cola drinks, chocolate) from 7 days prior to dosing in Parts A and B, until final discharge from the clinic (Day 2 in Part A and Day 15 in Part B).
Nicotine	Subjects will not be allowed to smoke or use nicotine-containing products during the course of the study (from screening until after the final follow-up visit)
Grapefruit, Red Wine, Seville Oranges	Subjects will not be allowed to consume red wine, Seville oranges, grapefruit juice or grapefruit within 7 days prior to the first dose of study medication until final discharge from the clinic (Day 2 in Part A and Day 15 in Part B).

9. CONCOMITANT MEDICATIONS AND NON-DRUG THERAPIES

9.1. Permitted Medications

All concomitant medications taken during the study will be recorded in the CRF. Subjects using the following medications must be on stable doses during the 3 months prior to Screening:

- Antihypertensives (e.g., beta blockers, ACE inhibitors, angiotensin II receptor antagonists, calcium channel blockers and thiazide diuretics), with the exception of diltiazem, verapamil, and other CYP3A4 inhibitors as noted below. The dosage(s) of antihypertensive medications should, if medically appropriate, remain unchanged while the subject is enrolled in the study. If a dosage change is necessary, information pertaining to the change must be documented on the concomitant medication pages in the eCRF.
- Thyroid hormone.

The use of non-steroidal anti-inflammatory drugs (NSAIDs), especially aspirin, is allowed only if prescribed by a physician on a regular schedule for cardiovascular prophylaxis or chronic pain control. Intermittent/ PRN use of NSAIDs is prohibited.

Acetaminophen may be used on an as-needed basis provided the total daily dose does not exceed 2g.

Permitted concomitant medications should be taken as prescribed and the usual time(s), and may be taken with water prior to scheduled study clinic visits without being considered to have broken their fast.

Other medications may be permitted following consultation with the GSK Medical Monitor if they are not considered to affect subject safety or the objectives of the study.

9.1.1. Anti-Viral Therapies for Influenza

Tamiflu and RELENZA™ are permitted for the treatment of influenza, following consultation with the GSK Medical Monitor. GSK will not supply flu treatment or vaccines.

9.2. Medications Permitted at Screening but Requiring Washout before Enrollment

The following medications require washout from Day -7 to final discharge from the unit of the study. Discontinuation and resumption of these medications should be documented in the eCRF.

- Statins and lipid-lowering drugs, including but not limited to the following single or combination products: atorvastatin, simvastatin, lovastatin, pravastatin, ezetimide, or simvastain / ezetimide combination
- Fat absorption blocking agents

- Bile acid sequestrants

Fibrates must be washed out from Day -14 to final discharge from the unit.

Other prior medications may be permitted that require washout following consultation with the GSK Medical Monitor if they are not considered to affect subject safety of the objectives of the study.

9.3. Prohibited Medications

- The use of anti-diabetic agents (other than metformin) is reason for exclusion and subjects will not be allowed to wash off of unapproved anti-diabetic medications in order to qualify for participation in this study. Insulin use is prohibited, unless it is required to control severe hyperglycemia during the study.
- Potent inhibitors of CYP3A4, including but not limited to diltiazem, verapamil, ketoconazole, cyclosporine.
- Potent inducers of CYP3A4, including but not limited to rifampicin, carbamazepine, phenobarbital, phenytoin.
- Cimetidine and other cationic drugs.
- Loop diuretics (e.g., bumetanide, torsemide, furosemide/ frusemide).
- Oral anticoagulants, including warfarin (note that aspirin and non-steroidal anti-inflammatory drugs are permitted as noted above).
- Oral or injectable corticosteroids (inhaled, intranasal and topical are permitted).
- Antiretroviral drugs.
- Methotrexate, cyclosporine or monoclonal antibodies (e.g., infliximab, adalimumab, etanercept, certolizimab pegol, rituximab) for autoimmune disease, rheumatoid arthritis or psoriasis.
- Atypical antipsychotic medications (e.g., aripiprazole, risperidone, clozapine, olanzapine, quetiapine, and ziprasidone).
- Monoamine oxidase inhibitors, selective serotonin reuptake inhibitors (SSRIs) or tricyclic antidepressants taken within the previous three months.
- Use of other drugs that may affect glucose and/or lipid metabolism such as danazol and niacin, are prohibited within 3 months prior to Day -7 and/or during the study.

9.4. Non-Drug Therapies

- Vitamins, herbal and dietary supplements (including St John’s Wort) are prohibited within 7 days or 5 half-lives (whichever is longer) prior to the first dose of study medication and through final discharge from the unit.

10. COMPLETION OR EARLY WITHDRAWAL OF SUBJECTS

10.1. Subject Completion

A completed subject is one who has completed all phases of the study including the follow-up visit.

The end of the study is defined as the last subject's last visit.

10.2. Subject Withdrawal Criteria

Refer to Section 4.5 and Section 4.6 for dose adjustment/stopping criteria based on safety/PK/PD criteria

A subject may withdraw from investigational product at any time at his/her own request, or may be withdrawn at any time at the discretion of the investigator for safety, behavioral or administrative reasons.

10.3. Subject Withdrawal Procedures

10.3.1. Subject Withdrawal from Study

Subjects may withdraw from the study at any time and for any reason. They are not obliged to state the reason for withdrawal. However, the reasons for withdrawal, or failure to provide a reason, must be documented by the physician on the eCRF. Every effort should be made by the physician to follow up subjects who withdraw from the study, by adhering to follow-up procedures specified in Section 4.7 and Section 4.8.

10.3.2. Subject Withdrawal from Investigational Product

If a subject does not receive all doses of randomized study drug planned for that subject, the subject will be considered to have prematurely discontinued study drug. Every effort should be made by the physician to follow up subjects who withdraw from the study, by adhering to follow-up procedures specified in Section 4.7 and Section 4.8.

Decisions regarding replacement of subjects prematurely discontinued from study drug will be made by the Investigator and GSK Medical Monitor on a case-by-case basis.

10.4. Treatment After the End of the Study

For subjects who washed out of medications, consistent instructions should be provided to the subjects defining how they should resume administration of these medications after the completion of final study procedures (Day 2 in Part A and Day 15 in Part B). The investigator may use his/her discretion with respect to modifying the doses of these medications when they are re-started.

Subjects will not receive any additional treatment after completion of the study because other treatment options are available.

10.5. Screen and Baseline Failures

Data for screen and baseline failures will be collected in source documentation at the site but will not be transmitted to GSK.

11. INVESTIGATIONAL PRODUCT(S)

Investigational product dosage and administration details are listed in Section 4.4.

11.1. Blinding

Subjects and all site personnel, with the exception of the study pharmacists, will be blinded to subject randomization throughout the trial.

Sitagliptin and metformin will be administered in this study in an unblinded manner.

Because the GSK study team will be assessing data on a real-time basis while the study is ongoing, the team will be unblinded during the trial to allow accurate correlation of safety and tolerability information with PK and PD values. For this reason, this study is characterized as a single-blind study.

In the case of a **medical emergency or in the event of a serious medical condition**, when knowledge of the investigational product is essential for the clinical management or welfare of the subject, **an investigator or other physician managing the subject may decide to unblind that subject's treatment code**. The investigator will make every effort to contact the GSK Medical Monitor or appropriate GSK study personnel before unblinding to discuss options. If the blind is broken for any reason and the investigator is unable to contact GSK prior to unblinding, the investigator must notify GSK **as soon as possible following** the unblinding incident **without revealing the subject's study treatment assignment,** unless the information is important to the safety of subjects remaining in the study. In addition, the investigator will record the date and reason for revealing the blinded treatment assignment for that subject in the appropriate data collection tool.

If a serious adverse event (SAE; as defined in Section 12.2 "Definition of an SAE") is reported to GSK, Global Clinical Safety and Pharmacovigilance (GCSP) staff may unblind the treatment assignment for the individual subject. If an expedited regulatory report to one or more regulatory agencies is required, the report will identify the subject's treatment assignment. When applicable, a copy of the regulatory report may be sent to investigators in accordance with relevant regulations, GSK policy, or both.

11.2. Packaging and Labeling

The contents of the label will be in accordance with all applicable regulatory requirements.

11.3. Preparation/Handling/Storage/Accountability

No special preparation of investigational product is required, including short-term protection from light.

Investigational product must be dispensed or administered according to procedures described herein. Only subjects enrolled in the study may receive investigational product. Only authorized site staff may supply or administer investigational product. All investigational products must be stored in a secure area with access limited to the investigator and authorized site staff. Investigational product is to be stored at up to 30°C. Maintenance of a temperature log (manual or automated) is required.

The investigator, institution, or the head of the medical institution (where applicable) is responsible for investigational product accountability, reconciliation, and record maintenance. The investigator or the head of the medical institution (where applicable), or designated site staff (e.g., storage manager, where applicable) must maintain investigational product accountability records throughout the course of the study. The responsible person(s) will document the amount of investigational product received from and returned to GSK and the amount supplied and/or administered to and/or returned by subjects. The required accountability unit for this study will be a bottle. Discrepancies are to be reconciled or resolved. Procedures for final disposition of unused investigational product are listed in the SPM.

Investigational product is not expected to pose significant occupational safety risk to site staff under normal conditions of use and administration. A Material Safety Data Sheet (MSDS)/equivalent document describing occupational hazards and recommended handling precautions either will be provided to the investigator, where this is required by local laws, or is available upon request from GSK.

11.4. Assessment of Compliance

When subjects are dosed at the study site, they will receive investigational products directly from the investigator or designee, under medical supervision. The date and time of each dose administered in the clinic will be recorded in the source documents. The dose of investigational product(s) and study participant identification will be confirmed at the time of dosing by a member of the study site staff other than the person administering the investigational product. Study site personnel will examine each subject's mouth to ensure that the investigational product was ingested.

11.5. Treatment of Investigational Product Overdose

The maximum total daily dose planned for this study is 600mg. For this study, any total daily dose of GSK1292263 greater than 600mg ingested in less than a 24 hour time period will be considered an overdose.

GSK does not recommend specific treatment for an overdose. The investigator will use clinical judgment to treat any overdose. Appropriate supportive treatment should be initiated according to subject's clinical signs and symptoms. Consideration should be given to fluid repletion and administration of exogenous glucose to maintain euglycemia if needed.

In the event of an overdose of sitagliptin, usual supportive measures are recommended (e.g., removal of unabsorbed materials from GI tract, employ clinical monitoring, including obtaining an ECG and initiation of supportive therapy as dictated by the subject's clinical needs).

12. ADVERSE EVENTS (AE) AND SERIOUS ADVERSE EVENTS (SAE)

The investigator or site staff is responsible for detecting, documenting and reporting events that meet the definition of an AE or SAE.

AEs will be collected from the start of the washout period and until the follow-up contact. AEs occurring within this period for Part A will be captured as Part A events, and those within this time frame for Part B will be captured as Part B events. Medical occurrences that begin prior to the start of investigational product but after obtaining informed consent may be recorded on the Medical History/Current Medical Conditions CRF.

SAEs will be collected over the same time period as stated above for AEs. However, any SAEs assessed as related to study participation (e.g. investigational product, protocol-mandated procedures, invasive tests, or change in existing therapy) or related to a GSK concomitant medication will be recorded from the time a subject consents to participate in the study up to and including any follow-up contact. All SAEs will be recorded and reported to GSK within 24 hours, as indicated in Section 12.2.

Investigators are not obligated to actively seek AEs or SAEs in former study participants. However, if the investigator learns of any SAE, including a death, at any time after a subject has been discharged from the study, and he/she considers the event reasonably related to the investigational product or study participation, the investigator would promptly notify GSK.

12.1. Definition of Adverse Events

An AE is any untoward medical occurrence in a patient or clinical investigation subject, temporally associated with the use of a medicinal product, whether or not considered related to the medicinal product.

Note: An AE can therefore be any unfavorable and unintended sign (including an abnormal laboratory finding), symptom, or disease (new or exacerbated) temporally associated with the use of a medicinal product.

Events meeting the definition of an AE **include**:

- Any abnormal laboratory test results (hematology, clinical chemistry, or urinalysis) or other safety assessments (e.g., ECGs, radiological scans, vital signs measurements), including those that worsen from baseline, and felt to be clinically significant in the medical and scientific judgement of the investigator.
- Exacerbation of a chronic or intermittent pre-existing condition including either an increase in frequency and/or intensity of the condition.
- New conditions detected or diagnosed after investigational product administration even though it may have been present prior to the start of the study.
- Signs, symptoms, or the clinical sequelae of a suspected interaction.
- Signs, symptoms, or the clinical sequelae of a suspected overdose of either investigational product or a concomitant medication (overdose per se will not be reported as an AE/SAE).

Events that **do not** meet the definition of an AE include:

- Any clinically significant abnormal laboratory findings or other abnormal safety assessments that are associated with the underlying disease, unless judged by the investigator to be more severe than expected for the subject's condition.
- The disease/disorder being studied or expected progression, signs, or symptoms of the disease/disorder being studied, unless more severe than expected for the subject's condition.
- Medical or surgical procedure (e.g., endoscopy, appendectomy); the condition that leads to the procedure is an AE.
- Situations where an untoward medical occurrence did not occur (social and/or convenience admission to a hospital).
- Anticipated day-to-day fluctuations of pre-existing disease(s) or condition(s) present or detected at the start of the study that do not worsen.
- The disease/disorder being studied, or expected progression, signs, or symptoms of the disease/disorder being studied, unless more severe than expected for the subject's condition

12.2. Definition of Serious Adverse Events

If an event is not an AE per Section 12.1, then it cannot be an SAE even if serious conditions are met (e.g., hospitalization for signs/symptoms of the disease under study, death due to progression of disease, etc).

An SAE is any untoward medical occurrence that, at any dose:

a. Results in death

b. Is life-threatening

NOTE: The term 'life-threatening' in the definition of 'serious' refers to an event in which the subject was at risk of death at the time of the event. It does not refer to an event, which hypothetically might have caused death, if it were more severe.

c. Requires hospitalization or prolongation of existing hospitalization

NOTE: In general, hospitalization signifies that the subject has been detained (usually involving at least an overnight stay) at the hospital or emergency ward for observation and/or treatment that would not have been appropriate in the physician's office or out-patient setting. Complications that occur during hospitalization are AEs. If a complication prolongs hospitalization or fulfills any other serious criteria, the event is serious. When in doubt as to whether "hospitalization" occurred or was necessary, the AE should be considered serious.

Hospitalization for elective treatment of a pre-existing condition that did not worsen from baseline is not considered an AE.

d. Results in disability/incapacity, or

NOTE: The term disability means a substantial disruption of a person's ability to conduct normal life functions. This definition is not intended to include experiences of relatively minor medical significance such as uncomplicated headache, nausea, vomiting, diarrhea, influenza, and accidental trauma (e.g. sprained ankle) which may interfere or prevent everyday life functions but do not constitute a substantial disruption.

e. Is a congenital anomaly/birth defect

f. Medical or scientific judgment should be exercised in deciding whether reporting is appropriate in other situations, such as important medical events that may not be immediately life-threatening or result in death or hospitalization but may jeopardize the subject or may require medical or surgical intervention to prevent one of the other outcomes listed in the above definition. These should also be considered serious. Examples of such events are invasive or malignant cancers, intensive treatment in an emergency room or at home for allergic bronchospasm, blood dyscrasias or convulsions that do not result in hospitalization, or development of drug dependency or drug abuse.

g. All events of possible drug-induced liver injury with hyperbilirubinaemia defined as ALT ≥ 3xULN **and** bilirubin ≥ 2xULN (>35% direct) (or ALT ≥ 3xULN and INR>1.5, if INR measured) termed 'Hy's Law' events (INR measurement is not required and the threshold value stated will not apply to patients receiving anticoagulants).

NOTE: bilirubin fractionation is performed if testing is available. If testing is unavailable, record presence of detectable urinary bilirubin on dipstick indicating direct bilirubin elevation and suggesting liver injury. If testing is unavailable and a subject meets the criterion of total bilirubin ≥ 2xULN, then the event is still reported as an SAE. If INR is obtained, include values on the SAE form. INR elevations >1.5 suggest severe liver injury.

12.3. Laboratory and Other Safety Assessment Abnormalities Reported as AEs and SAEs

Any abnormal laboratory test results (hematology, clinical chemistry, or urinalysis) or other safety assessments (e.g., ECGs, radiological scans, vital signs measurements), including those that worsen from baseline, and felt to be clinically significant in the medical and scientific judgement of the investigator are to be recorded as AEs or SAEs.

However, any clinically significant safety assessments that are associated with the underlying disease, unless judged by the investigator to be more severe than expected for the subject's condition, are **not** to be reported as AEs or SAEs.

12.4. Disease-Related Events and/or Disease-Related Outcomes Not Qualifying as SAEs

12.4.1. Pregnancy

Any pregnancy that occurs during study participation must be reported using a clinical trial pregnancy form. To ensure subject safety, each pregnancy must be reported to GSK within 2 weeks of learning of its occurrence. The pregnancy must be followed up to determine outcome (including premature termination) and status of mother and child. Pregnancy complications and elective terminations for medical reasons must be reported as an AE or SAE. Spontaneous abortions must be reported as an SAE.

Any SAE occurring in association with a pregnancy, brought to the investigator's attention after the subject has completed the study and considered by the investigator as possibly related to the investigational product, must be promptly reported to GSK.

In addition, the investigator must attempt to collect pregnancy information on any female partners of male study subjects who become pregnant while the subject is enrolled in the study. Pregnancy information must be reported to GSK as described above.

12.5. Method of Detecting AEs and SAEs

Care will be taken not to introduce bias when detecting AEs and/or SAEs. Open-ended and non-leading verbal questioning of the subject is the preferred method to inquire about AE occurrence. Appropriate questions include:

- "How are you feeling?"
- "Have you had any (other) medical problems since your last visit/contact
- "Have you taken any new medicines, other than those provided in this study, since your last visit/contact?"

12.6. Recording of AEs and SAEs

When an AE/SAE occurs, it is the responsibility of the investigator to review all documentation (e.g., hospital progress notes, laboratory, and diagnostics reports) relative to the event. The investigator will then record all relevant information regarding an AE/SAE in the appropriate data collection tool.

It is not acceptable for the investigator to send photocopies of the subject's medical records to GSK in lieu of completion of the GSK, AE/SAE data collection tool. However, there may be instances when copies of medical records for certain cases are requested by GSK. In this instance, all subject identifiers, with the exception of the subject number, will be blinded on the copies of the medical records prior to submission of to GSK.

The investigator will attempt to establish a diagnosis of the event based on signs, symptoms, and/or other clinical information. In such cases, the diagnosis will be documented as the AE/SAE and not the individual signs/symptoms.

12.7. Evaluating AEs and SAEs

12.7.1. Assessment of Intensity

The investigator will make an assessment of intensity for each AE and SAE reported during the study and will assign it to one of the following categories:

Mild: An event that is easily tolerated by the subject, causing minimal discomfort and not interfering with everyday activities.

Moderate: An event that is sufficiently discomforting to interfere with normal everyday activities.

Severe: An event that prevents normal everyday activities.

An AE that is assessed as severe will not be confused with an SAE. Severity is a category utilized for rating the intensity of an event; and both AEs and SAEs can be assessed as severe. An event is defined as 'serious' when it meets at least one of the pre-defined outcomes as described in the definition of an SAE.

12.7.2. Assessment of Causality

The investigator is obligated to assess the relationship between investigational product and the occurrence of each AE/SAE. A "reasonable possibility" is meant to convey that there are facts/evidence or arguments to suggest a causal relationship, rather than a relationship cannot be ruled out. The investigator will use clinical judgment to determine the relationship. Alternative causes, such as natural history of the underlying diseases, concomitant therapy, other risk factors, and the temporal relationship of the event to the investigational product will be considered and investigated. The investigator will also consult the Investigator Brochure (IB) and/or Product Information, for marketed products, in the determination of his/her assessment.

There may be situations when an SAE has occurred and the investigator has minimal information to include in the initial report to GSK. However, **it is very important that the investigator always make an assessment of causality for every event prior to the initial transmission of the SAE data to GSK.** The investigator may change his/her opinion of causality in light of follow-up information, amending the SAE data collection tool accordingly. The causality assessment is one of the criteria used when determining regulatory reporting requirements.

12.8. Follow-up of AEs and SAEs

After the initial AE/SAE report, the investigator is required to proactively follow each subject at subsequent visits/contacts. All AEs and SAEs will be followed until resolution, until the condition stabilizes, until the event is otherwise explained, or until the subject is lost to follow-up.

The investigator is obligated to perform or arrange for the conduct of supplemental measurements and/or evaluations as may be indicated or as requested by GSK to elucidate as fully as possible the nature and/or causality of the AE or SAE. The investigator is obligated to assist. This may include additional laboratory tests or investigations, histopathological examinations or consultation with other health care professionals. If a subject dies during participation in the study or during a recognized follow-up period, the investigator will provide GSK with a copy of any post-mortem findings, including histopathology.

New or updated information will be recorded in the originally completed data collection tool. The investigator will submit any updated SAE data to GSK within the designated reporting time frames.

12.9. Prompt Reporting of SAEs and Other Events to to GSK

SAEs, pregnancies and liver function abnormalities meeting pre-defined criteria will be reported promptly by the investigator to GSK as described in Table 14 once the investigator determines that the event meets the protocol definition for that event.

The SAE will be reported to GSK **within 24 hours**. Any follow-up information on a previously reported SAE will also be reported to GSK within 24 hours.

If the investigator does not have all information regarding an SAE, he/she will not wait to receive additional information before notifying GSK of the event and completing the appropriate data collection tool. The investigator will always provide an assessment of causality at the time of the initial report as described in Section 12.7.2, Assessment of Causality.

The primary mechanism for reporting SAEs to GSK will be the electronic data collection tool (e.g., InForm system). If the electronic system is unavailable for greater than 24 hours, the site will use the paper SAE data collection tool and fax it to the GSK Medical Monitor. Then the site will enter the serious adverse event data into the electronic system as soon as it becomes available.

After the study is completed at a given site, the electronic data collection tool (e.g., InForm system) will be taken off-line to prevent the entry of new data or changes to existing data. If a site receives a report of a new SAE from a study participant or receives updated data on a previously reported SAE after the electronic data collection tool has been taken off-line, the site can report this information on a paper SAE form or to their GSK protocol contact by telephone.

GSK contacts for SAE receipt can be found at the beginning of this protocol on the Sponsor/Medical Monitor Contact Information page.

Table 14 Reporting of SAEs, Pregnancies and Liver Function Abnormalities

	Initial Reports		Follow-up Information on a Previous Report	
Type of Event	Time Frame	Documents	Time Frame	Documents
All SAEs	24 hours	Electronic data collection tool (InForm system) "SAE" data collection tool	24 hours	Updated "SAE" data collection tool
Pregnancy	2 Weeks	Pregnancy Notification Form	2 Weeks	Pregnancy Follow up Form
Liver chemistry abnormalities Phase II:				
ALT≥3xULN and Bilirubin≥2xULN (>35% direct) (or ALT≥3xULN and INR>1.5, if INR measured)***	24 hours*	SAE data collection tool. **Liver Event Case Report Form (CRF) and liver imaging and/or biopsy CRFs if applicable	24 hours	Updated SAE data collection tool. **Updated Liver Event CRF
ALT≥5xULN; ALT≥3xULN with hepatitis or rash or 3xULN ≥4 weeks	24 hours*	**Liver Event CRF	24 hours	**Updated Liver Event CRF
ALT≥3xULN and <5xULN and biliribin <2xULN	24 hours*	**Liver Event CRF does not need completing unless elevations persist for 4 weeks or subject cannot be monitored weekly for 4 weeks		

*GSK to be notified at onset of liver chemistry elevations to discuss subject safety.
** Liver event documents should be completed as soon as possible.

The method of detecting, recording, evaluating and follow-up of AEs and SAEs and the procedures for completing and transmitting SAE reports to GSK are provided in the SPM. Procedures for post-study AEs/SAEs are provided in the SPM.

12.10. Regulatory Reporting Requirements for SAEs

Prompt notification of SAEs by the investigator to GSK is essential so that legal obligations and ethical responsibilities towards the safety of subjects are met.

GSK has a legal responsibility to notify both the local regulatory authority and other regulatory agencies about the safety of a product under clinical investigation. GSK will comply with country specific regulatory requirements relating to safety reporting to regulatory authorities, IRBs/IECs and investigators.

Investigator safety reports are prepared for suspected unexpected serious adverse reactions according to local regulatory requirements and GSK policy and are forwarded to investigators as necessary. An investigator who receives an investigator safety report describing an SAE(s) or other specific safety information (e.g., summary or listing of SAEs) from GSK will file it with the IB and will notify the IRB/IEC, if appropriate according to local requirements.

13. LIVER CHEMISTRY FOLLOW-UP PROCEDURES

Refer to the diagram in Appendix 1 for a visual presentation of the procedures listed below.

The procedures listed below are to be followed if a subject meets the liver chemistry stopping criteria defined in Section 4.6.1:

When any of the liver chemistry stopping criteria 1-3 is met, do the following:

- **Immediately** withdraw investigational product
- Report the event to GSK **within 24 hours** of learning its occurrence
- Complete the liver event CRF and SAE data collection tool if the event also meets the criteria for an SAE. All events of ALT ≥ 3xULN **and** bilirubin ≥ 2xULN (>35% direct bilirubin) (or ALT ≥ 3xULN **and** INR>1.5, if INR measured; INR measurement is not required and the threshold value stated will not apply to patients receiving anticoagulants), termed 'Hy's Law', **must be reported as an SAE**.

NOTE: if serum bilirubin fractionation is not immediately available, study drug should be discontinued if ALT ≥ 3xULN **and** bilirubin ≥ 2xULN. Serum bilirubin fractionation should be performed if testing is available. If testing is unavailable, **record presence of detectable urinary bilirubin on dipstick**, indicating direct bilirubin elevations and suggesting liver injury.

- Complete the liver imaging and/or liver biopsy CRFs if these tests are performed
- Perform liver event follow up assessments, and monitor the subject until liver chemistries resolve, stabilize, or return to baseline values as described below.
- Withdraw the subject from the **study** (unless further safety follow up is required) after completion of the liver chemistry monitoring as described below.
- Do not re-challenge with investigational product.

In addition, for criterion 1:

- Make every reasonable attempt to have subjects return to clinic within **24 hours** for repeat liver chemistries, liver event follow up assessments (see below), and close monitoring
- A specialist or hepatology consultation is recommended
- Monitor subjects twice weekly until liver chemistries (ALT, AST, alkaline phosphatase, bilirubin) resolve, stabilize or return to within baseline values

For criteria 2 and 3:

- Make every reasonable attempt to have subjects return to clinic **within 24-72 hrs** for repeat liver chemistries and liver event follow up assessments (see below)
- Monitor subjects weekly until liver chemistries (ALT, AST, alkaline phosphatase, bilirubin) resolve, stabilize or return to within baseline values; criterion 5 subjects should be monitored as frequently as possible.

Subjects with ALT ≥3xULN ***but*** *<5xULN* ***and*** *bilirubin <2xULN, without hepatitis symptoms or rash, and who can be monitored weekly for 4 weeks*

- Notify the GSK medical monitor within 24 hours of learning of the abnormality to discuss subject safety.
- Can continue investigational product
- Must return weekly for repeat liver chemistries (ALT, AST, alkaline phosphatase, bilirubin) until they resolve, stabilize or return to within baseline
- If at any time these subjects meet the liver chemistry stopping criteria, proceed as described above
- If, after 4 weeks of monitoring, ALT <3xULN and bilirubin <2xULN, monitor subjects twice monthly until liver chemistries normalize or return to within baseline values.

For criteria 1-3, make every attempt to carry out the ***liver event follow up assessments*** *described below:*

- Viral hepatitis serology including:
    - Hepatitis A IgM antibody;
    - Hepatitis B surface antigen and Hepatitis B Core Antibody (IgM);
    - Hepatitis C RNA;
    - Cytomegalovirus IgM antibody;
    - Epstein-Barr viral capsid antigen IgM antibody (or if unavailable, obtain heterophile antibody or monospot testing);
    - Hepatitis E IgM antibody (if subject resides outside the US or Canada, or has travelled outside US or Canada in past 3 months);

- Blood sample for pharmacokinetic (PK) analysis, obtained within 24h of last dose. Record the date/time of the PK blood sample draw and the date/time of the last dose of investigational product prior to blood sample draw on the CRF. If the date or time of the last dose is unclear, provide the subject's best approximation. If the date/time of the last dose can not be approximated OR a PK sample can not be collected in the time period indicated above, **do not obtain a PK sample**. Instructions for sample handling and shipping are included in the SPM
- Serum creatine phosphokinase (CPK) and lactate dehydrogenase (LDH).
- Fractionate bilirubin, if total bilirubin≥2xULN
- Obtain complete blood count with differential to assess eosinophilia
- Record the appearance or worsening of clinical symptoms of hepatitis, or hypersensitivity, such as fatigue, nausea, vomiting, right upper quadrant pain or tenderness, fever rash or eosinophilia as relevant on the AE report form
- Record use of concomitant medications, acetaminophen, herbal remedies, other over the counter medications, or putative hepatotoxins, on the concomitant medications report form.
- Record alcohol use on the liver event alcohol intake case report form

The following are required for subjects with ALT ≥3xULN and bilirubin ≥2xULN (>35% direct) but are optional for other abnormal liver chemistries:

- Anti-nuclear antibody, anti-smooth muscle antibody, and Type 1 anti-liver kidney microsomal antibodies.
- Liver imaging (ultrasound, magnetic resonance, or computerized tomography) to evaluate liver disease.

14. STUDY CONDUCT CONSIDERATIONS

14.1. Posting of Information on clinicaltrials.gov

Study information from this protocol will be posted on clinicaltrials.gov before enrollment of subjects begins.

14.2. Regulatory and Ethical Considerations, Including the Informed Consent Process

GSK will obtain favorable opinion/approval to conduct the study from the appropriate regulatory agency in accordance with any applicable country-specific regulatory requirements prior to a site initiating the study in that country.

The study will be conducted in accordance with all applicable regulatory requirements including an U.S. IND.

The study will also be conducted in accordance with "good clinical practice" (GCP), all applicable subject privacy requirements, and, the guiding principles of the 2008 Declaration of Helsinki. This includes, but is not limited to, the following:

- IRB/IEC review and favorable opinion/approval to conduct the study and of any subsequent relevant amended documents
- Written informed consent (and any amendments) to be obtained for each subject before participation in the study
- Investigator reporting requirements (e.g. reporting of AEs/SAEs/protocol deviations to IRB/IEC)

14.3. Quality Control (Study Monitoring)

In accordance with applicable regulations including GCP, and GSK procedures, GSK monitors will contact the site prior to the start of the study to review with the site staff the protocol, study requirements, and their responsibilities to satisfy regulatory, ethical, and GSK requirements. When reviewing data collection procedures, the discussion will also include identification, agreement and documentation of data items for which the CRF will serve as the source document.

GSK will monitor the study and site activity to verify that the:

- Data are authentic, accurate, and complete.
- Safety and rights of subjects are being protected.
- Study is conducted in accordance with the currently approved protocol and any other study agreements, GCP, and all applicable regulatory requirements.

The investigator and the head of the medical institution (where applicable) agrees to allow the monitor direct access to all relevant documents.

14.4. Quality Assurance

To ensure compliance with GCP and all applicable regulatory requirements, GSK may conduct a quality assurance audit. Regulatory agencies may also conduct a regulatory inspection of this study. Such audits/inspections can occur at any time during or after completion of the study. If an audit or inspection occurs, the investigator and institution agree to allow the auditor/inspector direct access to all relevant documents and to allocate his/her time and the time of his/her staff to the auditor/inspector to discuss findings and any relevant issues.

14.5. Study and Site Closure

Upon completion or premature discontinuation of the study, the monitor will conduct site closure activities with the investigator or site staff, as appropriate, in accordance with applicable regulations including GCP, and GSK procedures.

In addition, GSK reserves the right to temporarily suspend or prematurely discontinue this study at any time for reasons including, but not limited to, safety or ethical issues or

severe non-compliance. For multicenter studies, this can occur at one or more or at all sites. If GSK determines such action is needed, GSK will discuss this with the investigator or the head of the medical institution (where applicable), including the reasons for taking such action. When feasible, GSK will provide advance notification to the investigator or the head of the medical institution, where applicable, of the impending action prior to it taking effect.

If the study is suspended or prematurely discontinued for safety reasons, GSK will promptly inform investigators or the head of the medical institution (where applicable) and the regulatory authorities of the suspension or premature discontinuation of the study and the reason(s) for the action. If required by applicable regulations, the investigator or the head of the medical institution (where applicable) must inform the IRB promptly and provide the reason for the suspension or premature discontinuation.

14.6. Records Retention

Following closure of the study, the investigator or the head of the medical institution (where applicable) must maintain all site study records, except for those required by local regulations to be maintained by someone else, in a safe and secure location. The records must be maintained to allow easy and timely retrieval, when needed (e.g., audit or inspection), and, whenever feasible, to allow any subsequent review of data in conjunction with assessment of the facility, supporting systems, and staff. Where permitted by local laws/regulations or institutional policy, some or all of these records can be maintained in a format other than hard copy (e.g., microfiche, scanned, electronic); however, caution needs to be exercised before such action is taken. The investigator must assure that all reproductions are legible and are a true and accurate copy of the original and meet accessibility and retrieval standards, including re-generating a hard copy, if required. Furthermore, the investigator must ensure there is an acceptable back-up of these reproductions and that an acceptable quality control process exists for making these reproductions.

GSK will inform the investigator of the time period for retaining these records to comply with all applicable regulatory requirements. The minimum retention time will meet the strictest standard applicable to that site for the study, as dictated by any institutional requirements or local laws or regulations, or GSK standards/procedures; otherwise, the retention period will default to 15 years.

The investigator must notify GSK of any changes in the archival arrangements, including, but not limited to, archival at an off-site facility or transfer of ownership of the records in the event the investigator leaves the site.

14.7. Provision of Study Results to Investigators, Posting to the Clincal Trials Register and Publication

Where required by applicable regulatory requirements, an investigator signatory will be identified for the approval of the clinical study report. The investigator will be provided reasonable access to statistical tables, figures, and relevant reports and will have the

opportunity to review the complete study results at a GSK site or other mutually-agreeable location.

GSK will provide the investigator with the randomization codes for their site after the statistical analysis for the entire study has been completed.

GSK will also provide the investigator with the full summary of the study results. The investigator is encouraged to share the summary results with the study subjects, as appropriate.

The results summary will be posted to the Clinical Study Register at the time of the first regulatory approval or within 12 months of any decision to terminate development. In addition, a manuscript will be submitted to a peer reviewed journal for publication no later than 12 months after the first approval or any decision to terminate development. When manuscript publication in a peer reviewed journal is not feasible, further study information will be posted to the GSK Clinical Study Register to supplement the results summary.

14.8. Data Management

GSK Data Management will identify and implement the most effective data acquisition and management strategy for each clinical trial protocol and deliver datasets which support the protocol objectives. Subject data will be entered into GSK defined CRFs and combined with data provided from other sources (e.g. diary data, laboratory data) in a validated data system. Subject initials will not be transmitted to GSK for inclusion in the datasets. Clinical data management will be performed in accordance with applicable GSK standards and data cleaning procedures with the objective of removing errors and inconsistencies in the data which would otherwise impact on the analysis and reporting objectives, or the credibility of the Clinical Study Report. Adverse events and concomitant medications terms will be coded using validated dictionaries. Original CRFs will be retained by GSK, while the investigator will retain a copy.

In all cases, subject initials will not be collected nor transmitted to GSK.

15. REFERENCES

Brazg R, Xu L., Dalla M.C, Cobelli C, Thomas K, and Stein PP. Effect of adding sitagliptin, a dipeptidyl peptidase-4 inhibitor, to metformin on 24-h glycaemic control and β-cell function in patients with type 2 diabetes. *Diabetes, Obesity, and Metabolism.* 2007;9:186-193.

Carter D, Howlett HC, Wiernsperger NF, Bailey CJ. Differential effects of metformin on bile salt absorption from the jejunum and iluem. *Diabetes, Obesity and Metabolism.* 2003;5(2):120-125.

Correia S, Carvalho C, Santos MS, Seica R, Oliveira CR, Moreira PI. Mechanisms of action of metformin in type 2 diabetes and associated complications: an overview. *Mini Review of Medical Chemistry.* 2008;Nov 8(13):1343-1354.

GlaxoSmithKline Document Number RM2008/00434/00 Study ID GSK1292263. Investigator's Brochure. Report Date 2008.

GlaxoSmithKline Document Number RM2009/00168/00 Study ID GSK1292263. Investigator's Brochure, Supplement 1. Report Date 2009.

GlaxoSmithKline Document Number RM2009/00168/01 Study ID GSK1292263. Investigator's Brochure, Supplement 2. Report Date 2009.

GLUCOPHAGE (Metformin) Product Information. January, 2009.

JANUMET (sitagliptin) Product Information. March, 2009.

JANUVIA (sitagliptin) Product Information. March, 2009.

Kirpichnikov D, McFarlane SI, Sowers JR. Metformin: an update. *Annals of Internal Medicine.* 2002;Jul 2; 137(1):25-33.

Mannucci E, Tesi F, Bardini G, Ognibene A, Petracca MG, Ciani S, Pezzatini A, Brogi M, Dicembrini I, Cremasco F, Messeri G, Rotella CM. Effects of metformin on glucagon-like-peptide-1 levels in obese patients with and without Type 2 diabetes. *Diabetes Nutrition and Metabolism.* 2004;Dec;17(6):336-342.

Natali A, Ferrannini E. Effects of metformin and thiazolidinediones on suppression of hepatic glucose production and stimulation of glucose uptake in type 2 diabetes: a systematic review. *Diabetologia.* 2006;Mar:49(3):434-41. Epup 2006 Feb 14.

Appendix 1: Liver Safety Algorithms

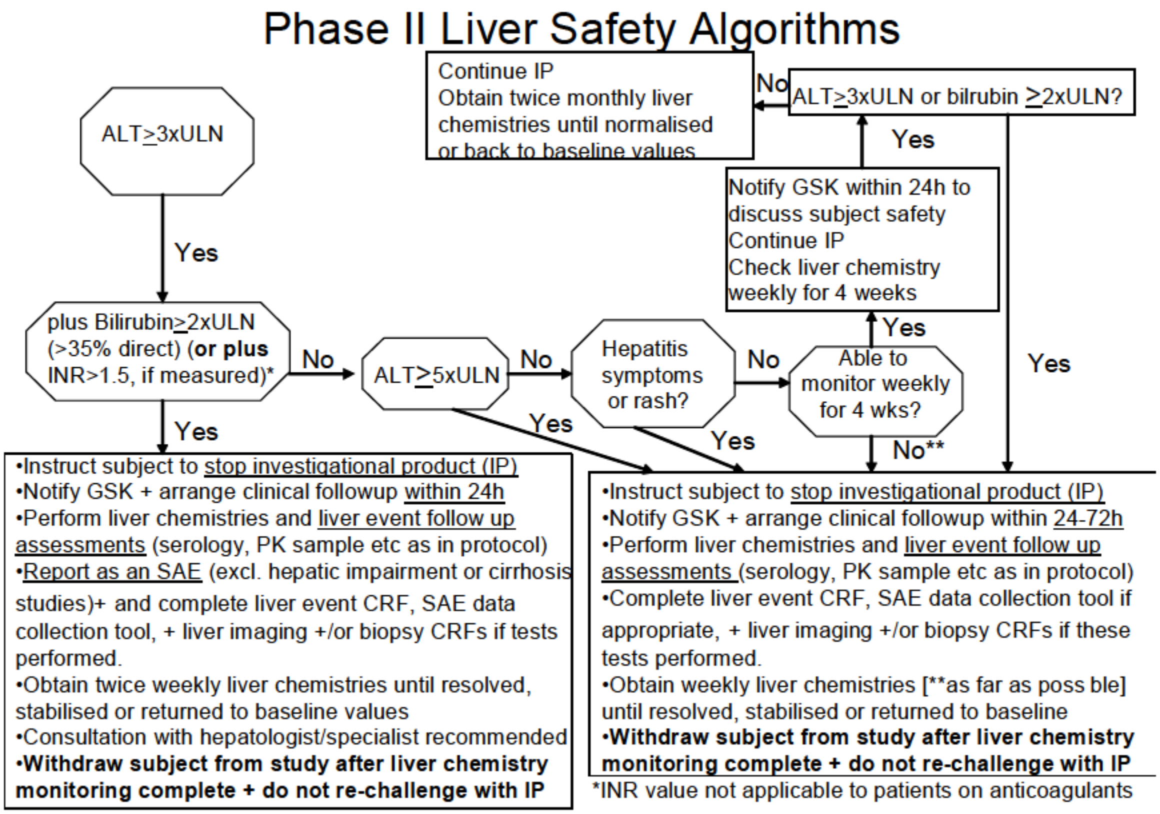

Appendix 2: Pharmacogenetic research

Pharmacogenetics - Background

Pharmacogenetics (PGx) is the study of variability in drug response due to hereditary factors in different populations. There is increasing evidence that an individual's genetic composition (i.e., genotype) may impact the pharmacokinetics (absorption, distribution, metabolism, elimination), pharmacodynamics (relationship between concentrations and pharmacologic effects or the time course of pharmacologic effects) and/or clinical outcome (in terms of efficacy and/or safety and tolerability). Collection of whole blood samples, even when no a priory hypothesis has been identified, may enable PGx analysis to be conducted if at any time it appears that there is a potential unexpected or unexplained variation in handling or response to GSK1292263.

Pharmacogenetic Research Objectives

The objective of the PGx research (if there is a potential unexpected or unexplained variation) is to investigate a possible genetic relationship to handling or response to GSK1292263. If at any time it appears there is potential variability in response in this clinical study or in a series of clinical studies with GSK1292263 that may be attributable to genetic variations of subjects, the following objectives may be investigated:

- Relationship between genetic variants and the pharmacokinetics of investigational product
- Relationship between genetic variants and safety and/or tolerability of investigational product
- Relationship between genetic variants and efficacy of investigational product

Informed Consent

Subjects who do not wish to participate in the PGx research may still participate in the clinical study. PGx informed consent must be obtained prior to any blood being taken for PGx research. Refusal to participate will involve no penalty or loss of benefits to which the subject would otherwise be entitled.

Study Population

Any subject who has given informed consent to participate in the clinical study, has met all the entry criteria for the clinical study, and receives investigational product may take part in the PGx research provided the subject has given consent to the specific collection of a PGx sample. Any subject who has received an allogeneic bone marrow transplant must be excluded from the PGx research.

Subject participation in the PGx research is voluntary and refusal to participate will not indicate withdrawal from the clinical study.

Study Assessments and Procedures

In addition to any blood samples taken for the clinical study, a whole blood sample (~6mL) will be collected for the PGx research using a tube containing EDTA. The PGx sample is labelled (or "coded") with a study specific number that can be traced or linked back to the subject by the investigator or site staff. Coded samples do not carry personal identifiers (such as name or social security number). The blood sample will be taken on a single occasion, unless a duplicate sample is required due to inability to use the original sample. It is recommended that the blood sample be taken at the first opportunity after a subject has been randomized and provided informed consent for PGx research, but the sample may be taken at any time during the subject's participation in the clinical study.

If deoxyribonucleic acid (DNA) is extracted from the blood sample, the DNA may be subjected to sample quality control analysis. This analysis will involve the genotyping of several genetic markers to confirm the integrity of individual samples. If inconsistencies are noted in the analysis, then those samples may be destroyed.

The need to conduct PGx analysis may be identified after a study (or a set of studies) of GSK1292263 has been completed and the study data reviewed. For this reason, samples may be kept for up to 15 years after the last subject completes the study or GSK may destroy the samples sooner. In special cases, the samples may not be studied, e.g., if there are not enough subjects, if the study is stopped for other reasons, or if no questions are raised about how people respond to GSK1292263. GSK or those working with GSK (for example, other researchers) will only work with samples collected from the study for the use stated in this protocol and in the informed consent form. Samples will be stored securely. Subjects can request their sample to be destroyed at any time.

Subject Withdrawal from Study

If a subject who has consented to participate in PGx research withdraws from the clinical study for any reason other than being lost to follow-up, the subject will be given a choice of one of the following options concerning the PGx sample, if already collected:

- PGx research continues per the subject's consent (i.e., the sample is retained); or,
- Any remaining sample is destroyed

If a subject withdraws consent from the PGx research or requests sample destruction, the investigator must request sample destruction by completing the appropriate documentation within the specified timeframe specified, and maintain the documentation in the site study records. In either case, GSK will only keep study information collected/generated up to that point.

Screen and Baseline Failures

If a blood sample for PGx research has been collected and it is then determined that the subject does not meet the entry criteria for participation in the clinical study, then the investigator must request sample destruction by completing the appropriate documentation within the specified timeframe, and maintain the documentation in the site study records.

Pharmacogenetics Analyses

The need to conduct PGx analysis may be identified after a study (or set of studies) has been completed. For this reason, samples may be kept for up to 15 years after the last subject completes the study. GSK may destroy the samples sooner.

Generally, GSK will utilize any of three approaches to explore genetic variation in drug response.

Specific genetic markers may be selected from "candidate genes" known to encode the drug target, drug metabolizing enzymes, molecules associated with mechanisms underlying adverse events (for example, molecules important for immune response), and those linked to drug response. Candidate genes that may be investigated in this study are genes from the GSK Absorption, Distribution, Metabolism and Excretion (ADME) panel. ADME genes play a central role in drug pharmacokinetics and pharmacodynamics (PK-PD). The GSK ADME panel contains genetic markers from one hundred and thirty-five enzymes, transporters and other genes involved in drug absorption, distribution, metabolism and excretion. The ADME panel may be used to investigate the relationship between genetic variants on the panel and pharmacokinetics, safety and efficacy of the investigational product.

Additional candidate genes that may be investigated in this study include, but are not limited to, the following:

- GPR119 receptor

- Genes altering response to DPP-IV inhibitors or GLP-1 analogues

- Genes altering response to metformin

In addition, continuing research may identify other enzymes, transporters, proteins, or receptors that may be involved in response to GSK1292263. The genes that may code for these proteins may also be studied.

Evaluate markers that comprise pre-defined "panels" for association with specified endpoints.

Examples of such panels include the GSK ADME (Absorption, Distribution, Metabolism, and Excretion) Panel and the GSK DILI (Drug Induced Liver Injury) Panel which consist of genetic markers from set of genes that are known to be related to pharmacokinetic, pharmacodynamic, immune, or adverse drug response.

Evaluate markers throughout the genome using a whole genome screen (WGS).

By evaluating large numbers of genetic markers (e.g., single nucleotide polymorphisms or SNPs) throughout the genome, sets of markers may be identified that correspond to differential drug response.

In all cases, appropriate statistical methods will be used to analyze the genetic markers in the context of other clinical data. The statistical methods for analysis may include, but are not limited to Hardy-Weinberg Equilibrium (HWE) Analysis, Linkage Disequilibrium Analysis, Evaluation of Genotypic Effects, Evaluation of Treatment by Genotype and Gene-Gene Interaction, Multiple Comparisons and Multiplicity, and/or Power and Sample Size Considerations. Detailed description of all analyses to be conducted will be documented in the Reporting and Analysis Plan.

Provision of Study Results and Confidentiality of Subject's PGx Data

GSK may summarize the cumulative PGx research results in the clinical study report.

In general, GSK will not inform the investigator, subject, or anyone else (e.g., family members, study investigators, primary care physicians, insurers, or employers) of the PGx research results under any circumstances unless required by law. This is because the information generated from PGx studies is preliminary in nature, and the significance and scientific validity of the results are undetermined at such an early stage of research.

Appendix 3: Hunger, Craving, and Fullness Questionnaire

	CONFIDENTIAL		Page 1
Protocol Identifier<br>GPR111598	Subject Identifier		Visit Description<br>Day __________

Hunger, Craving, and Fullness Questionnaire

This questionnaire asks you how often you were hungry, craved food, and about how full you feel when you finished meals, on average, **in the past 24-hours.**

For each question below, place a cross (✕) in the box next to the option that best describes your answer. Place a cross in only one box per question.

In the past 24 hours I was hungry

Always ☐

Often ☐

Sometimes ☐

Rarely ☐

Never ☐

In the past 24 hours I thought about food

Always ☐

Often ☐

Sometimes ☐

Rarely ☐

Never ☐

	CONFIDENTIAL		Page 2
Protocol Identifier GPR111598	Subject Identifier		Visit Description Day __________

In the past 24 hours I wanted to eat

Always ☐

Often ☐

Sometimes ☐

Rarely ☐

Never ☐

In the past 24 hours I ate more than I think I should have.

Yes definitely ☐

Yes probably ☐

I don't know ☐

Probably not ☐

Definitely not ☐

In the past 24 hours I craved specific foods

Always ☐

Often ☐

Sometimes ☐

Rarely ☐

Never ☐

	CONFIDENTIAL		Page 3
Protocol Identifier GPR111598	Subject Identifier		Visit Description Day __________

In the past 24 hours, when I finished my meals I felt

Much too full ☐

Very full ☐

Comfortably full ☐

Slightly hungry ☐

Very hungry ☐

In the past 24 hours, when I finished my meals I felt

Extremely satisfied ☐

Satisfied ☐

Neutral ☐

Dissatisfied ☐

Extremely dissatisfied ☐

Appendix 4: Protocol Amendment Changes

AMENDMENT 2

Where the Amendment Applies

This amendment applies to all sites.

Summary of Amendment Changes with Rationale

This amendment adds (i) an extra arm in Part B to evaluate the safety, tolerability, PK and PD of 600mg QD when co-administered with metformin, and (ii) apolipoprotein and CRP measurements to the fasting laboratory analyses. The addition of the QD arm will allow comparison of the PK and PD of 600mg QD GSK1292263 when administered as monotherapy and as an add-on to metformin.

List of Specific Changes

ABBREVIATIONS

ADDED TEXT

**Apo**	**Apolipoprotein**
**CRP**	**C-Reactive Protein**

Section 1.2.2.3. Part C (Ongoing)

PREVIOUS TEXT

Fifty-six subjects have been enrolled in Part C as of Jan 4, 2010.

Safety

Points of note:

- Six subjects have been withdrawn from the study.
    - [redacted]

  - [redacted]
- One additional subject was randomized but not dosed because the blood pressure and QTc parameters were outside of the protocol permitted limits.
- There have been no clinically significant AEs or changes in vital signs.
- There have been no clinically significant ECG changes, including QTc.
- There have been no clinically significant safety lab changes, other than increased blood glucose levels typical of T2DM.

REVISED TEXT

Seventy four subjects have been enrolled in Part C as of **February** 4, 2010.

Safety

Points of note:

- **Ten** subjects have been withdrawn from the study.
  - [redacted]
- One additional subject was randomized but not dosed because the blood pressure and QTc parameters were outside of the protocol permitted limits.
- There have been no clinically significant AEs or changes in vital signs **other than those indicated above.**

- There have been no clinically significant ECG changes, including QTc.
- There have been no clinically significant safety lab changes, other than increased blood glucose levels typical of T2DM.

Section 1.5.2.1. GSK1292263

PREVIOUS TEXT

Considerations for Part B Dose Selection

As outlined in the protocol, the planned doses of GSK1292263 are 75mg BID and 300mg BID. In this Amendment 1, the maximum allowable total daily dose of 600mg (300mg BID) is based on preliminary data from the 13 week toxicology study in the dog (Section 1.1.2.3 and Section 1.1.2.4).

The following factors have been considered to arrive at the doses and regimens for Part B:

- BID dosing is used to coincide with metformin dosing.
- The broadest spectrum of doses should be investigated within the safety limits available…

…The dose of GSK1292263 to be tested in Part A is 300mg and is considered adequate to characterize any potential effect of metformin on the pharmacokinetics of GSK1292263. The planned doses of GSK1292263 for Part B are 75mg BID and 300mg BID co-administered with metformin...

REVISED TEXT

Considerations for Part B Dose Selection

As outlined in the protocol, the **initial** planned doses of GSK1292263 **were** 75mg BID and 300mg BID. In ~~this~~ Amendment 1, the maximum allowable total daily dose of 600mg (300mg BID) **was** based on preliminary data from the 13 week toxicology study in the dog (Section 1.1.2.3 and Section 1.1.2.4). **In Amendment 2, a 5th arm is added to Part B to evaluate the safety, tolerability, PK and PD of 600mg QD GSK1292263 when co-administered with metformin.**

The following factors have been considered to arrive at the doses and regimens for Part B:

- BID dosing is used to coincide with metformin dosing.
- **QD dosing is added in Amendment 2 to allow comparison of the effects of 600mg QD of GSK1292263 when administered as monotherapy to T2DM subjects washed off prior anti-diabetic medications (Part C of study GPR111598) and as an add-on to metformin (Part B of this study GPR113132).**
- The broadest spectrum of doses should be investigated within the safety limits available…

…The dose of GSK1292263 to be tested in Part A is 300mg and is considered adequate to characterize any potential effect of metformin on the pharmacokinetics of GSK1292263. The planned doses of GSK1292263 for Part B are 75mg BID and 300mg BID co-administered with metformin**, and Amendment 2 adds a dose of 600mg QD co-administered with metformin…**

Section 1.6.2. Risks Related to GSK1292263

PREVIOUS TEXT

While the maximum allowable total daily dose in the current study was 800mg based on a number of impurities in the drug substance, (current IB [GlaxoSmithKline Document Number RM2008/00434/00] and Supplement2 of the IB [GlaxoSmithKline Document Number RM2009/00168/01]), this Amendment limits the maximum total daily dosage for Part B in this protocol to 600mg (300mg BID) based on the preliminary data from the 13-week toxicology study in the dog. (See Section 1.6.2 and Section 1.6.3).

REVISED TEXT

While the maximum allowable total daily dose in the current study was 800mg based on a number of impurities in the drug substance, (current IB [GlaxoSmithKline Document Number RM2008/00434/00] and Supplement2 of the IB [GlaxoSmithKline Document Number RM2009/00168/01]), ~~**this**~~ Amendment **1** limit**ed** the maximum total daily dosage for Part B in this protocol to 600mg (300mg BID) based on the preliminary data from the 13-week toxicology study in the dog. (See Section 1.6.2 and Section 1.6.3).

In Amendment 2, a 5th arm is added to Part B to evaluate the safety, tolerability, PK and PD of 600mg QD GSK1292263 when co-administered with metformin. A single dose of 800mg QD GSK1292263 was generally safe and well tolerated in Part A of study GPR111598. In addition, based on data from Part C of study GPR111598 (Table 7), the systemic exposures of GSK1292263 expected from 600mg QD GSK1292263 co-administered with metformin are anticipated to be well below the exposures achieved with 300mg BID.

Section 2.1. Primary

ADDED TEXT

- To investigate the safety and tolerability of GSK1292263 as single dose (Part A) and repeat oral BID **or QD** doses (Part B) when co-administered in subjects with T2DM already taking metformin.
- To determine the pharmacokinetic parameters of GSK1292263 in subjects with T2DM already taking metformin following single dose (Part A) and repeat oral doses administered BID **or QD** (Part B).
- To evaluate in T2DM subjects already taking metformin the pharmacodynamic effects of GSK1292263 following single dose (Part A) and repeated oral doses administered BID **or QD** (Part B), and the pharmacokinetic/pharmacodynamic relationships.

Section 3.1. Primary

ADDED TEXT

- Safety and tolerability parameters following single and repeat doses of GSK1292263 administered BID **or QD**, including adverse events, and assessments of clinical laboratory, ECGs and vital signs.
- Pharmacokinetic parameters following a single dose of GSK1292263 in Part A: Cmax, Tmax, t½, tlag, CL/F, V/F, AUC(0-24h), and AUC(0-∞). For repeat-dosing (BID **or QD**) in Part B the following PK parameters will be calculated: Cmax, Tmax, tlag (Day 1 only),AUC(0-10h) (**BID regimen only)**, AUC(0-24h), and t1/2 (Day 14 only) accumulation ratio (Ro), as data permit.

Section 4.1. Study Design/Schematic

ADDS QD ARM

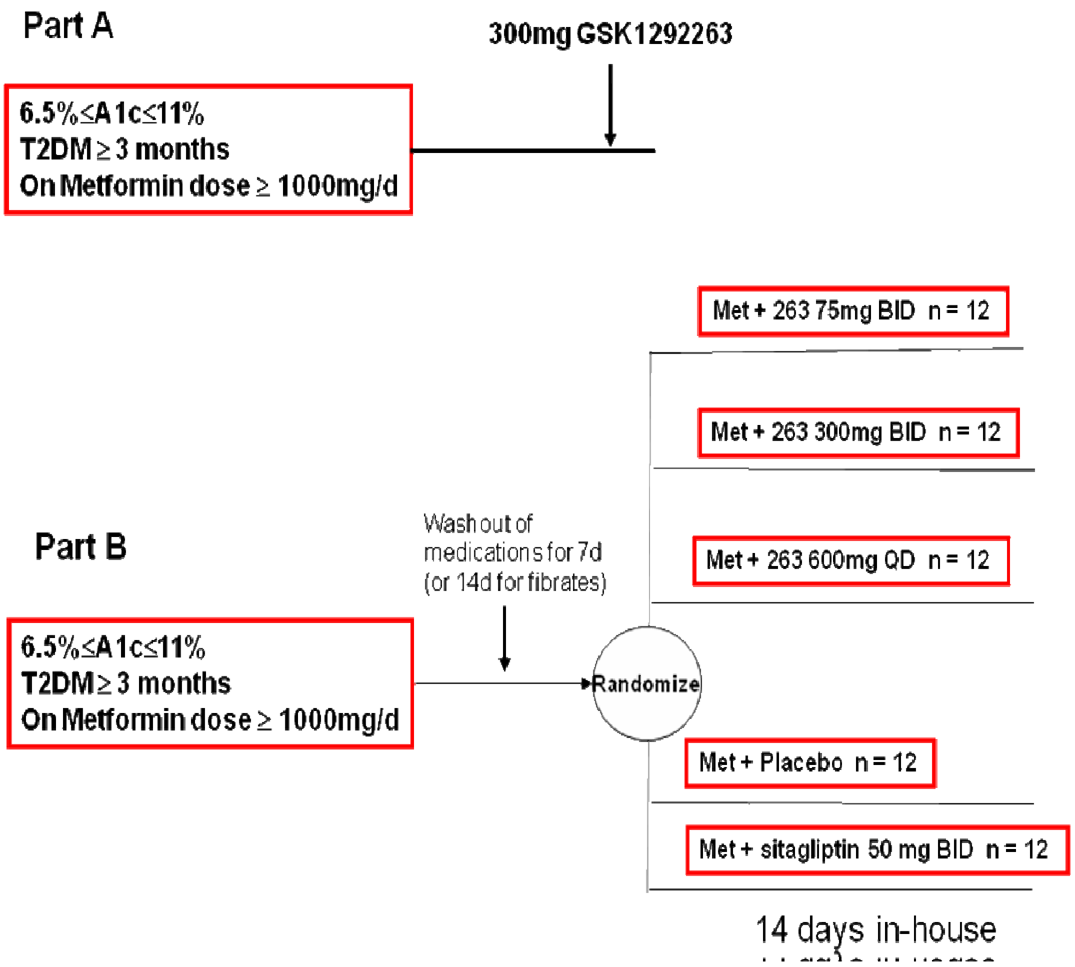

Section 4.2.2. Part B: Repeat Dosing of GSK1292263 to T2DM Subjects Taking Metformin

PREVIOUS TEXT

Part B is a single-blind, randomized, placebo-controlled, 4-arm cohort of 48 subjects dosed for 14 days with one of two doses of GSK1292263, placebo or open-label sitagliptin 50mg BID. It is being conducted to assess safety, tolerability, PK and PD of GSK1292263 and open-label sitagliptin after 14-days of dosing in T2DM subjects already taking metformin monotherapy.

The planned doses of GSK1292263 in Part B are 75mg BID and 300mg BID. Modeling and simulation indicates that doses of 75mg BID and 300mg BID will provide an estimate of the safety, tolerability, PK and PD of GSK1292263 in T2DM subjects taking metformin and will not exceed exposure limits based on non-clinical toxicology studies. These doses may be changed based on emergent safety, tolerability, PK and PD data from Part A of this study, as well as the on-going Part C of study GPR111598, but the maximum dose will not exceed 300mg BID (total daily dose of 600mg).

REVISED TEXT

Part B is a single-blind, randomized, placebo-controlled, **5**-arm cohort of **60** subjects dosed for 14 days with one of **3** doses of GSK1292263, placebo or open-label sitagliptin 50mg BID. It is being conducted to assess safety, tolerability, PK and PD of GSK1292263 and open-label sitagliptin after 14-days of dosing in T2DM subjects already taking metformin monotherapy.

The planned doses of GSK1292263 in Part B are 75mg BID, ~~and~~300mg BID, **and 600mg QD**. Modeling and simulation indicates **these doses** will provide an estimate of the safety, tolerability, PK and PD of GSK1292263 in T2DM subjects taking metformin and will not exceed exposure limits based on non-clinical toxicology studies. These doses may be changed based on emergent safety, tolerability, PK and PD data from Part A of this study, as well as the on-going Part C of study GPR111598, but the maximum **total daily** dose will not exceed **600mg** (300mg BID **or 600mg QD)**.

Section 4.2.5.2. Part B

PREVIOUS TEXT

Subjects will be randomized to 14 days of dosing with one of two dose levels of GSK1292263 (planned doses: 75mg BID and 300mg BID) or matching placebo BID, or open-label sitagliptin 50mg BID. Subjects will check into the unit on Day -2, followed by 24h PD assessments on Days -1 and 14, and PK…

REVISED TEXT

Subjects will be randomized to 14 days of dosing with one of **three dosing regimens** of GSK1292263 (doses: 75mg BID, 300mg BID **and 600mg QD**) or matching placebo

BID, or open-label sitagliptin 50mg BID. Subjects will check into the unit on Day -2, followed by 24h PD assessments on Days -1 and 14, and PK…

Section 4.3. Treatment Assignment

ADDED TEXT

In Part B, subjects were randomized to receive an active treatment (one of two dose levels of GSK1292263 BID or sitagliptin 50mg BID) or placebo BID regimen in a ratio of 1:1:1:1. **However, with the addition of GSK1292263 600mg QD arm, the ratio will change to 1:1:1:1:6 after 10 subjects per arm are randomized per the original randomization schedule. This will allow for a constant block size once the GSK1292263 600mg QD arm is added.** Appropriate stratification will be made to prevent an enrolment bias based on screening BMI ($\geq$ 30 and <30 kg/m^2).

Section 4.4. Investigational Product Dosage/Administration

ADDED TEXT

The doses in Part B are planned to be administered as 14 days of dosing in conjunction with metformin, randomized into one of 5 groups:

- **75mg GSK1292263 BID**
  as 1 x 75mg GSK1292263+ 2 placebo (AM and PM)
- **300mg GSK1292263 BID**
  as 1 x 200mg + 1 x 75mg +1 x 25mg all GSK1292263 (AM and PM)
- **600mg GSK1292263 QD**
  as 3 x 200mg GSK1292263 (AM) + 3 x Placebo (PM)
- **Placebo BID**
  as 3 x placebo tablets (AM and PM)
- **Sitagliptin**
  50mg BID (open label, unblinded)

Section 4.5. Dose Adjustment Criteria

ADDED TEXT

The planned doses in this study are 300mg as a single dose in Part A, and 75 mg BID 300mg BID**, and 600mg QD** in Part B. These doses were selected to allow for a robust characterization of safety, tolerability, PK and PD of GSK1292263 when co-administered with metformin in T2DM volunteers, while keeping within this toxicokinetics limits indicated in Section 1.1.2.4. If modification of doses is necessary, the highest total daily dose of GSK1292263 to be administered in the study will be 600mg (administered as 300mg BID **or 600mg QD**) based on preliminary data from the 13-week toxicology study in the dog (see Section 1.1.2.3 and Section 1.1.2.4).

Section 4.8. Time and Events Table (Part B)

ADDED TEXT

11. Serial blood samples for the determination of the PK of GSK1292263 will be collected on Days 1, 7 and 14. Metformin and sitagliptin concentrations will also be measured using Day 14 samples. PK sampling times may be changed based on observed PK profile, but the total number of samples will not change. Blood samples for PK will be collected on Days 1 and 14, at immediately pre-morning dose, 1, 2, 4, 6, 8, 10, 11, 12, 14, 16, 18, 24 and 48 hrs post-morning dose. On Day 7, blood samples for PK will be collected at predose (=post- breakfast), 1, 2, 4 (= pre lunch), 6 and 10 (= immediately post-dinner, predose for BID regimen).

Section 5.1. Number of Subjects

PREVIOUS TEXT

Approximately 56 subjects may be enrolled (up to 8 subjects in Part A, 48 in Part B) and complete dosing and study assessments. Additional subjects/cohorts may be enrolled to allow for evaluation of additional dose levels, with notification of IRBs.

For Part B, 12 subjects will be randomized into each of two GSK1292263 treatment arms (planned BID doses 75mg and 300mg GSK1292263), the open-label sitagliptin arm and the placebo arm.

REVISED TEXT

Approximately **68** subjects may be enrolled (up to 8 subjects in Part A, **60** in Part B) and complete dosing and study assessments. Additional subjects/cohorts may be enrolled to allow for evaluation of additional dose levels, with notification of IRBs.

For Part B, 12 subjects will be randomized into each of **3** GSK1292263 treatment arms (~~**planned**~~ BID doses 75mg and 300mg GSK1292263 **and QD dose 600mg**), the open-label sitagliptin arm and the placebo arm.

Section 6.1. Hypotheses and Treatment Analyses

ADDED TEXT

In Part B point estimates and 90% confidence intervals for the slope for ln(dose) from the analysis of GSK1292263 PK parameters [AUC(0-10) **(BID regimens only)**, AUC(0-24), AUC(0-∞) (as data permit), and Cmax following single dosing on Day 1 and AUC(0-10) **(BID regimens only)**, AUC(0-24), and Cmax following repeat dosing] will be calculated to assess dose-proportionality. Dose proportionality will also be assessed by comparing the PK parameters for each of the test doses to those for the reference dose and by visual inspection (test and reference doses to be determined). Point estimates and 90% confidence intervals will be presented for each of the differences (test-reference).

Repeat-dose AUC(0-10h) **(BID regimens only)**, AUC(0-24h) and Cmax on Days 1 and 14 will be compared to assess accumulation (as data permit). Point estimates and 90% confidence intervals will be presented for each of the comparisons.

Section 6.3.2.2. Pharmacokinetic Analyses

ADDED TEXT

Following repeat dosing in Part B:

- Cmax, tmax, tlag (Day 1 only), area under the plasma concentration-time curve over the dosing interval [AUC(0-10) **(BID regimens only)**, AUC(0-24)] and AUC(0-10) on Day 7 **(all regimens)**, pre-dose (trough) concentration at the end of the dosing interval (Cτ) (Days 4, 5, 6 and 7), and t½ (Day 14 only) will be estimated for GSK1292263, as data permit.
- Cmax, tmax, and area under the plasma concentration-time curve over the dosing interval [AUC(0-24)] on Day 14, will be estimated for sitagliptin.
- AUC(0-24h), AUC(0-10h) **(BID regimens only)**, and Cmax following dosing on Days 1 and 14 will be used for assessment of dose proportionality of GSK1292263. Trough concentration (Cτ) samples collected on Days 4, 5, 6 and 7 will be used to assess attainment of steady state for GSK1292263. To estimate the extent of accumulation after repeat dosing, the observed accumulation ratio (Ro) will be determined.

Section 7.2.4. Clinical Laboratory Assessments

ADDED TEXT

Apolipoprotein A1	Apolipoprotein B	Apolipoproteiin E (if test is available from local lab)	C-Reactive Protein

AMENDMENT 1

Where the Amendment Applies

This amendment applies to all sites.

Summary of Amendment Changes with Rationale

This amendment includes (i) preliminary safety, tolerability and PK information from Part C of study GPR111598, (ii) preliminary data from 13-week toxicology studies in the rat and dog, (iv) sets the maximum daily dose to be evaluated in Part B at 600mg, and (v) allows up to 8 subjects to be enrolled in Part A if required to define the PK of single dose GSK1292263 when co-administered with metformin.

List of Specific Changes

Section 1.1.2.3 Non-Clinical Toxicology

ADDED TEXT

Amendment No. 1 summarizes preliminary data from the 13-week toxicology studies in the dog and rat.

ADDED SECTIONS

Section 1.1.2.3.1 Preliminary Data from 13-week Toxicology Study in the Dog

In the 13-week dog study (4, 20, 1000mg/kg/day), preliminary histopathological evaluation revealed adverse GSK1292263-related changes in (i) the testes (minimal to slight tubular degeneration/depletion and Leydig cell hypertrophy) at ≥ 20mg/kg/day, and (ii) the liver (elevated ALT and GLDH in both sexes; minimal inflammation in males) at 1000mg/kg/day. There were no treatment-related findings in the liver or testes in the dog at 4mg/kg/day.

Testis:

Three of four males given 1000mg/kg/day and one male given 20mg/kg/day had minimal to mild degeneration/depletion of seminiferous epithelium in the testes. The change primarily affected spermatids in the superficial layers of the seminiferous epithelium indicating the test article effect was occurring in the later steps of the spermatogenic process while sparing the early spermatogonial stem cells. This suggests reversibility is likely to occur on cessation of treatment. However, no recovery groups were included in this study.

Liver:

Adverse GSK1292263-related changes were observed in the liver (increased ALT and GLDH in both sexes; minimal inflammation in males) at 1000mg/kg/day. No recovery groups were included on this study. Moderate to marked increases in ALT

(3.3X to 11.3X in males, 4.3X to 11.7X in females based on individual animal values relative to pre-test) and marked increases in GLDH (4.4X to 22.3X in males, 5.8X to 15X in females based on individual animal values relative to pre-test) were observed at 1000mg/kg/day, with changes increasing in severity between Week 4 and Week 13. Although minimal inflammation with a mixed inflammatory cell population was present in males given 1000mg/kg/day, it was not present in females although females had higher enzyme elevations. There was no hepatocellular necrosis evident in any of the dogs given 1000mg/kg/day so there was no clear histopathology correlate explaining the ALT and GLDH elevations.

Section 1.1.2.3.2 Preliminary Data from 13-week Toxicology Study in the Rat

In the 13-week rat study (10, 150, 2000mg/kg/day) there were no treatment-related findings in liver or testes up to 2000mg/kg/day.

Section 1.1.2.4. Toxicokinetics

Based on the results of non-clinical toxicology studies [*GlaxoSmithKline Document Number RM2008/00434/00],* exposures in the current study were not to exceed a mean steady-state AUC(0-24h) of 49,400ng.h/mL and mean Cmax of 2693ng/mL which are 80% of the gender-averaged no-observed-adverse-effect-level (NOAEL) mean exposure in beagle dogs at Day 14 at the highest dose of 1000mg/kg/day.

Furthermore, no individual was to exceed an AUC(0-24h) of 72,700ng.h/mL or Cmax of 3585ng/mL, which are 80% of the highest AUC and Cmax observed in dog at 1000mg/kg NOAEL dose group in the 14 day toxicology study.

These exposures were consistent with limits that were approved for the conduct of the previous studies, GPR111956 and GPR111598, by the GSK Global Safety Board and the local IRBs.

Section 1.1.2.4.1 Toxicokinetic Parameters from the 13-Week Toxicology Studies in Rat and Dog

The GSK1292263 concentrations in these studies are shown in Table 1.

Table 1 Mean Systemic Exposures Following Oral Administration of GSK1292263 in the Rat and Dog 13-week studies

Duration	Dose (mg/kg/day)	Sex	C_{max} (ng/mL)		AUC_{0-24} (ng.h/mL)	
			1st TK	End of Study[1]	1st TK	End of Study[1]
Rat (13 week)	10	M	488	395	4429	3702
		F	712	995	7002	10868
	150	M	1696	1081	22016	16285
		F	2390	2991	33178	42869
	2000	M	3394 (2899-3684)	2543 (2332-2755)	55601 (49322-63416)	43551 (30628-56474)
		F	6779 (5818-7372)	5749 (4977-6521)	92517 (83695-103185)	89976 (82895-97057)
Dog (13 week)	4	M	870	1087	14034	17517
		F	702	969	9541	12860
	20	M	2100 (1824-2467)	2573 (1885-3145)	32536 (28785-38282)	43504 (27838-59195)
		F	1716 (1402-1956)	2377 (2237-2478)	23153 (20103-25929)	36930 (32808-39672)
	1000	M	5091 (2801-6846)	5453 (2757-7304)	86890 (38952-124997)	107751 (48863-144523)
		F	5323 (4857-5816)	5743 (4628-6900)	94643 (81316-108038)	106938 (89998-124774)

1. First TK was Day 14 in rat and Day 1 in dog. End of study was Day 87 for rat and Day 90 for dog.

The AUC(0-24h) and Cmax associated with the no effect threshold for testicular findings in the dog were 36,772ng.h/mL and 2,537ng/mL, respectively, following 13 weeks of dosing. The lowest individual AUC(0-24h) and Cmax for the dog testicular effect were 48,863ng.h/mL and 2,757 ng/mL, respectively, following 13 weeks of dosing..

Protocol Amendment 1 now limits the maximum total daily dose in this study to 600mg (300mg BID) based on the results of the 13 week toxicology study in the dog (Section 1.1.2.3.1). A dose of 300mg BID, or a dosing regimen resulting in equivalent exposure, given for up to 14 days does not pose a significant clinical risk from the perspective of either mean or individual exposures to date (see summary of human pharmacokinetics in Section 1.2.2.3).

It is important to note that there were no treatment-related effects in liver or testes in the rat and dog up to 2000mg/kg/day and 1000mg/kg/day GSK1292263, respectively, at mean systemic exposures of 58,405ng·hr/mL (male rats) and 49,505ng·hr/mL (male dogs) on Day 14 of the 14-day toxicology study. In addition, stage-dependent qualitative evaluation of spermatogenesis demonstrated normal progression up to the limit dose in both species indicating there were no subtle morphological changes in the testes following 14 days of dosing. In the 13-week rat

study (10, 150, 2000mg/kg/day) there were no treatment-related findings in liver or testes up to 43,551ng.hr/mL (mean male rat AUC0-24hr).

Section 1.2.2 First Time in T2DM Subjects

ADDED SECTIONS

Section 1.2.2.3 Part C (Ongoing)

Fifty-six subjects have been enrolled in Part C as of Jan 4, 2010.

Safety

Points of note:

- **Six subjects have been withdrawn from the study.**
    - **Subject 1201: Wide QRS complex present pre-dose and in a previous** [redacted]
    - [redacted]
    - [redacted]
    - [redacted]
    - [redacted]
    - [redacted]
- **One additional subject was randomized but not dosed because the blood pressure and QTc parameters were outside of the protocol permitted limits.**
- **There have been no clinically significant AEs or changes in vital signs.**
- **There have been no clinically significant ECG changes, including QTc.**
- **There have been no clinically significant safety lab changes, other than increased blood glucose levels typical of T2DM.**

Pharmacokinetics

The maximum GSK1292263 exposures observed in the GPR111598 study have been associated with steady-state during the on-going Part C. The final doses of GSK1292263 selected in Part C were 50mg BID, 150mg BID, 300mg BID and 600mg QD. A summary of preliminary exposures of GSK1292263 (n=6) at steady-state (Day 13 and Day 14) are presented in Table 7 (n=5-7 in each group).

Table 7 Summary of Preliminary GSK1292263 Exposure at Steady-State in Part C of study GPR111598

Dose	Day 13		Day 14	
	AUC(0-24h) (ng.h/mL) Mean ±SD [min, max]	Cmax (ng/mL) Mean ±SD [min,max]	AUC(0-24h) (ng.h/mL) Mean ±SD [min, max]	Cmax (ng/mL) Mean ±SD [min, max]
50mg BID	7944 ± 1362 [5357, 9203]	429 ± 58 [396, 536]	8153 ± 1240 [5758, 9331]	452 ± 53 [369, 515]
150mg BID	16986 ± 5863 [12449, 27137]	985 ± 312 [691, 1517]	17257 ± 5656 [12789, 26895]	952 ± 304 [717, 1479]
300mg BID	23101 ± 7900 [15606, 35959]	1258 ± 376 [912, 1885]	23810 ± 8517 [15585, 38771]	1222 ± 343 [863, 1834]
600mg QD	9330 ± 1847 [6555, 11964]	866 ± 145 [653, 1098]	10221 ± 2519 [7333, 13870]	795 ± 207 [601, 1221]

The maximum observed group mean AUC(0-24h) and Cmax values to date are 23,810ng.h/mL and 1,258ng/mL, respectively. The maximum individual AUC(0-24h) and Cmax values to date are 38,771ng.h/mL and 1,885ng/mL, respectively. Of note, (i) the highest exposures have been observed in the 300mg BID group, and (ii) there is no significant effect of sitagliptin on GSK1292263 exposures in these T2DM subjects.

1.2.2.3.1. PK Simulations based on available Part C data

PK simulations were performed using preliminary data from Part C (Table 7, n=5-7 in each group) to characterize the dose/exposure relationship for GSK1292263 in T2DM subjects and relate them to the toxicokinetic data from the 13 week dog study.

The probabilities of the steady state exposure in a T2DM subject exceeding the testicular no-effect threshold (AUC(0-24h) = 36,772 ng.h/mL and Cmax = 2,537 ng/mL) are shown in Table 8 and the probabilities of the steady state exposure in a T2DM subject exceeding the testicular effect threshold (AUC(0-24h) = 48,863 ng.h/mL and Cmax = 2,757 ng/mL) are shown in Table 9.

Table 8 Probability of exceeding no-effect threshold for Testicular Findings

Treatment	No Effect Cmax, ng/mL	No Effect AUC(0-24h), ng.h/mL
50 mg BID	~0%	~0%
150 mg BID	0.15%	0.7%
300 mg BID	1.1%	5.9%
600 mg QD	~0%	~0%

Table 9 Probability of exceeding exposures associated with Testicular Findings

Treatment	Effect Cmax, ng/mL	Effect AUC(0-24h), ng.h/mL
50 mg BID	~0%	~0%
150 mg BID	0.2%	0.1%
300 mg BID	1.1%	0.8%
600 mg QD	~0%	~0%

Table 8 indicates that there is a small probability (<6%) that an AUC will exceed the no-effect threshold and Table 9 shows that there is a probability of <1% that an AUC will exceed the lowest exposure associated with the testicular effect in dogs following 13 weeks of dosing.

Section 1.5.2 Dose Rationale

PREVIOUS TEXT

Considerations for Part B Dose Selection

As outlined in the protocol, the planned doses of GSK1292263 are 75mg BID and 300mg BID with the option to adjust doses up to a maximal total daily dose of 800mg. The maximum allowable dose is based on the presence of a starting material impurity, GSK2116107 (See Section 1.6.2 for additional details).

REVISED TEXT

Considerations for Part B Dose Selection

As outlined in the protocol, the planned doses of GSK1292263 are 75mg BID and 300mg BID. **In this Amendment 1, t**he maximum allowable **total daily** dose **of 600 mg (300 mg BID)** is based on **preliminary data from the 13 week toxicology study in the dog (Section 1.1.2.3 and Section 1.1.2.4).** ~~**the presence of a starting material impurity, GSK2116107. and three hydroperoxide impurities (GSK2233661A, GSK2266332A, GSK2233664A) that have been identified in the drug substance (Section), and on preliminary data from the 13 week toxicology study in the dog (Sections and).**~~

Section 1.5.2.3 Sitagliptin

PREVIOUS TEXT

This is the recommended dose of sitagliptin when co-administered with metformin for the treatment of T2DM.

REVISED TEXT

This is the recommended dose of sitagliptin when co-administered with metformin for the treatment of T2DM **in subjects without contraindications**.

Section 1.6.2 Risks Related to GSK1292263

ADDED TEXT

Amendment 1 allows up to 8 subjects to complete study assessments in Part A, if required to define the PK of single dose GSK1292263 when co-administered with metformin.

PREVIOUS TEXT

As indicated in Supplement 2 of the IB (GlaxoSmithKline Document Number RM2009/00168/01), the maximum allowable total daily dose in the current study is 800mg (administered as single or divided doses) because the drug substance used in this study may contain a trichloromethyloxadiazole process impurity, GSK2116107A, which was mutagenic in a mini-Ames test. Quantitative assessments indicate that GSK2116107A is not present in the final drug substance at a limit of detection of 50ppm (which based on a clinical dose of 800mg/day, equates to a total oral dose of less than 40μg/day). There are no safety concerns for the conduct of a clinical trial at doses up to 800mg/day for up to 28 days because the level of this impurity is below the staged TTC (Threshold of Toxicological Concern) limit of 60μg/day for clinical trials of up to one month duration.

Three hydroperoxides impurities (GSK2233661A, GSK2266332A, GSK2233664A) have been identified in the drug substance. Two of the three hydroperoxides (GSK2233661A and GSK2233664A) were mutagenic at concentrations ranging up to 500μg (limited by

precipitation) per plate in the presence or absence of S9-mix. Quantitative assessments indicate that the total combined content of all three hydroperoxides was 2.5ppm in the final drug substance (which, based on a clinical dose of 800mg/day, equates to a total oral dose of 4µg/day). There are no safety concerns for the conduct of a clinical trial up to 800mg/day because the levels of these impurities are well below the staged TTC limit of 60µg/day for clinical trials of up to one month duration.

REVISED TEXT

~~As indicated in Supplement 2 of the IB (GlaxoSmithKline Document Number [RM2009/00168/01]), the maximum allowable total daily dose in the current study is 800mg (administered as single or divided doses) because the drug substance used in this study may contain a trichloromethyloxadiazole process impurity, GSK2116107A, which was mutagenic in a mini-Ames test. Quantitative assessments indicate that GSK2116107A is not present in the final drug substance at a limit of detection of 50ppm (which based on a clinical dose of 800mg/day, equates to a total oral dose of less than 40µg/day). There are no safety concerns for the conduct of a clinical trial at doses up to 800mg/day for up to 28 days because the level of this impurity is below the staged TTC (Threshold of Toxicological Concern) limit of 60µg/day for clinical trials of up to one month duration.~~

~~Three hydroperoxides impurities (GSK2233661A, GSK2266332A, GSK2233664A) have been identified in the drug substance. Two of the three hydroperoxides (GSK2233661A and GSK2233664A) were mutagenic at concentrations ranging up to 500µg (limited by precipitation) per plate in the presence or absence of S9-mix. Quantitative assessments indicate that the total combined content of all three hydroperoxides was 2.5ppm in the final drug substance (which, based on a clinical dose of 800mg/day, equates to a total oral dose of 4µg/day). There are no safety concerns for the conduct of a clinical trial up to 800mg/day because the levels of these impurities are well below the staged TTC limit of 60µg/day for clinical trials of up to one month duration.~~

While the maximum allowable total daily dose in the current study was 800mg based on a number of impurities in the drug substance, (current IB [GlaxoSmithKline Document Number RM2008/00434/00] and Supplement2 of the IB [GlaxoSmithKline Document Number RM2009/00168/01]), this Amendment limits the maximum total daily dosage for Part B in this protocol to 600mg (300mg BID) based on the preliminary data from the 13-week toxicology study in the dog. (See Section 1.1.3.2 and Section 1.1.3.3).

ADDED SECTIONS

Section 1.6.2.1. Risk Assessment based on the Preliminary Observations from the 13-week Toxicology Studies in Rats and Dogs

There were no treatment-related effects in liver or testes in the rat and dog up to 2000mg/kg/day and 1000mg/kg/day GSK1292263, respectively, at systemic mean exposures of 58,405ng·hr/mL (male rats) and 49,505ng·hr/mL (male dogs) on Day 14 of the 14-day toxicology study. In addition, stage-dependent qualitative evaluation

of spermatogenesis demonstrated normal progression up to the limit dose in both species indicating there were no subtle morphological changes in the testes following 14 days of dosing in the 14-day toxicology study. In the 13-week rat study (10, 150, 2000mg/kg/day) there were no treatment-related findings in liver or testes up to 43,551ng.hr/mL (mean male rat AUC0-24hr).

For single dose administration of GSK1292263 in clinical subjects, preclinical data support mean clinical exposures up to 49,400ng.hr/mL (as described in the current Investigator Brochure, [GlaxoSmithKline Document Number RM2008/00434/00]).

Testis: In the ongoing clinical trials there are no means to readily monitor for the testicular effect observed in the 13-week dog study. The mean clinical AUC achieved at 300 mg BID (23,810ng.h/mL), the highest intended dose, is below the no-effect AUC for the testicular effect in any dog in the 3-month study (36,772ng.h/mL), and is less than 50% of the mean no-effect AUC in the dog 14-day study (49,505ng.h/mL).

The highest individual clinical AUC achieved at 300mg BID (38,771ng.h/mL) is well below the highest exposure achieved in an individual dog at the NOAEL following 14 days of dosing (78,414ng·hr/mL), and is also less than the lowest individual dog AUC for a testicular effect following 3 months of dosing (48,863ng.h/mL). Although 38,771ng.mL does exceed the highest individual no-effect exposure in the 3-month dog study (36,772ng.h/mL), the dog testicular change is regarded as a time-dependant effect and the intended clinical dose duration is 1/6 of the duration that caused testicular toxicity (14 versus 90 days) in a single species.

Liver: There are safety margins based on systemic exposure (AUC) for the liver effects observed in the 13-week dog study 4.5-fold at the effect dose of 1000mg/kg/day (107,344ng.hr/mL); 1.7-fold at the no-effect dose of 20mg/kg/day (40,217ng.hr/mL), compared to the clinical AUC at 300 mg BID) (Table 11). Importantly, liver effects are readily monitorable and liver function monitoring and withdrawal criteria are already in place in this study.

In summary, the doses of GSK1292263 being evaluated in Part B of this study (top BID dose limited to 300mg BID, or a dosing regimen resulting in equivalent exposure) do not pose a significant clinical risk from the perspective of either mean or individual exposures to date.

Section 1.6.2.2 Overall Assessment of GSK1292263 Risk

Overall, the potential risk to subjects who receive GSK1292263 is considered low because:

- **The likelihood that plasma exposures will reach levels of toxicological concern is small based on prior data from healthy volunteers and T2DM subjects (see Section 1.2.1 and Section 1.2.2). Furthermore, PK data from Part A of the current study will be used to refine the exposure simulations that may be observed in Part B with the planned doses of GSK1292263 (75mg BID and 300mg BID).**

- **So far, GSK1292263 has been generally well tolerated in healthy subjects at doses ≤ 400mg and T2DM subjects at single doses up to 800mg and multiple doses up to 300mg BID, with no clinically significant changes in vital signs, ECGs, telemetry and lab parameters related to the study drug.**
- **The subjects will be dosed in the clinical unit and observed closely for 24h for the single dose and remain in house for two weeks during the dosing period with appropriate monitoring of clinical status, labs, vital signs, etc to decrease risk.**
    - **There is no indication that the mechanism of action of this drug would predict an increased risk of hypoglycemia in subjects with T2DM, and this was confirmed in Parts A and B of study GPR111598 in T2DM subjects.**
- **The drug substance purity levels comply with current guidelines for short-term studies.**

Section 4.2.1 Part A: Assessment of Single Dose PK of GSK1292263 in T2DM Subjects Taking Metformin

ADDED TEXT

Amendment 1 allows up to 8 subjects to complete study assessments in Part A, if required to define the PK of single dose GSK1292263 when co-administered with metformin.

Section 4.2.2 Part B: Repeat Dosing of GSK1292263 to T2DM Subjects Taking Metformin

PREVIOUS TEXT

… but the maximum dose will not exceed 400mg BID (total daily dose of 800mg).

REVISED TEXT

… but the maximum dose will not exceed 4 **3**00mg BID (total daily dose of ~~8~~ **6**00mg).

Section 4.2.7.4 Exploratory Biomarkers

ADDED TEXT

Details of PD sample collection and processing are found in the SPM. The timings of all the PD collections may be adjusted on the basis of emerging PK or PD data from this study or other new information in order to ensure optimal evaluation of the PD endpoints.

Section 4.5 Dose Adjustment Criteria

PREVIOUS TEXT

The planned doses in this study are 300mg as a single dose in Part A and 75mg BID and 300mg BID in Part B. These doses were selected to allow for a robust characterization of

safety, tolerability, PK and PD of GSK1292263 when co-administered with metformin in T2DM volunteers, while keeping within this toxicokinetics limits indicated in Section 1.1.2.4. If modification of doses is necessary, the highest daily dose of GSK1292263 to be administered in the study will be 800mg (administered as BID doses), as indicated in Section 1.6.2.

REVISED TEXT

The planned doses in this study are 300mg as a single dose in Part A and 75 mg BID and 300mg BID in Part B. These doses were selected to allow for a robust characterization of safety, tolerability, PK and PD of GSK1292263 when co-administered with metformin in T2DM volunteers, while keeping within this toxicokinetics limits indicated in Section 1.1.2.4. If modification of doses is necessary, the highest **total** daily dose of GSK1292263 to be administered in the study will be ~~**800mg**~~ **600mg (administered as 300mg BID) based on preliminary data from the 13-week toxicology study in the dog (see Section 1.1.2.3 and Section 1.1.2.4)**~~**administered as BID doses), as indicated in Section 1.6.2**~~**.**

DELETED TEXT

- ~~**No dose will be administered that is projected to exceed: (i) for the cohort as a whole, a mean plasma AUC(0-24) 49,400ng.h/mL and mean Cmax of 2693ng/mL, which is 80% of the gender-averaged NOAEL mean exposure in beagle dogs at Day 14 at the highest dose of 1000mg/kg/day, or (ii) for an individual subject an AUC of 72,700ng.h/mL and Cmax of 3585ng/mL, which is 80% of the highest AUC and Cmax observed in dog at 1000mg/kg NOAEL dose group. These exposure limits were approved for the conduct of the FTIH study (GPR111596) and the repeat-dose study (GPR111598) by the GSK Global Safety Board and the local IRB.**~~

PREVIOUS TEXT

…so as not to exceed 800mg total daily dose of GSK1292263…

REVISED TEXT

…so as not to exceed **a 600mg** total daily dose of GSK1292263…

Section 5.1 Number of Subjects

PREVIOUS TEXT

Approximately 52 subjects will be enrolled (4 in Part A, 48 in Part B) and complete dosing and study assessments. Additional subjects/cohorts may be enrolled to allow for evaluation of additional dose levels, with notification of IRBs.

REVISED TEXT

Approximately **56** subjects **may** be enrolled (**up to 8 subjects** in Part A, 48 in Part B) and complete dosing and study assessments. Additional subjects/cohorts may be enrolled to allow for evaluation of additional dose levels, with notification of IRBs.

Section 7.4.1 Blood Sample Collection

DELETED TEXT

Blood samples for pharmacokinetic analysis of GSK1292263, sitagliptin (Part B only) and metformin ~~**(Part B only)**~~ will be collected…

Section 7.4.2 Sample Analysis

DELETED TEXT

Concentrations of GSK1292263, sitagliptin (Part B only) and metformin ~~**(Part B only)**~~ will be determined in plasma samples…

Section 9.3 Prohibited Medications

ADDED TEXT

The use of anti-diabetic agents **(other than metformin)** is reason for exclusion…

Section 11.1 Blinding

ADDED TEXT

Sitagliptin **and metformin** will be administered in this study in an unblinded manner.

Section of 11.5 Treatment of Investigational Product Overdose

PREVIOUS TEXT

The maximum dose planned for this study is 800mg. For this study, any dose of GSK1292263 greater than 800mg ingested in less than a 24 hour time period will be considered an overdose.

REVISED TEXT

The maximum **total daily** dose planned for this study is **600mg**. For this study, any **total daily** dose of GSK1292263 greater than **600mg** ingested in less than a 24 hour time period will be considered an overdose.
